# Detecting Large Chromosomal Modifications Using Short Read Data From Genotyping-by-Sequencing

**DOI:** 10.3389/fpls.2019.01133

**Published:** 2019-09-24

**Authors:** Jens Keilwagen, Heike Lehnert, Thomas Berner, Sebastian Beier, Uwe Scholz, Axel Himmelbach, Nils Stein, Ekaterina D. Badaeva, Daniel Lang, Benjamin Kilian, Bernd Hackauf, Dragan Perovic

**Affiliations:** ^1^Institute for Biosafety in Plant Biotechnology, Julius Kuehn Institute, Quedlinburg, Germany; ^2^Research Group Bioinformatics and Information Technology, Leibniz Institute of Plant Genetics and Crop Plant Research (IPK), Gatersleben, Germany; ^3^Research Group Genomics of Genetic Resources, Leibniz Institute of Plant Genetics and Crop Plant Research (IPK), Gatersleben, Germany; ^4^Laboratory of Genetic Basis of Plant Identification, Vavilov Institute of General Genetics, Russian Academy of Sciences, Moscow, Russia; ^5^PGSB, Helmholtz Center Munich, Neuherberg, Germany; ^6^Global Crop Diversity Trust, Bonn, Germany; ^7^Institute for Breeding Research on Agricultural Crops, Julius Kuehn Institute, Quedlinburg, Germany; ^8^Institute for Resistance Research and Stress Tolerance, Julius Kuehn Institute, Quedlinburg, Germany

**Keywords:** genebank, crop wild relatives, characterization and utilization of plant genetic resources, translocation, copy number variation (CNV), coverage, bioinformatics, breeding

## Abstract

Markers linked to agronomic traits are of the prerequisite for molecular breeding. Genotyping-by-sequencing (GBS) data enables to detect small polymorphisms including single nucleotide polymorphisms (SNPs) and short insertions or deletions (InDels) that can be used, for instance, for marker-assisted selection, population genetics, and genome-wide association studies (GWAS). Here, we aim at detecting large chromosomal modifications in barley and wheat based on GBS data. These modifications could be duplications, deletions, substitutions including introgressions as well as alterations of DNA methylation. We demonstrate that GBS coverage analysis is capable to detect *Hordeum vulgare/Hordeum bulbosum* introgression lines. Furthermore, we identify large chromosomal modifications in barley and wheat collections. Hence, large chromosomal modifications, including introgressions and copy number variations (CNV), can be detected easily and can be used as markers in research and breeding without additional wet-lab experiments.

## Introduction

Due to the progress in DNA sequencing, collections of plant species can be compared at the genome-level to analyze the diversity within the collection. However, these analyses often resort to small differences of a few bases in the genome, as for instance, single nucleotide polymorphisms (SNPs), and seldom look at large chromosomal modifications of several kb or Mb.

In contrast, introgressions from crop wild relatives are substitutions or additions of large chromosomal regions and have been used to improve crop plants (Zamir, 2001; Dempewolf et al., 2017), e.g., as source of resistance or tolerance to biotic and abiotic stress in wheat (Rabinovich, 1998; Crespo-Herrera et al., 2017). Experimental methods, such as C-banding (Friebe et al., 1996), dot-blot genomic hybridization (Rey and Prieto, 2017), fluorescence *in-situ* hybridization (FISH) (Rayburn and Gill, 1986; Schneider et al., 2005), genomic *in-situ* hybridization (GISH) (Le et al., 1989; Schwarzacher et al., 1989) and acid or SDS-PAGE (Milovanović et al., 1998), are the state-of-the-art wet-lab techniques for detection and characterization of introgressions. However, these techniques are sophisticated and can only be handled by few labs. If specific markers are available, PCR-based methods can also be used to detect well-known introgressions (Ko et al., 2002).

Furthermore, some pipelines using bioinformatics were proposed in the last years. SNP data from GBS were used to identify introgressions provided that donor and parent plants are known (Wendler et al., 2014; Wendler et al., 2015). Alternatively, an introgression was identified by analyzing the coverage of whole-genome-sequencing data in tomato (Causse et al., 2013). In addition, Liu et al. (2004) found that foreign DNA introgression into a plant genome can induce extensive alterations in DNA methylation, which has been observed earlier in animals (Heller et al., 1995). DNA methylation was also discussed in a wider context of genomic immunity in plants with respect to transposons silencing (Kim and Zilberman, 2014). Hence, methods using methylation-sensitive restriction enzymes might be able to identify genomic regions harboring alien introgressions. Methods like amplified fragment-length polymorphism (AFLP) using a methylation-sensitive restriction enzyme (Xu et al., 2000) or standard GBS could be used for such analyses. In contrast to these methods, a huge set of different sequencing methods has been established to identify DNA methylation (Kurdyukov and Bullock, 2016)

Furthermore, large chromosomal modifications might be CNVs including duplication and deletions, which were especially identified in gene clusters (Boycheva et al., 2014). Exemplary, tandem and segmental duplications have been reported to be important for the distribution of genes involved in plant disease resistance (Leister et al., 1998; Leister, 2004; Himmelbach et al., 2010) and are of increased interest for breeding. Lu et al. (2015) developed a first approach to map the presence/absence of GBS tags genetically and incorporated these into genome-wide association scans in maize.

Here, we investigate whether it is possible to identify large chromosomal modifications from short read GBS data. Thereby, we do not utilize SNPs that are rare compared to the number of sequenced bases. Furthermore, such an approach would not use SNP calling, filtering and imputation saving runtime and avoiding artifacts that might possibly be introduced during these steps (Li, 2014). The idea of using coverage data of GBS for detecting large deletions in soybean after fast neutron mutagenesis has been presented by Lemay et al. (2019) at the Plant and Animal Genome Conference, San Diego 2018. Here, we present a bioinformatics approach that is able to detect large chromosomal modifications using standard GBS coverage data and demonstrate its applicability for barley and wheat. Furthermore, we demonstrate that this method is also able to detect large chromosomal modifications, e.g., introgression and CNV, if no pedigree or data from the parent plants is available.

## Materials and Methods

### Plant Material and Genotyping-by-Sequencing

Barley and wheat collections were analyzed for this study. While GBS data of barley collections was publicly available, a diverse winter wheat collection was compiled from European elite winter wheat cultivars and 209 genebank accessions from the Federal *ex situ* Genebank for Agricultural and Horticultural Plant Species of Germany, maintained at the Leibniz Institute of Plant Genetics and Crop Plant Research (IPK) in Gatersleben, which hosts one of the largest barley and wheat germplasm collections in the world (Knüpffer, 2009). Genebank accessions were selected using the normalized rank products for plant height, flowering time and thousand grain weight yielding eight contrasting groups (Keilwagen et al., 2014).

Single seed descended (2× SSD) wheat plants were grown in soil under greenhouse conditions to three leave seedling stage. DNA extraction for GBS analysis followed previously published protocols (Milner et al., 2018). Genomic DNA was digested with *PstI* and *MspI* (New England Biolabs) and processed for GBS library construction essentially as described previously (Wendler et al., 2014). Barcoded samples were pooled in an equimolar manner and sequenced on the Illumina HiSeq2500 device, using a custom sequencing primer (Wendler et al., 2014) according to the manufacturer’s instructions (Illumina).

### NGS Preprocessing and Coverage Analysis

Adapter and quality trimming of barley GBS raw reads was performed using Trim Galore (https://github.com/FelixKrueger/TrimGalore, version 0.4.0, non-default parameters: — quality 30 — length 50). Subsequently, these trimmed reads were mapped to the barley reference genome (Mascher et al., 2017) using BWA mem (version 0.7.12) and default parameters (Li, 2013).

Obtained wheat GBS raw read pairs were adapter trimmed using cutadapt (version 1.9.1, non-default parameters: -m 30 -a AGATCGGAAGAGC) (Martin, 2011) and mapped to the bread wheat reference genome sequence (IWGSC, 2018) using BWA mem (version: 0.7.13, non-default parameters: -M -v 3) (Li, 2013). GNU parallel (version: 20150222) was applied to all of these steps for multi-threading purposes (Tange, 2011).

Using a custom Java script, the genome was divided in non-overlapping windows of equal size *w* = 500,000bp. For each window, the number of high-quality mapped reads starting in this window was counted. More specifically, mapped reads were filtered for being the primary alignment, possessing a minimal mapping quality with at least PHRED score 20, and being not overclipped with at least 30 bases. Only reads passing these filters were counted. Subsequently, these counts were normalized by dividing them by the number of reads passing the filter (and multiplying with 1E6 for convenience) and denoted as normalized count *c* *_i_* of window *i*.

The normalized counts are highly variable along the chromosomes with higher values close to the telomers and smaller values close to the centromers. Hence, these counts need to be compared to some reference $\vec{r}=\left(r_{1},...,r_{N}\right)$ If a specific reference is given as, for instance, the counts of one of the ancestors $\vec{a}$, it can be used directly $\left(\vec{r}=\vec{a}\right)$ However, there are also cases where the ancestors or the normalized counts of the ancestors are unknown. In such cases, we compute the reference value *r* *_i_* as the median of normalized counts of all samples.^1^ Given a reference value *r* *_i_*, the ratio:

$$d_{}=\log_{2}\left(\frac{c_{i}+\varepsilon }{r_{i}+\varepsilon }\right)$$

was defined for *i* ∈ [1, *N*], where $\varepsilon =\frac{1E6}{N}$ is the number of expected reads per interval, which is used to dampen the noise in the measurement. Furthermore, a rolling average with window size 5 was used for each sample and each chromosome to denoise the signal and obtain a denoised profile ${\vec{p}}_{s}$ for each sample *s*. Finally, we determine outliers in the profile ${\vec{p}}_{s}$ of each sample *s* separately using the scores method of the R package outliers with type MAD and a probability of 99.99% (Komsta, 2011; R Core Team, 2018). Longer stretches of outliers were determined as a continuous sequence of at least 3 windows, whereby only one of two consecutive windows may not be an outlier.

### Ontology Term Enrichment

In order to study the functional composition of the genes affected by the CNVs, we compared the respective genomic regions to the coordinates of the high-confidence (HC) gene set of the latest barley (IBSC PGSB V1.0) and wheat (IWGSC V1.1) genome annotation releases. The resulting protein-coding HC gene loci where then mapped to the Gene and Plant Ontology annotations for barley and wheat (release v1.0; https://github.com/PGSB-HMGU/ontology_annotataions). Enrichment of specific ontology terms among the given genes sets was tested using the “Parent-Child-Union” algorithm implemented in the Ontologizer software (Grossmann et al., 2007) using all annotated wheat genes as a references and applying multiple testing correction of p-values using the Benjamini-Hochberg method (p < 0.01).

The year and country of origin was manually curated using several online databases using variant identifiers, names, breeders and geographical information to match genotypes to database entries collecting the earliest registration dates with the German and European Plant Varieties Office or the earliest date of collection registered in the IPK and CIMMYT database. German genotypes were grouped into eight decades ranging from 1940 to 2020. We excluded the 1980s because our dataset only comprised one line from this period. All other periods are represented by at least 8 genotypes. To account for the in part large difference in number of genotypes, for ontology term enrichment analysis we discarded loci that were represented in less than 75% of the lines per decade. To compare functional enrichment across decades, we performed Ontologizer analyses comparing the unique loci for each decade to those showing CNVs in genotypes from the other decades.

### Validation

Genomic DNA was extracted from young leaf tissues according to Paris and Carter (2000). Wheat genotypes carrying the 1AL.1RS or 1BL.1RS wheat-rye translocation were detected based on rye insertion site based polymorphism (ISBP) markers ora3, ora16 and ora17 (Bartoš et al., 2008), a primer set tagging the rye ω-secalin gene (Shimizu et al., 1997) as well as the STS marker IA-294 (Mago et al., 2004). Rye genome specific repetitive sequence pSc20H (Ko et al., 2002) was used to derive a primer pair (forward primer: 5’ ATT TCA TGC CGA AGG AGA TG 3’, reverse primer: 5’ ACT CGT TGT TCC CAA AGG TG 3’) for a universal detection of rye chromatin in wheat. Amplification of the 612bp fragment was conducted in 35 cycles using an annealing temperature of 55°C. The wheat marker UMN19 (Liu et al., 2008) was used as a positive control.

### Rye SNP Calling and Genetic Distance

Preprocessed GBS reads of ten winter wheat genotypes, which carry at least the short arm of rye chromosome 1 (1RS), were mapped against a combined genome of rye (Bauer et al., 2017) and wheat (IWGSC, 2018) using BWA mem (version 0.7.15-r1140) and default parameters (Li, 2013). Subsequently, SNP calling was performed using samtools mpileup (version 1.2) and default parameter except output-tags DP and DPR (Li, 2011). Finally, SNPs were filtered to have minimum SNP quality 100, minimum GT quality 5, minimum 4 reads per sample as well as to be bi-allelic, to be called in all 10 genotypes, and to be located on rye chromosome 1R.

### Mapping of Functional Markers

Based on sequences of publicly available primer pairs (Liu et al., 2012), the location of functional markers on the wheat chromosome was determined using blastn (Altschul et al., 1990). The sequences were blasted against the wheat reference genome using blastn (version: 2.2.29+, non-default parameter: -evalue 100.0). The results were filtered to match the expected chromosome, primers pointing to each other, and about the expected length of the fragment to be amplified.

## Results

### Analysis of Barley Introgression Lines

Since the 1990s, a limited number of *H. vulgare*/*H. bulbosum* introgression lines has been generated that harbor segments introgressed from *H. bulbosum* and show for a diverse set of desirable traits (cf. https://www.cwrdiversity.org/). Wendler et al. (2015) analyzed 146 of such introgression lines and compared the location of detected introgressed regions based on cytological analysis or SNPs derived from GBS data. Most of these introgression lines are the offspring of barley cultivars with available GBS data. In addition, three introgression lines were based on intermediate crosses where no GBS data was available. Here, these introgression lines were reanalyzed using GBS coverage analysis on publicly available GBS data.

**Figure 1:**
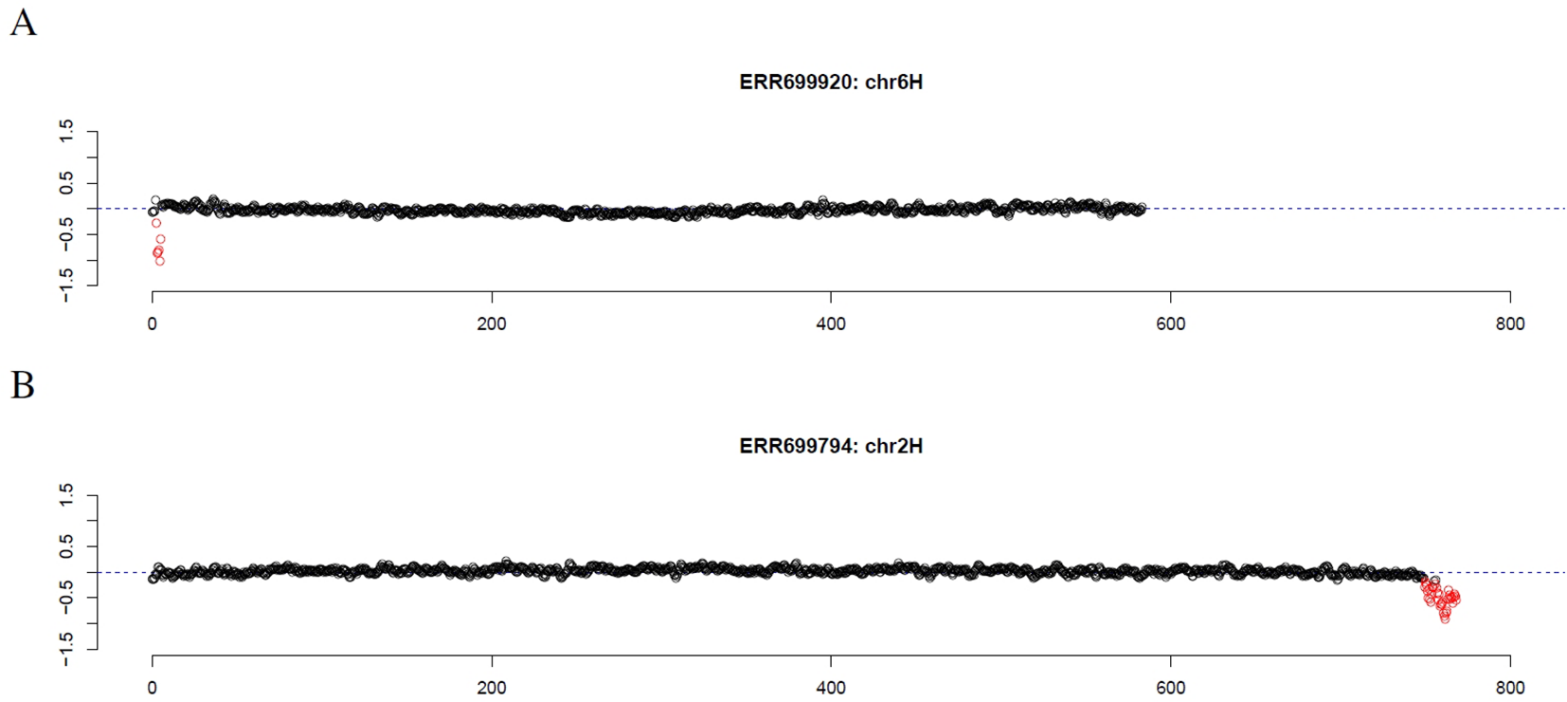
GBS coverage profiles of two introgression lines **(A)** ERR69920 (chromosome 6H) and **(B)** ERR366794 (chromosome 2H). The x-axis depicts the position within the chromosome in Mb, while the y-axis depicts normalized coverage. Each dot visualizes the denoised coverage value of a non-overlapping 500kb window, while the dashed line depicts the expectation. Dots are depicted in red if they are marked as outliers indicating large chromosomal modifications.

First, introgression lines were analyzed where GBS data of the corresponding parental barley cultivar was available. Comparing the location of the longest stretch of outliers to the location of the introgression described by cytological investigation and SNP analysis of GBS data (Wendler et al., 2015), an overlap of 92% and 96% was observed, respectively (**Supplemental Table 1**, **Supplemental Data Sheet 1**). Scrutinizing the results, introgression lines were checked that had no overlap between the location of the longest stretch of outliers and the location of the introgression as determined by SNP analysis. For introgression lines 38 (ERR699829) and 107 (ERR699893) that are supposed to have an introgression on chromosome 2HS and 3HS, respectively, GBS coverage analysis did not detect any outlier. For introgression line 60 (ERR699850) that is supposed to have an introgression on chromosome 1HL and 7HL, the longest stretch of outliers was observed on chromosome 2HS that showed an increased GBS coverage. However, another stretch of outliers can also be detected on chromosome 1HL with decreased GBS coverage. For introgression line 66 (ERR699856) that is supposed to have an introgression on chromosome 2HL and 6HL, the longest stretches of outliers were observed on chromosome 3HL and 7HL. Additional individual outliers have been detected on chromosome 2HL and 6HL. For introgression line 118 (ERR699902) that is supposed to have an introgression on chromosome 1HL, the longest stretch of outliers was observed on chromosome 3HL that shows an increased GBS coverage. However, another stretch of outliers can also be detected on chromosome 1HL with decreased GBS coverage. Interestingly, the GBS analysis was able to detect the introgression on chromosome 6HS in introgression line 137 (ERR699920, **Figure 1A**, **Supplemental Data Sheet 1**), which was missed by SNP analysis, but detected by cytological analysis.

Summarizing these observations, we can state that GBS coverage analysis detected outliers that correlated well with known introgressions. About 89% of the detected outliers had negative denoised profile values *p* *_s,i_*. Hence, the coverage of the outliers was often decreased compared to the reference (**Supplemental Data Sheet 1**).

Second, all introgression lines were analyzed using the median of coverage values of the barley cultivars Emir, Golden Promise, Morex, and Vada as reference to test whether the median reference performs similar to an ancestor reference. When comparing the location of the longest stretch of outliers to the location of the introgression described by cytological investigation and SNP analysis of GBS data, an overlap of 84% and 87% was detected (**Supplemental Table 1**). Although the GBS reference coverage profile was not based on the corresponding parental barley cultivar, the detection of the location of introgressions by the longest stretch of outliers was still high. Considering also smaller outlier stretches, the overlap between the location of introgressions and the detected outliers can be increased again.

**Figure 2:**
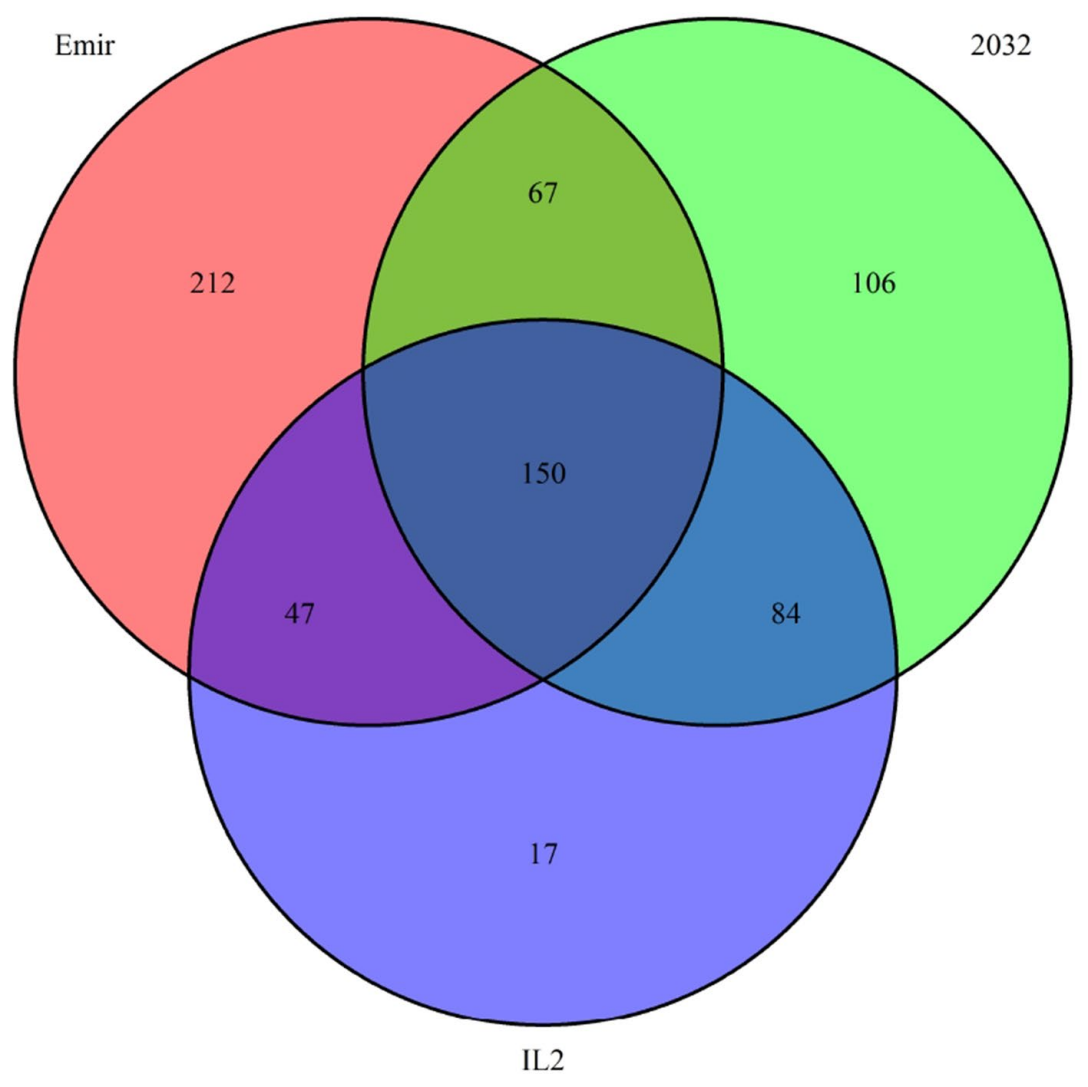
Venn diagram of loci with a combined coverage of at least 10 reads in the GBS data of the barley cultivar Emir, the *H. bulbosum* accession 2,032, and the introgression line 2. The numbers indicate the numbers of loci where at least one read has been observed.

Finally, we investigated the reason for the decreased GBS coverage within introgressed regions. Exemplarily, the introgressed region in introgression line 2 (ERR699794) was investigated. Introgression line 2 is based on a cross between the barley cultivar Emir (ERR699939) and the *H. bulbosum* accession 2032 (ERR699945) and harbors an introgression on chromosome 2HL. GBS coverage analysis using Emir as reference identified the region 749.5–768.5 Mb on chromosome 2H (**Figure 1B**, **Supplemental Table 1**). In this region, 683 loci were identified that provided a starting point for the identified GBS fragments and had a combined coverage of at least 10 reads (**Figure 2**). Coverage for Emir was observed at 476 loci, but only 217 out of these 476 loci had coverage in the *H. bulbosum* accession 2,032. This observation indicated differences between the barley cultivar Emir and the *H. bulbosum* accession either on sequence or on methylation level. However, only 150 out of these 217 loci had coverage in the introgression line 2 indicating a putative additional methylation of the corresponding loci in the introgression line compared to the donor *H. bulbosum* accession 2,032.

### Analysis of Barley Genebank Collection

Recently, almost the complete barley collection of the Federal *ex situ* Genebank for Agricultural and Horticultural Plant Species of Germany was genotyped using GBS and SNP markers were detected and associated with several plant traits (Milner et al., 2018). Here, we reanalyzed this comprehensive data set (PRJEB23967 and PRJEB24563) looking for large chromosomal modifications.

After read mapping, filtering and counting reads per genomic window, 86 barley accessions with less than 100,000 GBS reads were discarded yielding 21,319 barley accessions for further analysis. The median of the reads passing the filter per sample was about 517,000, while for the introgression lines investigated above this value was more than twice as high with about 1,129,000 reads. For this reason, only large consecutive stretches of at least 30 outliers were investigated trying to identify potential chromosomal modifications and to avoid artificial outliers based on low coverage. These long outlier stretches correspond to at least 15 Mb in the genome.

**Figure 3:**
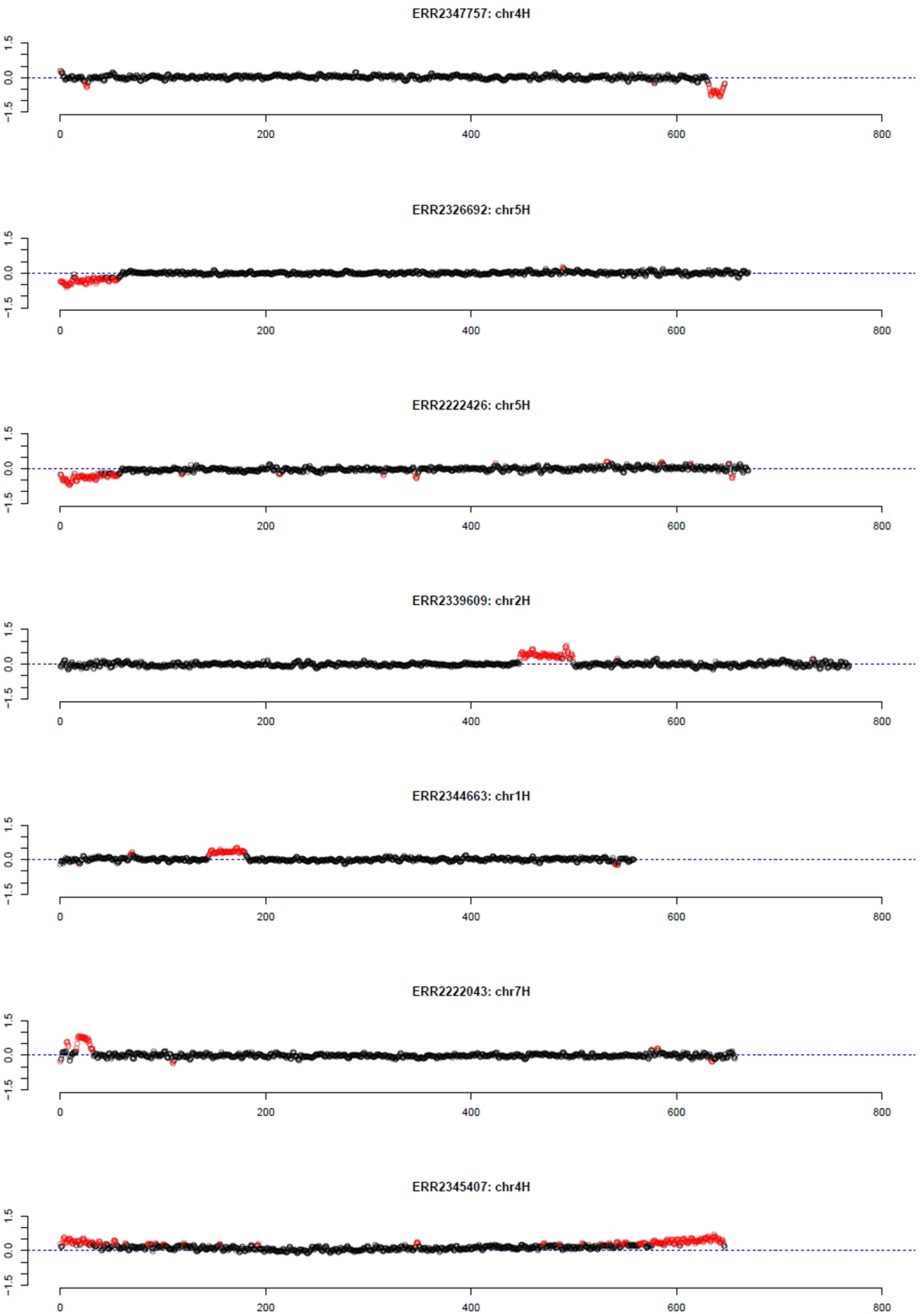
Outliers of GBS coverage data for barley collection of the German Genebank. The x-axis depicts the position within the chromosome in Mb, while the y-axis depicts normalized coverage. Each dot visualizes the denoised coverage value of a non-overlapping 500 kb window, while the dashed line depicts the expectation. Dots are depicted in red if they are marked as outliers indicating large chromosomal modifications.

As pedigree information was not available for all of these barley accessions, the median was used as reference. Using the filter for long outlier stretches, seven accessions could be identified (**Table 1**, **Figure 3**). Three of these accessions, namely HOR 685, HOR 7537, and BCC 213, showed a region with decreased GBS coverage on chromosome 4HL and 5HS. Another three accessions, namely HOR 16589, HOR 16951, and BCC 722, showed a region with increased GBS coverage on chromosome 2HL, 1HS, and 7HS, respectively. In contrast, the accession HOR 19592 shows a pattern of increased coverage on both telomers of chromosome 4H.

**Table 1:** Barley accessions with patterns of large chromosomal modifications from the Federal *ex situ* Genebank for Agricultural and Horticultural Plant Species of Germany.

Run	IPK	Year	Reads	Largest outlier stretch	Type
ERR2347757	HOR 685	1942	668,587	chr4H:631.5-647M	Low
ERR2326692	HOR 7537	1976	735,506	chr5H:15-44M	Low
ERR2222426	BCC 213	1983	847,792	chr5H:0-39.5M	Low
ERR2339609	HOR 16589	2003	480,694	chr2H:447.5-488.5M	High
ERR2344663	HOR 16951	2003	1,028,784	chr1H:145-180.5M	High
ERR2222043	BCC 722	2004	476,162	chr7H:16-32M	High
ERR2345407	HOR 19592	2003	600,495	chr4H:604-646M	Chromosome

The first column contains the run ID from EMBL-EBI ENA, the second column contains the ID from the genebank at IPK, the third column contains the year of acquisition in the genebank, the fourth column contains the number of GBS reads that passed the filters, and the fifth column contains the longest stretch of outliers indicating the largest chromosomal modification detected. Finally, column six contains the type of modification, where ‘low’ indicates decreased coverage, ‘high’ indicates increased coverage and ‘chromosome’ indicates a modification over the complete chromosome.

**Figure 4:**
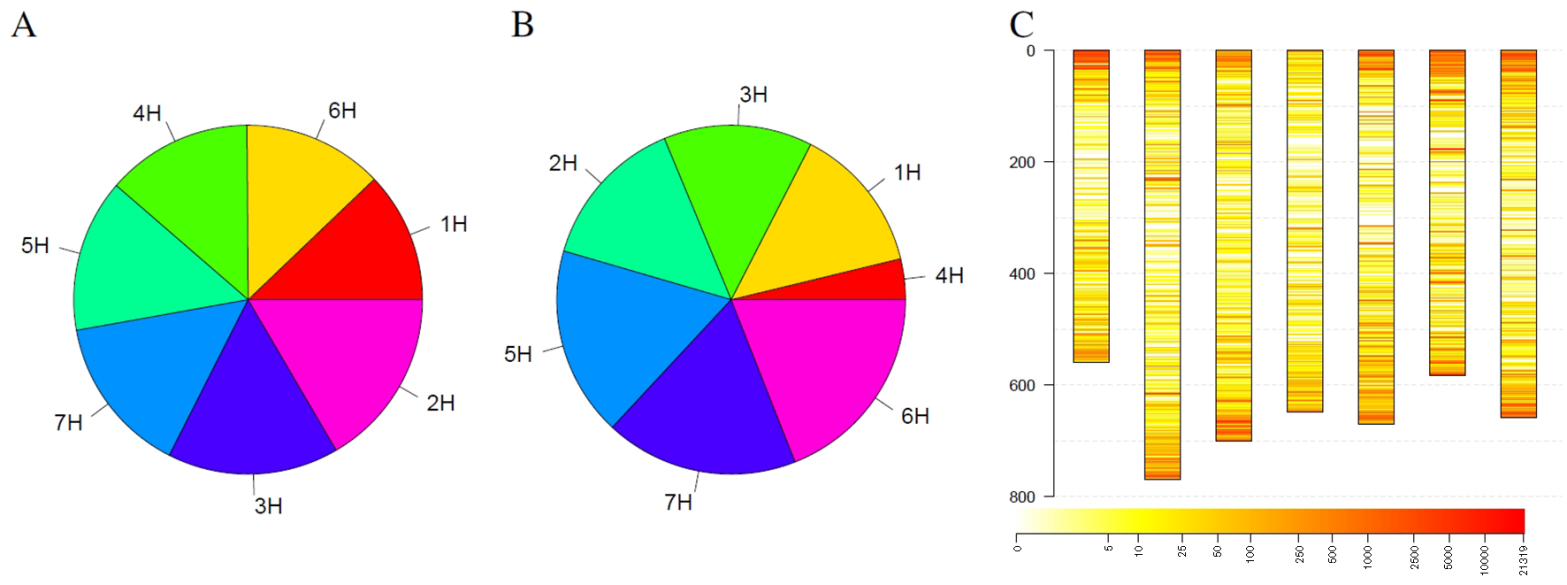
Statistics of outliers per chromosome for the barley genebank collection comprising 21,319 genotypes. **(A)** shows the statistics for all outliers, while **(B)** depicts the outliers that were detected in at least 3% of the genotypes. **(C)** depicts the spatial distribution of the outliers along the chromosomes (in Mb) blue along the y-axis and their frequency. Heat colors are used to visualize the frequency of outliers on a logarithmic scale where white indicates no barley genotype with an outlier and red indicates many barley genotypes with an outlier at this locus.

In addition to individual coverage profiles, summarizing information for the complete collection were visualized in **Figure 4**. Considering all detected outliers, all chromosomes harbor a similar number of outlier loci (**Figure 4A**). Restricting the outlier loci only to those that have been detected in at least 3% barley genotypes, the picture slightly changes. Chromosomes 6H, 7H, and 5H harbor together more than 50% of such robust outlier loci, while chromosome 4H harbors the lowest number of such loci (**Figure 4B**). Looking at the spatial distribution in the complete genome, outliers are more frequent at the telomeres compared to centromers (**Figure 4C**). Furthermore, several thin, reddish lines were recognizable along the chromosomes indicating for robust, tight loci with changed coverage.

**Figure 5:**
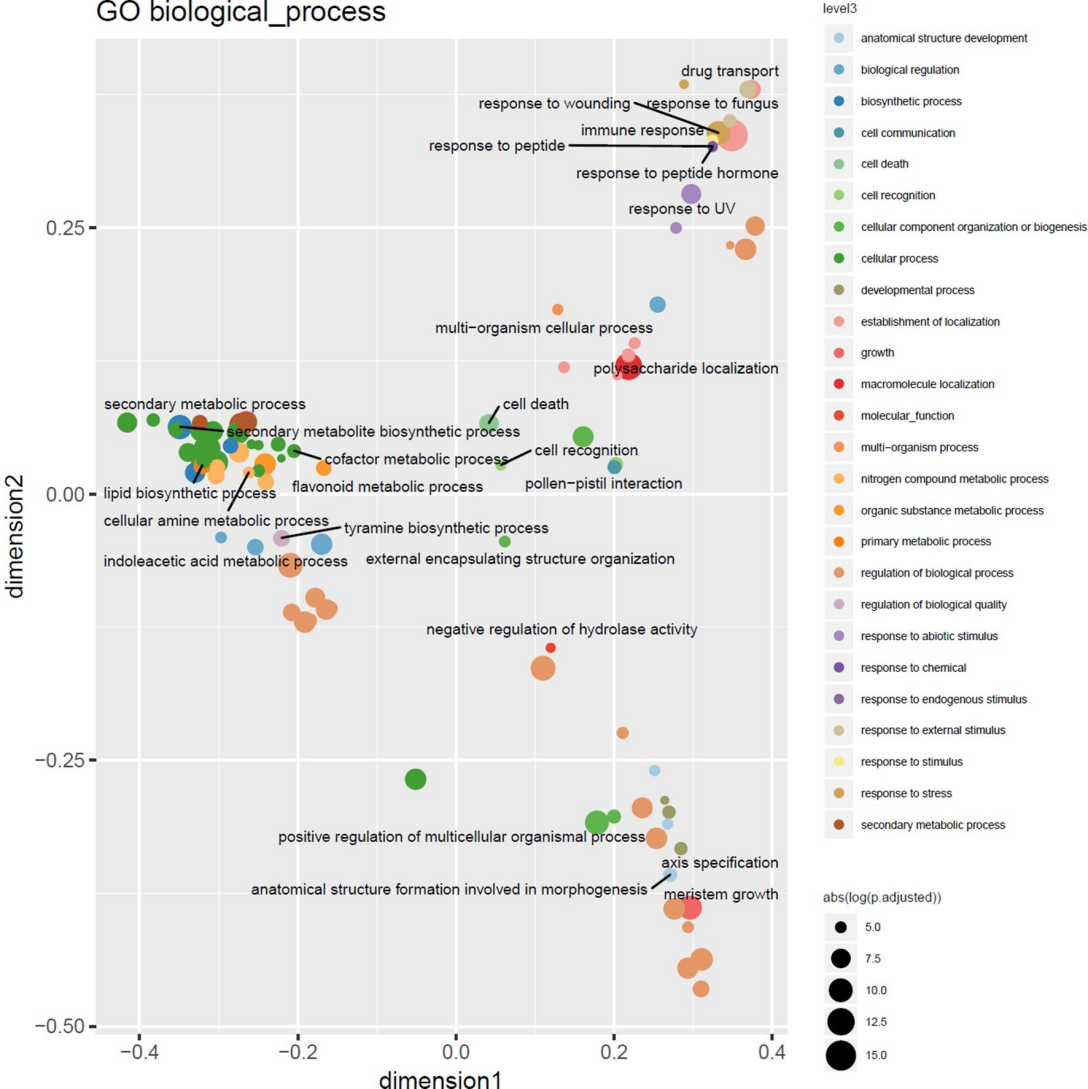
Multidimensional scaling overview plot using semantic similarities of significantly enriched GO biological process categories in the barley 3% outlier CNV regions. Points are color coded according to parental terms at depth 3 of the “biological process” ontology subgraph (level3). Size is relative to the absolute log-transformed FDR values. Labels are given for the least specific terms (by information content) of each level3 subcategory.

Highly consistent with the results of a previous study targeting CNVs in 14 barley genotypes using Comparative Genomic Hybridization arrays (Muñoz-Amatriaín et al., 2013), functional enrichment analysis using Gene and Plant Ontology annotations of outlier loci detected in at least 3% of the barley genotypes displayed enrichment of genes involved in cell death and immune response, particularly the defense and recognition of fungi and oomycetes using receptor like kinases (RLKs) with consistent major hotspots e.g. in the subtelomeric regions on 7H. Nevertheless, as depicted in **Figure 5** the increased resolution in terms of number of genotypes and ontology annotation now enables a more fine-grained picture of the gene functions modulated by CNVs in these lines comprising response to stimuli, cellular metabolic processes, and regulatory processes (**Supplemental Table 2**).

### Analysis of Elite Cultivars and Genebank Accessions of Wheat

A diverse winter wheat collection was single seed descended twice and genotyped using GBS. As pedigree information is partially unknown and ancestors of most wheat genotypes were not included in the collection, GBS coverage analysis was performed using the median as reference.

**Figure 6:**
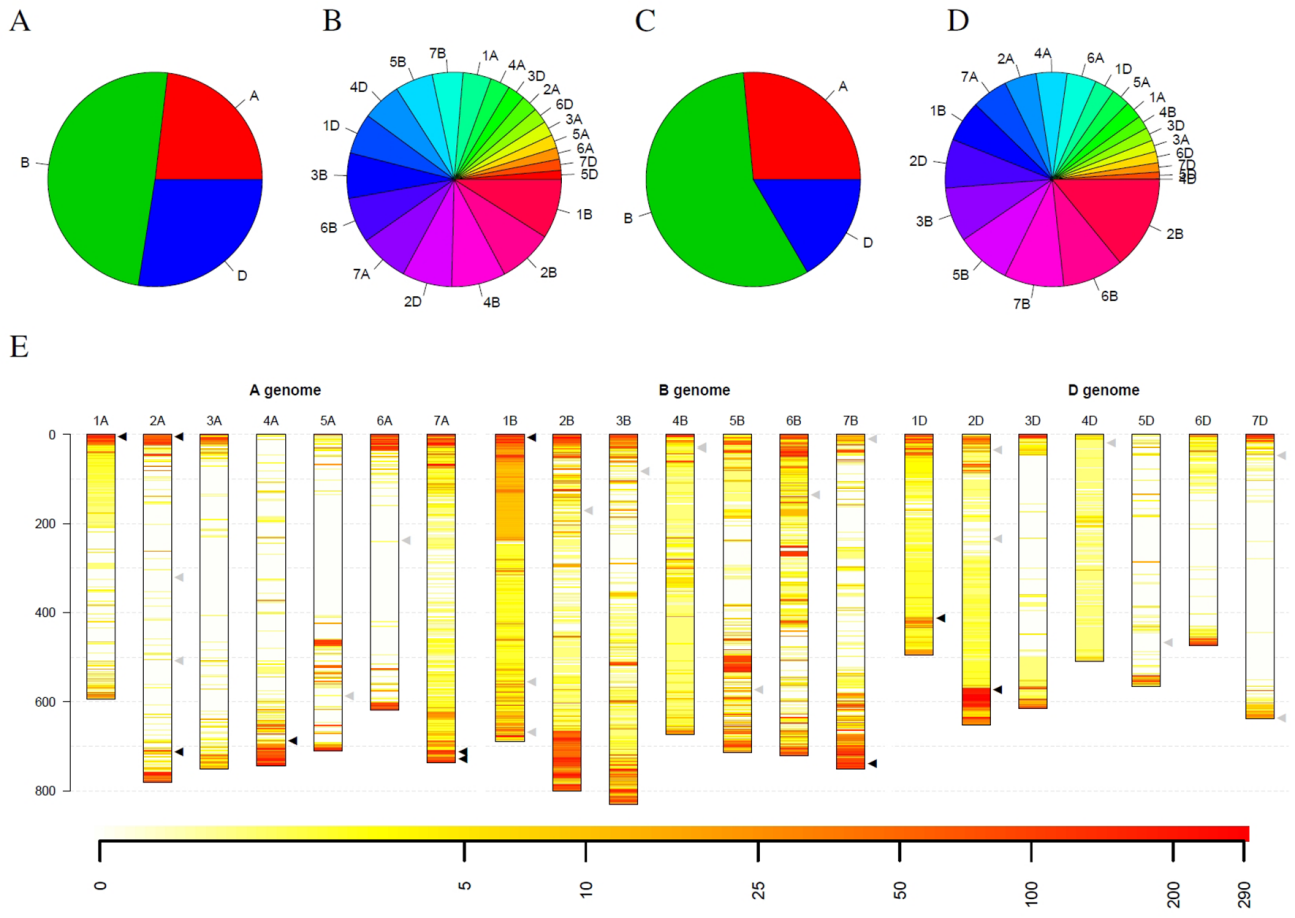
Statistics of outliers per genome and chromosome for the winter wheat collection comprising 290 genotypes. **(A)** and **(B)** show the statistics for all outliers, while **(C)** and **(D)** depict the outliers that were detected in at least 10% of the genotypes. **(E)** depicts the spatial distribution of the outliers along the chromosomes (in Mb) and their frequency. Heat colors are used to visualize the frequency of outliers on a logarithmic scale where white indicates no wheat genotype with an outlier and red indicates many wheat genotypes with an outlier at this locus. Triangles indicate genes with interest for breeding. Black triangles indicate genes that are located in regions with many outliers within the collection, while gray triangles indicate genes in regions with a low number of outliers.

Statistics for the detected outliers were given in **Figure 6**. Considering all detected outliers, the B genome harbors nearly twice as much outlier loci compared to the A and D genome. In contrast, the D genome harbors only slightly more outlier loci than the A genome (**Figure 6A**). Looking at individual chromosomes, severe differences between the chromosomes can be detected. On the one hand side the chromosomes 1B, 2B, and 4B harbor approximately 25% of the outlier loci, while, on the other hand, ten chromosomes from the A and D genomes harbor less than 25% of the outlier loci (**Figure 6B**).

Restricting the outlier loci only to those that have been detected in at least 10% winter wheat genotypes (29 genotypes), the picture slightly changes. Again, the B genome harbors more than twice as much outlier loci than the A genome. The D genome harbors the least outlier loci with approximately 63% of the outlier loci compared to the A genome (**Figure 6C**). Also for individual chromosomes, a shift was observed. Chromosome 2B harbors the most outlier loci and chromosomes 6B, 5B and 7B are the runner-up. In contrast, chromosome 4B only harbors the seventh least outlier loci compared to the second most when considering all outlier loci (**Figure 6D**). For chromosome 4D, only two outliers could be detected that occur in at least 10% of genotypes, although more than 88% of the windows on chromosome 4D have been marked as outliers in at least one wheat genotype.

Looking at the spatial distribution in the complete genome, outliers are more frequent at the telomeres compared to centromers (**Figure 6E**). Some of the frequent outliers overlap with regions that contain genes of increased interest for breeding (Liu et al., 2012), as for instance, *Pm3* on 1AS, *Yr17* on 2AS, *Ppo-A1* on 2AL, *Wx-B1* on 4AL, *Lr19* on 7AL, *Psy-A1* on 7AL, *Glu-B3* on 1BS, *Psy-B1* on 7BL, *Glu-D1* on 1DL, and *Ppo-D1* on 2DL (**Supplemental Table 3**). For some of these genes, allelic variation was introduced by introgressions from crop wild relatives, e.g., *Yr17* from *Aegilops ventricosa* Tausch and *Lr19* from *Thinopyrum ponticum* (Liu et al., 2012). However, for several introgressions used in breeding programs, no molecular markers are publicly available. In addition, Thind et al. (2018) hypothesize that an interstitial introgression from *Ae. tauschii* is present in the wheat cultivar CH CampalaLr22a overlapping the large region with suspicious coverage on chromosome 2DL.

Looking at the size distribution of outlier stretches, about 66% of the winter wheat genotypes had consecutive outlier stretches with at least 50 outliers corresponding to at least 25 Mb (**Supplemental Table 4**). However, several other short stretches of outliers were found on all chromosomes.

**Figure 7:**
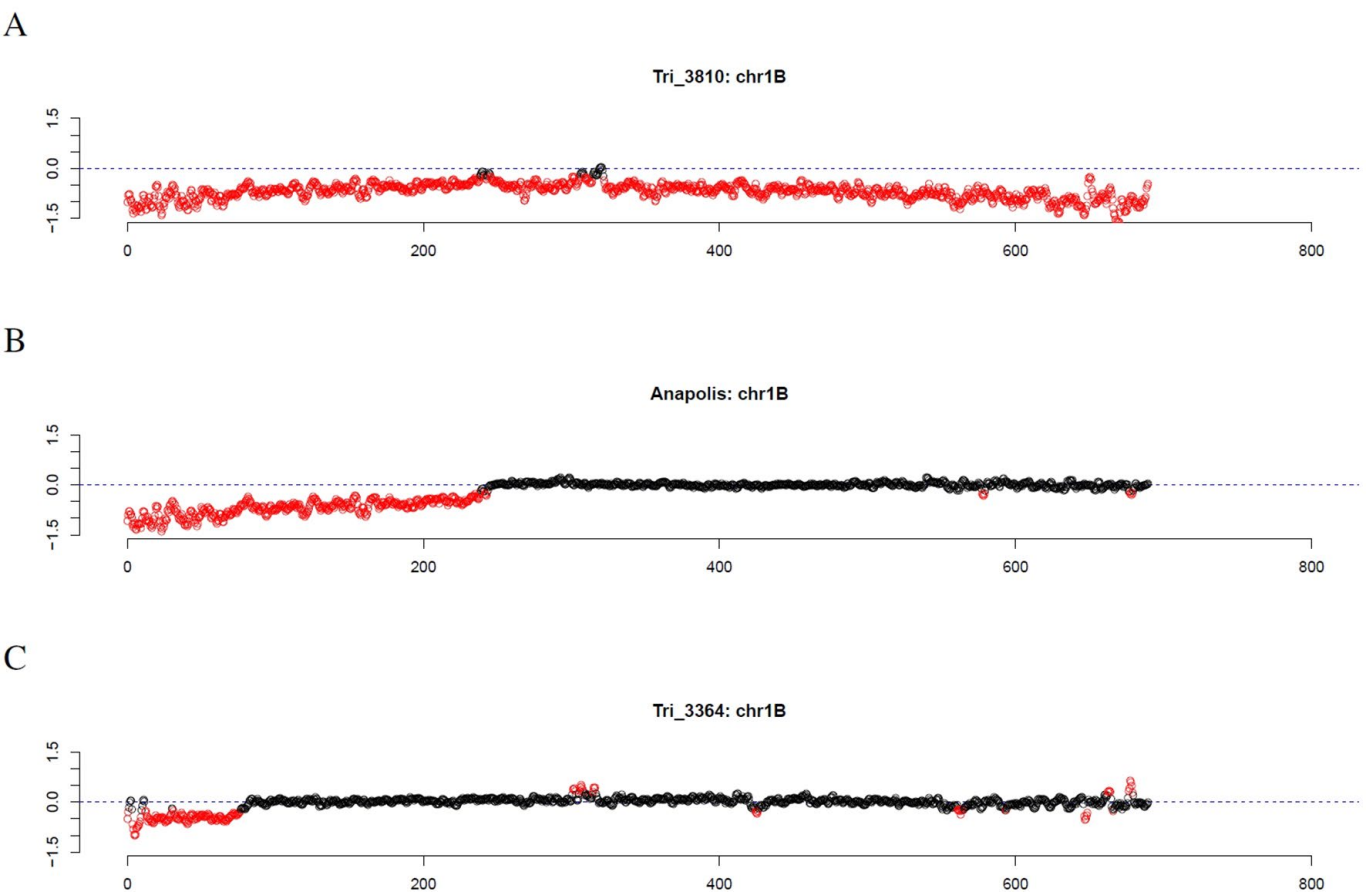
Outliers of GBS coverage data for wheat chromosome 1B for the wheat genotypes TRI 3810, Anapolis, and TRI 3364. The x-axis depicts the position within the chromosome in Mb, while the y-axis depicts normalized coverage. Each dot visualizes the denoised coverage value of a non-overlapping 500kb window, while the dashed line depicts the expectation. Dots are depicted in red if they are marked as outliers indicating large chromosomal modifications.

Investigating individual winter wheat genotypes, we observed several striking patterns. For the Genebank accessions TRI 3810 (Salzmünder 14/44) and TRI 9323 (Mildress), almost all windows on the complete chromosome 1B were marked as outliers (**Figure 7A**). Further winter wheat genotypes comprising Anapolis, Brilliant, Matrix, Pamier, Winnetou, TRI 9367, TRI 10373, and TRI 11247 only had outliers on the short arm of chromosome 1B (**Figure 7B**). Other wheat genotypes, as for instance TRI 3364, had even smaller stretches of outliers on chromosome arm 1BS (**Figure 7C**).

Similar to chromosome 1B, other chromosomes also exhibited large patterns of decreased coverage including chromosome 1A for Memory and TRI 6874; chromosome 1D for Kometus; chromosome 3B for TRI 994; chromosome 3D for TRI 10166; chromosome 4B for Smaragd; chromosome 4D for TRI 7040; chromosome 5B for TRI 12027; chromosome 6B for TRI 5164 and TRI 6775; and chromosome 7A for Brilliant and TRI 1005 (**Supplemental Data Sheet 2**). In contrast, large patterns of increased coverage were observed, for instance, on chromosome 1D for TRI 6868, and on chromosome 2D for TRI 5042 and TRI 7716 (**Supplemental Data Sheet 3**). The large pattern detected in the wheat line TRI 7040 explains the high percentage of windows on chromosome 4D that are marked as outlier in at least one wheat line.

Based on literature, some of these patterns can be explained. For instance, Rabinovich (1998) reported that TRI 3810 (Salzmünder 14/44) and TRI 9323 (Mildress) have a substitution of wheat chromosome 1B by the rye chromosome 1R. For several wheat lines, including TRI 9367 (Skorospelka 35) (Gupta and Shepherd, 1992) and TRI 10373 (Benno) (Luo et al., 2008), the rye-wheat translocation 1RS.1BL was reported. Using rye-specific primers, rye DNA was detected in a variable number of genotypes. Rye DNA was indicated for 26, 10, 10, and 10 genotypes using the markers OP20H, SCM9, ora003, and ora007, respectively (**Supplemental Table 4**, **Supplemental Figure 1**). The combination of markers indicated an introgression of the short arm of rye chromosome 1R for ten genotypes comprising Anapolis, Brilliant, Memory, Pamier, Winnetou, TRI 3810, TRI 9323, TRI 9367, TRI 10373, and TRI 11247. These data in combination with the coverage profiles allows to identify the locus of the introgression. A substitution of wheat chromosome 1B by the rye chromosome 1R was indicated for TRI 3810 and TRI 9323 and a 1RS.1AL translocation was indicated for Memory, while for the other seven genotypes a 1RS.1BL translocation was indicated.

Read mapping against a combined wheat and rye reference was performed using the wheat genotypes carrying 1RS. Subsequently, SNP calling was performed on chromosome 1R obtaining 177 bi-allelic SNPs that have been called in all of these genotypes (**Supplemental Table 5**). SNPs were classified heterozygous ranging from about 15% for Memory to about 30% for all other genotypes (∼26% for Anapolis to ∼32% for TRI 9323 and TRI 11247). A position in the high-density genetic map of rye (Bauer et al., 2017) could be determined for 133 SNPs (75.2%), while the map position for 44 SNPs (24.8%) remains unknown. In total, 125 SNPs map to the short arm of chromosome 1R (**Supplemental Table 4**). Focusing only on SNPs that have been called homozygous for all ten genotypes yielded only 95 SNPs (75 SNPs below 70cm, 18 SNPs unknown, and only 2 SNPs above 70cm). Based on these 95 SNPs only two haplotypes were identified: one for Memory and the other for the remaining nine genotypes.

The genotypes contained in our dataset span almost eight decades of German wheat breeding ranging from 1940 to the present day. This provides the opportunity to assess variation of the molecular targets in German wheat breeding over the decades. Indeed, ontology term enrichment analyses of the gene sets varying in coverage across the different decades reveal specific changes in the biological processes and anatomical structures of these wheat lines (**Supplemental Figures 2** and **3**; **Supplemental Table 6**). Overall, as suggested by the substantially higher number of significantly enriched terms in modern elite lines, current breeding efforts seem to target a broad spectrum of biological processes and structures. This ranges from improvement of pathogen/herbivore resistance, climate change effects including flooding and hypoxia, modulation of sulphur compounds for storage protein quality, increased nutrient and water uptake by improvement of the root system and overall vasculature, robustness of the stele in all plant body parts to avoid lodging to overall fine tuning of developmental and reproductive processes to increase yields in specific climate conditions. The few cases with an enrichment of GO and PO terms from older decades provide striking examples for the accuracy and the potential of our method. We observe an enrichment of genes related to trichomes in genotypes from the 1940s and 1970s. This illustrates the hidden potential in these older genotypes, because trichome length and density are an important resistance factor for the cereal leaf beetle whose larvae and adults feed on leaves and also increase the probability of fungal infections (Hoxie et al., 1975; Konyspaevna, 2012). Plants originating from the 1970s surprisingly show an enrichment of oligopeptide transport. The literature record provides a compelling explanation for this observation. Oligopeptide transporters like yellow-stripe-like transporters (YSL) are important for micronutrient uptake including Fe and Zn (Kumar et al., 2019). Strikingly, previous studies reported substantially higher mineral micronutrient grain contents especially for Zn and Fe in wheat genotypes from that time period also including several German lines (Zhao et al., 2009).

## Discussion

In order to better understand the genomic composition of barley and wheat genotypes, we aim at the detection of large chromosomal modifications using GBS data and a bioinformatics pipeline. Instead of using SNPs, we investigate the sequence coverage. Differences in the coverage of GBS data might be attributed to (a) missing or duplicated genomic regions, (b) mutations in the recognition site of the restriction enzyme, or (c) changes in the methylation of the recognition site of the methylation-sensitive restriction enzyme.

Increased coverage normally indicates more reads than expected and, hence, an addition or duplication, while decreased coverage normally indicates less reads than expected and, hence, a deletion or substitution. If no reference plant is available, at each locus the median at this locus within the collection can be used. However, this renders the interpretations of coverage profiles a little bit more difficult.

We perform three case studies analyzing barley and wheat collections and find several interesting patterns of increased and decreased sequence coverage. These patterns indicate large modifications of chromosomal regions that might be larger deletions, duplications, and substitutions, as well as modification of DNA methylation.

Firstly, we demonstrate that GBS sequence coverage profiles can be used as an alternative method to detect large chromosomal modifications in barley (*H. vulgare/H. bulbosum*) introgression lines using the GBS data of the known parent as reference. Subsequently, we show that GBS data of the parental lines are dispensable for the identification of the introgressed regions. This observation is of central importance as it allows to determine the size and chromosomal location of introgressed regions if pedigree data or GBS data of ancestors is either not available or not accessible. In both cases, the results agreed very well with those reported by Wendler et al. (2015).

Secondly, we analyze the barley genebank collection from IPK Gatersleben looking for large chromosomal modifications. We also identify outliers in this collection. The amount of large outlier stretches is low, which might be a result of very low efficiency of natural inter-species hybridization for diploid plants. Nevertheless, we identify barley accessions with striking patterns of decreased or increased GBS sequence coverage. Three of these accessions show a clear pattern of decreased GBS sequence coverage which might be attributed to an introgression or a deletion. Since these accessions have been introduced in the genebank in 1942, 1976, and 1983, this might indicate natural inter-species hybridization or long deletions in barley. Three other accessions show a clear pattern of increased GBS sequence coverage which might be attributed to duplications. However, we also identify several smaller outlier stretches that are predominantly located in telomeric regions. Based on outliers that occur in at least 3% of the accessions, we identified several significantly enriched GO terms of genes located in these regions. The distribution of the observed patterns and the enriched GO terms match the findings for CNV in barley (Muñoz-Amatriaín et al., 2013).

Thirdly, we investigate GBS coverage profiles of wheat cultivars and genebank accessions. We find outliers for all wheat genotypes and large stretches of outliers for about 66% of the wheat genotypes. Some of these outliers can be associated with rye introgressions reported in literature or verified by wet-lab experiments. Based on SNP data, we could identify only two haplotypes of 1RS introgressions in 10 wheat genotypes carrying 1RS. Mainly four rye sources have been used to incorporate rye chromatin in wheat, deployed as (1B)1R substitution or 1BL.1RS and 1AL.1RS translocation lines (Crespo-Herrera et al., 2017). The most widely exploited source carries the 1RS.1BL translocation from Petkus rye, while the 1RS.1AL translocation has an independent origin (Schlegel and Korzun, 1997). The SNPs identified in the present study represent the currently most comprehensive description of both translocations at the molecular level and extends the molecular toolbox available for genetic analyses of this important alien introgression in wheat. Furthermore, the 1RS introgression serves as a positive control for our GBS-based approach to detect alien introgressions in wheat. However, as potential donors are unknown, it is hard to verify all detected patterns. Interestingly, we find only two outliers on chromosome 4D that occur in at least 10% wheat genotypes, which is consistent with reports of low diversity in wheat subgenome D and especially on chromosome 4D (Akhunov et al., 2010).

Summarizing these observations, we can state that GBS sequence coverage profiles are a new method to determine large chromosomal modifications in the major cereals barley and wheat. These modifications can be introgressions, but also other processes could be explanations for these patterns as for instance CNV. Besides SNPs, introgressions and CNVs are very important for breeding and can now be analyzed without additional wet-lab experiments if SNPs were detected using GBS. In principle, other sequencing protocols like exome capture (Mascher et al., 2013) or adaption of single-primer enrichment technology (Scaglione et al., 2019) might possibly be used as an alternative to GBS for coverage analysis. In this study, we analyze barley and wheat, but the method might be applicable for other species as well if a reference genome is available.

In summary, the GBS coverage analysis can be used to infer introgressed regions and to identify genotypes with suspicious coverage patterns in wheat and barley. Coverage analysis has several advantages compared to other detection methods. Firstly, coverage analysis is a simple way for detecting chromosomal modifications. Secondly, it allows to detect a variety of chromosomal modifications including introgressions and CNVs on a genome-wide scale. Thirdly, in case of introgressions no information about the donor is needed to provide probes for hybridization. Hence, the method allows to detect a wide range of introgressions from different crop wild relatives using a single wet-lab experiment. Fourthly, the resolution that was used in this study was 500kb which is much better than for many other detection methods. Fifthly, since sequencing data are generated, these data can also be used to identify SNPs and derive markers, e.g. for marker-assisted selection. Hence, GBS can provide information about SNPs, introgressions and CNVs with a single, simple and cheap wet-lab experiment. Although, analysis of missing values is also possible for SNP arrays (data not shown), much more information can be obtained from coverage analysis rendering GBS a more valuable resource for genotyping compared to SNP arrays. For this reason, coverage analysis on GBS data might be an important additional argument in the discussion about which marker system to be used (Darrier et al., 2019). Sixthly, based on SNP data the method allows to distinguish different donors from the same species (cf. wheat genotypes carrying 1RS). Finally, decreased GBS coverage could possibly lead to a shortage of detected polymorphisms in introgressed regions which hampers the detection of such regions based on SNPs compared to coverage analysis (e.g., ERR699920, **Figure 1A**).

However, the method might be limited in detecting very small modifications due to the limited number of GBS data and the applied window approach. Applying the idea to the genomic position of individual reads might increase the resolution (Lemay et al., 2019), but on the other hand could hinder the detection of larger chromosomal modifications. In addition, the phylogenetic distance between the wild donor and the crop plant might probably influence the detection rate of introgressed regions, where DNA from a closely related donor might be harder to detect. Furthermore, the method will not be able to detect modifications in regions that are not represented in the reference genome sequence as well as inversions or reciprocal translocations, as for instance the reciprocal translocation 5B:7B in the wheat cultivar Cappelle-Desprez (Badaeva et al., 2007).

Besides detecting regions of suspicious coverage, the method might be used for several downstream analyses. The proposed method is just a fast screening approach for regions with unexpected coverage, while association methods like genome-wide association studies (GWAS) try to identify genetic markers that explain the phenotypic data that was often collected in time-consuming experiments. Hence, combining GBS coverage data with phenotypic observations could potentially associate regions of unexpected coverage with plant traits. In addition, the method might be used to identify genomic regions under selection. Furthermore, the method could be applied to verify or falsify duplicates in genebank collections. Additionally, a shortage of detected polymorphisms in introgressed regions might lead to an underestimation of the genetic distance between different genotypes. Hence, there might be a need for the development of alternative genetic distance measures.

## Author Contributions

JK developed the idea. AH performed GBS of the winter wheat collection. SB performed read mapping of the winter wheat collection. TB performed read mapping of the barley collections and the combined read mapping against wheat and rye for some wheat genotypes. JK implemented the software and performed the computational analysis. DL performed ontology term enrichment. JK, HL, NS, EB, DL, BK, BH, and DP discussed the results. BH tested wheat genotypes with rye-specific markers. JK wrote the manuscript. All authors discussed and approved the final manuscript.

## Funding

HL and GBS data of the winter wheat collection were supported by the Federal Ministry of Food and Agriculture within the GenDiv project (grant no. 2814603813).

## Conflict of Interest Statement

The authors declare that the research was conducted in the absence of any commercial or financial relationships that could be construed as a potential conflict of interest.

## Footnotes

1 The median was chosen as it has the best possible breakdown point of 50%.

## Supplementary Material

Supplemental Table 1 Detected outliers of *H. bulbosum* introgression lines.

Supplemental Table 2 Significantly enriched GO terms of loci detected in at least 3% of barley genotypes.

Supplemental Table 3 Position of functional markers in wheat determined by blast.

Supplemental Table 4 Detected outliers of wheat genotypes.

Supplemental Table 5 SNPs and representative reads on 1R for 10 wheat genotypes carrying 1RS.

Supplemental Table 6 Significantly enriched GO terms detected in wheat genotypes per decade

Supplemental Data Sheet 1 Coverage profiles of *H. bulbosum* introgression lines.

Supplemental Data Sheet 2 Coverage profiles of large chromosomal modifications with decreased coverage form selected wheat genotypes.

Supplemental Data Sheet 3 Coverage profiles of large chromosomal modifications with increased coverage form selected wheat genotypes.

Supplemental Figure 1 Gel image for 30 winter wheat genotypes using 4 rye markers.

Supplemental Figure 2 Significantly enriched (FDR<0.01 in at least one decade set) GO biological process terms for all studied decades. Subplots distinguish whether the FDR values were < 0.1 (FALSE|TRUE).

Supplemental Figure 3 Significantly enriched (FDR<0.01 in at least one decade set) GO biological process terms for all studied decades. Subplots distinguish whether the FDR values were < 0.1 (FALSE|TRUE).
