# Supplementary material for: Detecting Large Chromosomal Modifications Using Short Read Data From Genotyping-by-Sequencing
Source: Front Plant Sci. 2019 Sep 24;10:1133. doi: 10.3389/fpls.2019.01133 (PMC6771380; doi:10.3389/fpls.2019.01133)
Supplement: Supplemental Data Sheet 1 — Coverage profiles of H. bulbosum introgression lines. [file DataSheet_1.pdf]

ERR699793

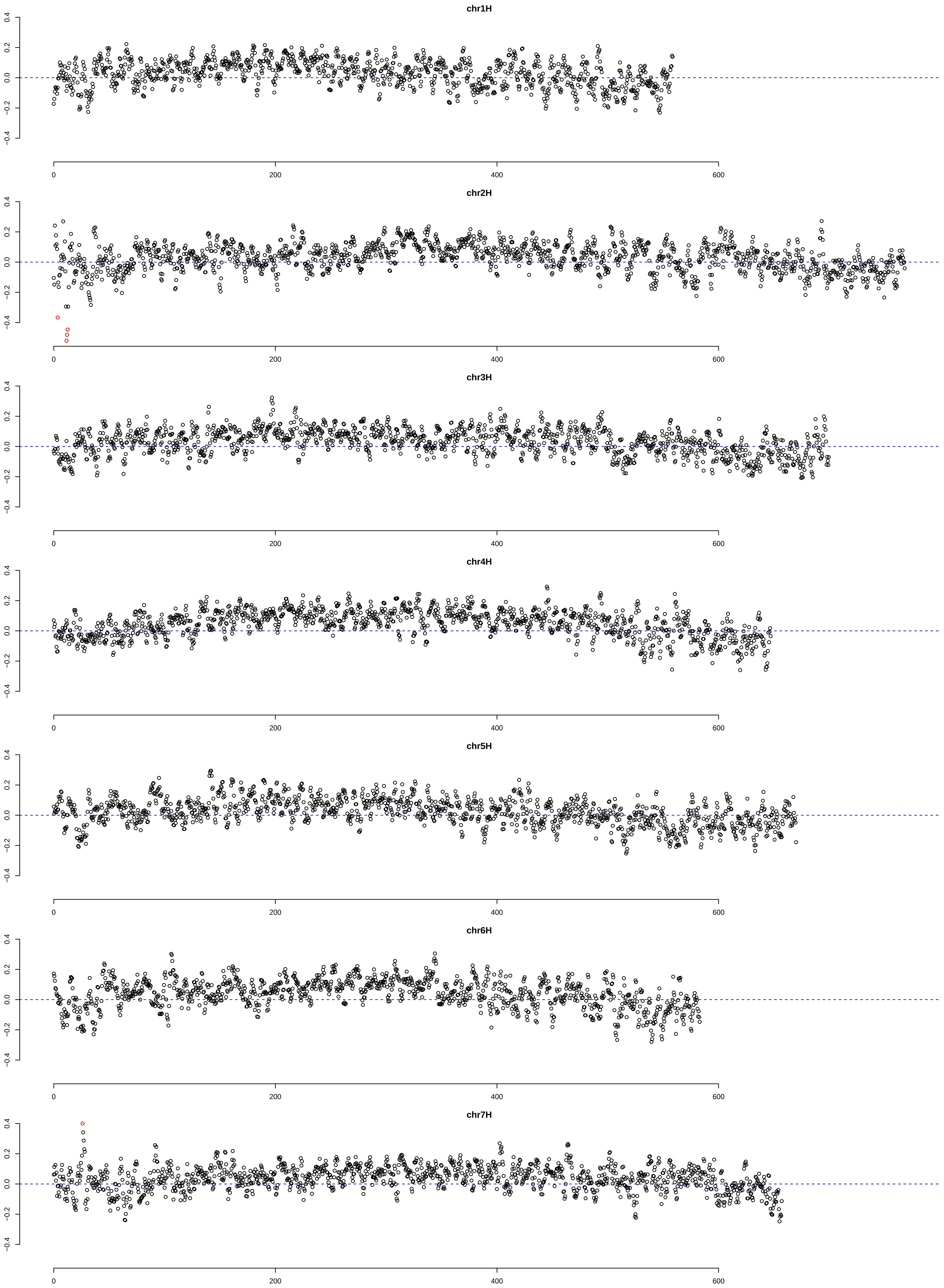

chr1H
0.4
0.2
0.0
−0.2
−0.4
0
200
400
600
chr2H
chr3H
chr4H
chr5H
chr6H
chr7H

ERR699794

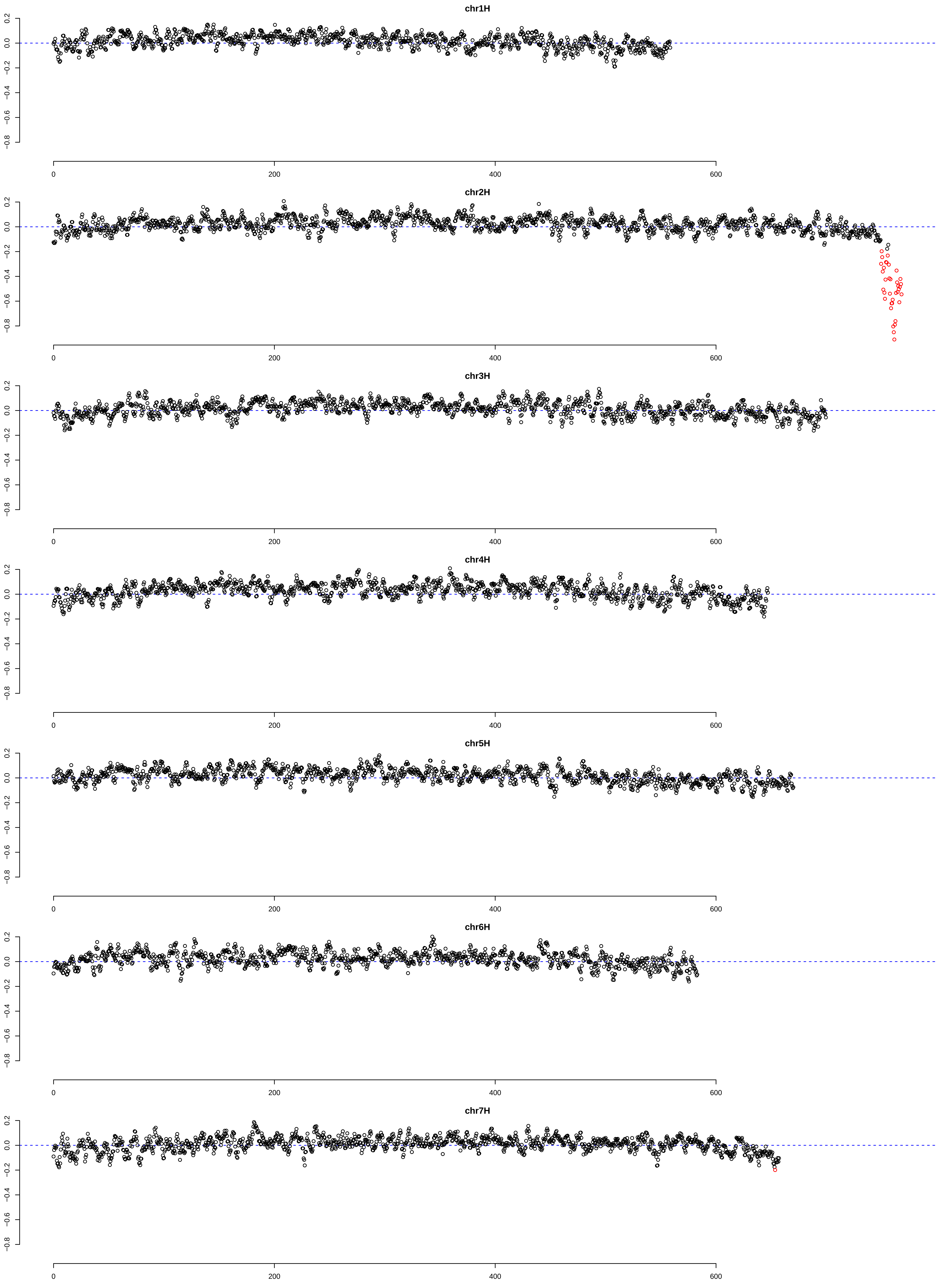

chr1H
chr2H
chr3H
chr4H
chr5H
chr6H
chr7H
0.2
0.0
−0.2
−0.4
−0.6
−0.8
0
200
400
600

ERR699795

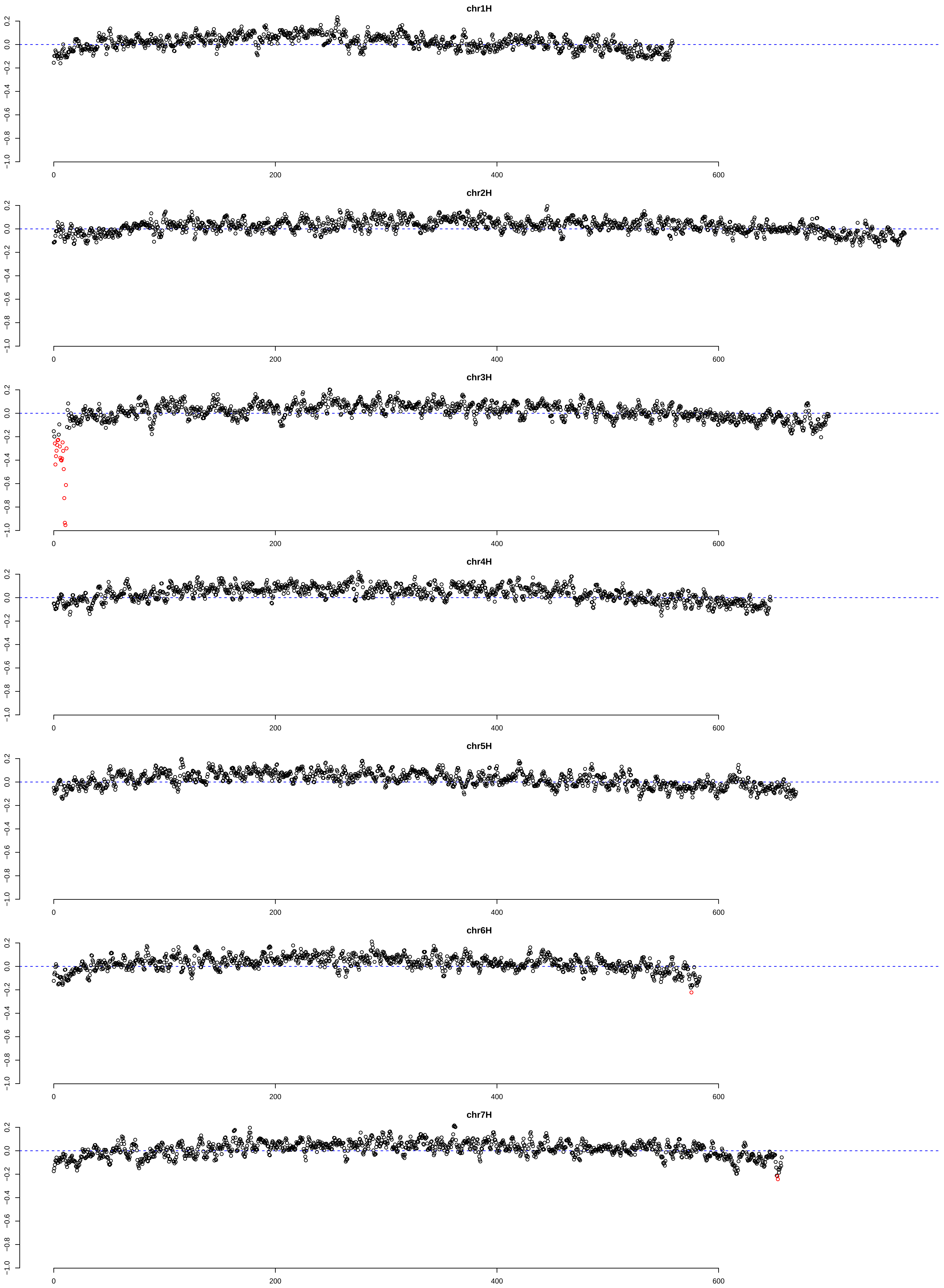

chr1H
chr2H
chr3H
chr4H
chr5H
chr6H
chr7H
0.2
0.0
-0.2
-0.4
-0.6
-0.8
-1.0
0
200
400
600

ERR699796

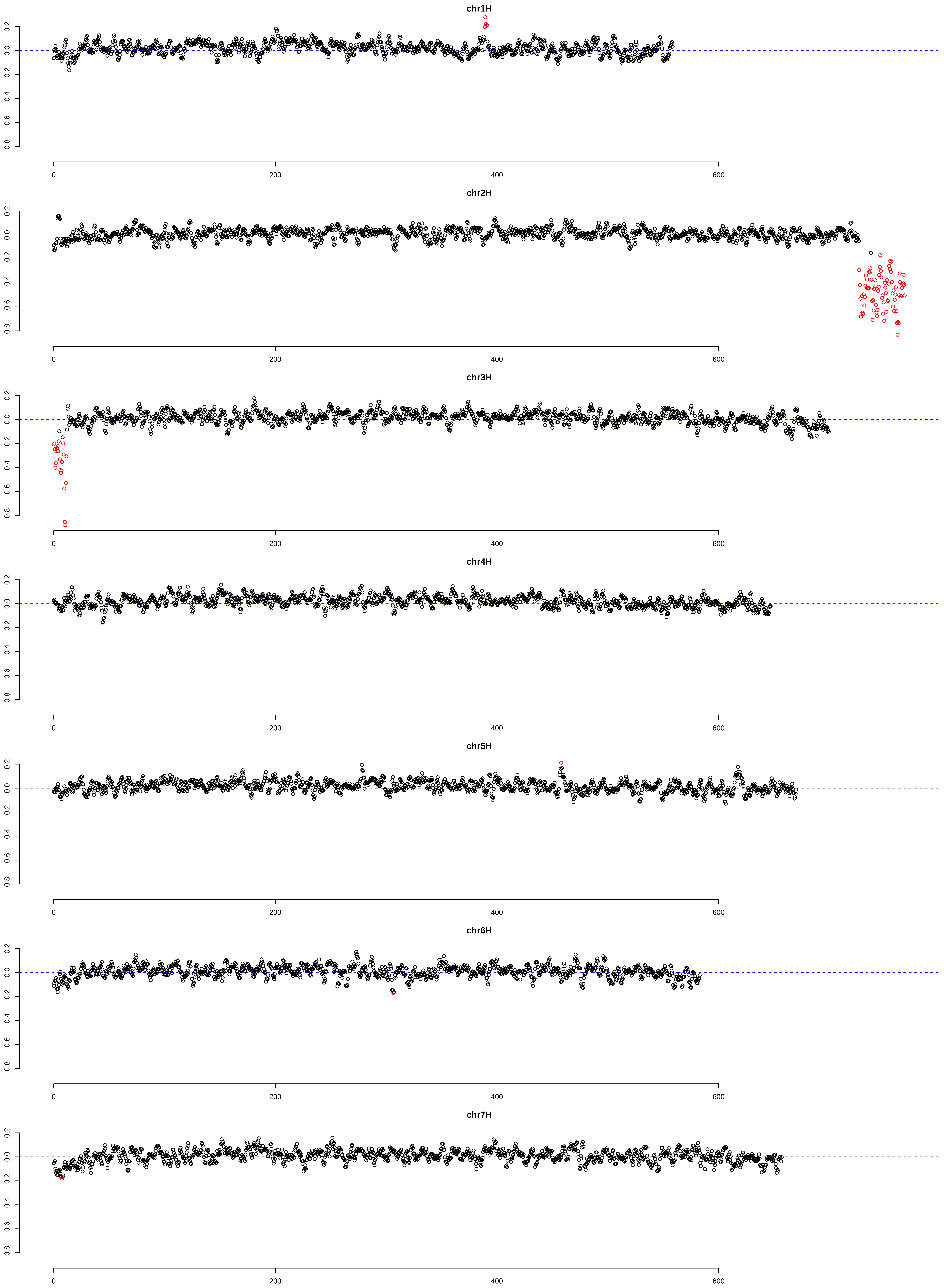

chr1H
chr2H
chr3H
chr4H
chr5H
chr6H
chr7H
0.2
0.0
-0.2
-0.4
-0.6
-0.8
0
200
400
600

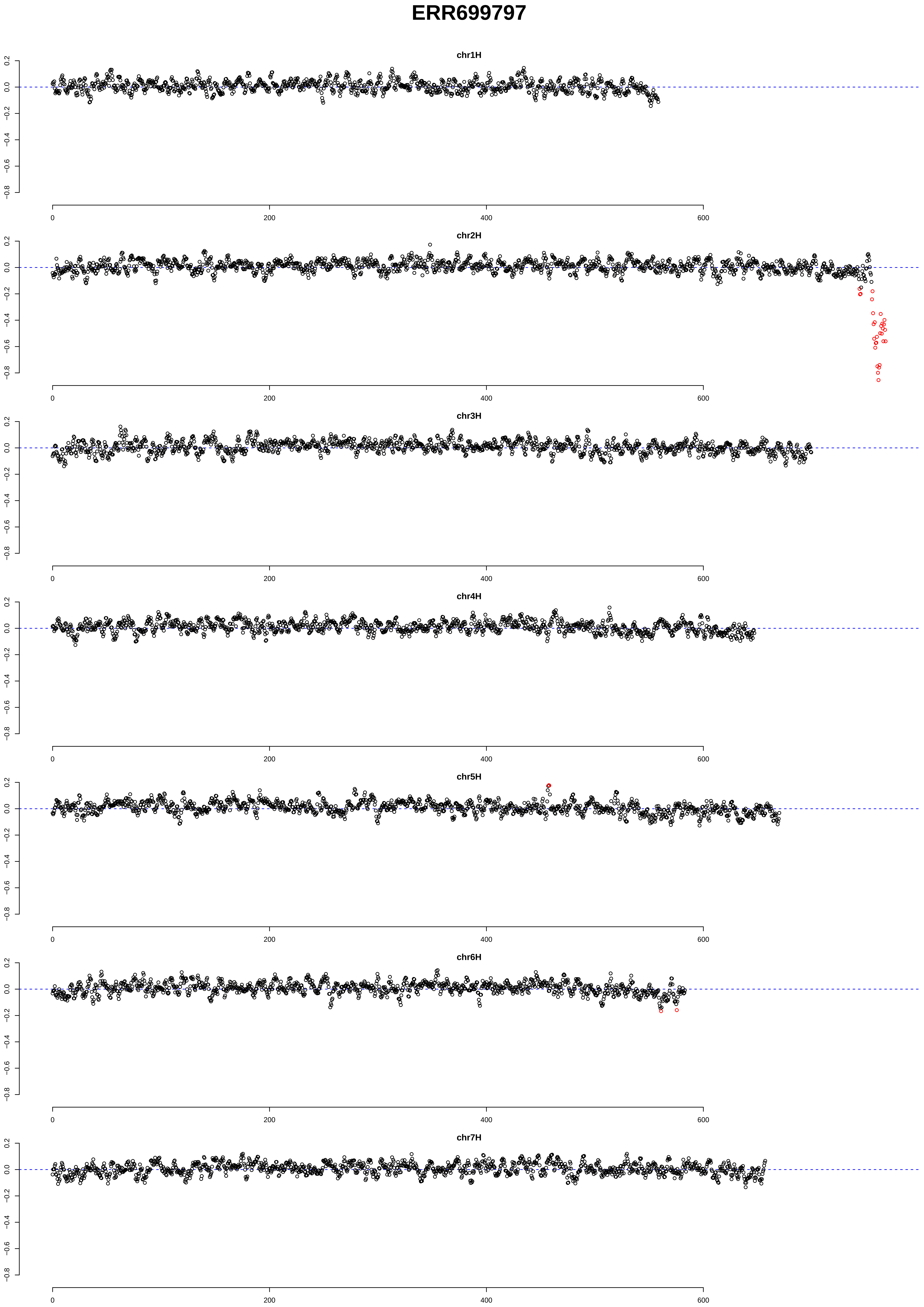
ERR699797
chr1H
chr2H
chr3H
chr4H
chr5H
chr6H
chr7H
0.2
0.0
-0.2
-0.4
-0.6
-0.8
0
200
400
600

ERR699798

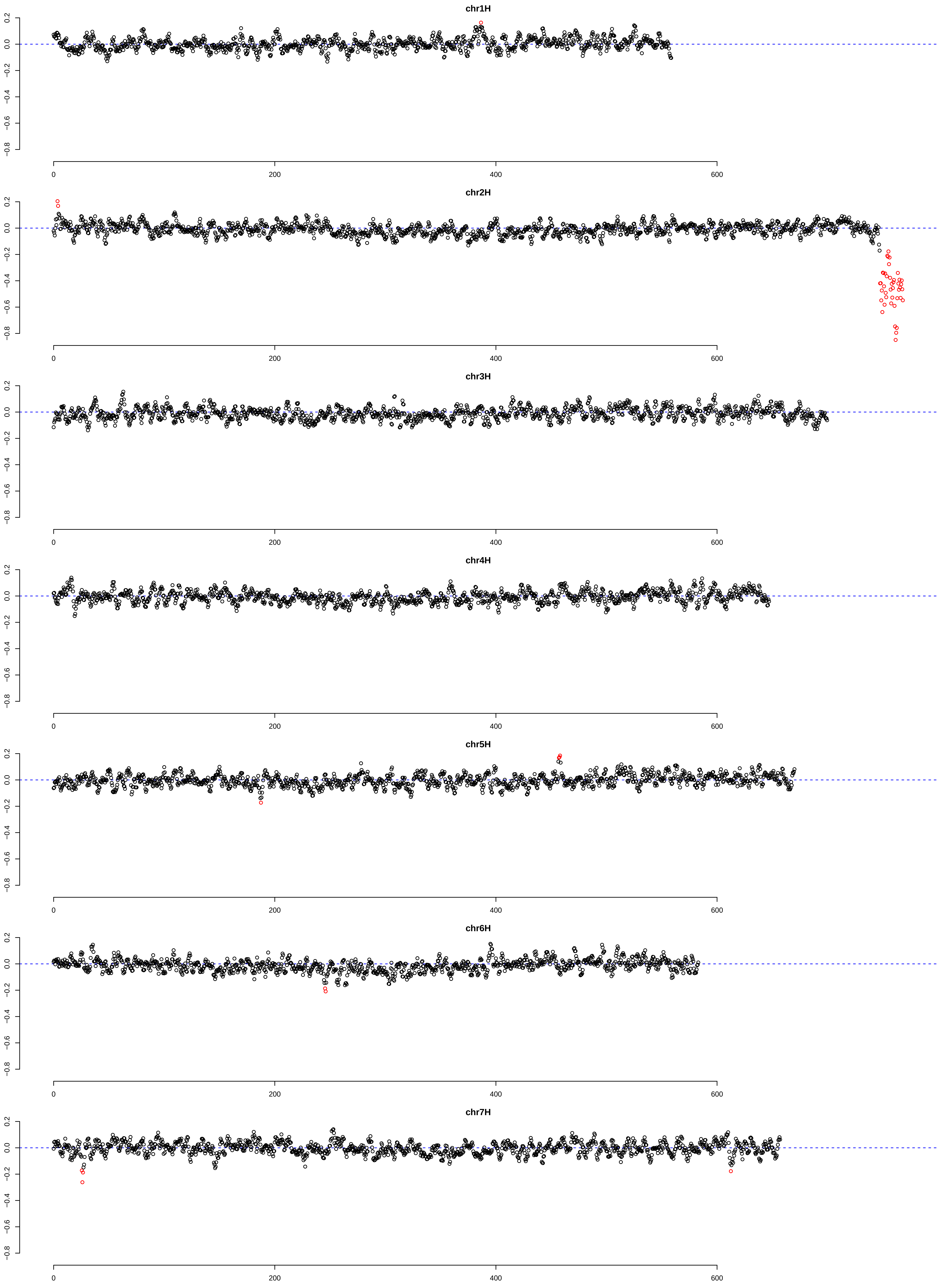

chr1H
chr2H
chr3H
chr4H
chr5H
chr6H
chr7H
0.2
0.0
-0.2
-0.4
-0.6
-0.8
0
200
400
600

ERR699799

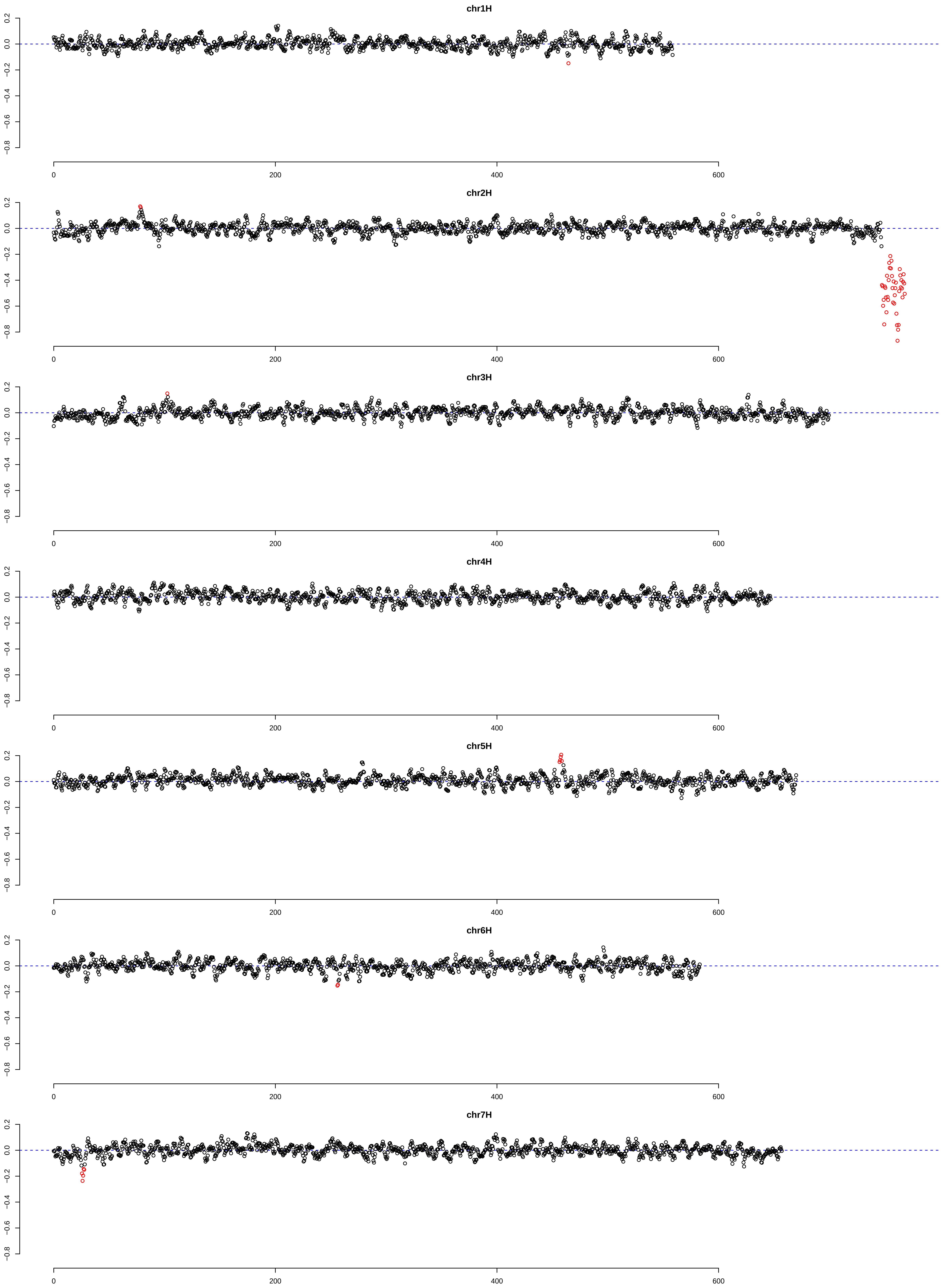
chr1H
chr2H
chr3H
chr4H
chr5H
chr6H
chr7H
0.2
0.0
-0.2
-0.4
-0.6
-0.8
0
200
400
600

ERR699800

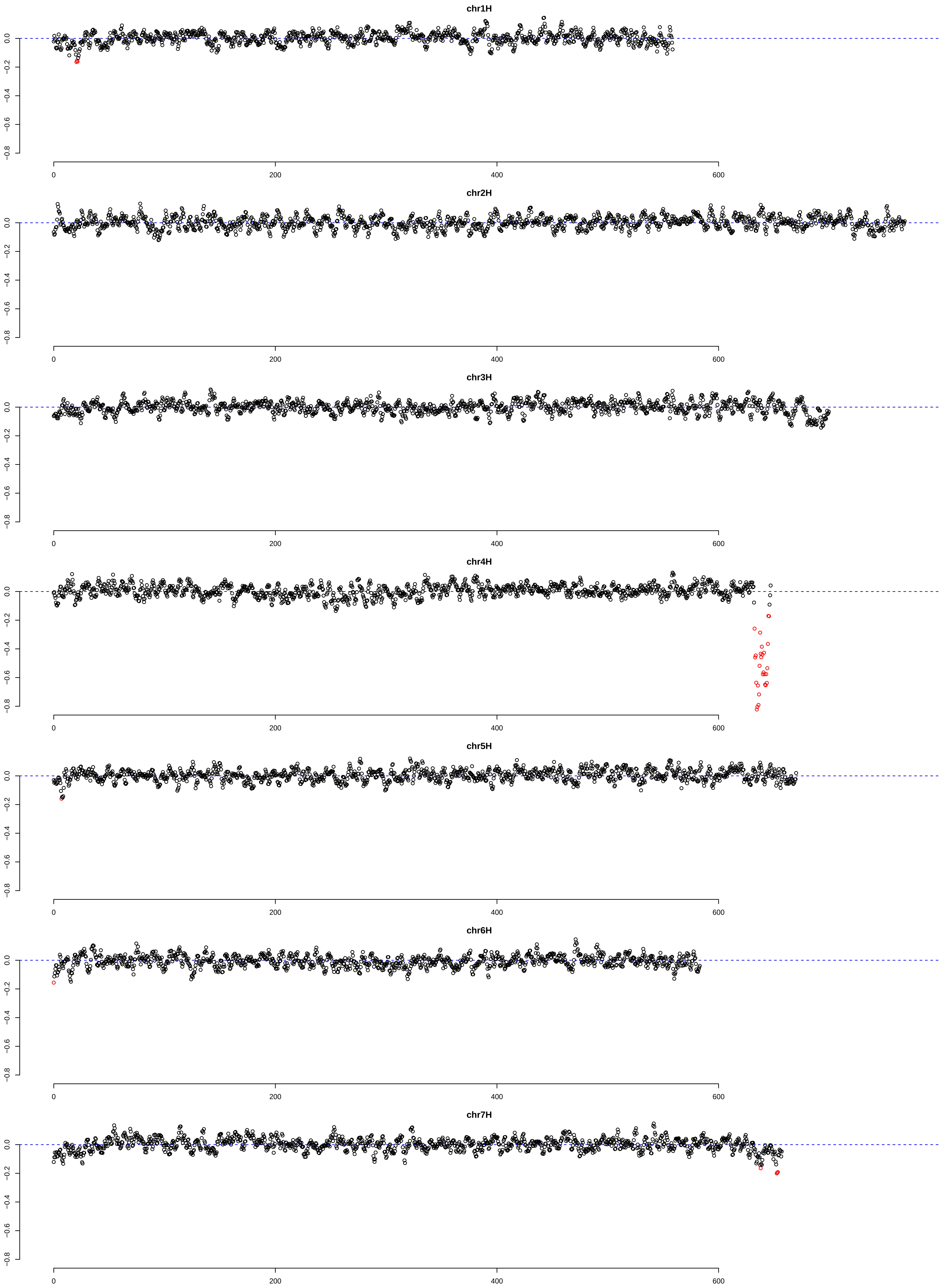
chr1H
chr2H
chr3H
chr4H
chr5H
chr6H
chr7H
0.0
-0.2
-0.4
-0.6
-0.8
0
200
400
600

ERR699801

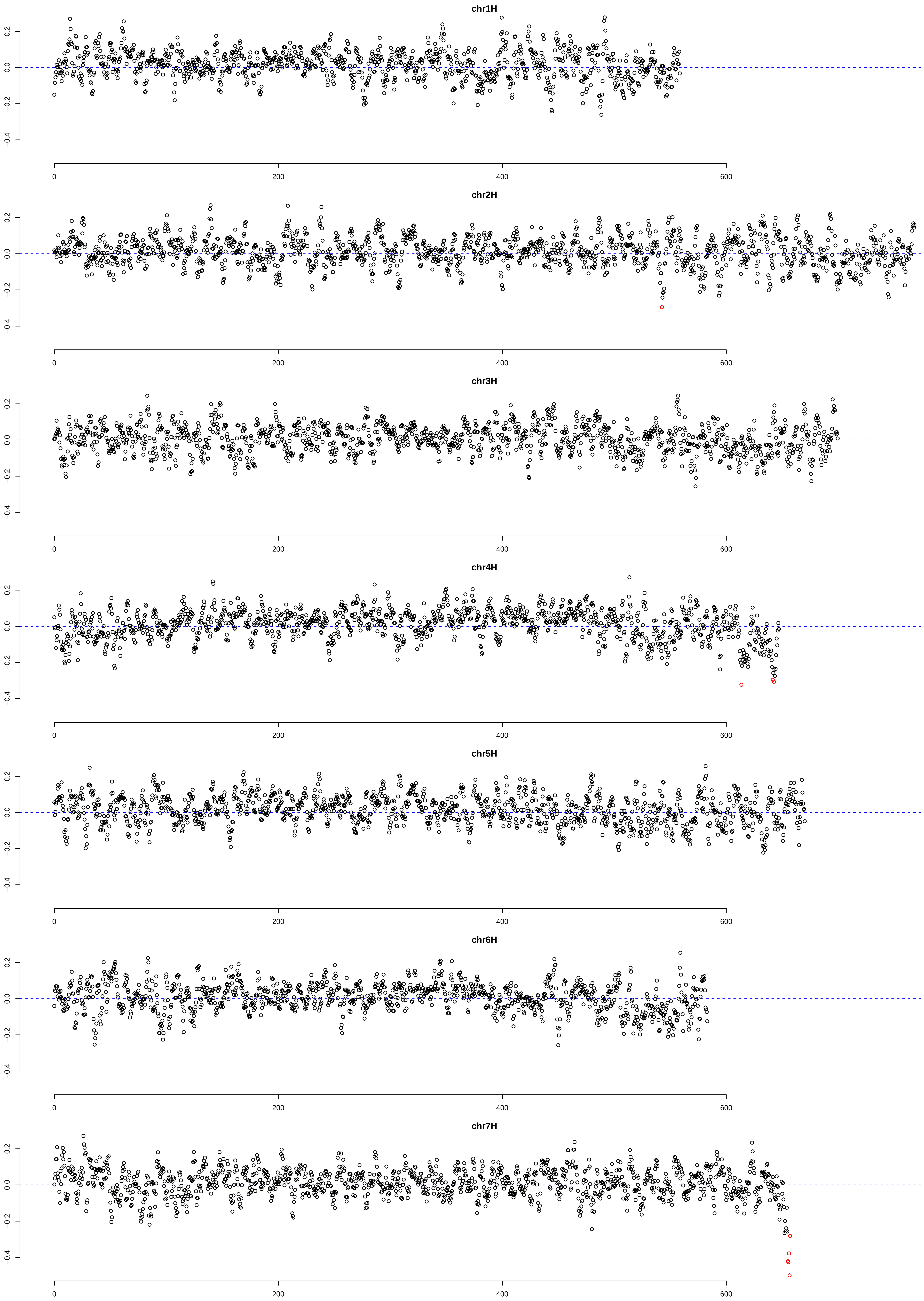

chr1H
chr2H
chr3H
chr4H
chr5H
chr6H
chr7H
0.2
0.0
-0.2
-0.4
0
200
400
600

ERR699802

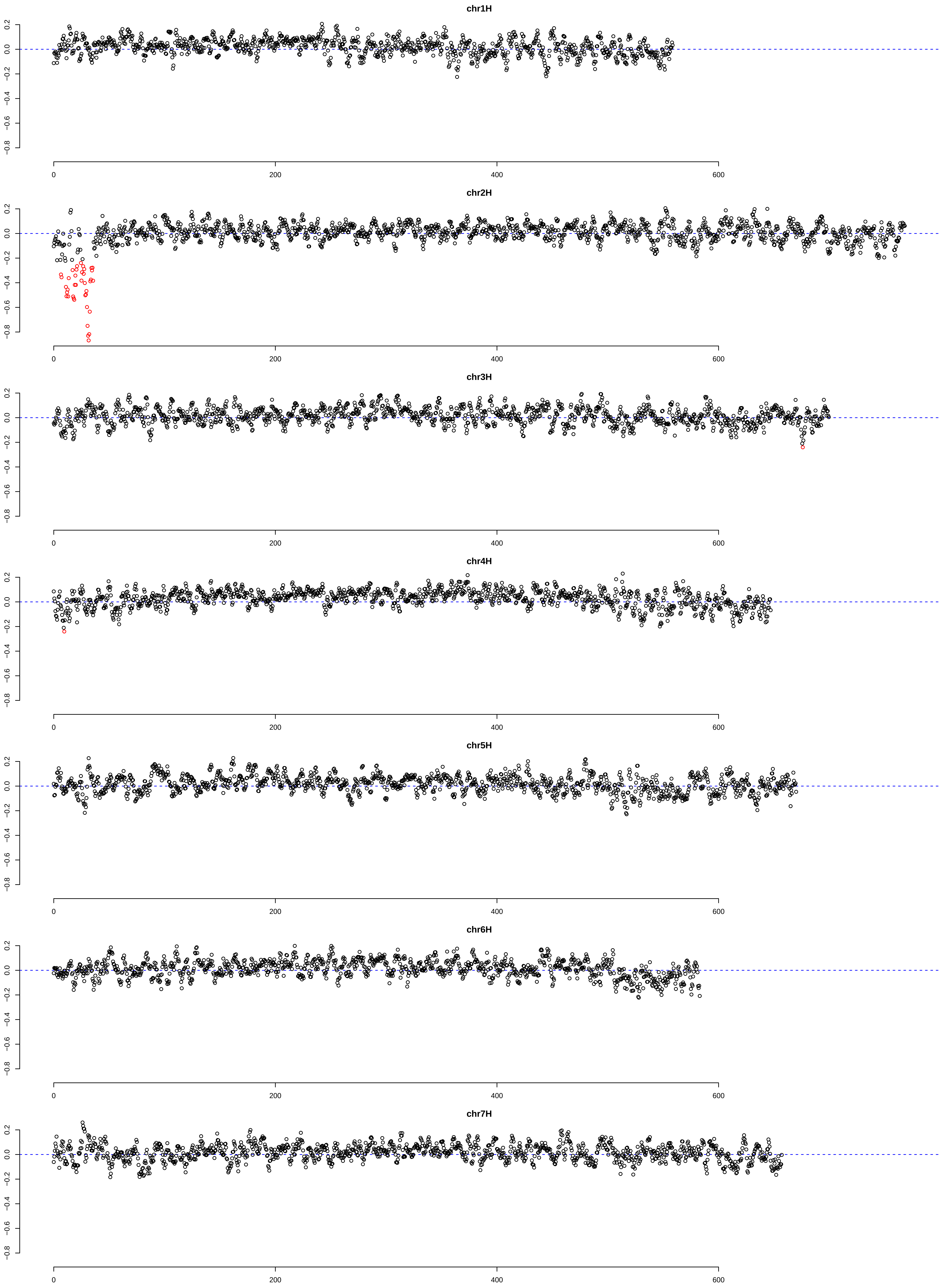

chr1H
chr2H
chr3H
chr4H
chr5H
chr6H
chr7H
0.2
0.0
-0.2
-0.4
-0.6
-0.8
0
200
400
600

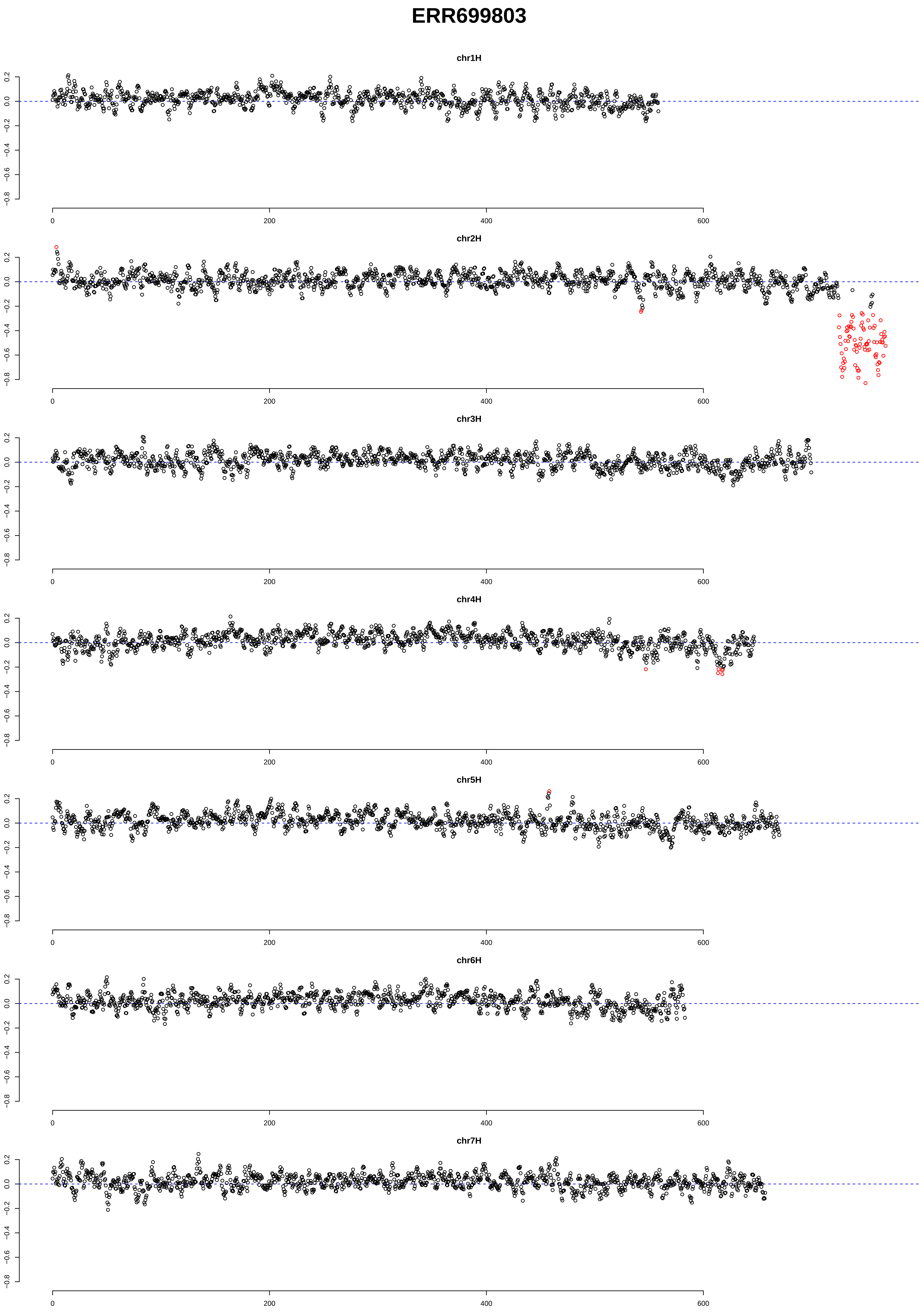
ERR699803
chr1H
chr2H
chr3H
chr4H
chr5H
chr6H
chr7H
0.2
0.0
-0.2
-0.4
-0.6
-0.8
0
200
400
600

ERR699804

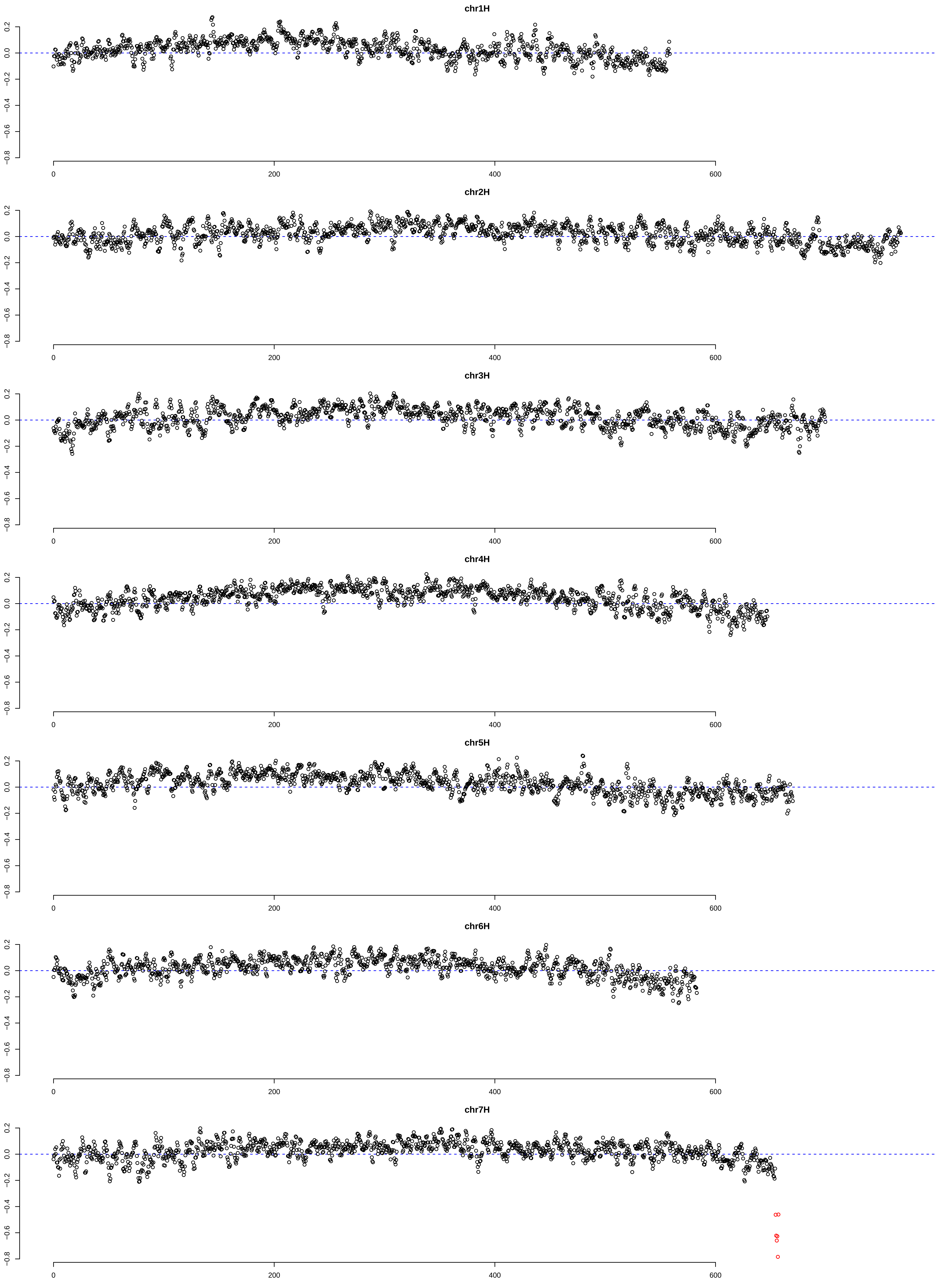
chr1H
chr2H
chr3H
chr4H
chr5H
chr6H
chr7H
0.2
0.0
-0.2
-0.4
-0.6
-0.8
0
200
400
600

ERR699805

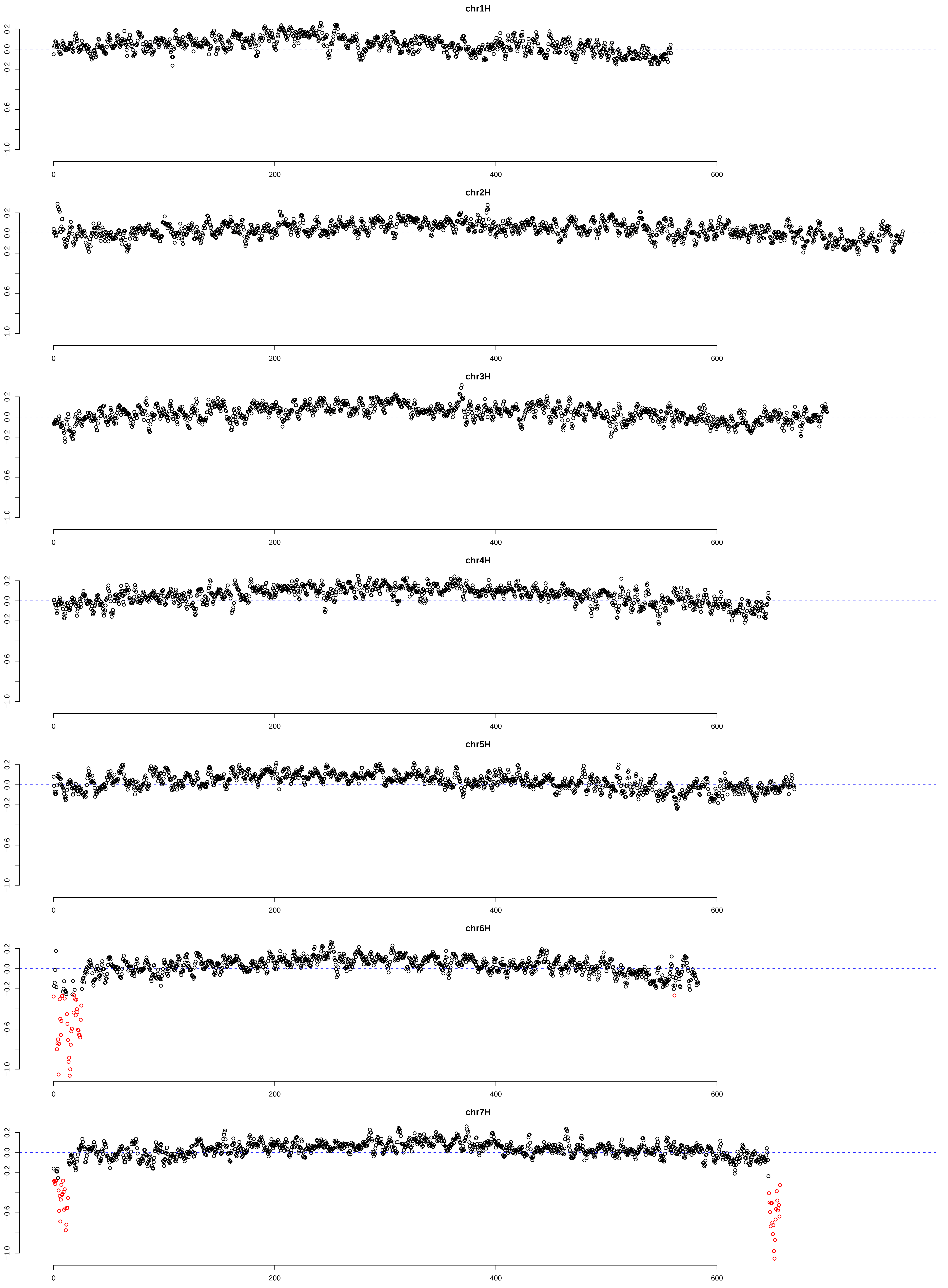

chr1H
chr2H
chr3H
chr4H
chr5H
chr6H
chr7H
0.2
0.0
-0.2
-0.6
-1.0
0
200
400
600

ERR699806

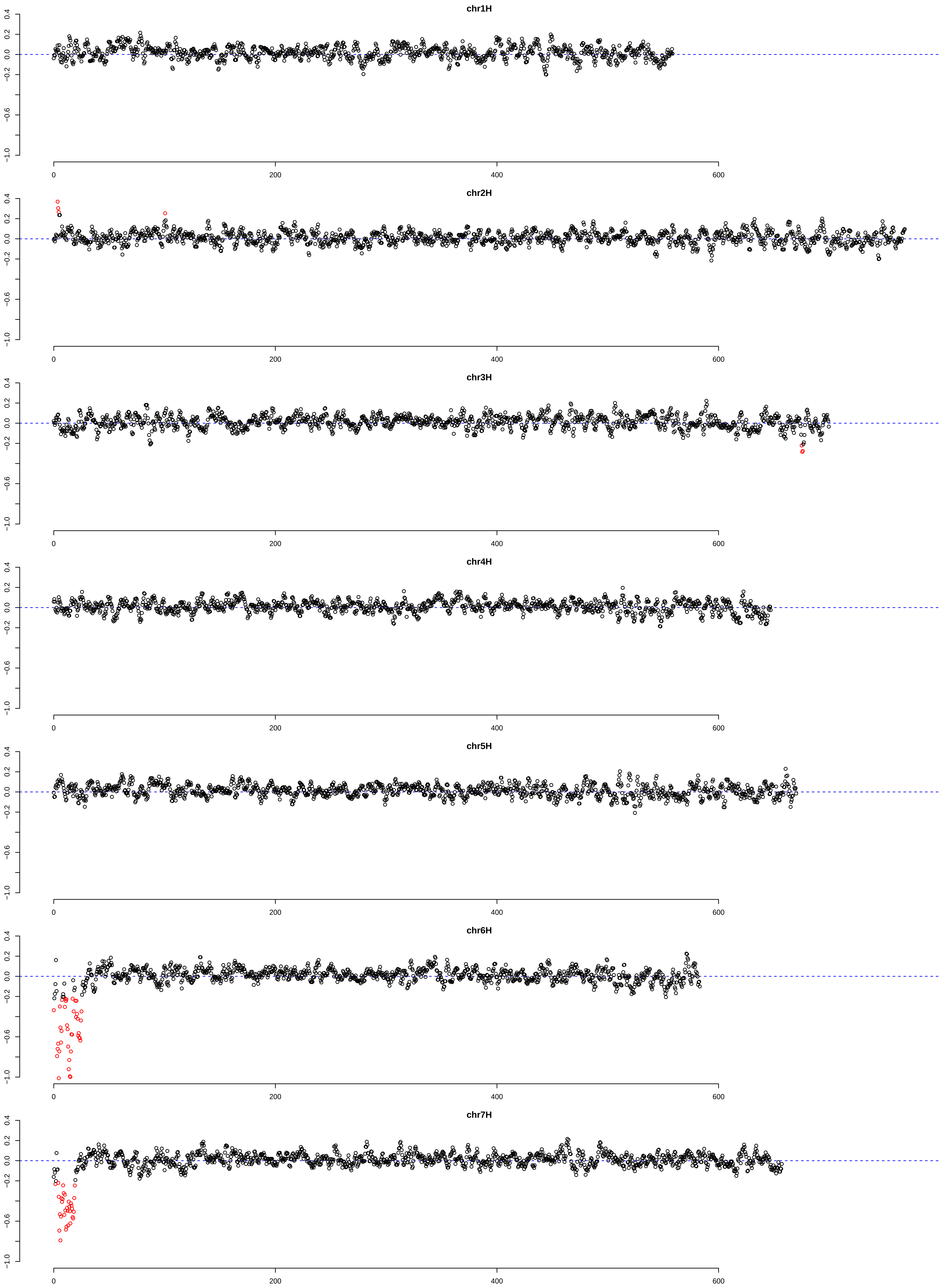
chr1H
chr2H
chr3H
chr4H
chr5H
chr6H
chr7H
0.4
0.2
0.0
-0.2
-0.6
-1.0
0
200
400
600

ERR699807

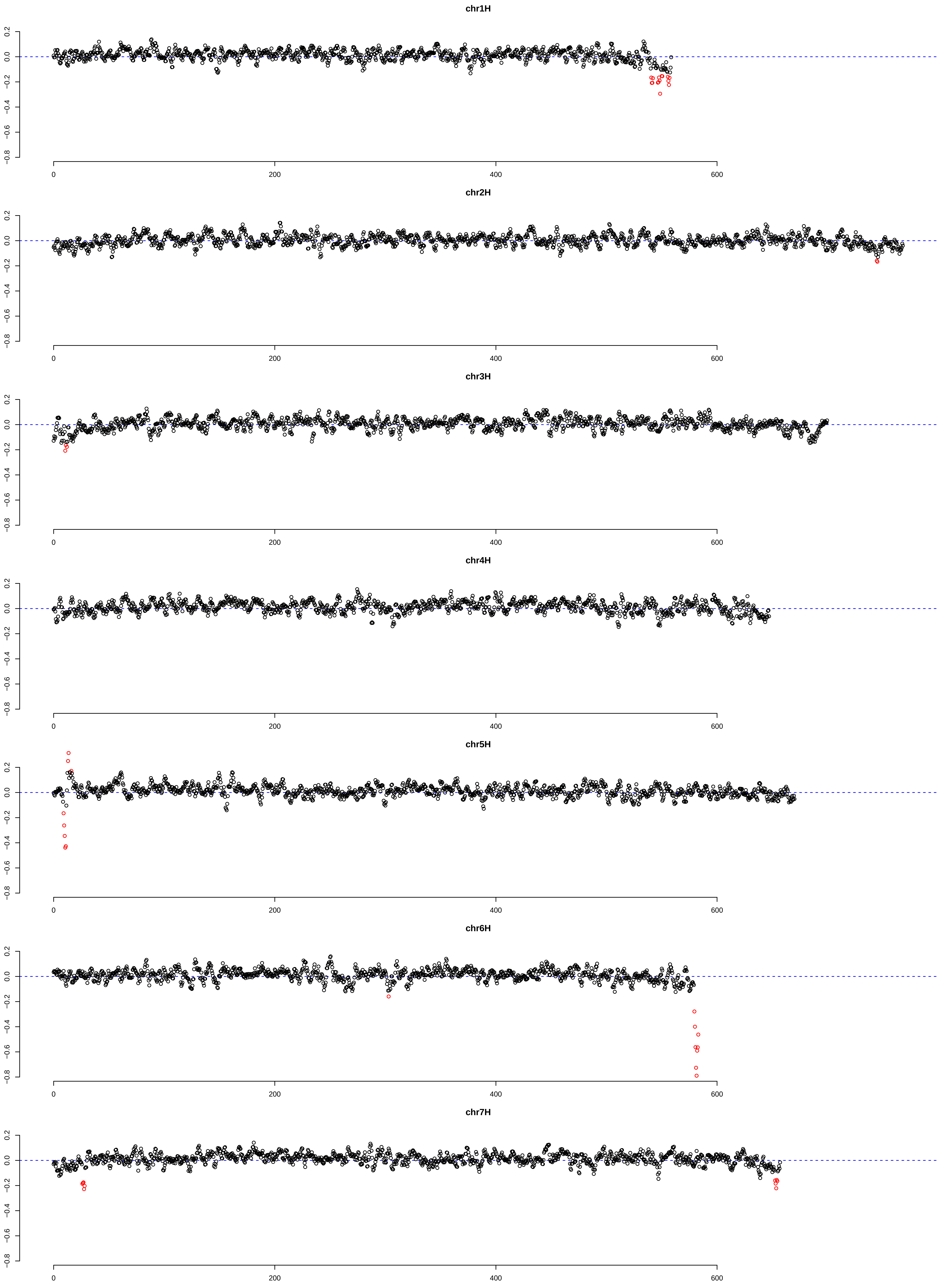
chr1H
chr2H
chr3H
chr4H
chr5H
chr6H
chr7H
0.2
0.0
-0.2
-0.4
-0.6
-0.8
0
200
400
600

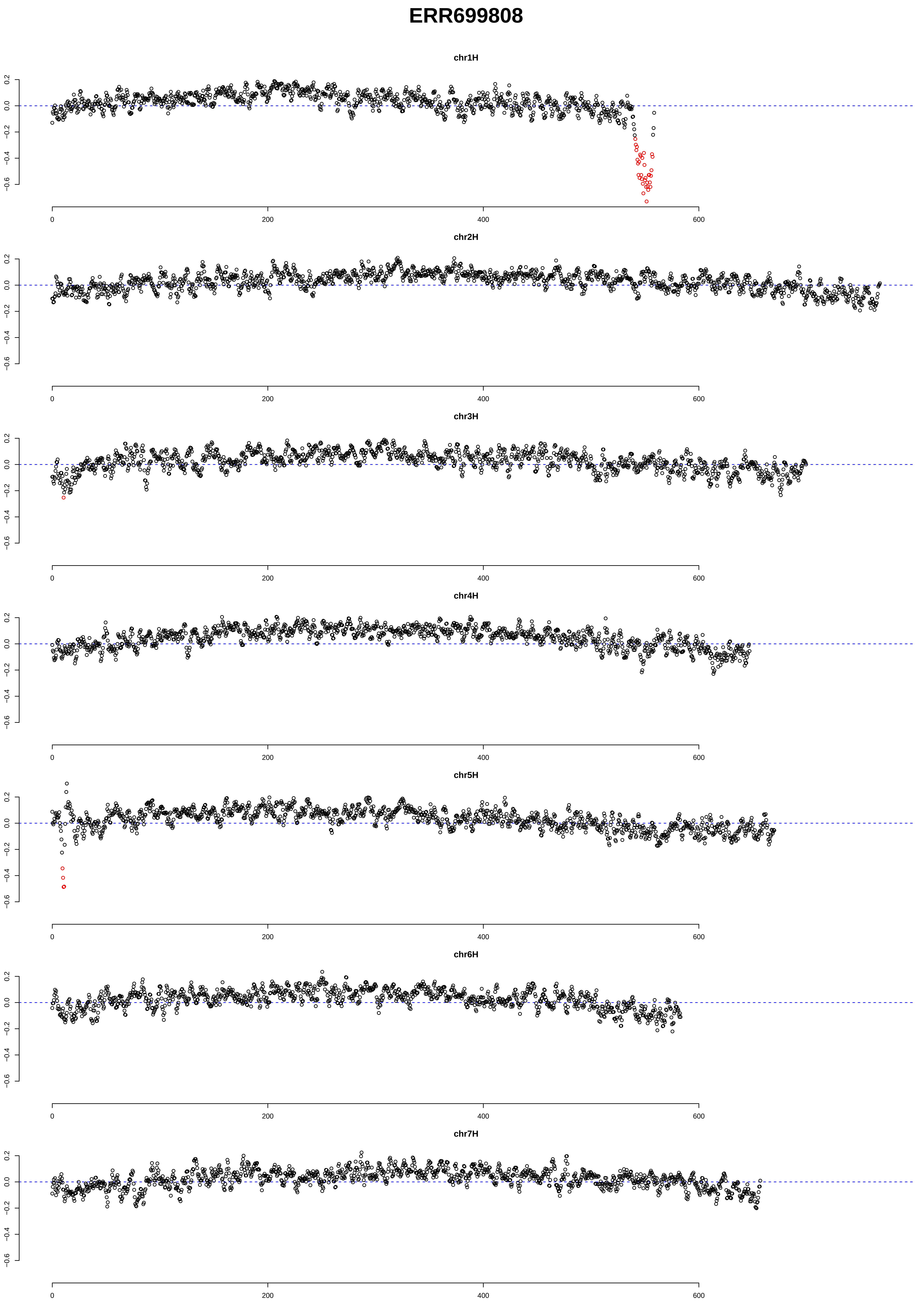
ERR699808
chr1H
chr2H
chr3H
chr4H
chr5H
chr6H
chr7H
0.2
0.0
-0.2
-0.4
-0.6
0
200
400
600

ERR699809

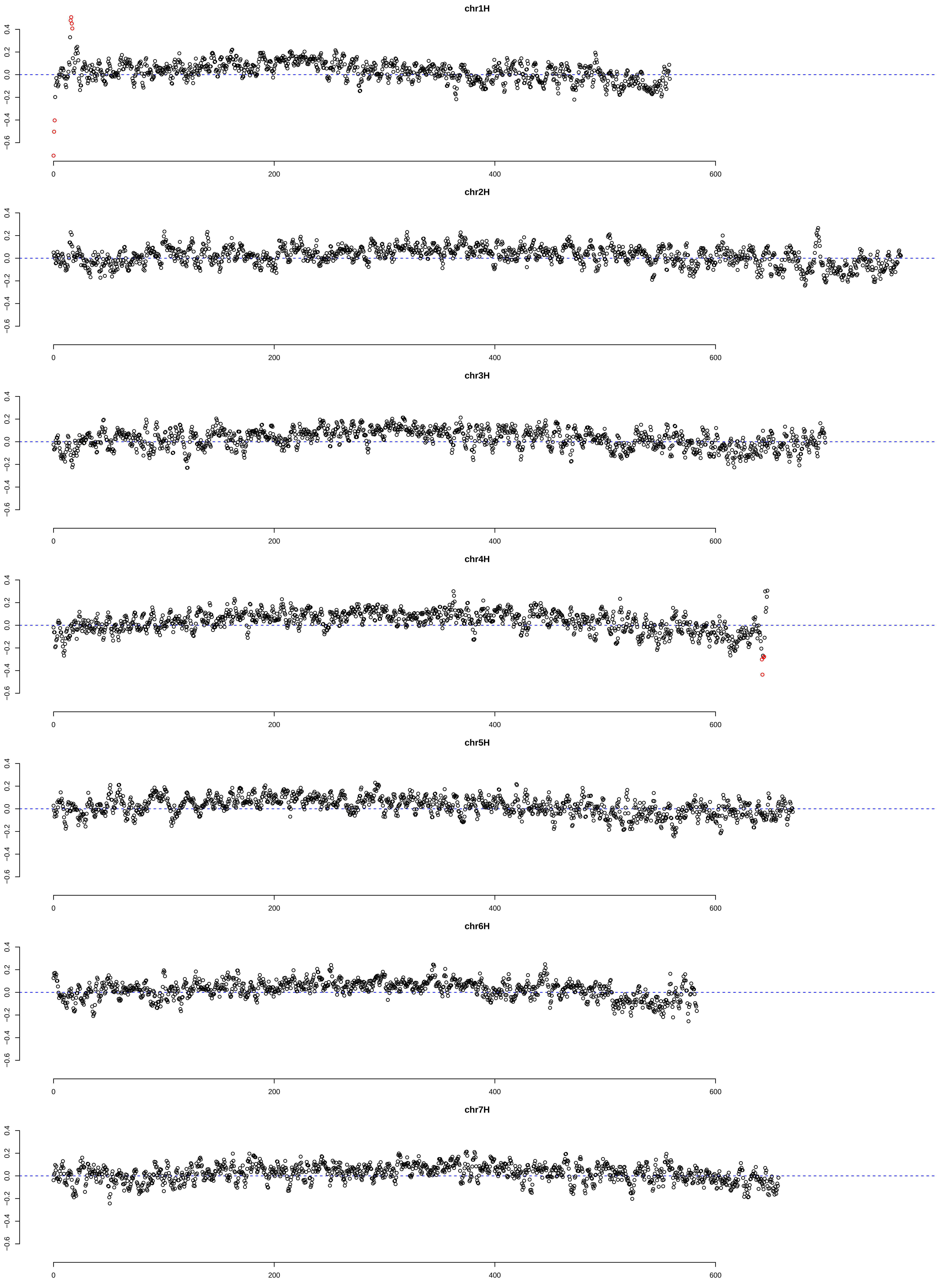
chr1H
chr2H
chr3H
chr4H
chr5H
chr6H
chr7H
0.4
0.2
0.0
-0.2
-0.4
-0.6
0
200
400
600

ERR699810

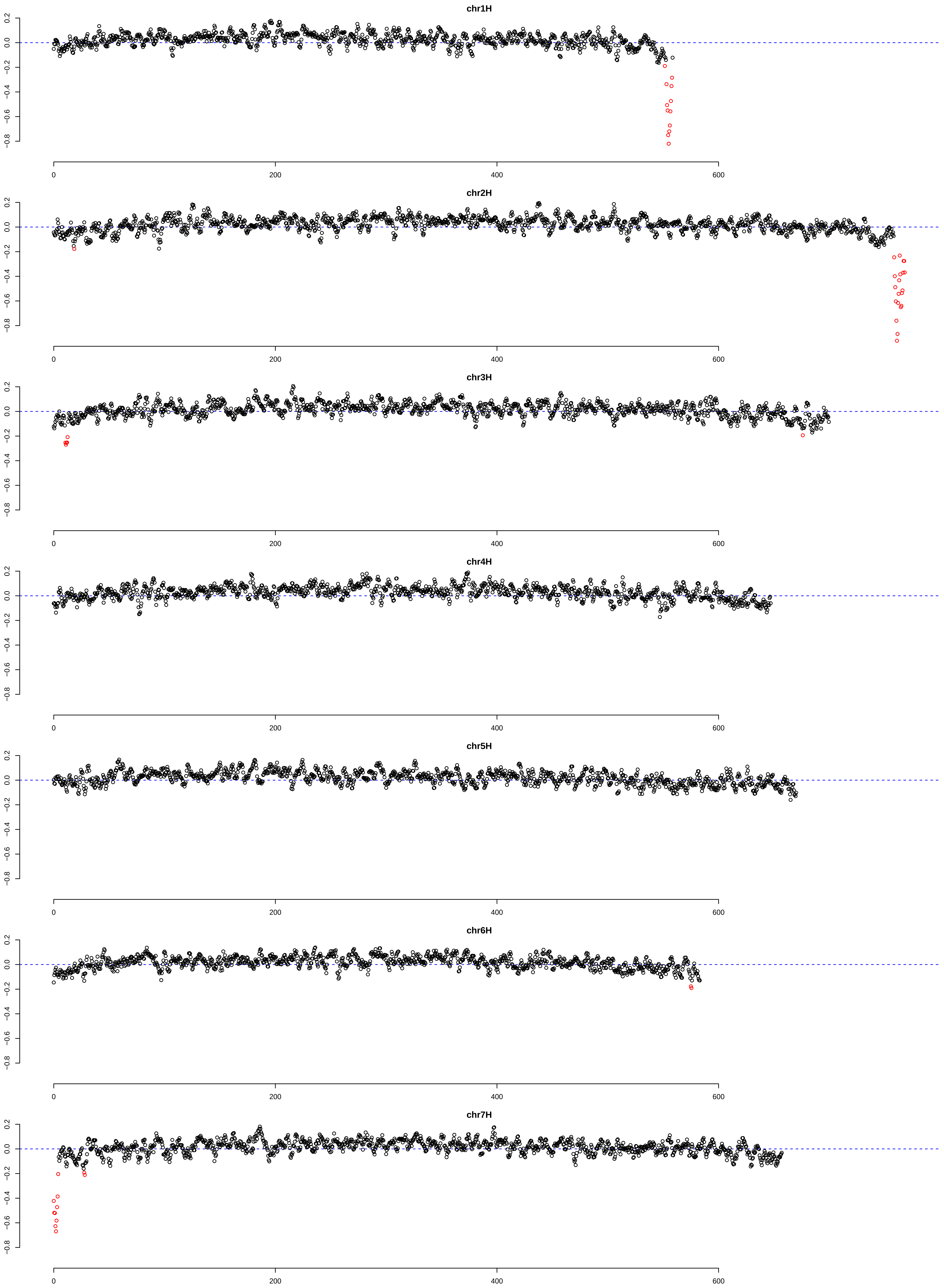
chr1H
chr2H
chr3H
chr4H
chr5H
chr6H
chr7H
0.2
0.0
-0.2
-0.4
-0.6
-0.8
0
200
400
600

ERR699811

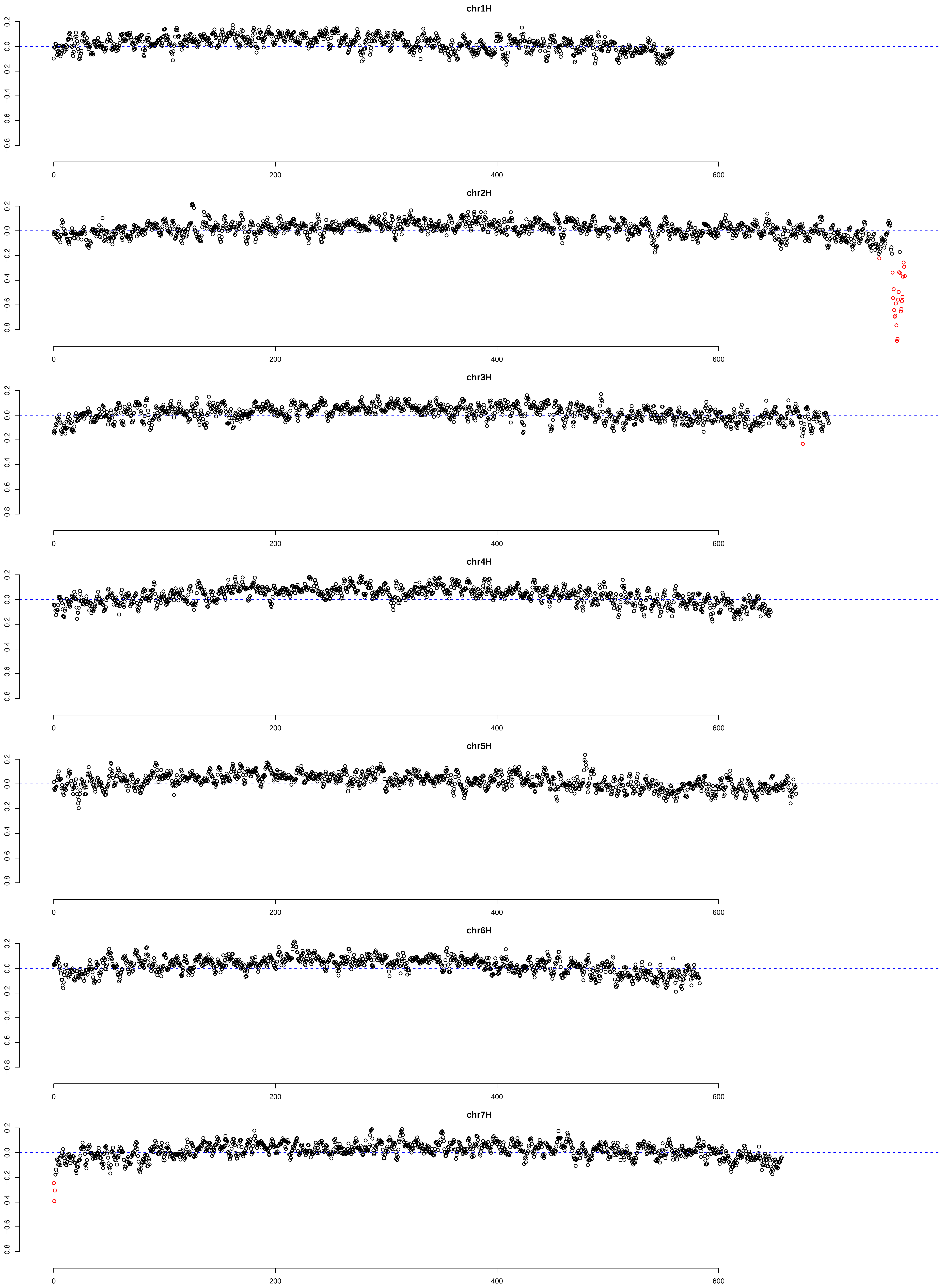
chr1H
chr2H
chr3H
chr4H
chr5H
chr6H
chr7H
0.2
0.0
-0.2
-0.4
-0.6
-0.8
0
200
400
600

ERR699812

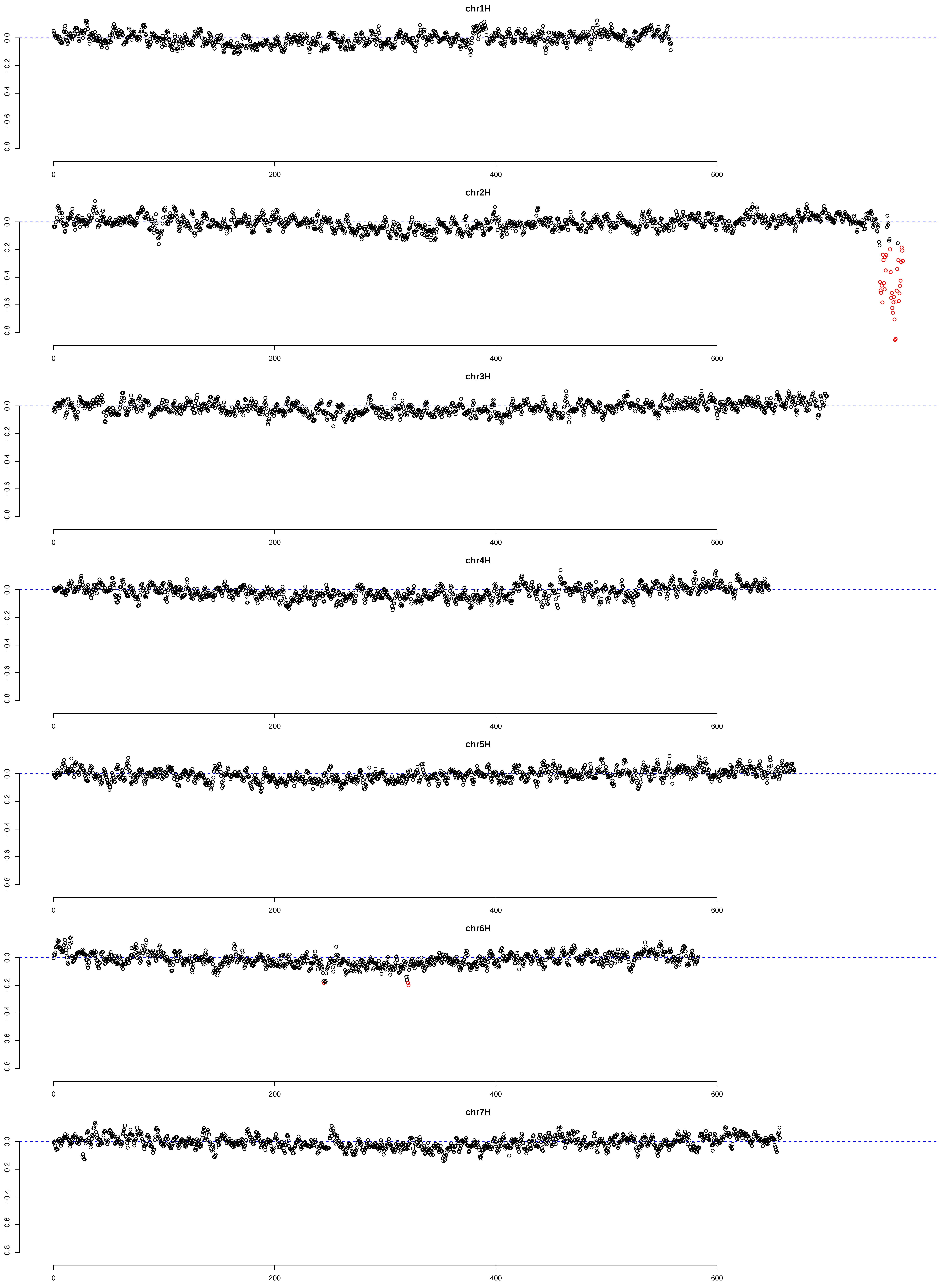

chr1H
chr2H
chr3H
chr4H
chr5H
chr6H
chr7H
0.0
-0.2
-0.4
-0.6
-0.8
0
200
400
600

ERR699813

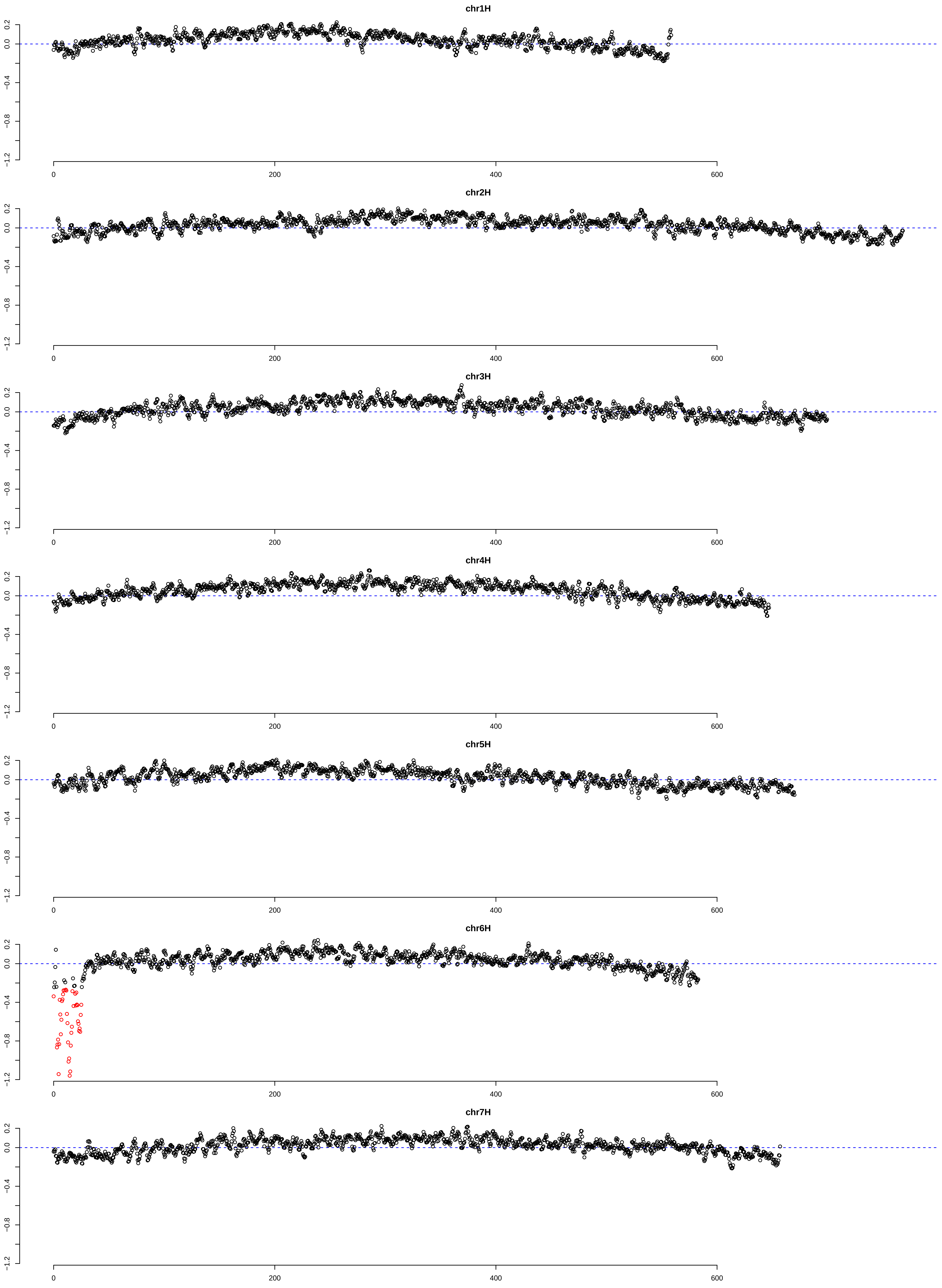

chr1H
chr2H
chr3H
chr4H
chr5H
chr6H
chr7H
0.2
0.0
-0.4
-0.8
-1.2
0
200
400
600

ERR699814

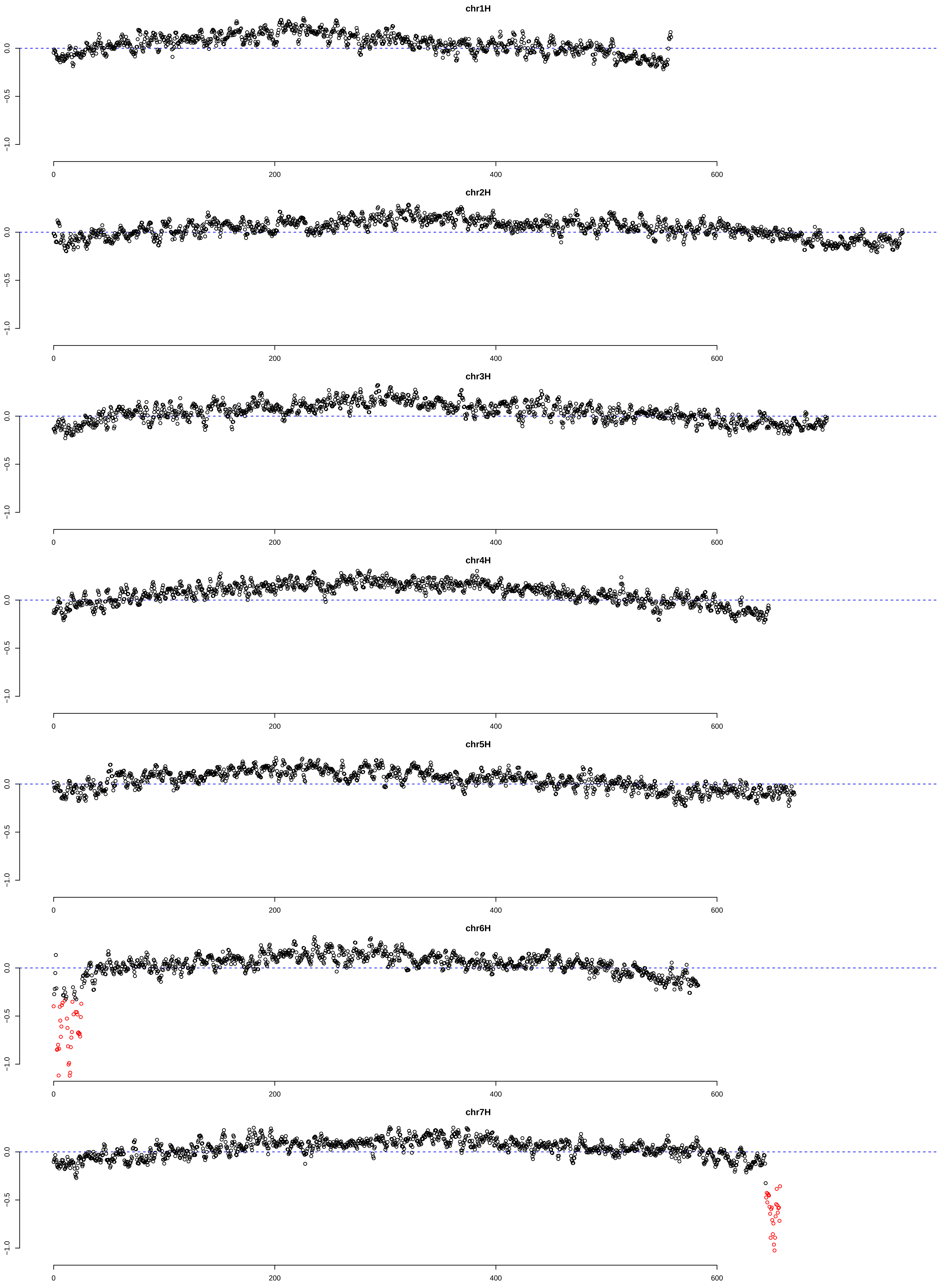
chr1H
chr2H
chr3H
chr4H
chr5H
chr6H
chr7H
0.0
-0.5
-1.0
0
200
400
600

ERR699815

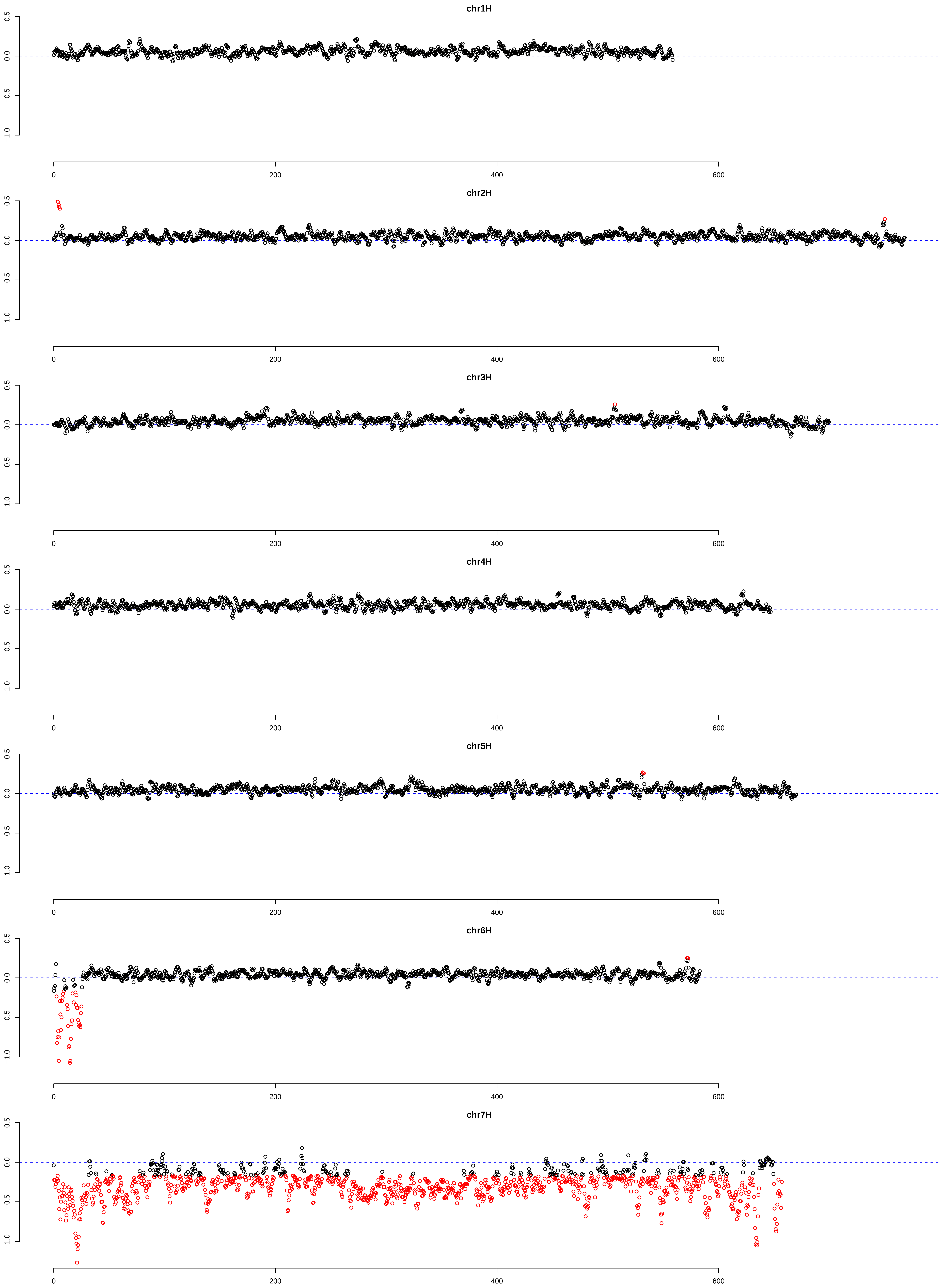

chr1H
chr2H
chr3H
chr4H
chr5H
chr6H
chr7H
0.5
0.0
-0.5
-1.0
0
200
400
600

ERR699816

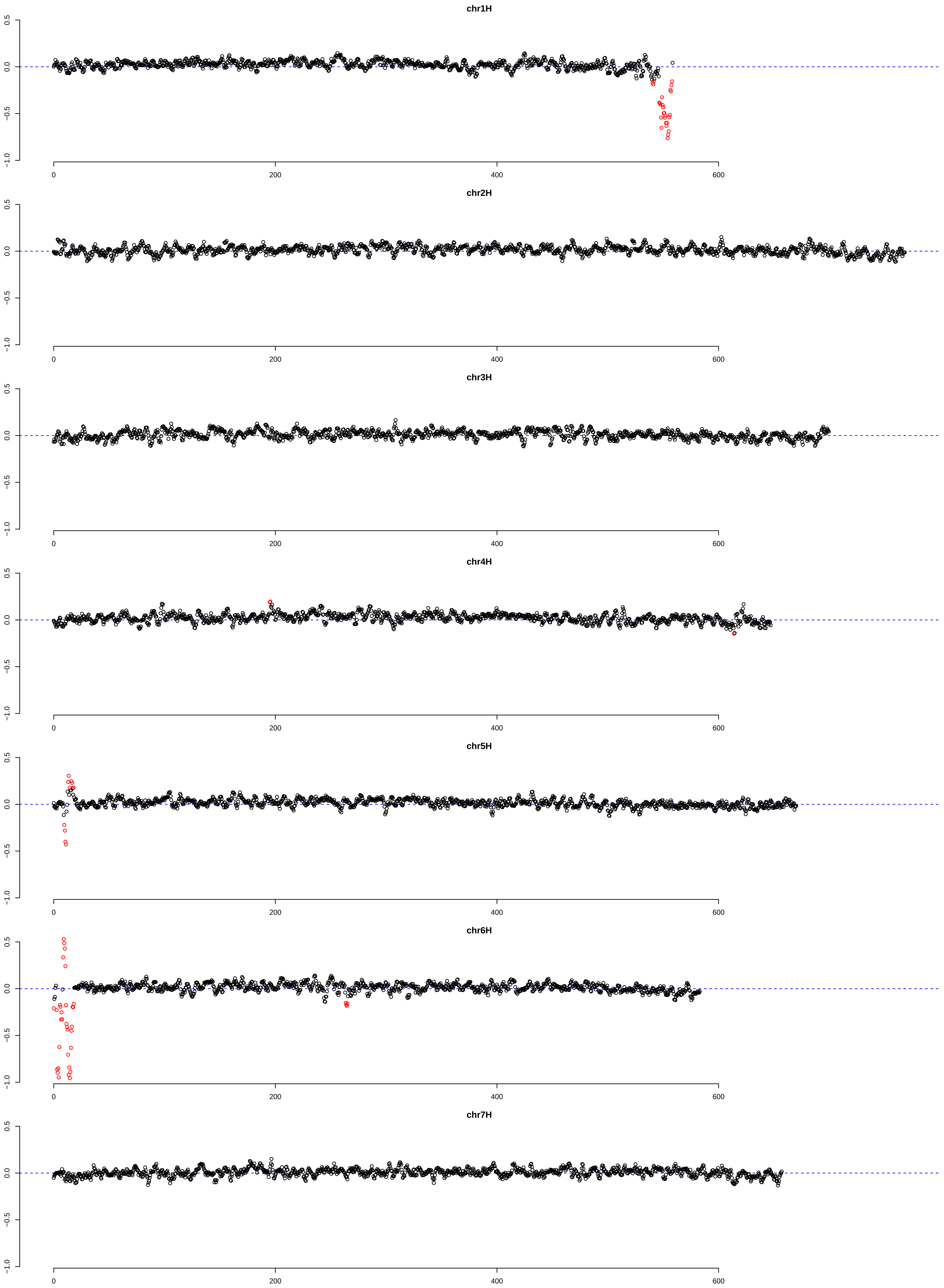
chr1H
chr2H
chr3H
chr4H
chr5H
chr6H
chr7H
0.5
0.0
-0.5
-1.0
0
200
400
600

ERR699817

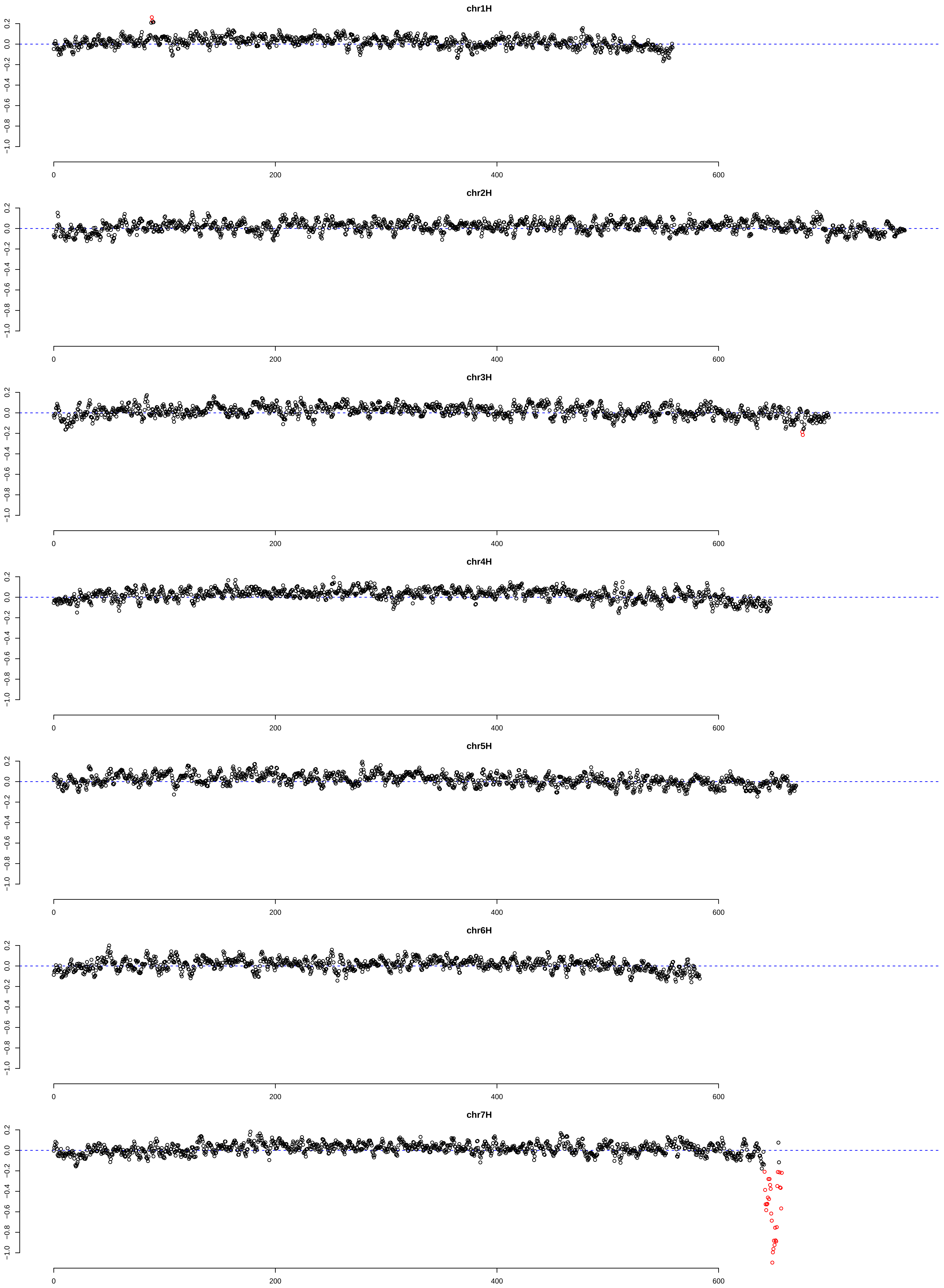

chr1H
chr2H
chr3H
chr4H
chr5H
chr6H
chr7H
0.2
0.0
−0.2
−0.4
−0.6
−0.8
−1.0
0
200
400
600

ERR699818

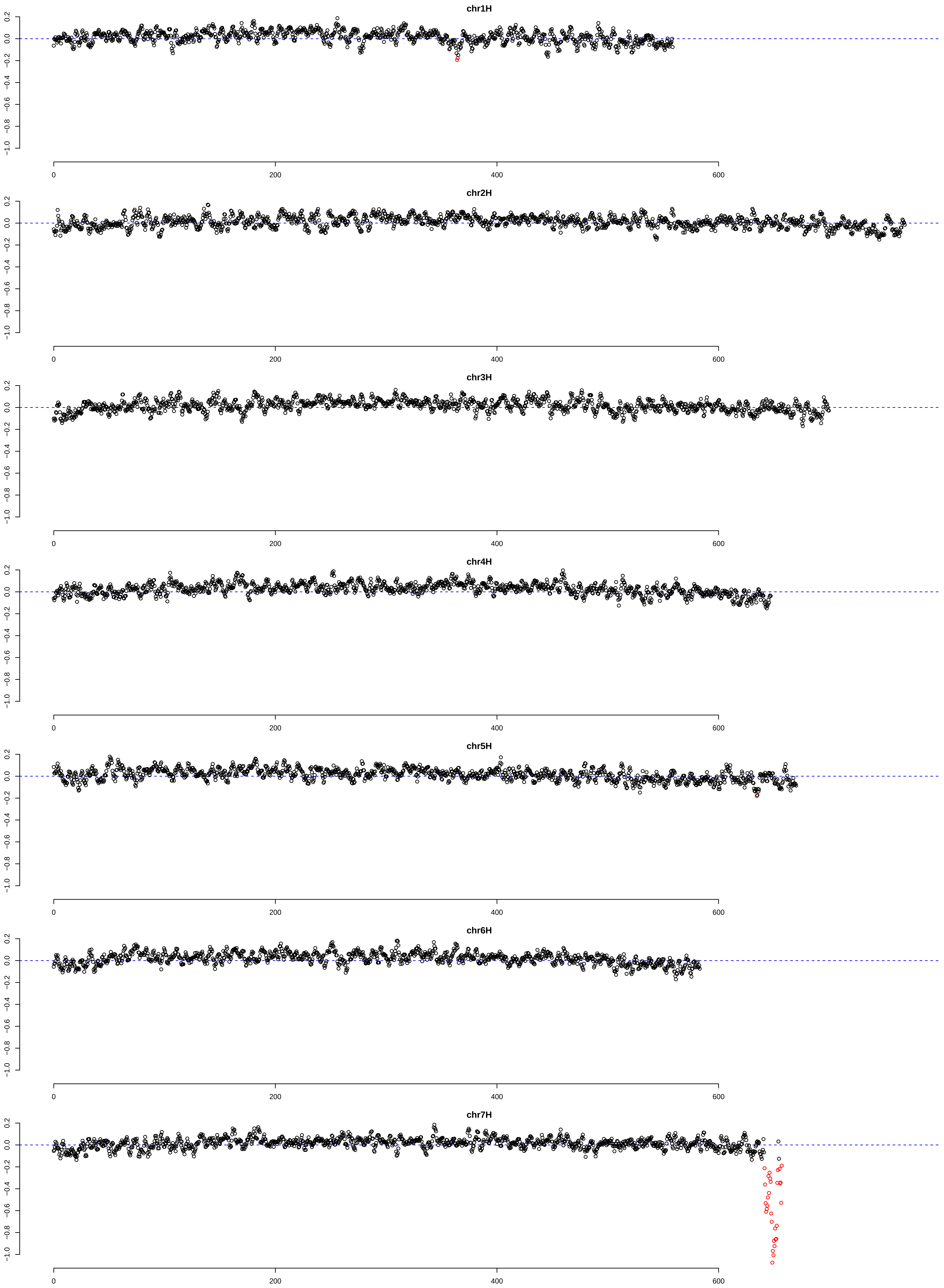
chr1H
chr2H
chr3H
chr4H
chr5H
chr6H
chr7H
0
200
400
600
0.2
0.0
−0.2
−0.4
−0.6
−0.8
−1.0

ERR699819

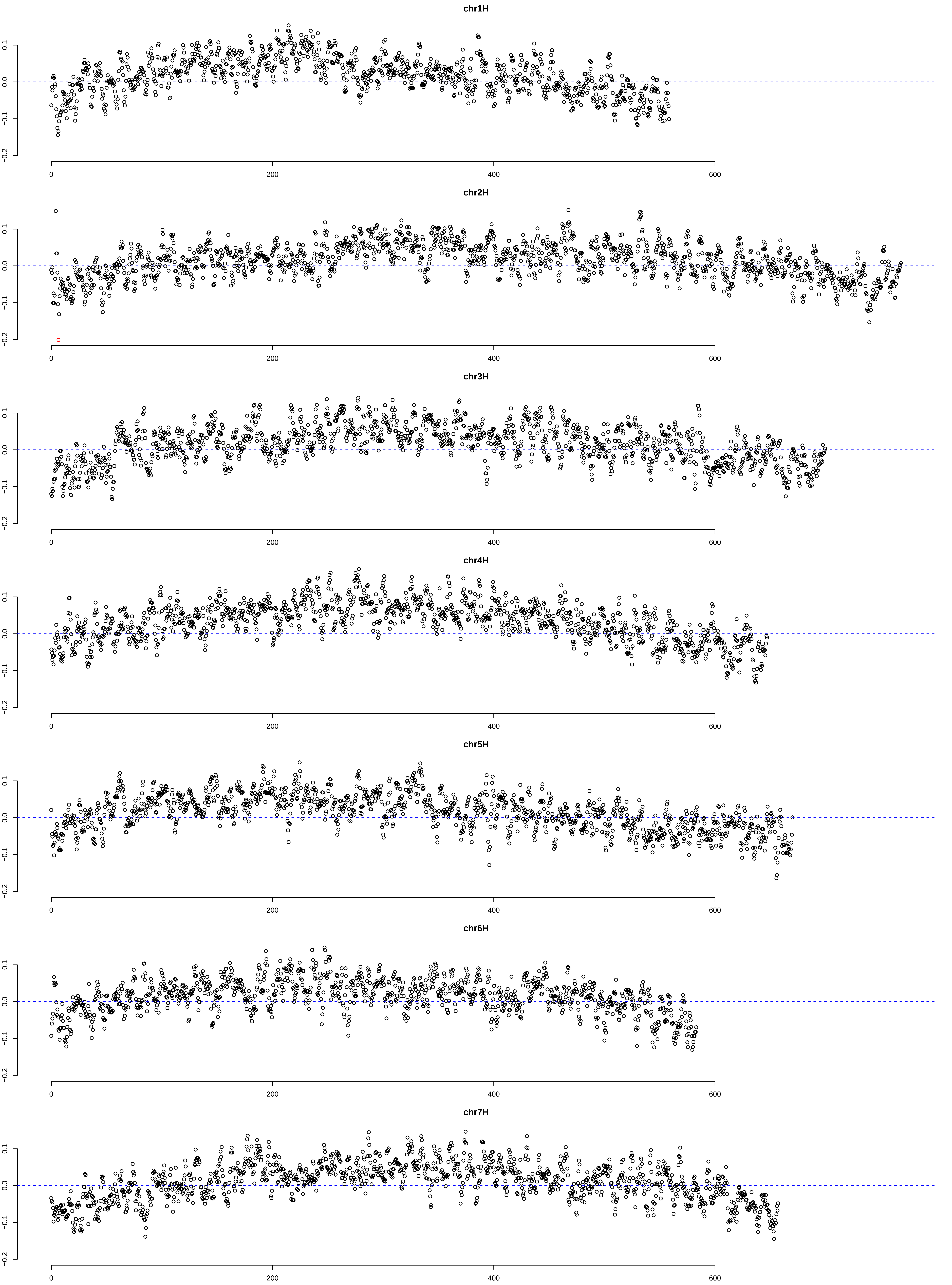
chr1H
chr2H
chr3H
chr4H
chr5H
chr6H
chr7H
0.1
0.0
-0.1
-0.2
0
200
400
600

ERR699820

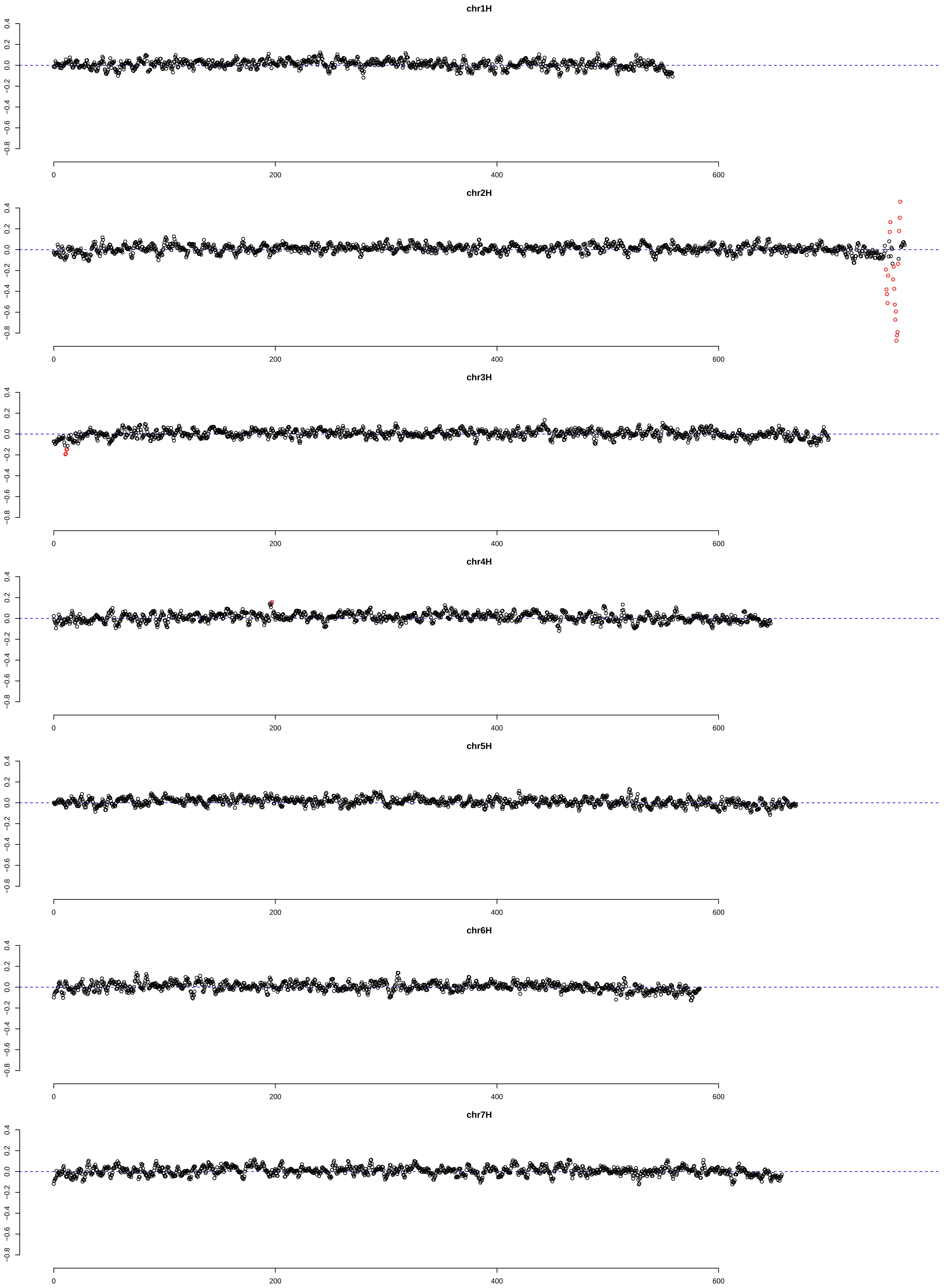
chr1H
chr2H
chr3H
chr4H
chr5H
chr6H
chr7H
0.4
0.2
0.0
−0.2
−0.4
−0.6
−0.8
0
200
400
600

ERR699821

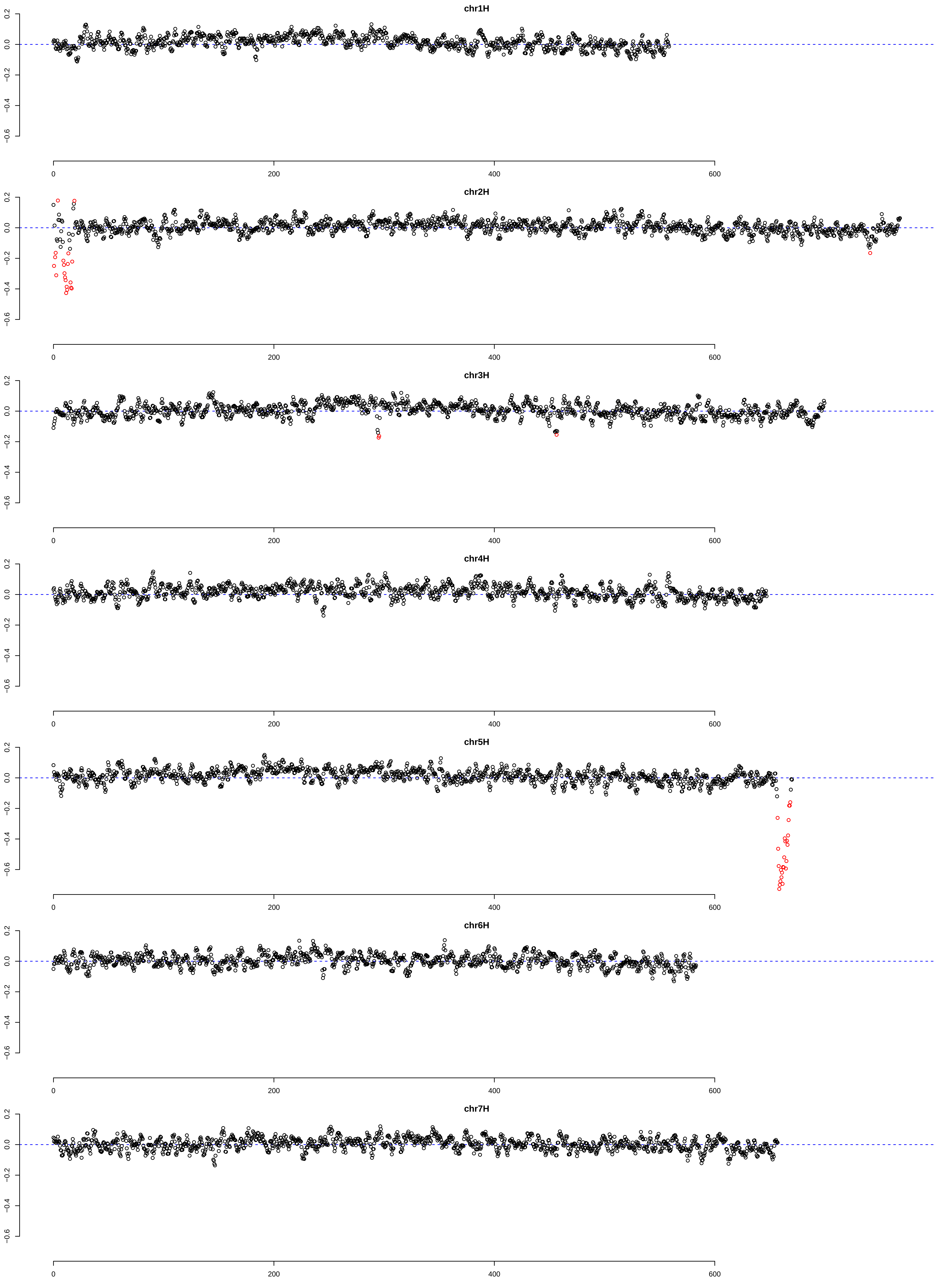
chr1H
chr2H
chr3H
chr4H
chr5H
chr6H
chr7H
0.2
0.0
−0.2
−0.4
−0.6
0
200
400
600

ERR699822

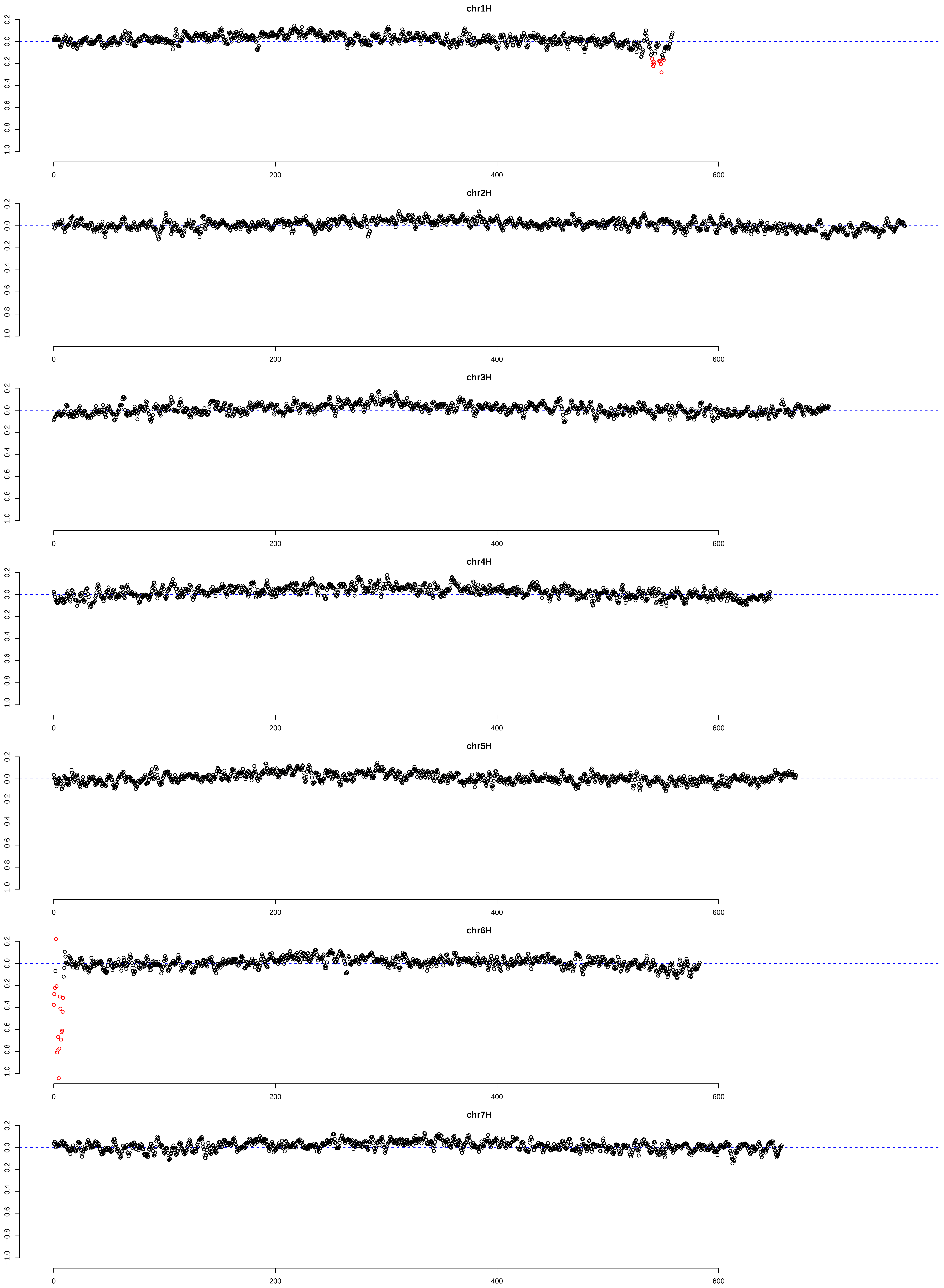
chr1H
chr2H
chr3H
chr4H
chr5H
chr6H
chr7H
0.2
0.0
-0.2
-0.4
-0.6
-0.8
-1.0
0
200
400
600

ERR699823

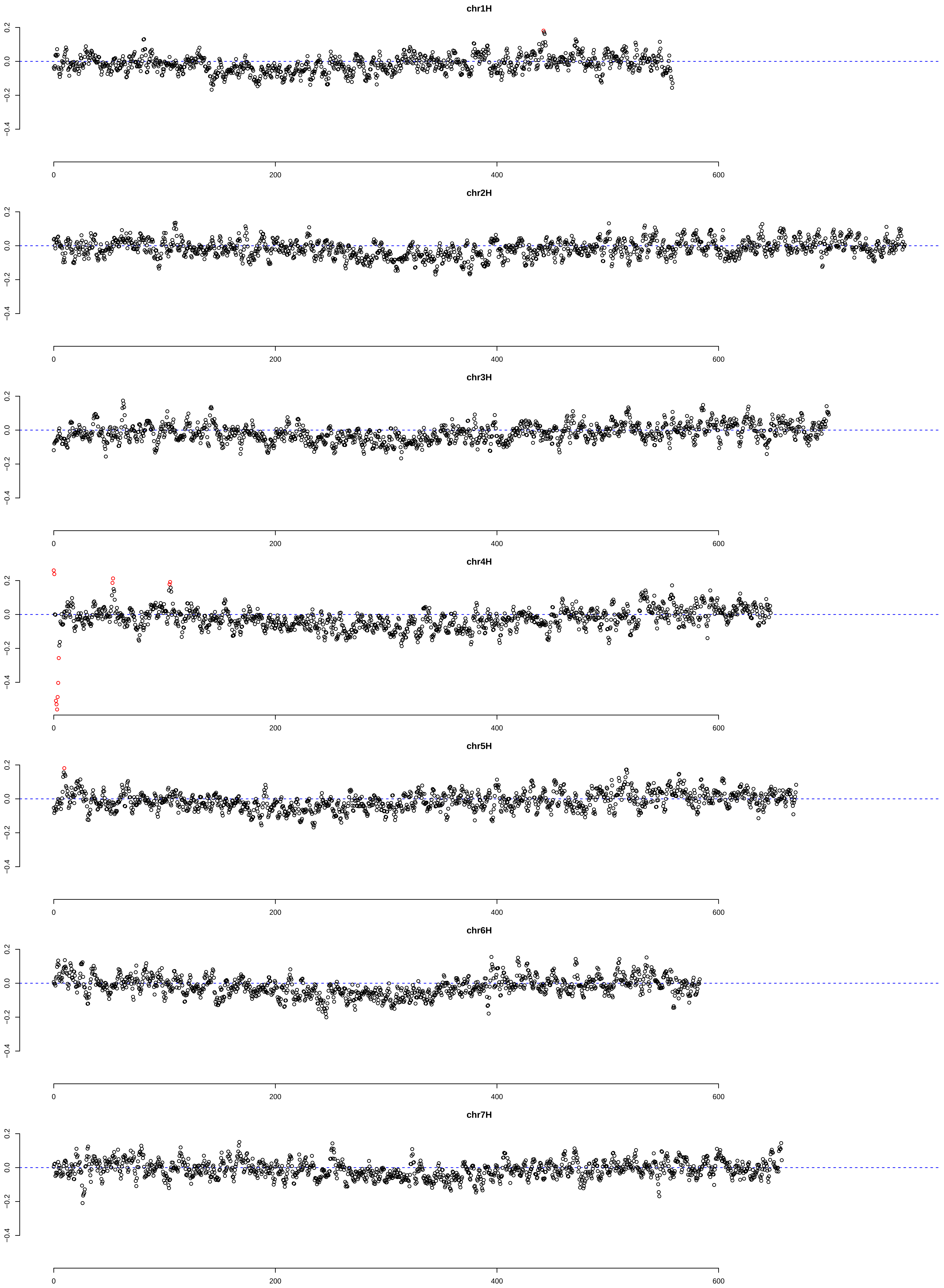

chr1H
chr2H
chr3H
chr4H
chr5H
chr6H
chr7H
0.2
0.0
-0.2
-0.4
0
200
400
600

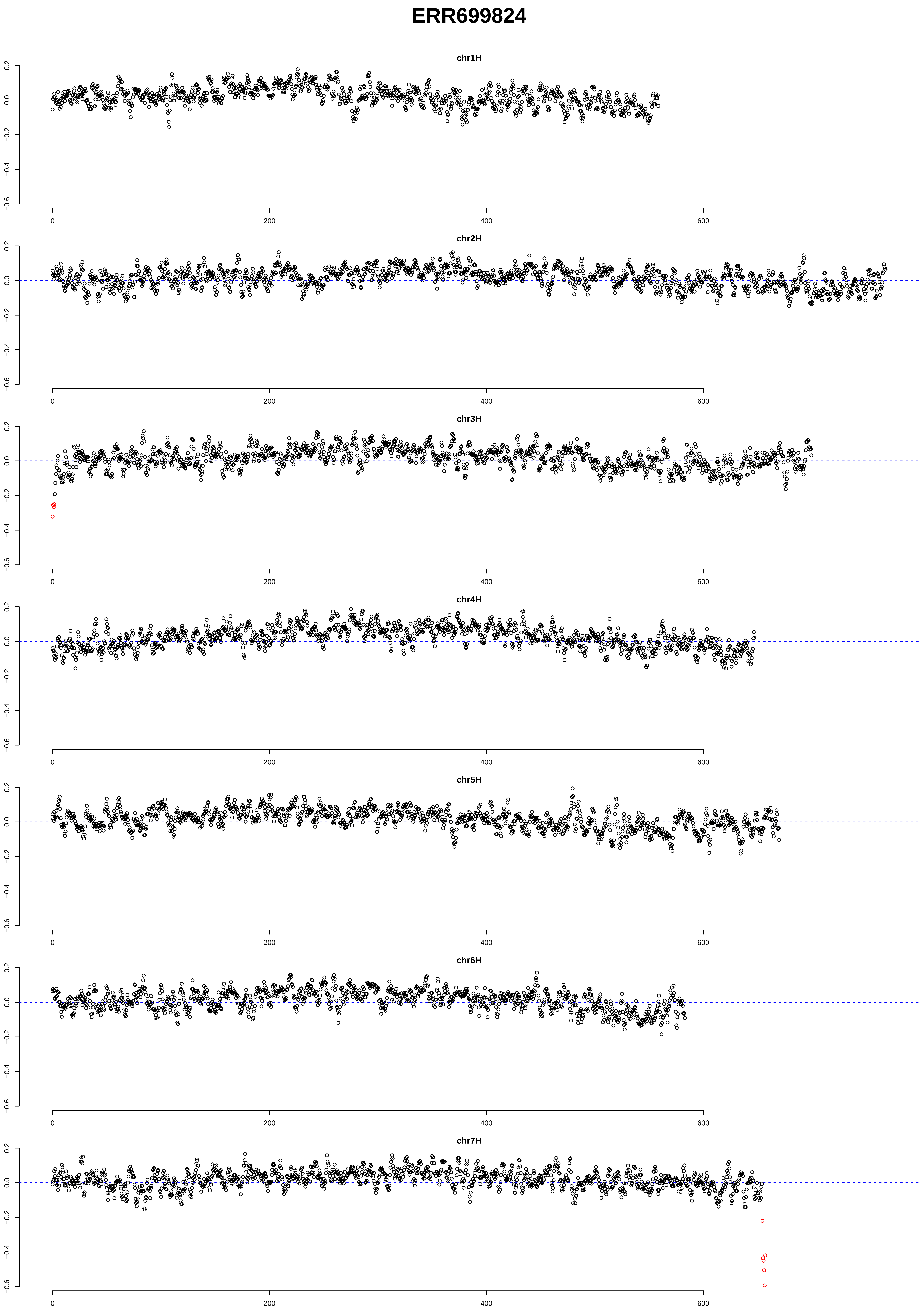
ERR699824
chr1H
chr2H
chr3H
chr4H
chr5H
chr6H
chr7H
0.2
0.0
-0.2
-0.4
-0.6
0
200
400
600

ERR699825

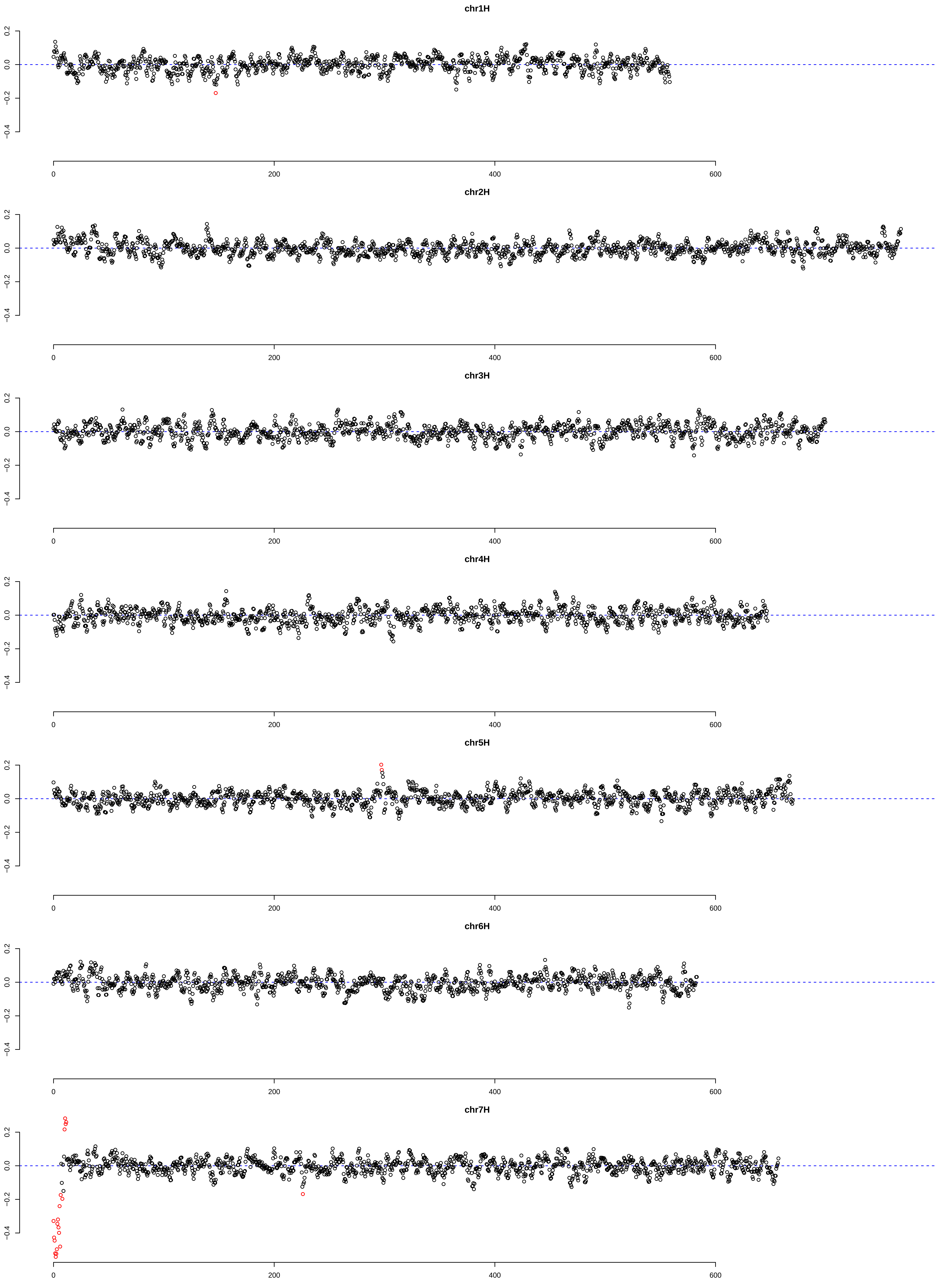
chr1H
chr2H
chr3H
chr4H
chr5H
chr6H
chr7H
0.2
0.0
-0.2
-0.4
0
200
400
600

ERR699826

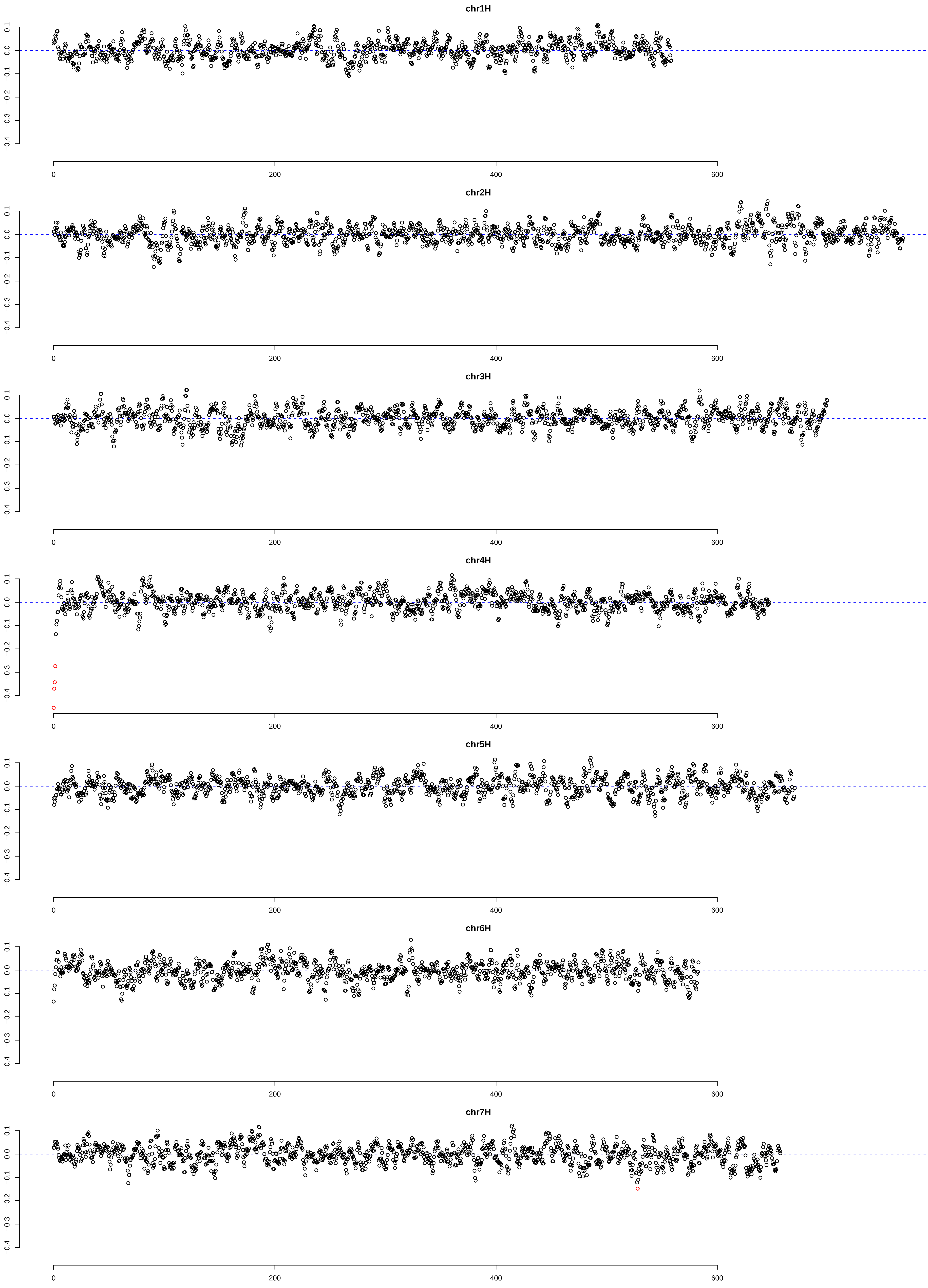
chr1H
chr2H
chr3H
chr4H
chr5H
chr6H
chr7H
0.1
0.0
-0.1
-0.2
-0.3
-0.4
0
200
400
600

ERR699827

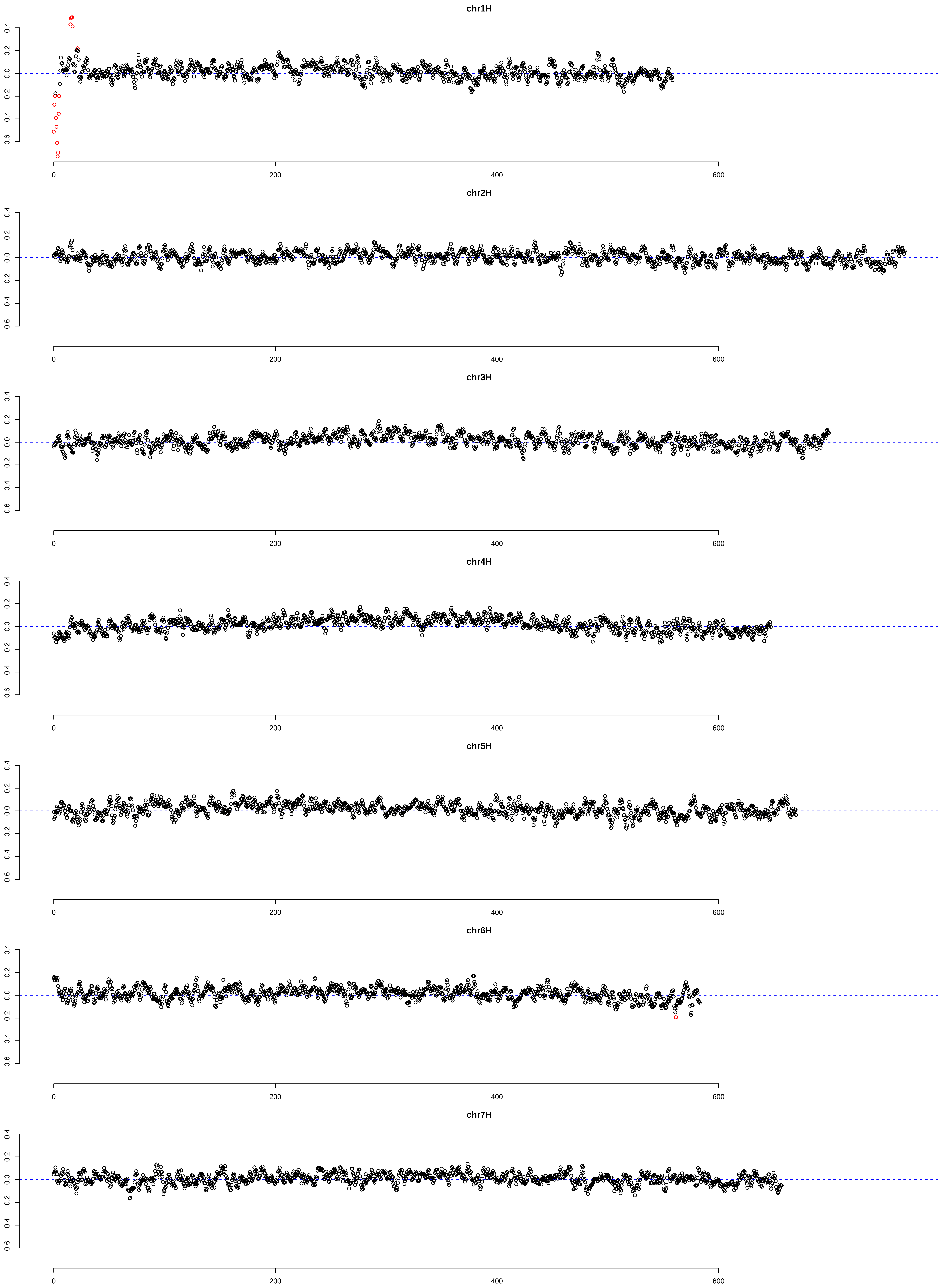
chr1H
chr2H
chr3H
chr4H
chr5H
chr6H
chr7H
0.4
0.2
0.0
−0.2
−0.4
−0.6
0
200
400
600

ERR699828

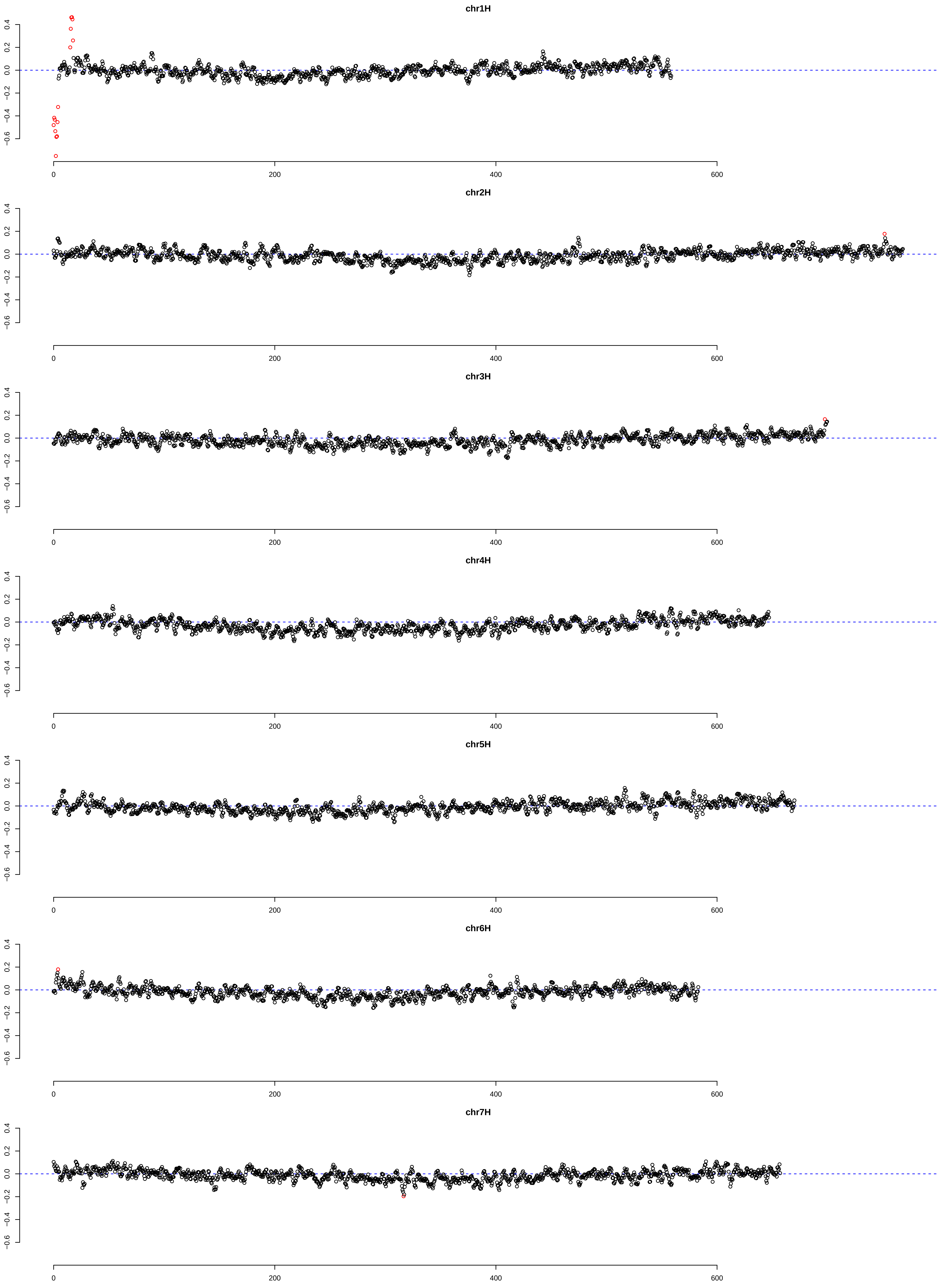

chr1H
chr2H
chr3H
chr4H
chr5H
chr6H
chr7H
0.4
0.2
0.0
-0.2
-0.4
-0.6
0
200
400
600

ERR699829

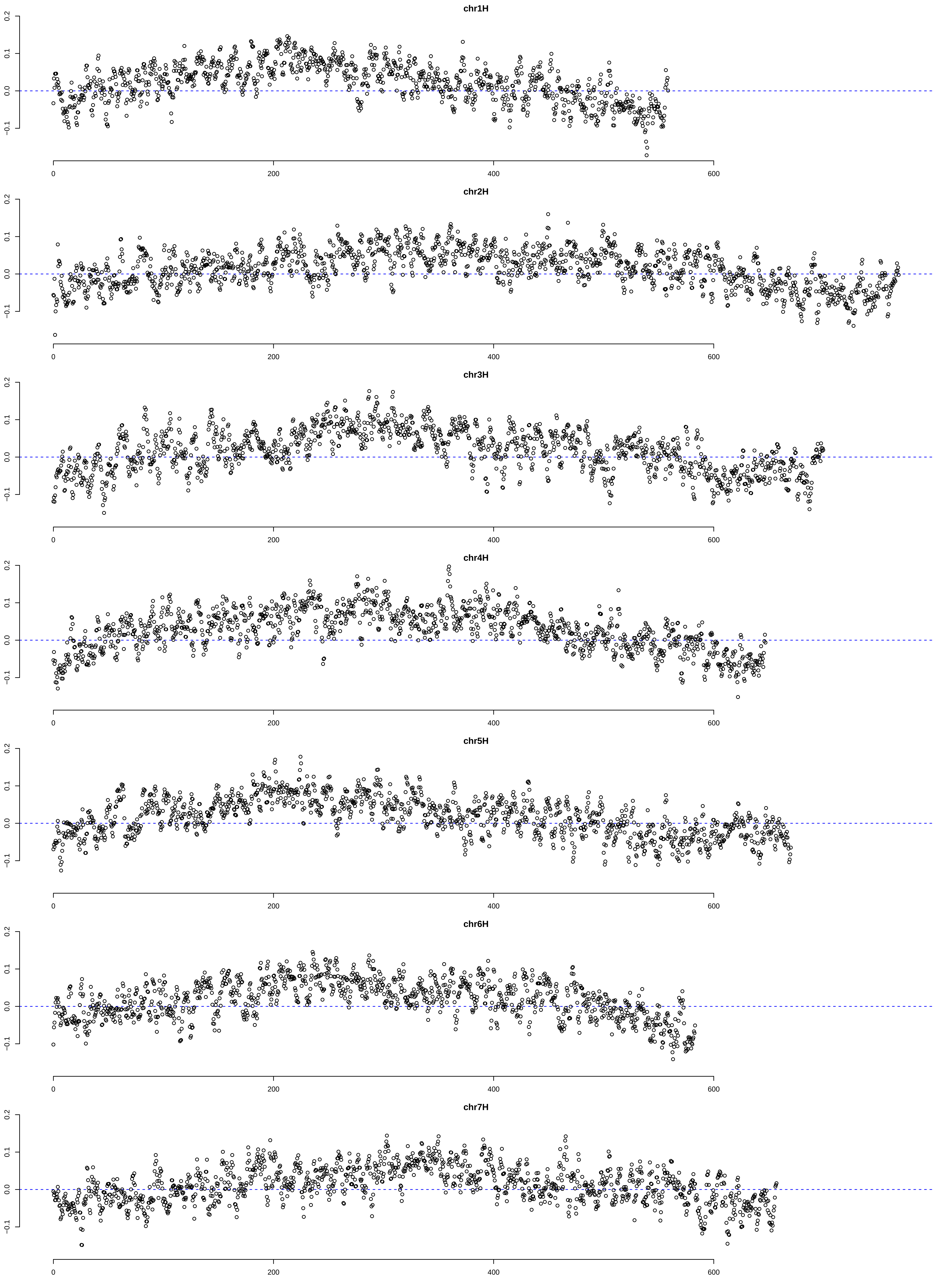
chr1H
chr2H
chr3H
chr4H
chr5H
chr6H
chr7H
0
200
400
600
-0.1
0.0
0.1
0.2

ERR699830

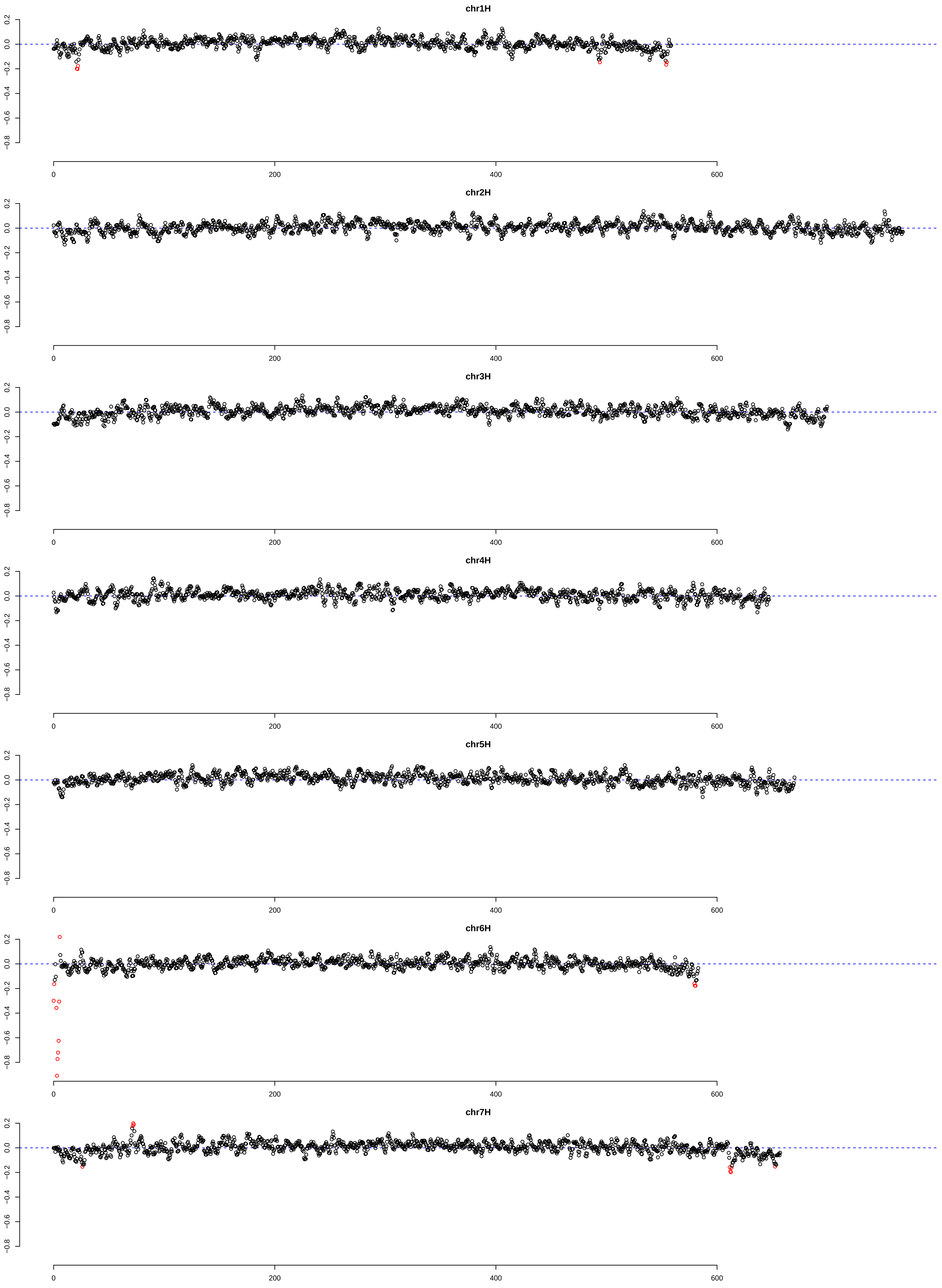

chr1H
chr2H
chr3H
chr4H
chr5H
chr6H
chr7H
0.2
0.0
-0.2
-0.4
-0.6
-0.8
0
200
400
600

ERR699831

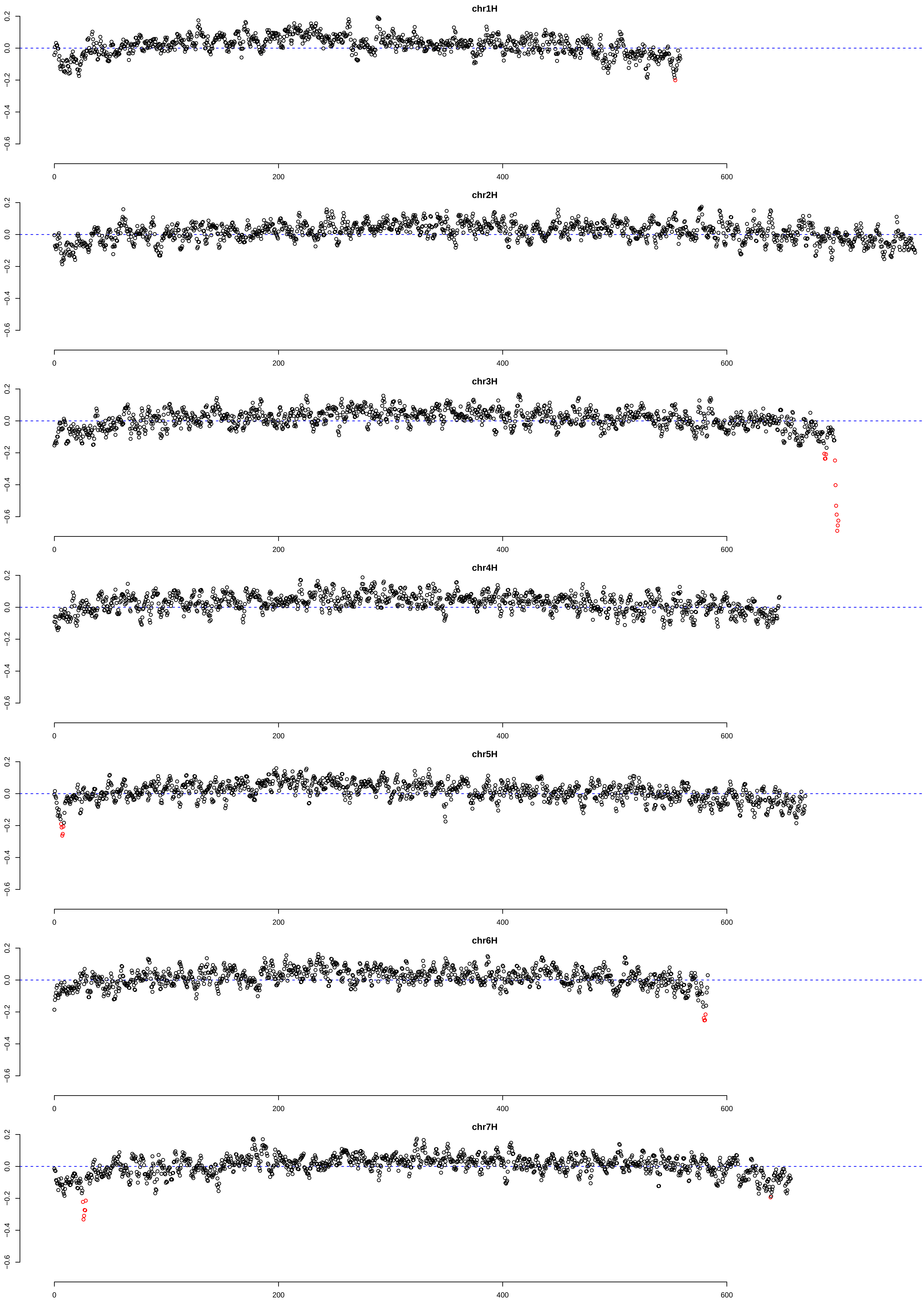
chr1H
chr2H
chr3H
chr4H
chr5H
chr6H
chr7H
0.2
0.0
-0.2
-0.4
-0.6
0
200
400
600

ERR699832

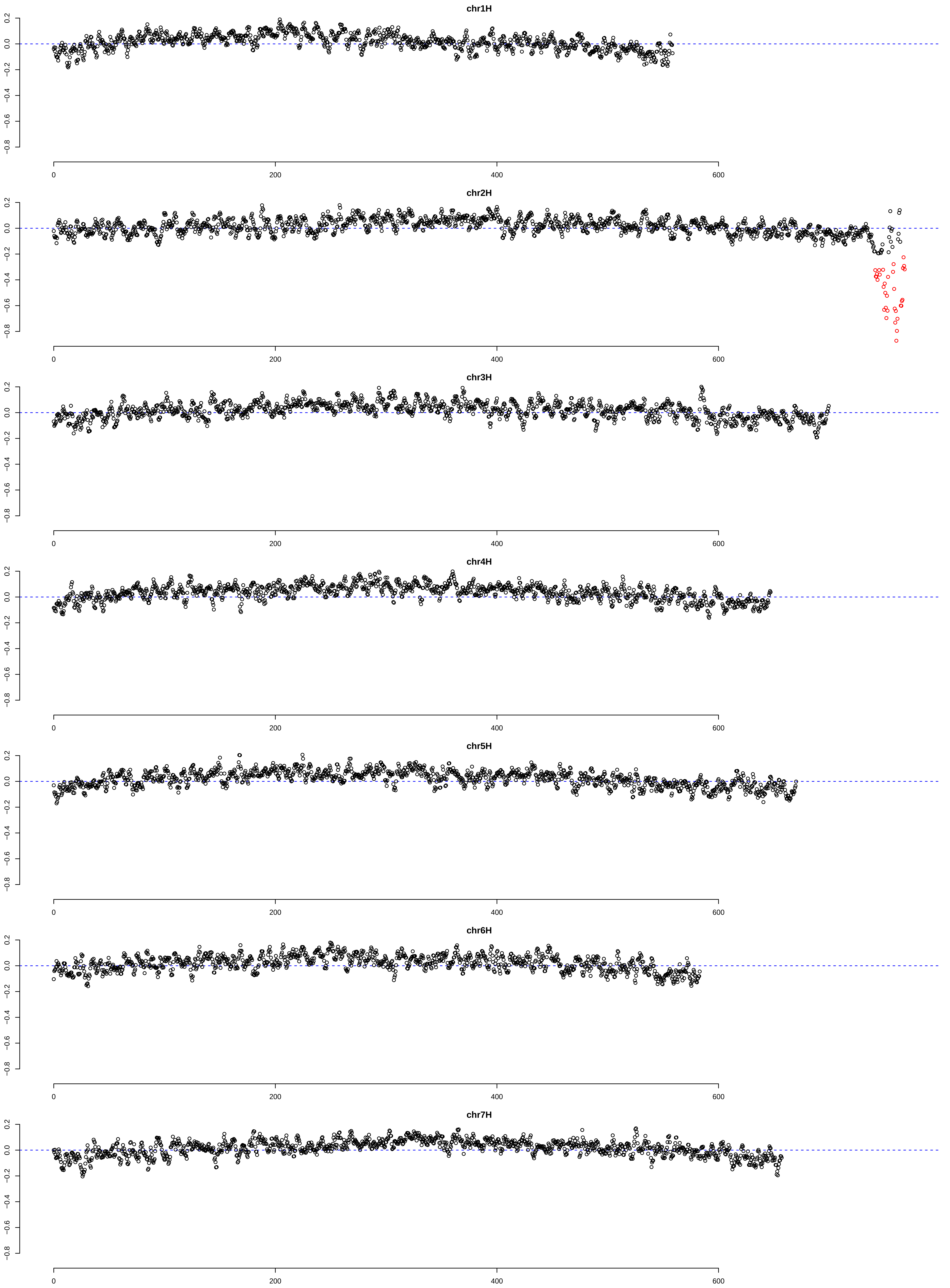

chr1H
chr2H
chr3H
chr4H
chr5H
chr6H
chr7H
0.2
0.0
-0.2
-0.4
-0.6
-0.8
0
200
400
600

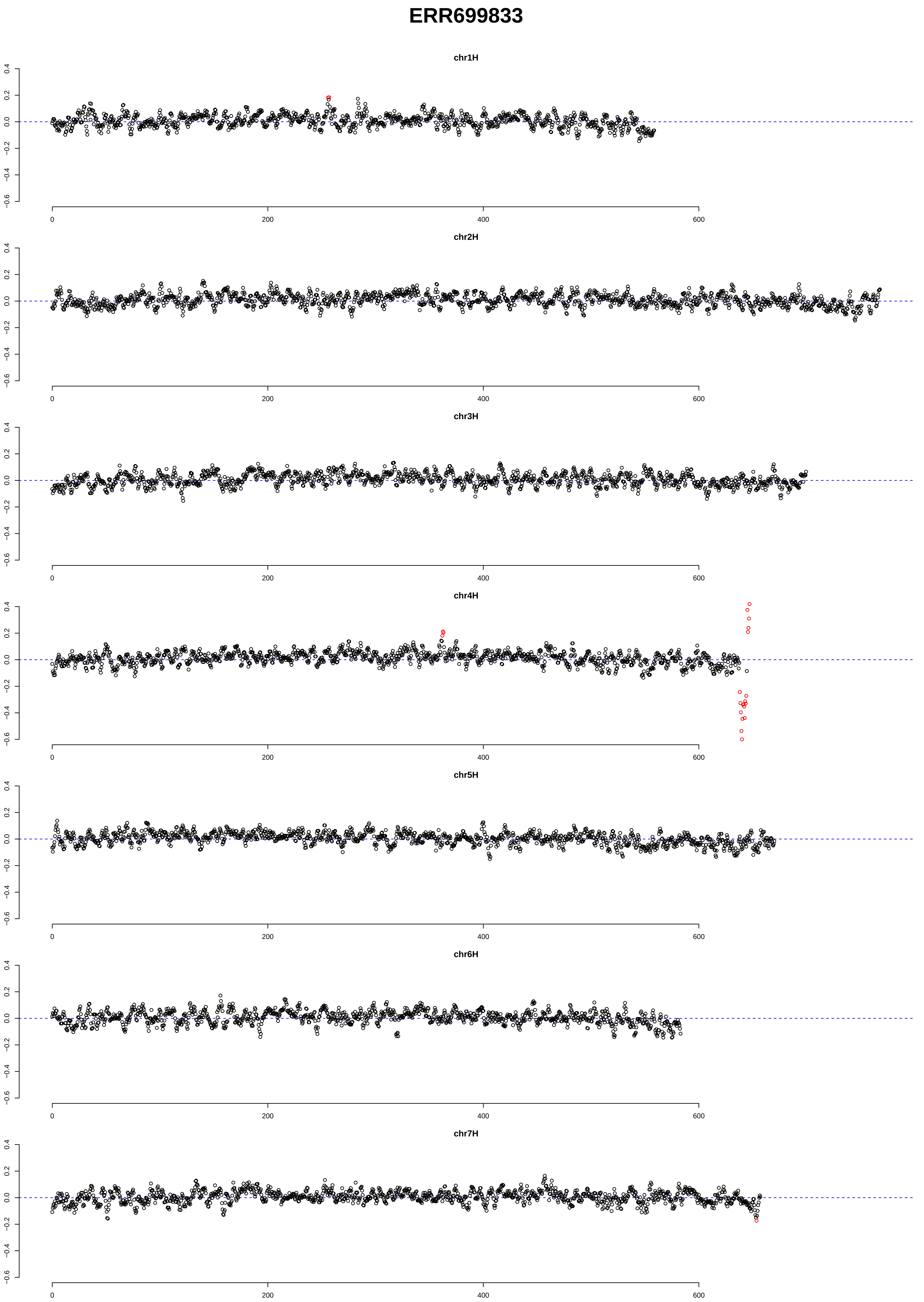
ERR699833
chr1H
chr2H
chr3H
chr4H
chr5H
chr6H
chr7H
0.4
0.2
0.0
−0.2
−0.4
−0.6
0
200
400
600

ERR699834

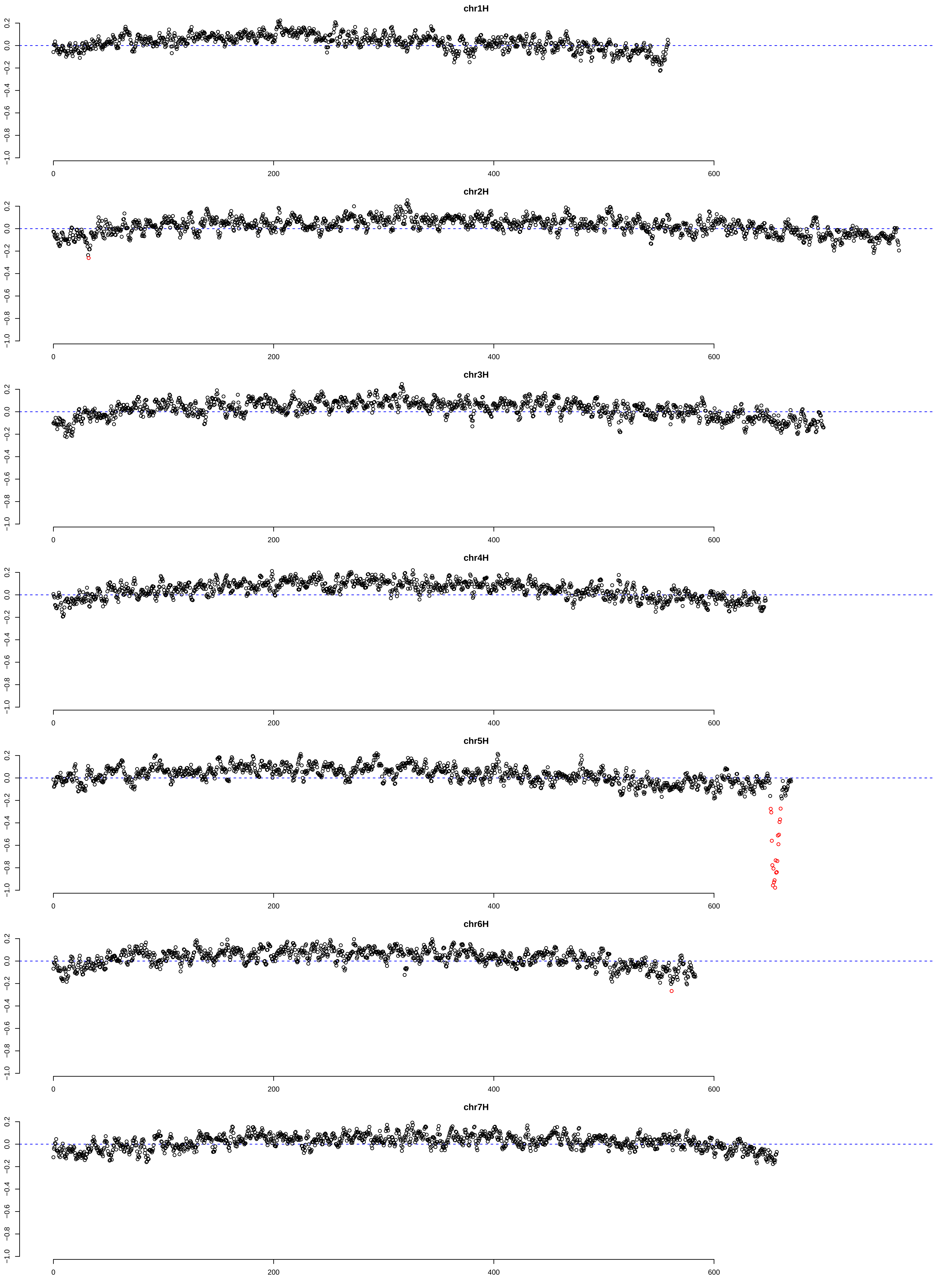
chr1H
chr2H
chr3H
chr4H
chr5H
chr6H
chr7H
0.2
0.0
-0.2
-0.4
-0.6
-0.8
-1.0
0
200
400
600

ERR699835

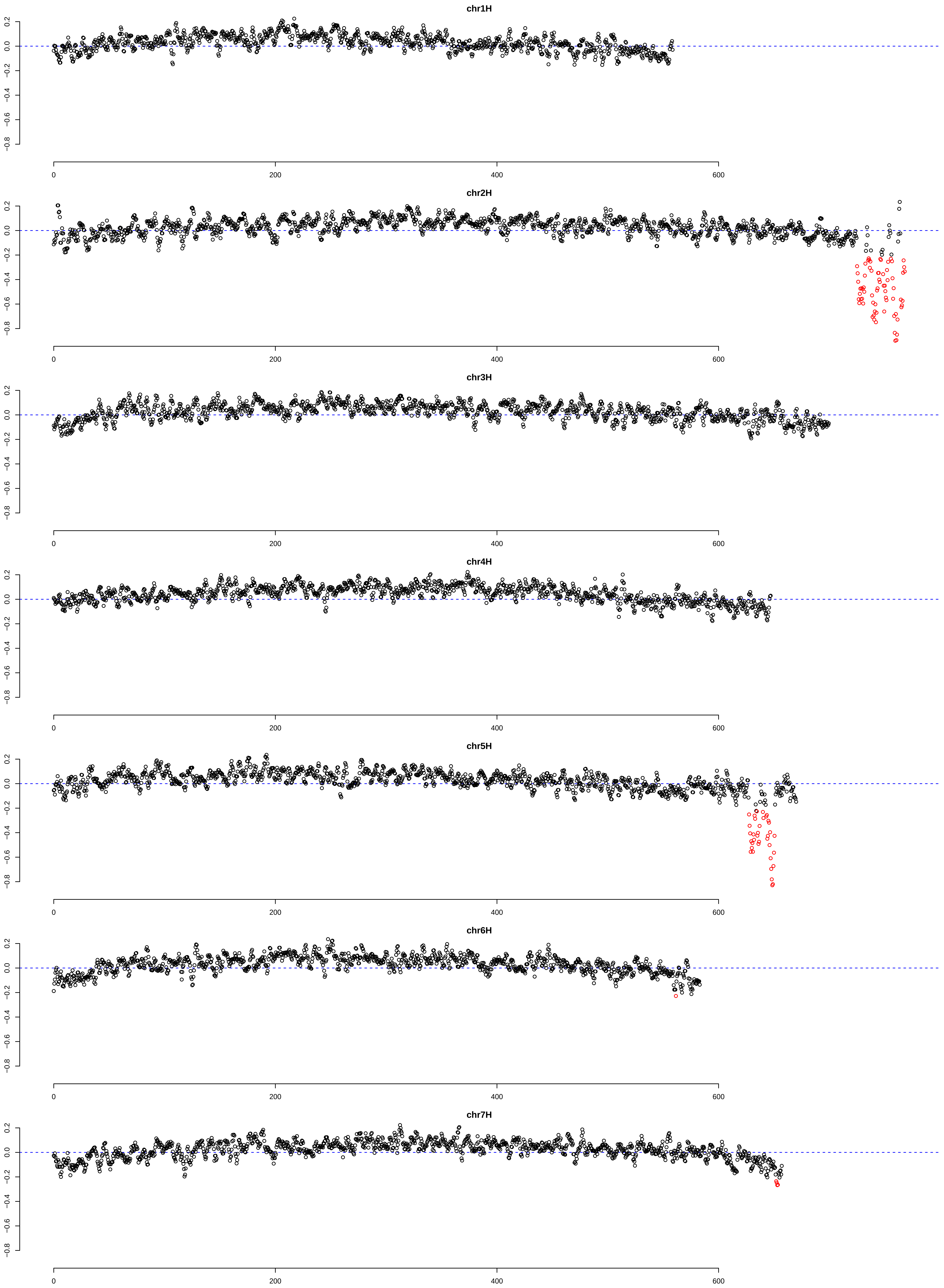
chr1H
chr2H
chr3H
chr4H
chr5H
chr6H
chr7H
0.2
0.0
-0.2
-0.4
-0.6
-0.8
0
200
400
600

ERR699836

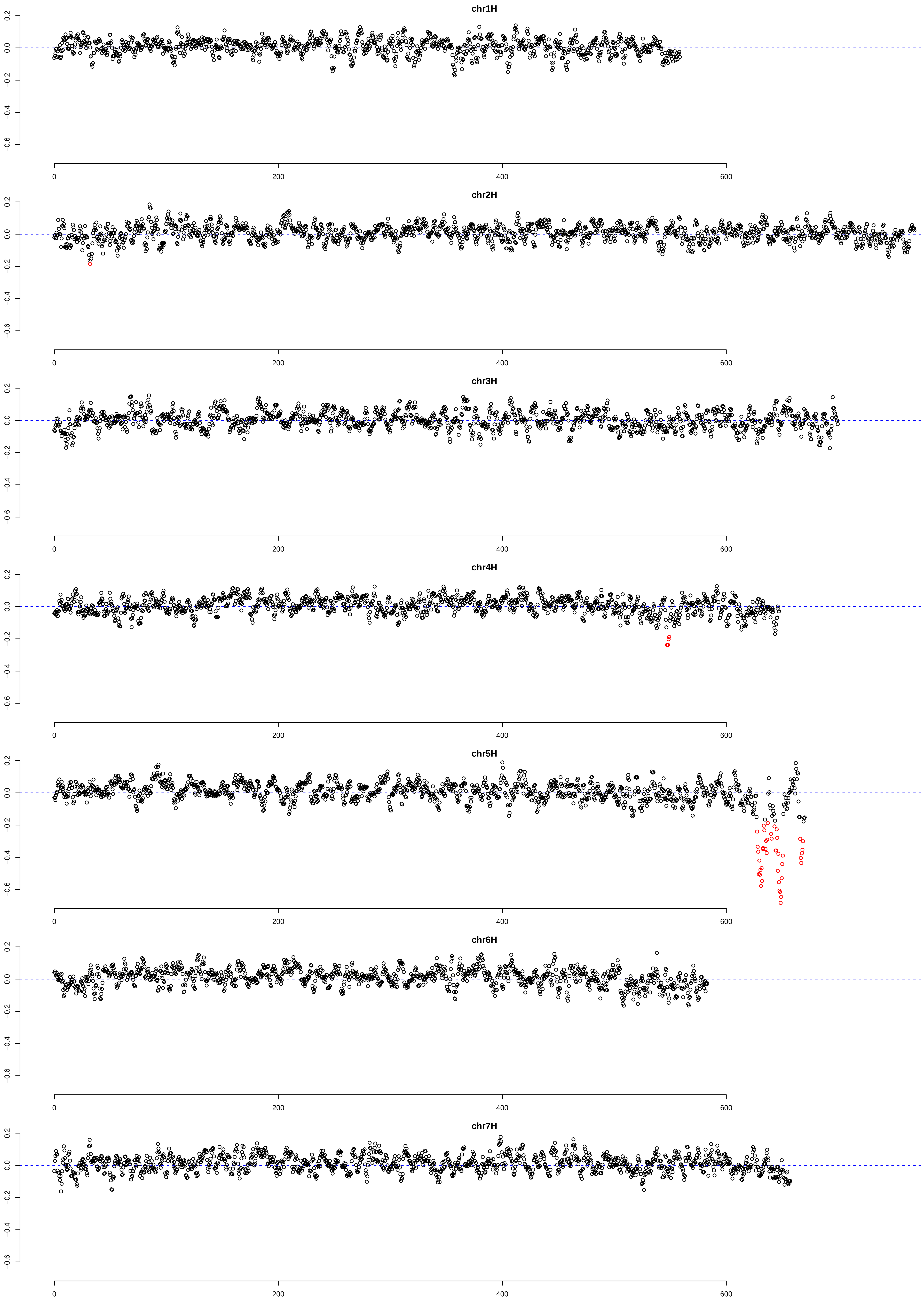
chr1H
chr2H
chr3H
chr4H
chr5H
chr6H
chr7H
0.2
0.0
-0.2
-0.4
-0.6
0
200
400
600

ERR699837

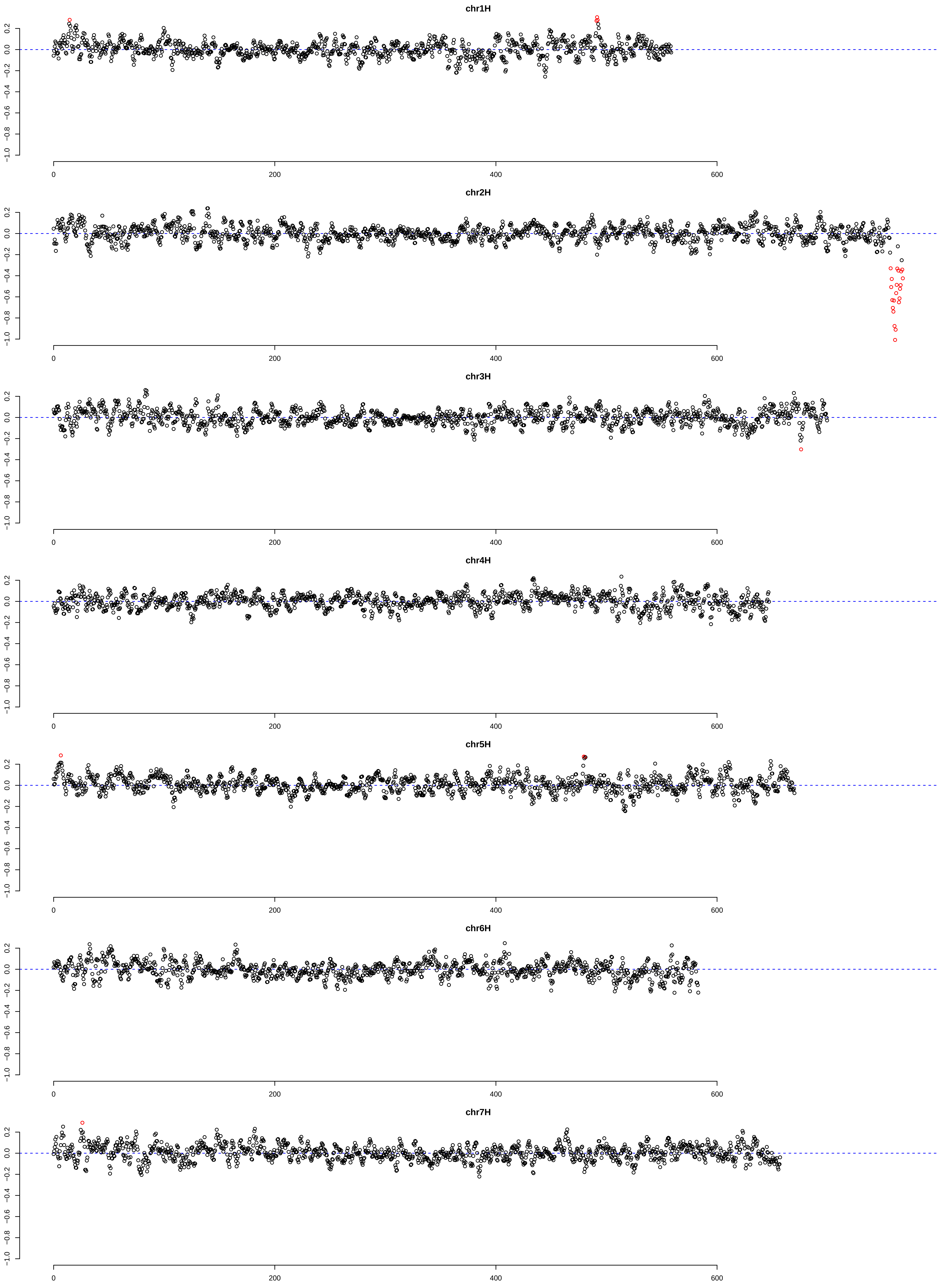
chr1H
chr2H
chr3H
chr4H
chr5H
chr6H
chr7H
0.2
0.0
−0.2
−0.4
−0.6
−0.8
−1.0
0
200
400
600

ERR699838

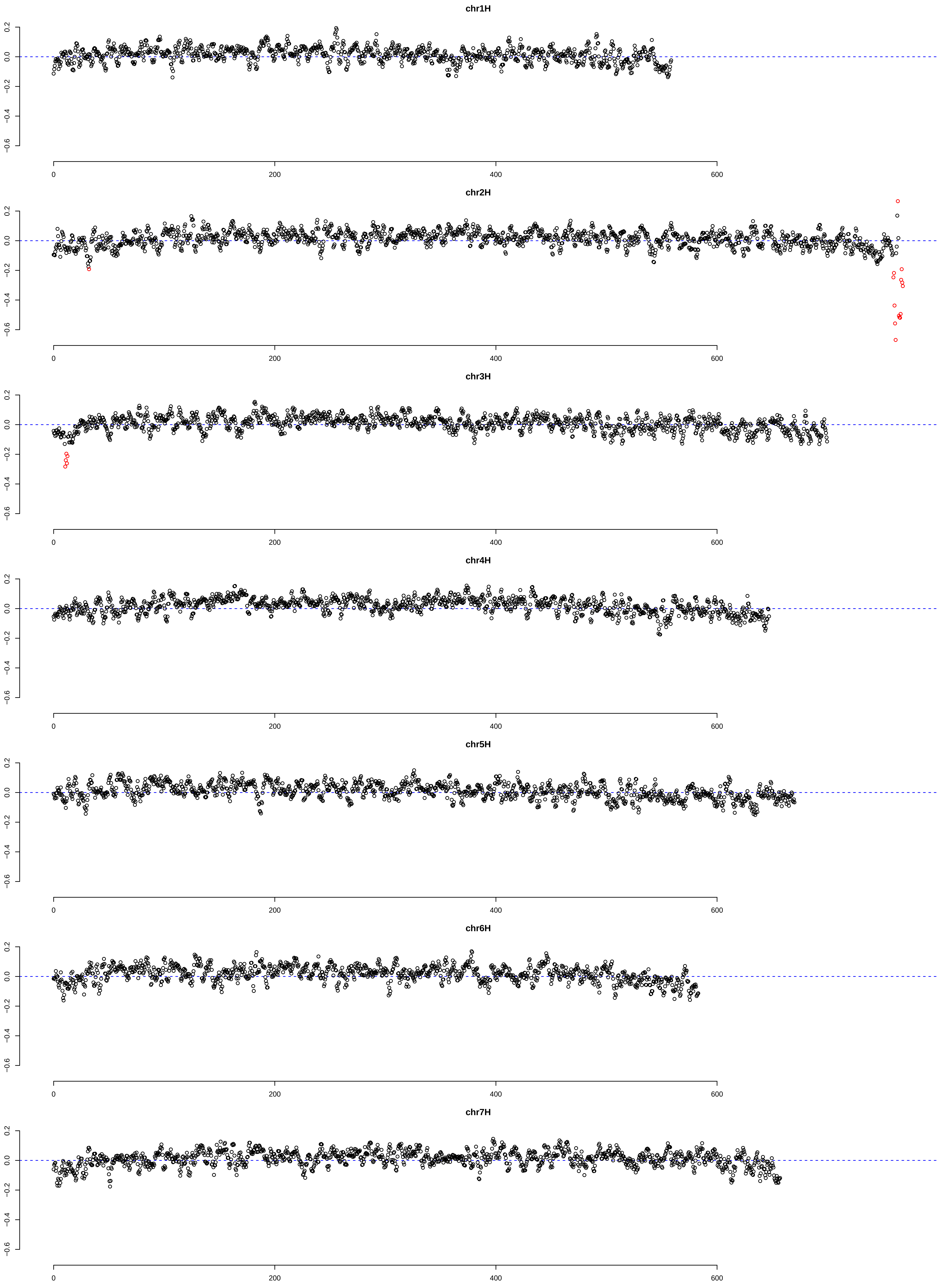

chr1H
chr2H
chr3H
chr4H
chr5H
chr6H
chr7H
0.2
0.0
-0.2
-0.4
-0.6
0
200
400
600

ERR699839

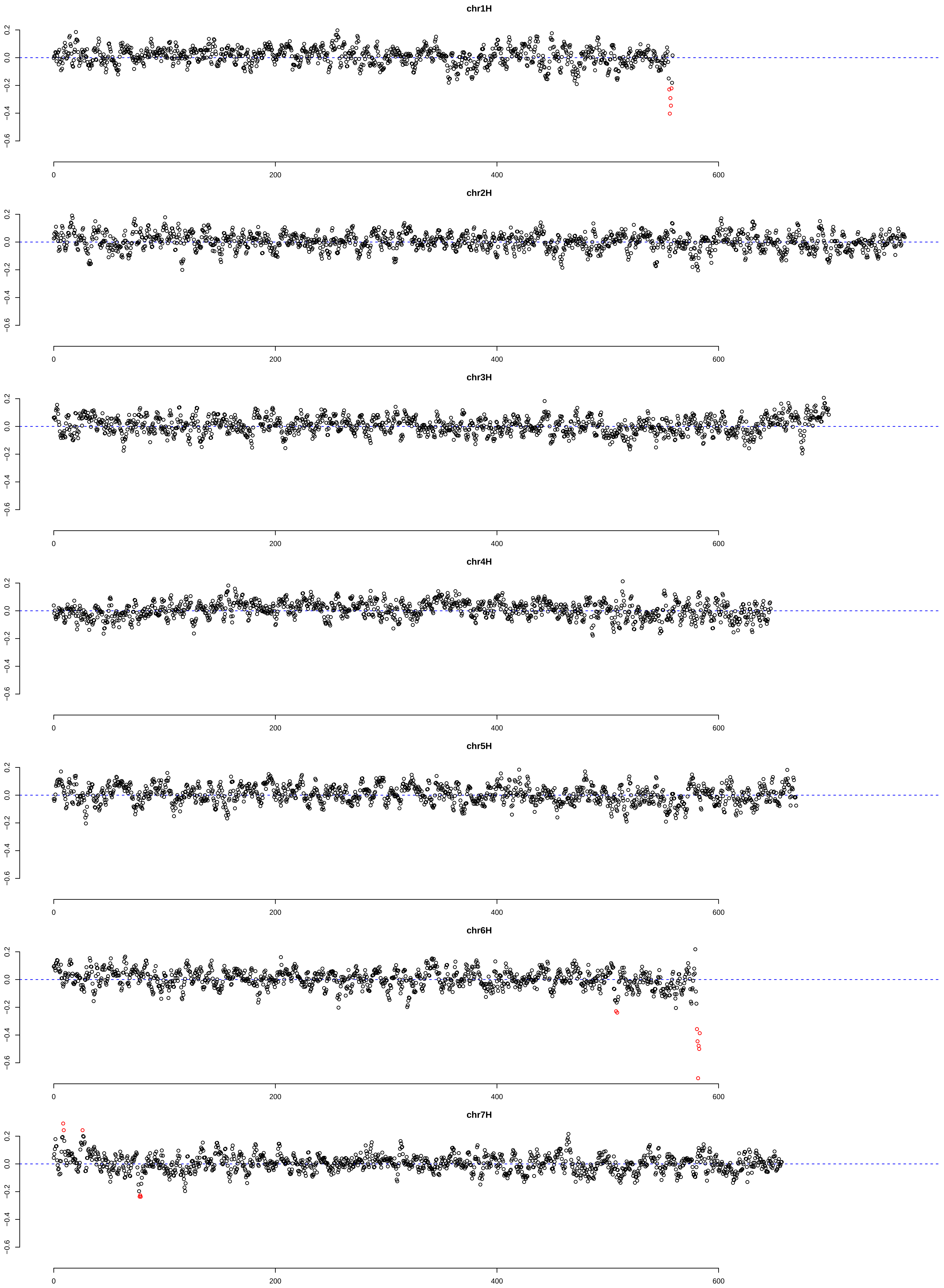

chr1H
0.2
0.0
-0.2
-0.4
-0.6
0
200
400
600
chr2H
0.2
0.0
-0.2
-0.4
-0.6
0
200
400
600
chr3H
0.2
0.0
-0.2
-0.4
-0.6
0
200
400
600
chr4H
0.2
0.0
-0.2
-0.4
-0.6
0
200
400
600
chr5H
0.2
0.0
-0.2
-0.4
-0.6
0
200
400
600
chr6H
0.2
0.0
-0.2
-0.4
-0.6
0
200
400
600
chr7H
0.2
0.0
-0.2
-0.4
-0.6
0
200
400
600

ERR699840

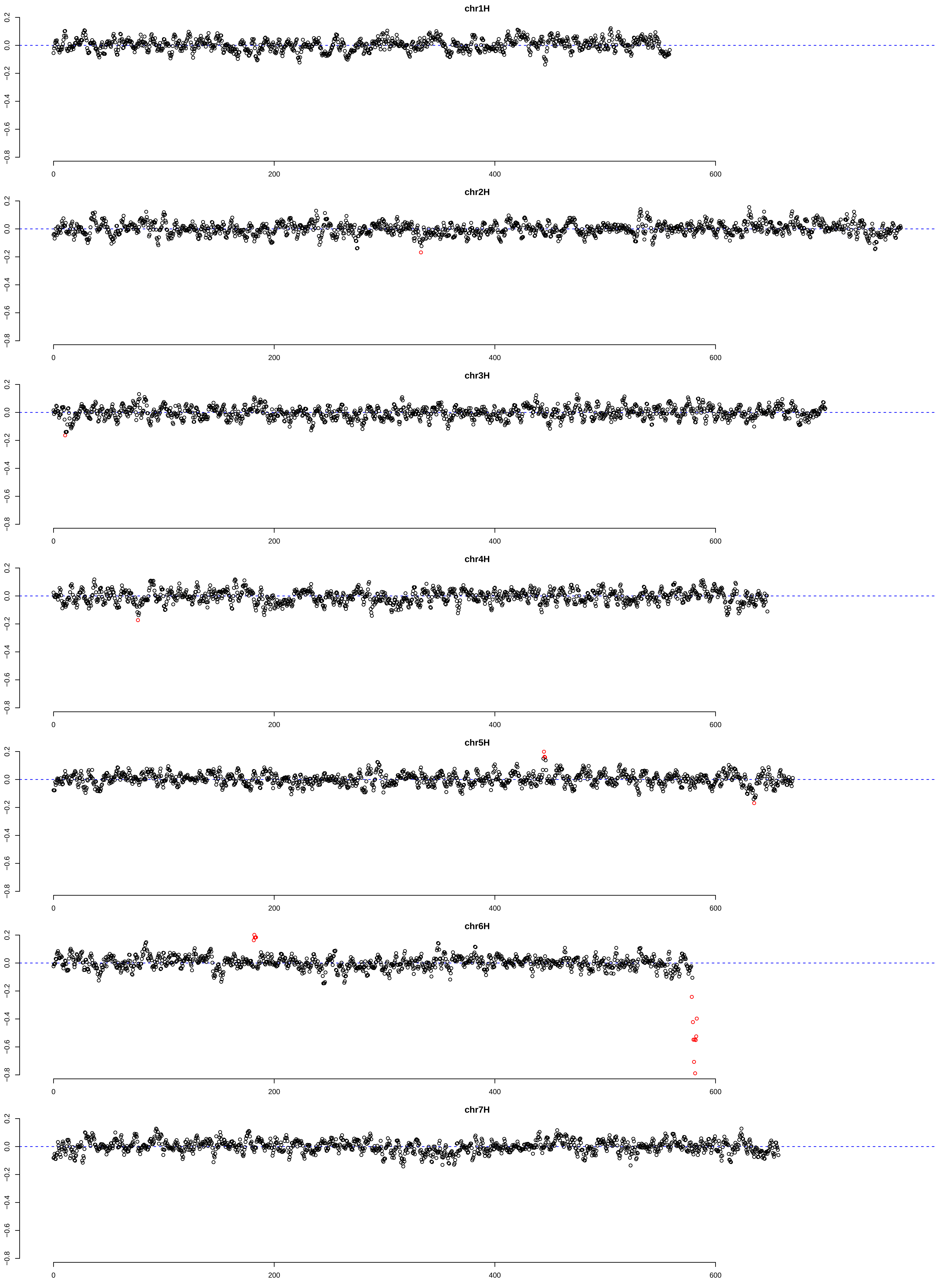

chr1H
chr2H
chr3H
chr4H
chr5H
chr6H
chr7H
0.2
0.0
-0.2
-0.4
-0.6
-0.8
0
200
400
600

ERR699841

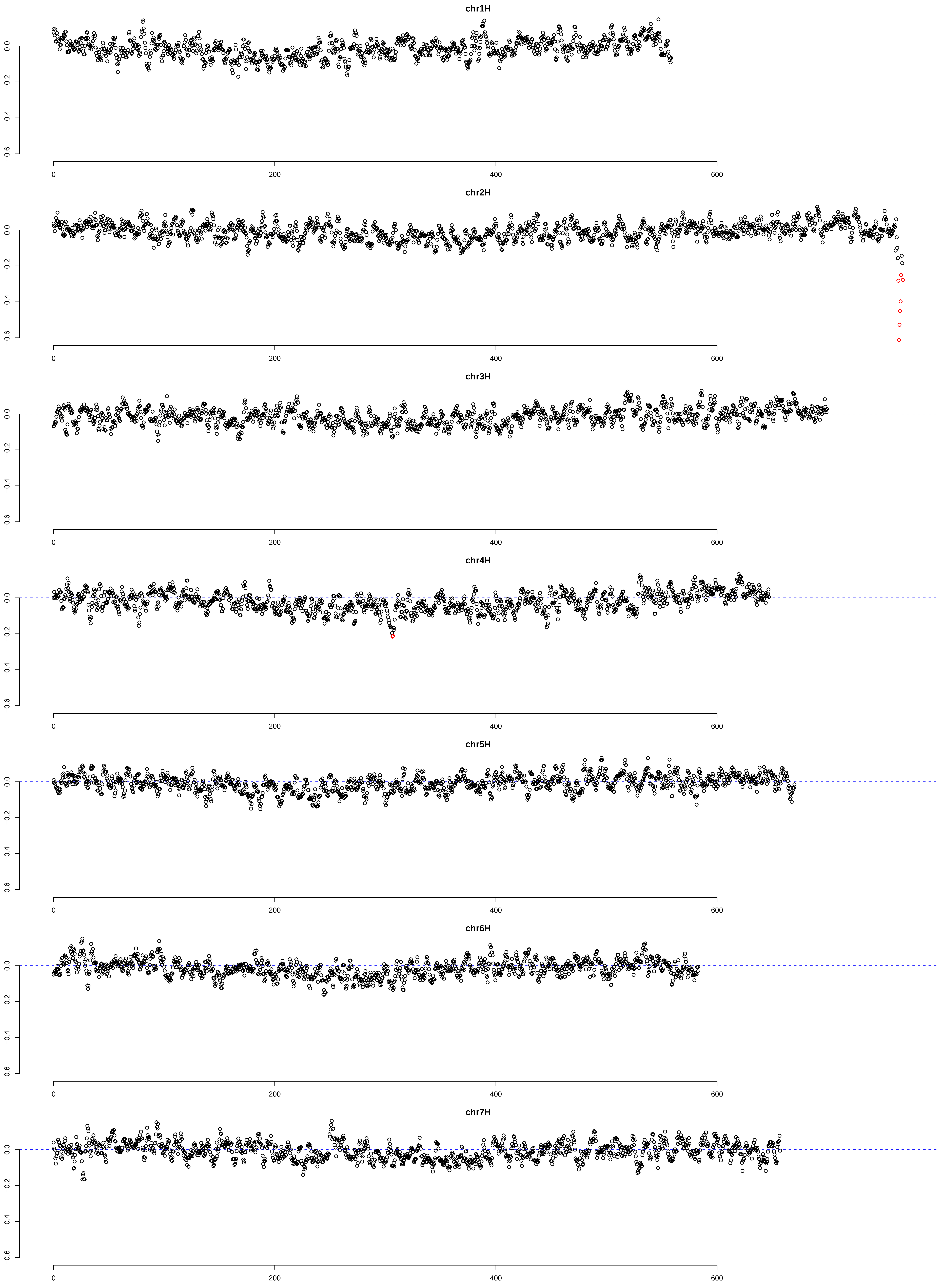

chr1H
0.0
-0.2
-0.4
-0.6
0
200
400
600
chr2H
0.0
-0.2
-0.4
-0.6
0
200
400
600
chr3H
0.0
-0.2
-0.4
-0.6
0
200
400
600
chr4H
0.0
-0.2
-0.4
-0.6
0
200
400
600
chr5H
0.0
-0.2
-0.4
-0.6
0
200
400
600
chr6H
0.0
-0.2
-0.4
-0.6
0
200
400
600
chr7H
0.0
-0.2
-0.4
-0.6
0
200
400
600

ERR699842

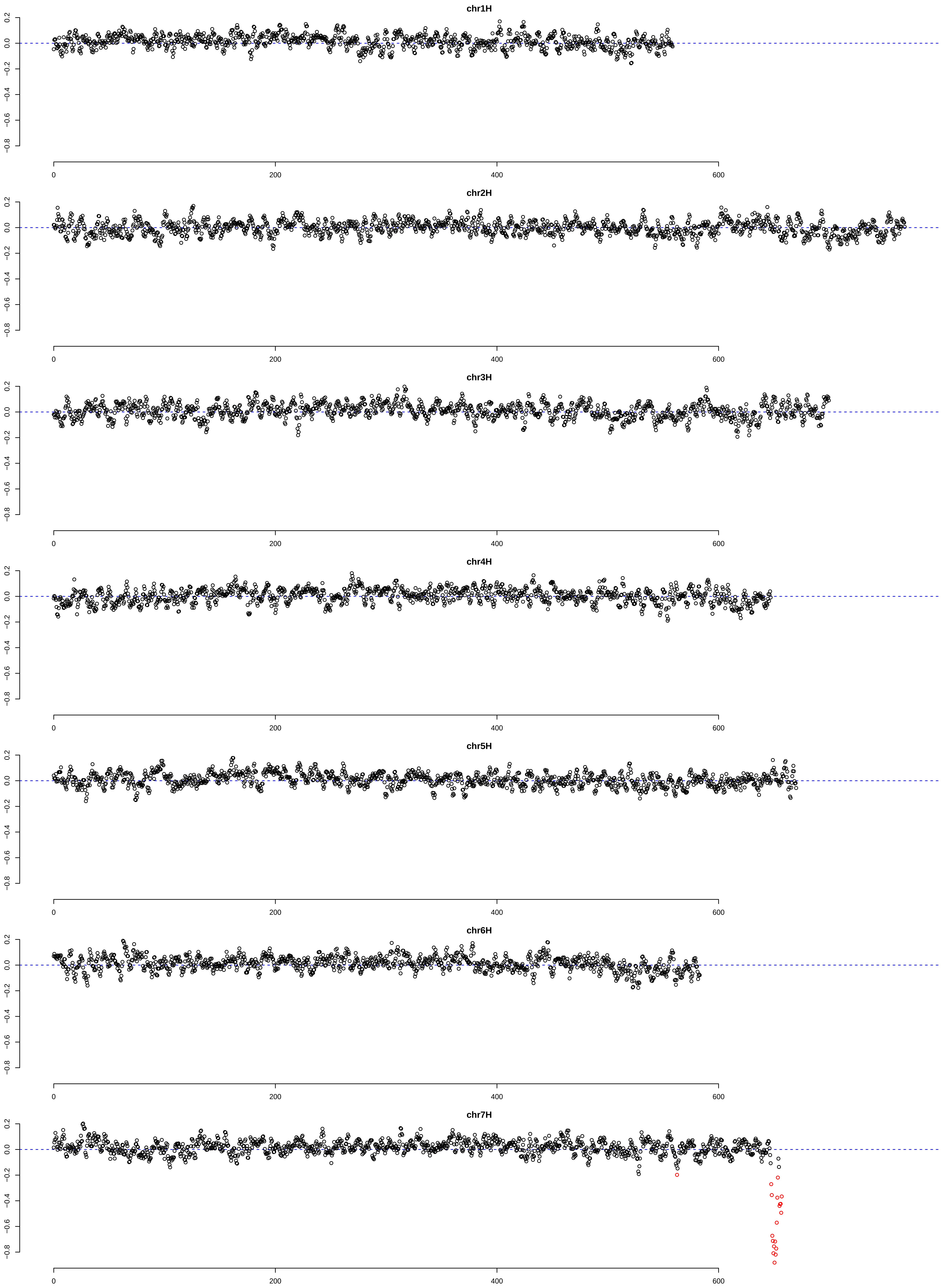
chr1H
chr2H
chr3H
chr4H
chr5H
chr6H
chr7H
0.2
0.0
-0.2
-0.4
-0.6
-0.8
0
200
400
600

ERR699843

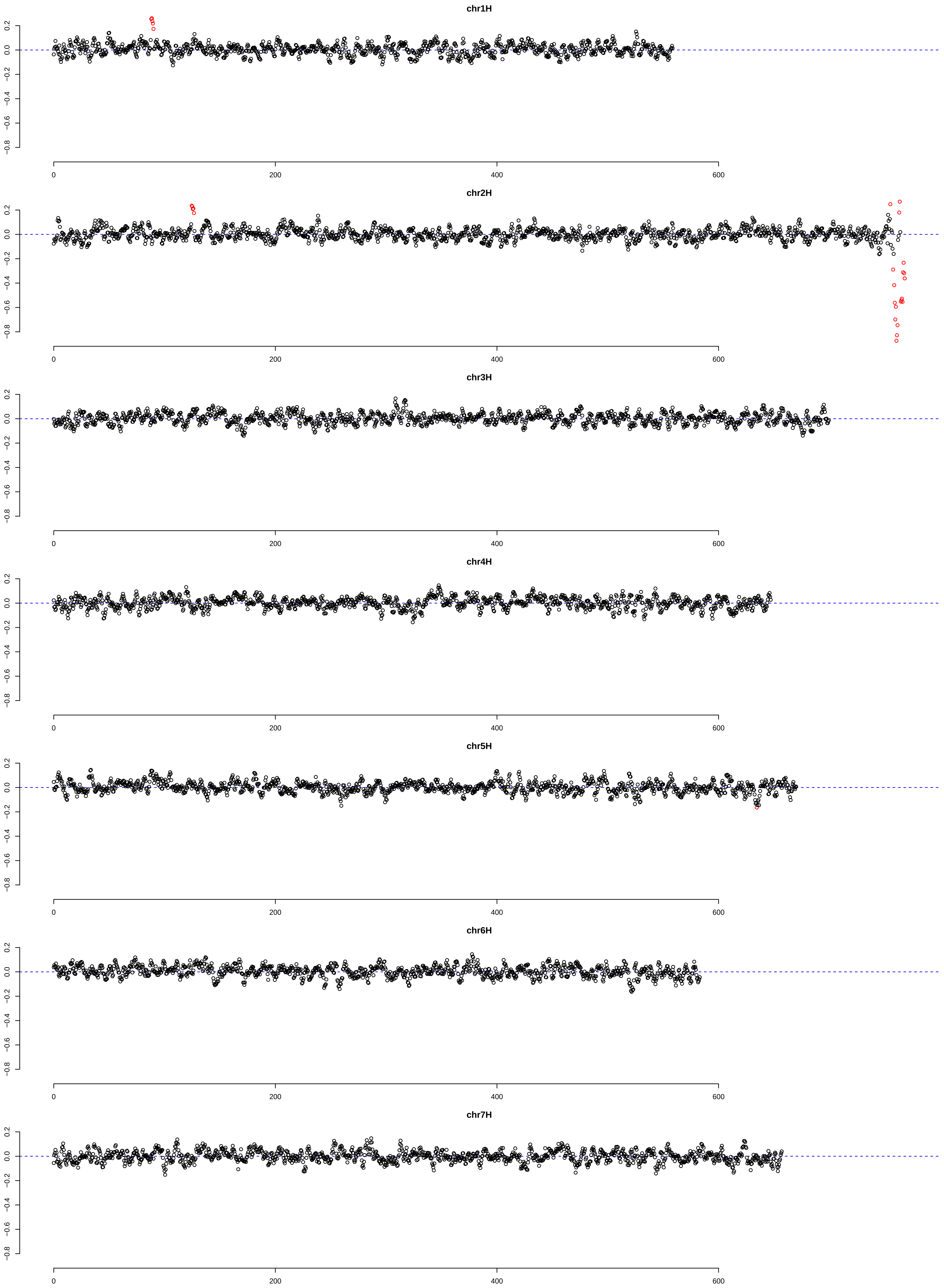

chr1H
chr2H
chr3H
chr4H
chr5H
chr6H
chr7H
0.2
0.0
-0.2
-0.4
-0.6
-0.8
0
200
400
600

ERR699844

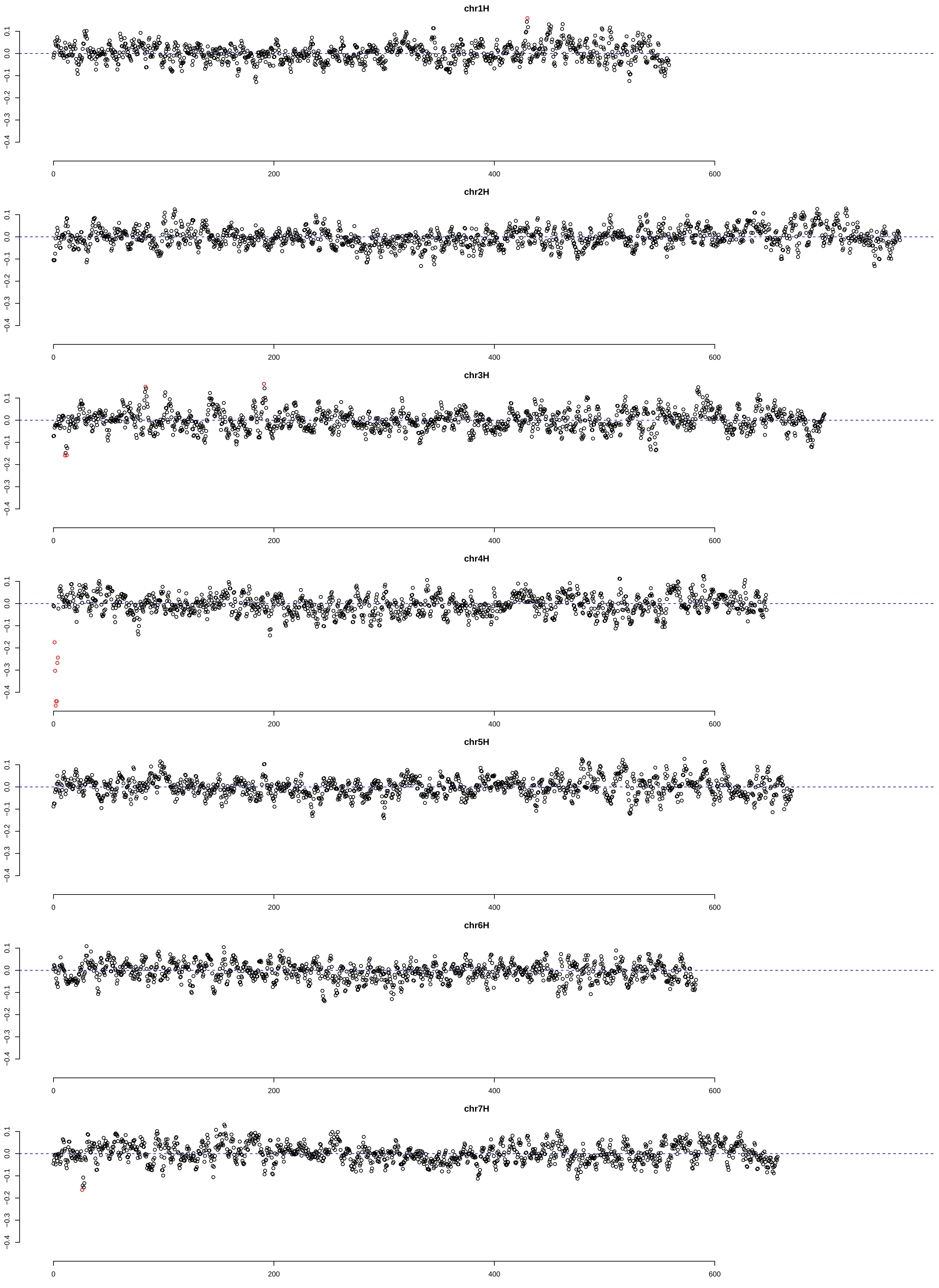

chr1H
chr2H
chr3H
chr4H
chr5H
chr6H
chr7H
0.1
0.0
-0.1
-0.2
-0.3
-0.4
0
200
400
600

ERR699845

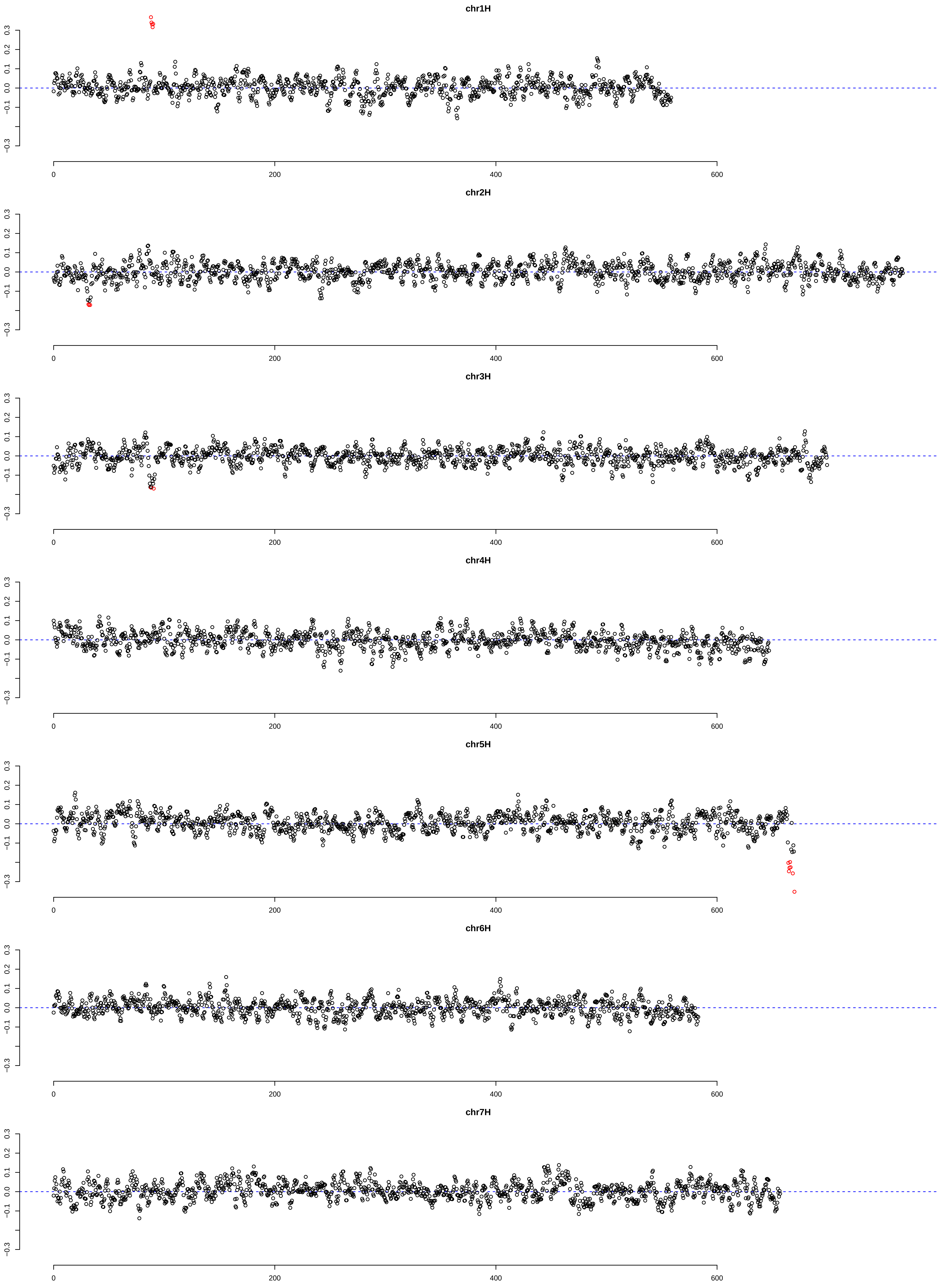
chr1H
chr2H
chr3H
chr4H
chr5H
chr6H
chr7H
0.3
0.2
0.1
0.0
-0.1
-0.3
0
200
400
600

ERR699846

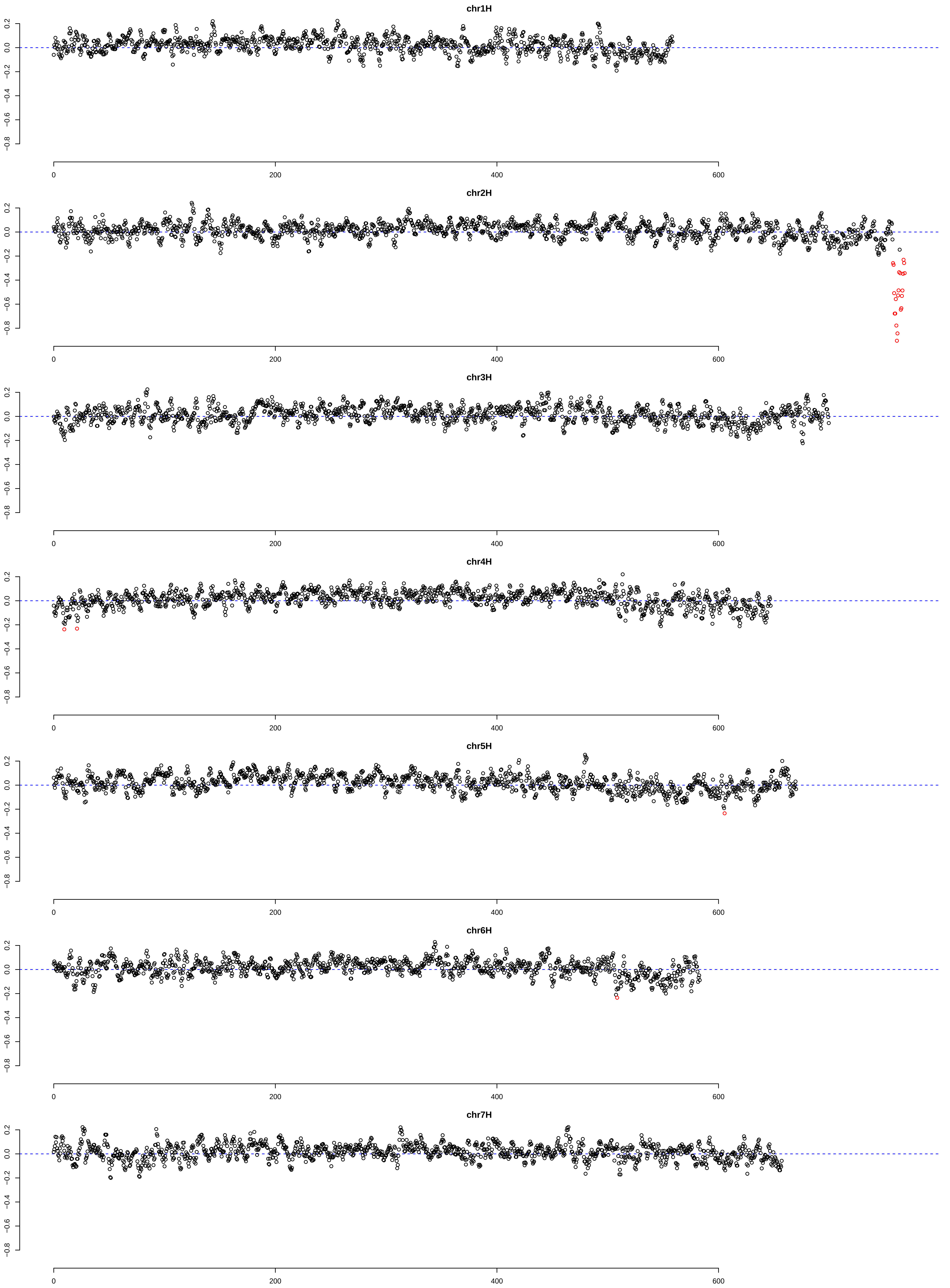

chr1H
chr2H
chr3H
chr4H
chr5H
chr6H
chr7H
0.2
0.0
-0.2
-0.4
-0.6
-0.8
0
200
400
600

ERR699847

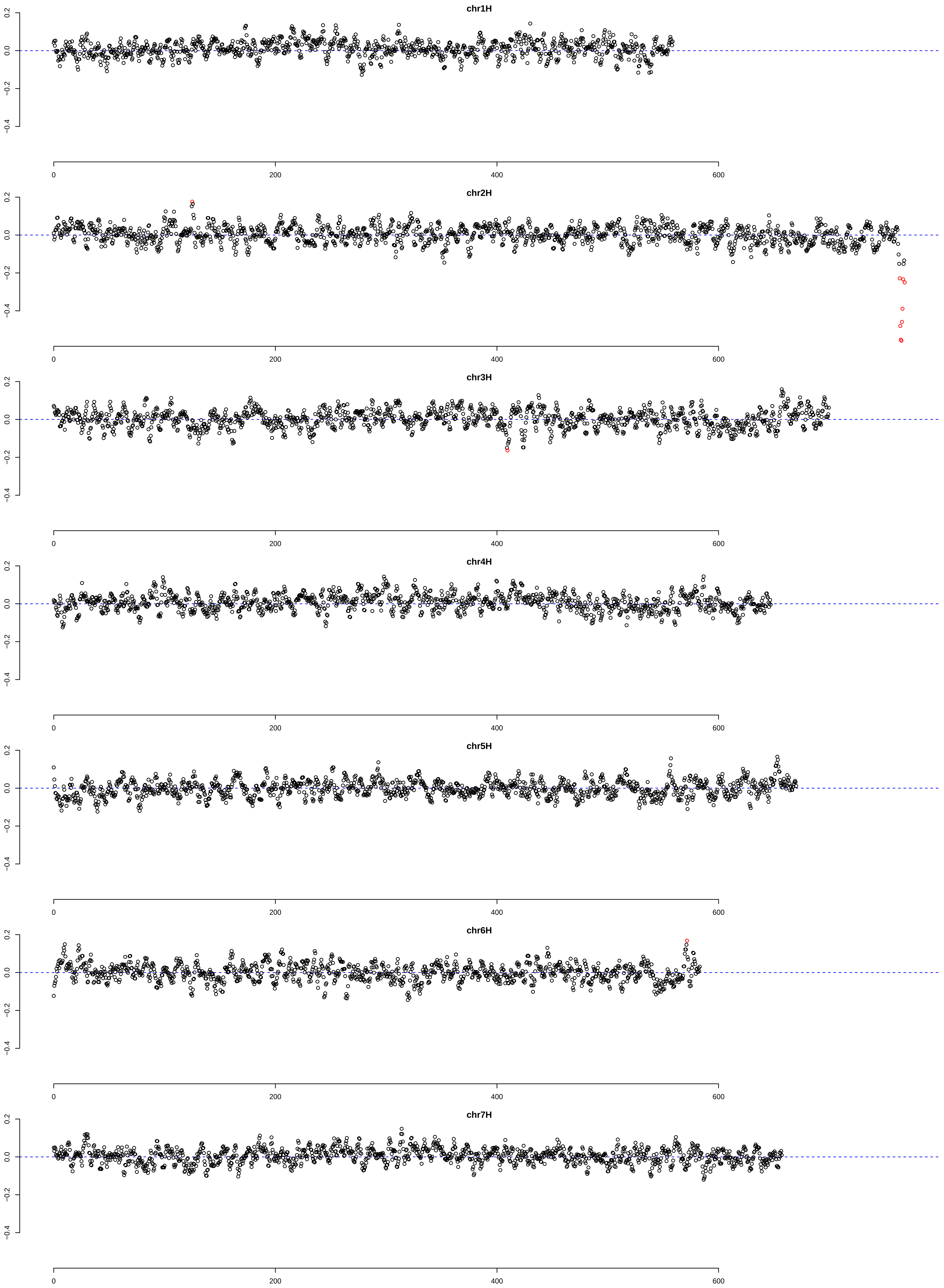
chr1H
chr2H
chr3H
chr4H
chr5H
chr6H
chr7H
0.2
0.0
-0.2
-0.4
0
200
400
600

ERR699848

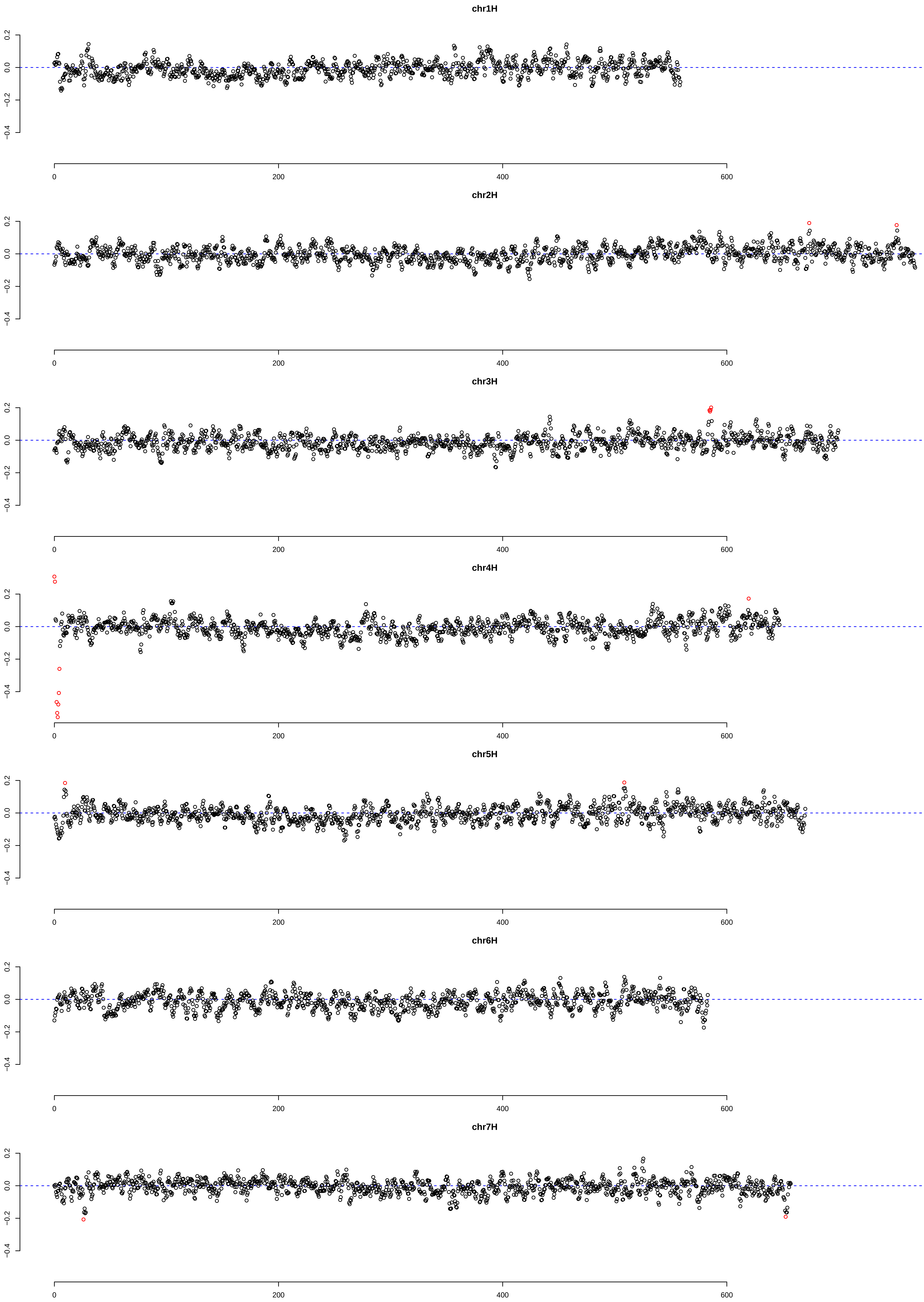

chr1H
chr2H
chr3H
chr4H
chr5H
chr6H
chr7H
0.2
0.0
-0.2
-0.4
0
200
400
600

ERR699849

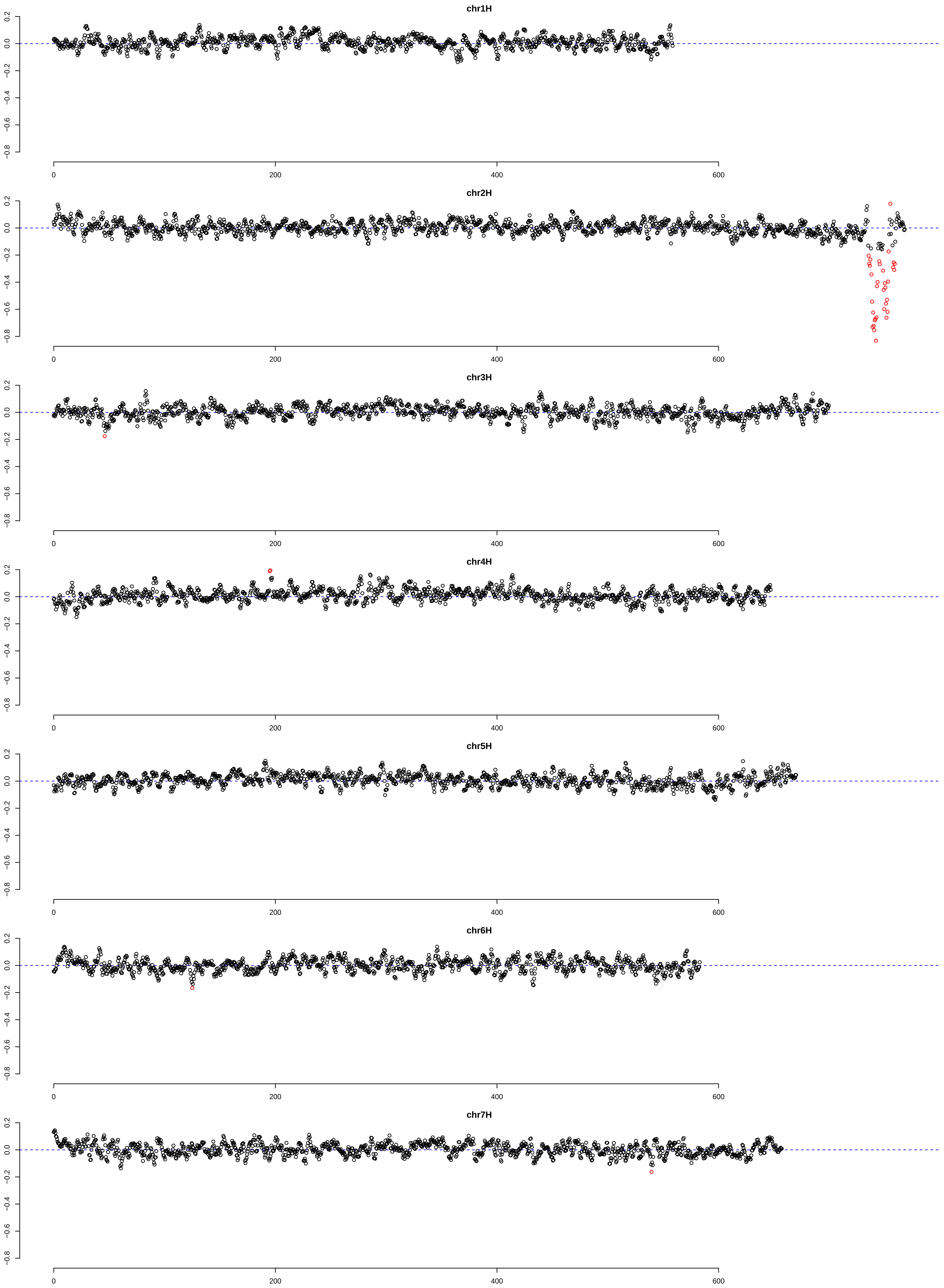
chr1H
chr2H
chr3H
chr4H
chr5H
chr6H
chr7H
0.2
0.0
-0.2
-0.4
-0.6
-0.8
0
200
400
600

ERR699850

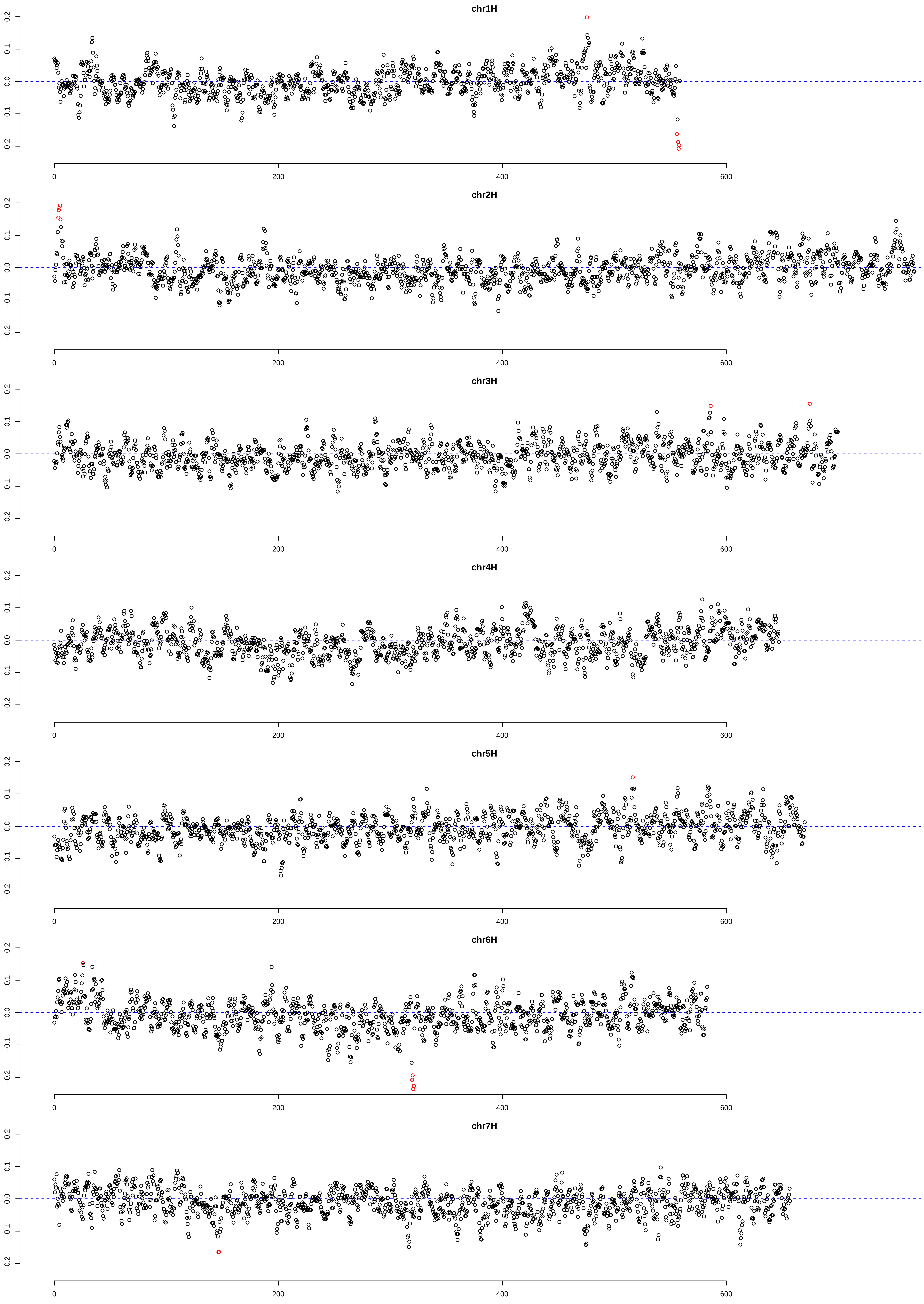
chr1H
chr2H
chr3H
chr4H
chr5H
chr6H
chr7H
0.2
0.1
0.0
-0.1
-0.2
0
200
400
600

ERR699851

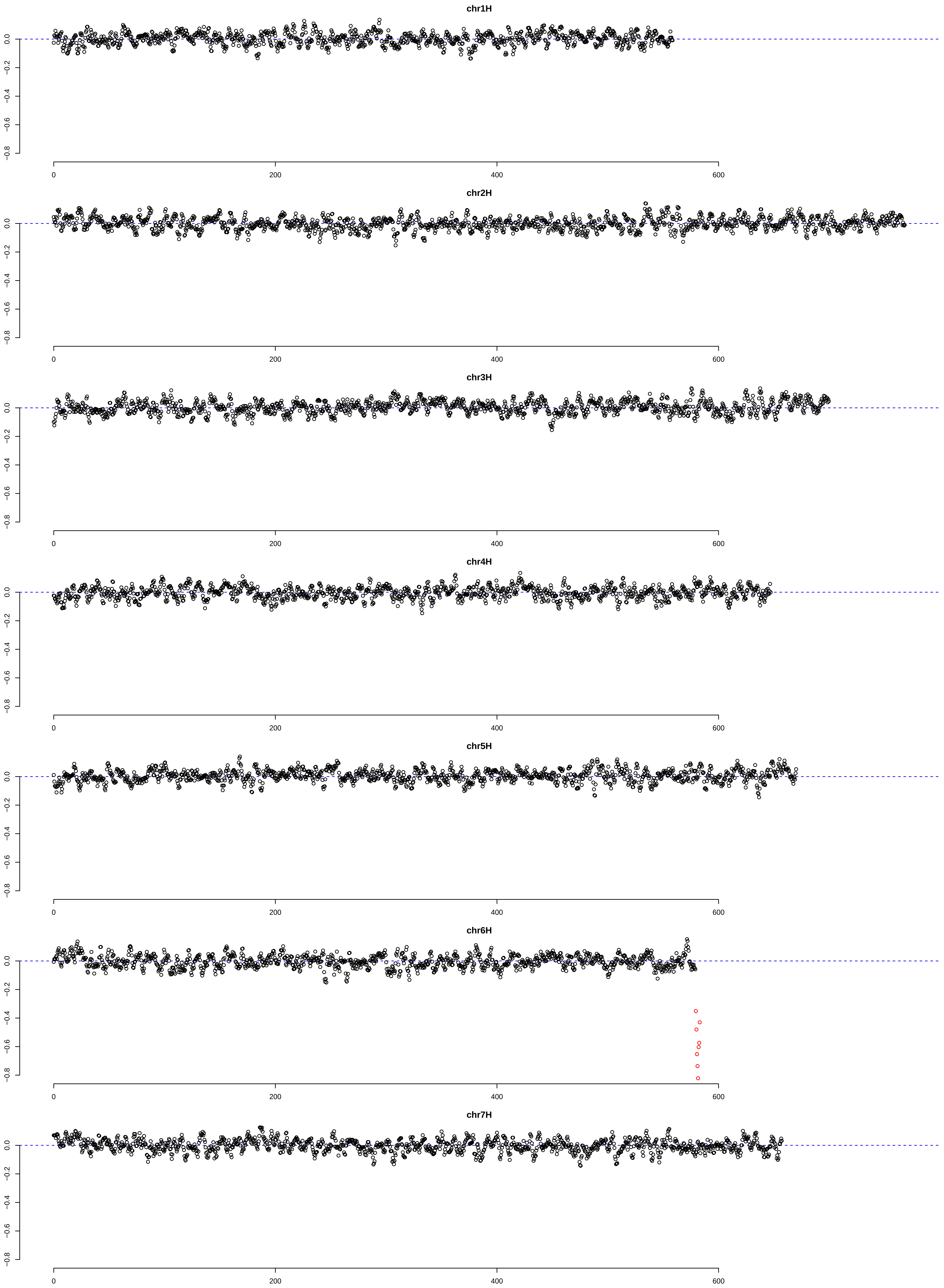
chr1H
chr2H
chr3H
chr4H
chr5H
chr6H
chr7H
0.0
-0.2
-0.4
-0.6
-0.8
0
200
400
600

ERR699852

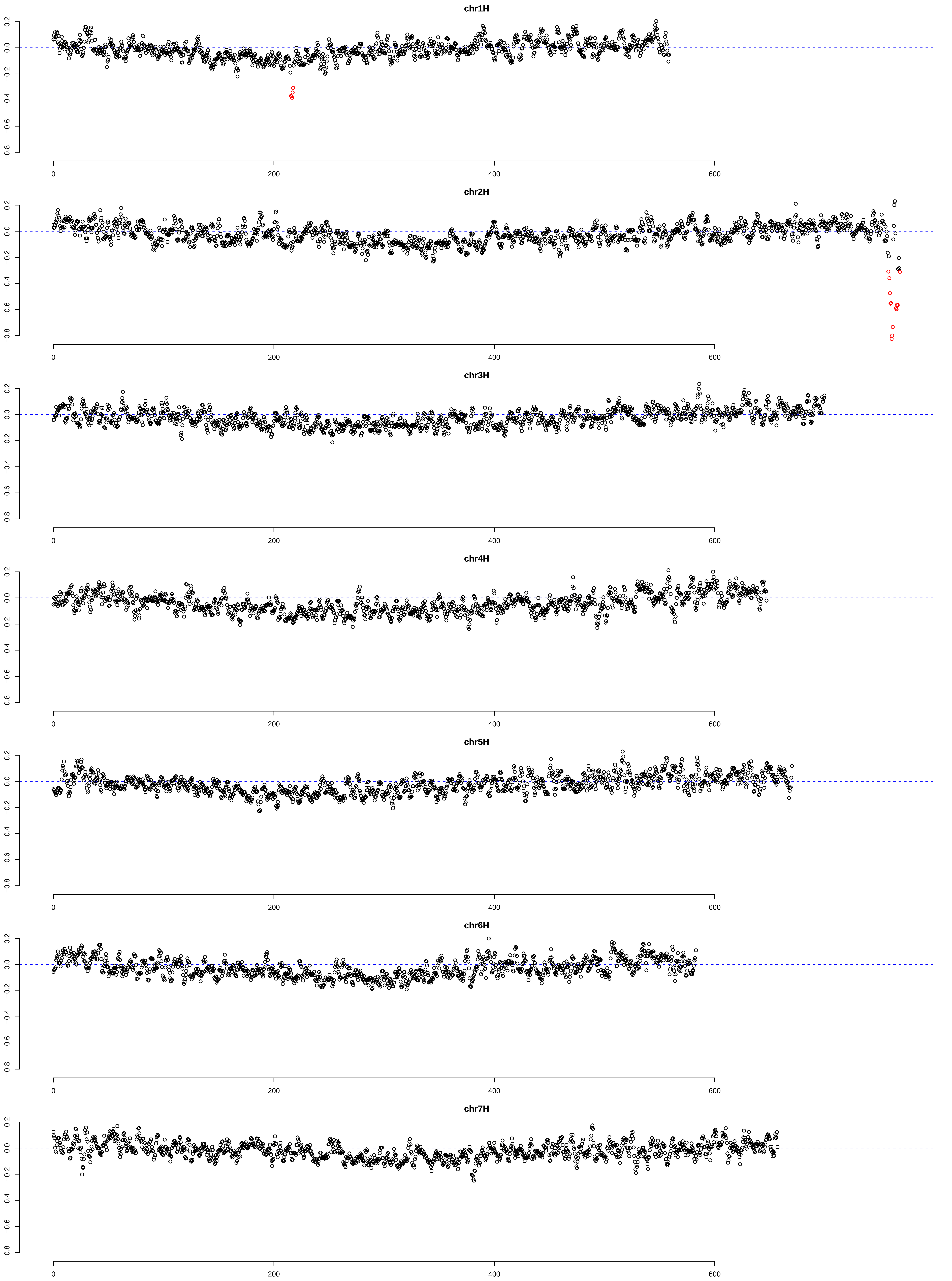

chr1H
chr2H
chr3H
chr4H
chr5H
chr6H
chr7H
0.2
0.0
−0.2
−0.4
−0.6
−0.8
0
200
400
600

ERR699853

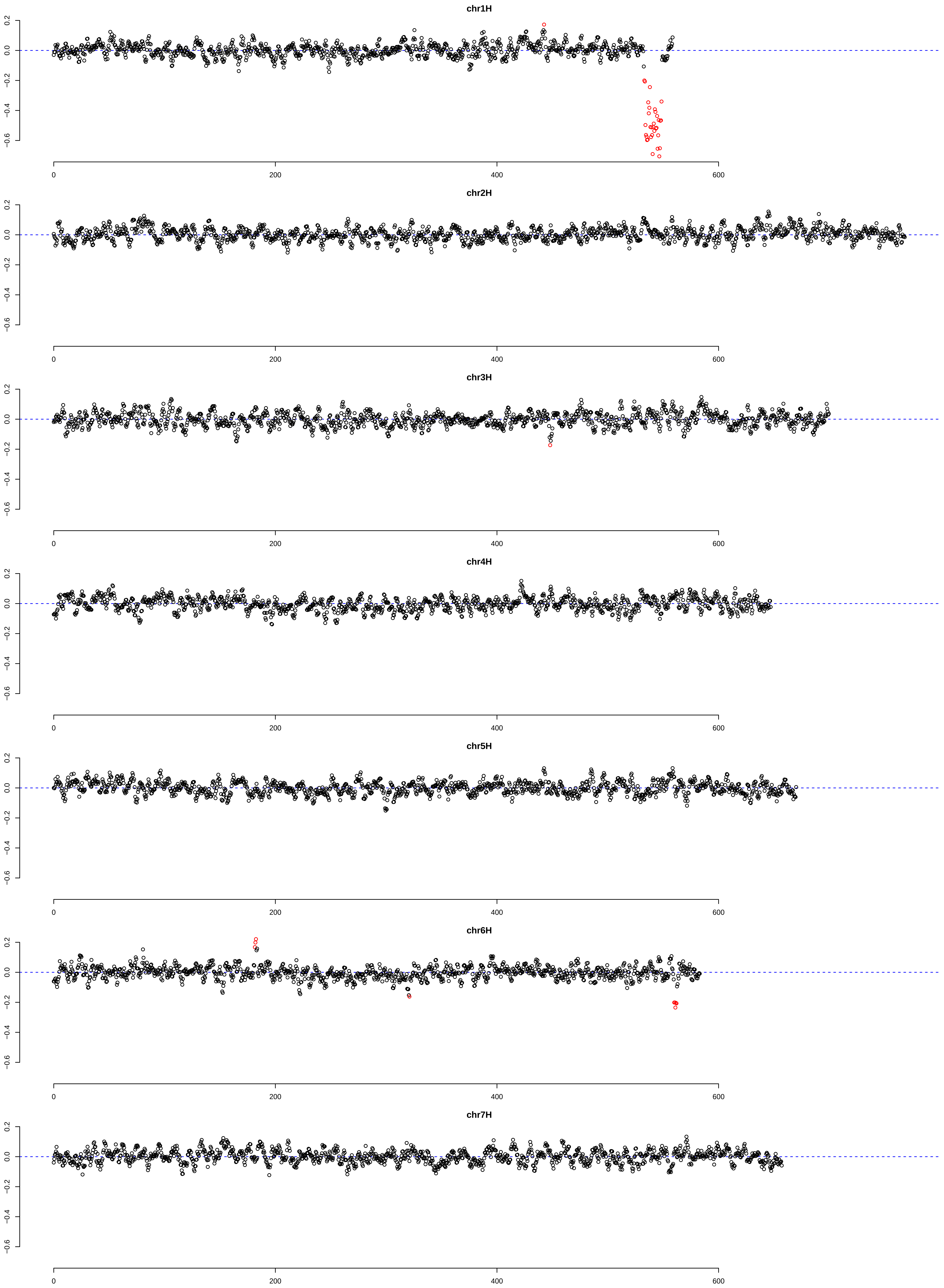

chr1H
chr2H
chr3H
chr4H
chr5H
chr6H
chr7H
0.2
0.0
-0.2
-0.4
-0.6
0
200
400
600

ERR699854

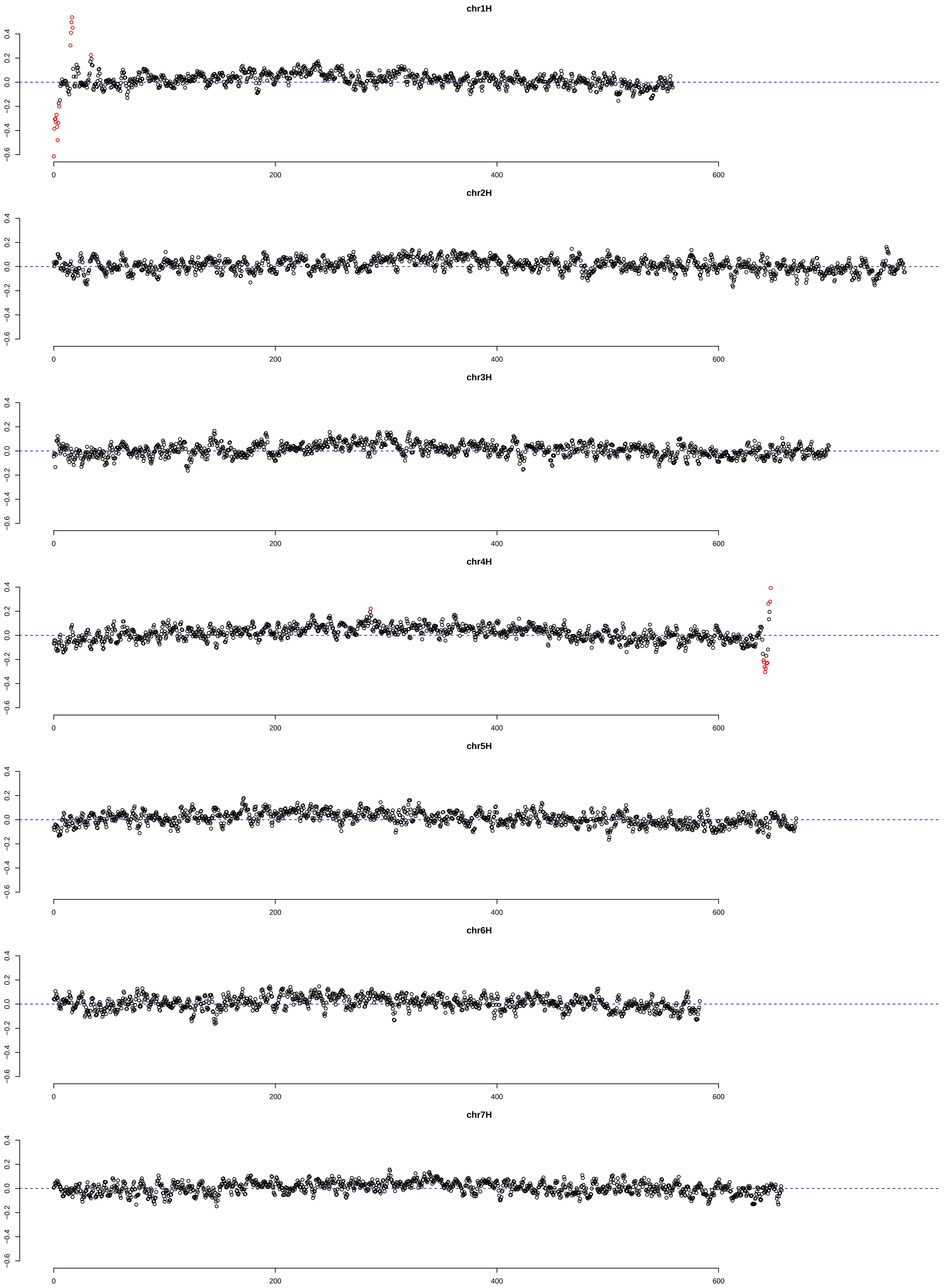
chr1H
chr2H
chr3H
chr4H
chr5H
chr6H
chr7H
0.4
0.2
0.0
-0.2
-0.4
-0.6
0
200
400
600

ERR699855

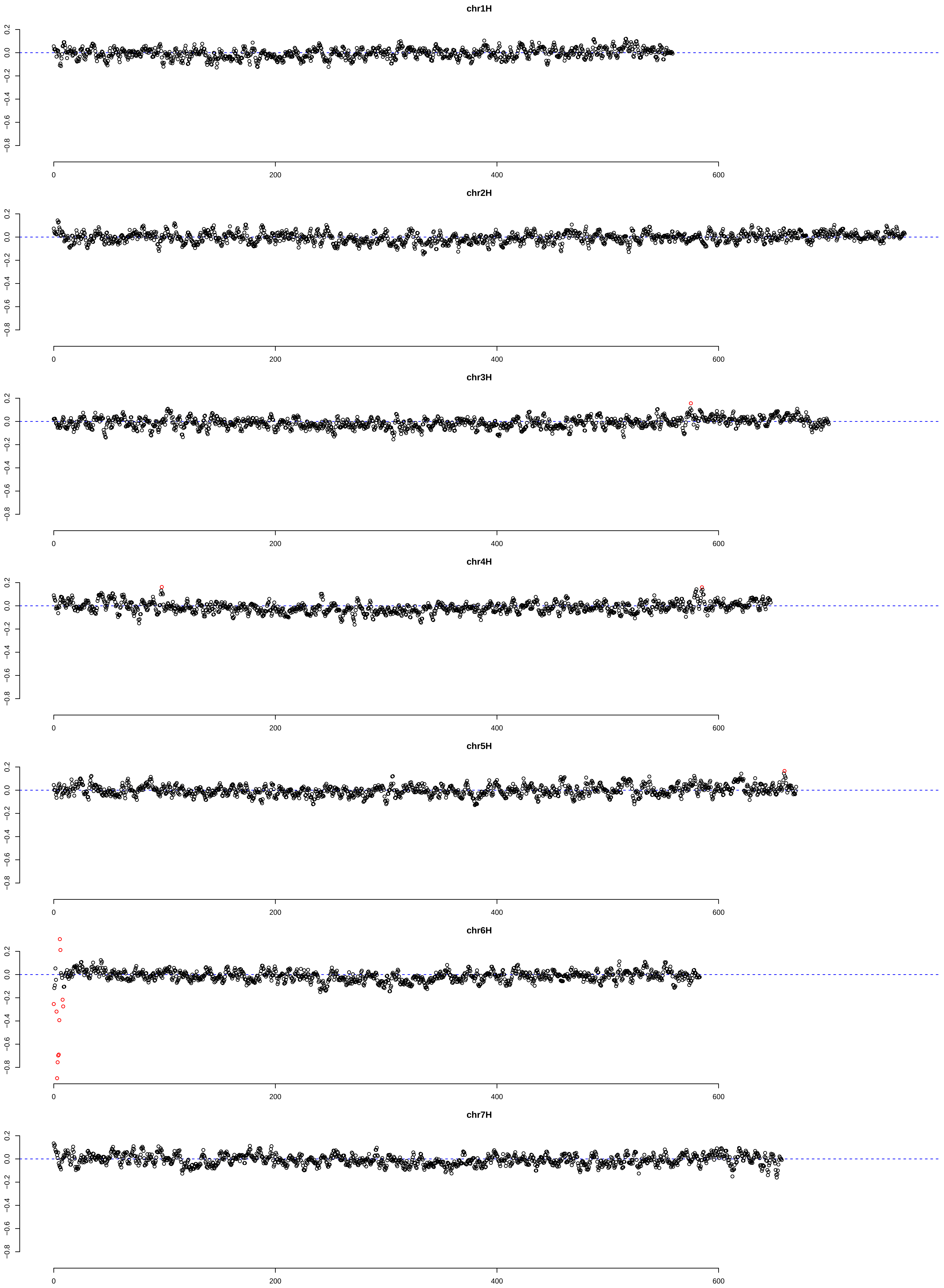

ERR699856

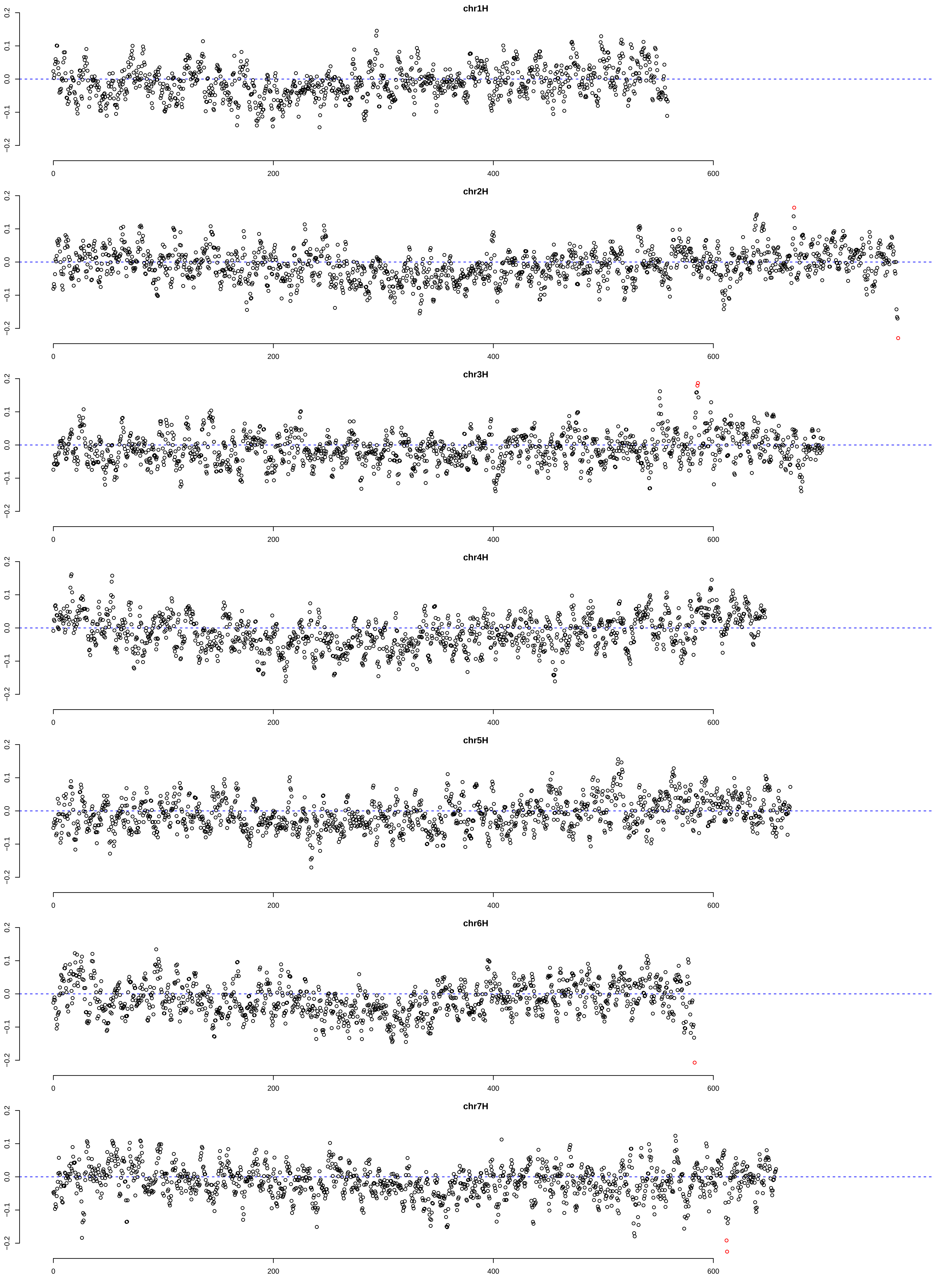
chr1H
chr2H
chr3H
chr4H
chr5H
chr6H
chr7H
0.2
0.1
0.0
-0.1
-0.2
0
200
400
600

ERR699857

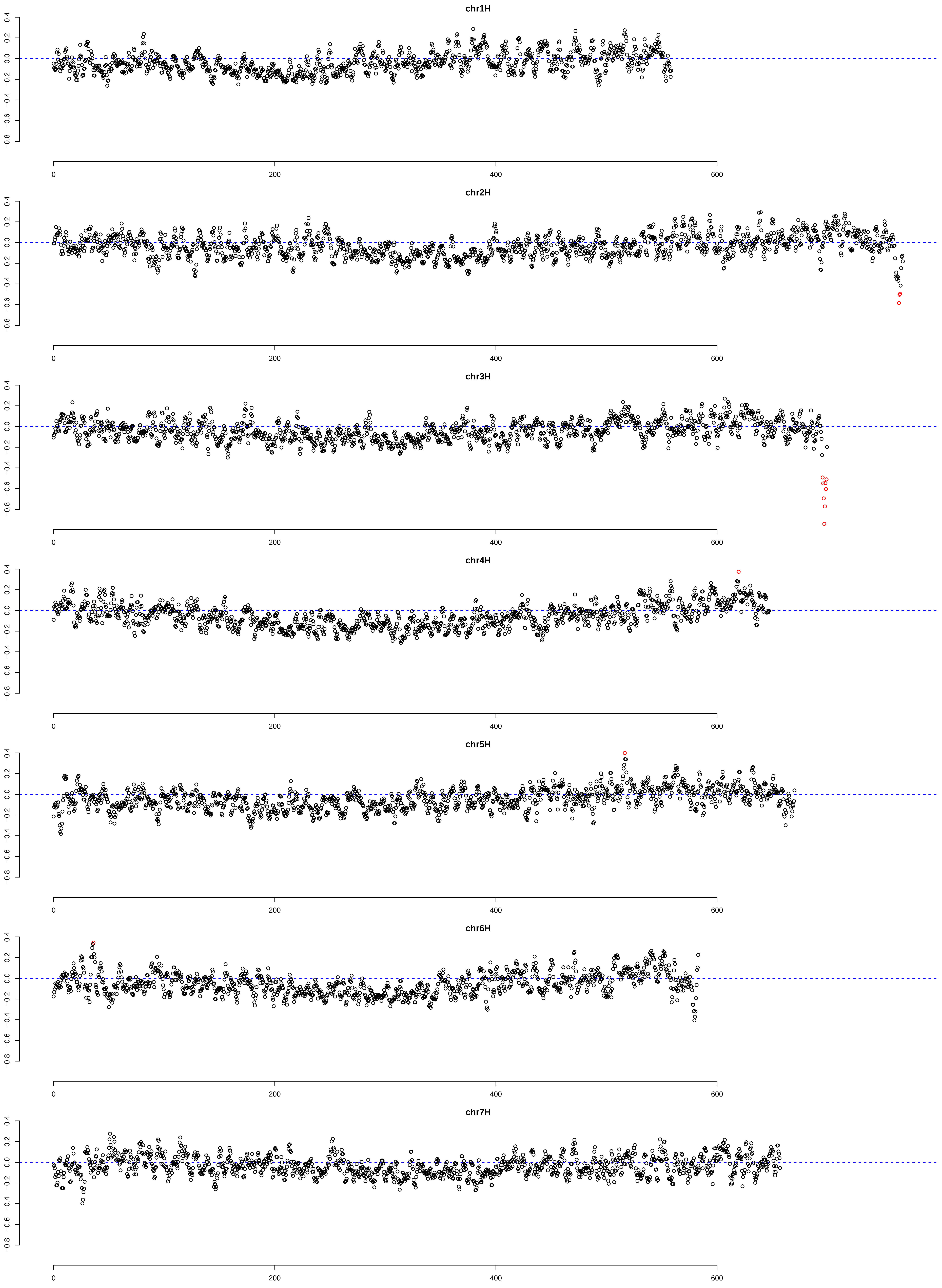
chr1H
chr2H
chr3H
chr4H
chr5H
chr6H
chr7H
0.4
0.2
0.0
-0.2
-0.4
-0.6
-0.8
0
200
400
600

ERR699858

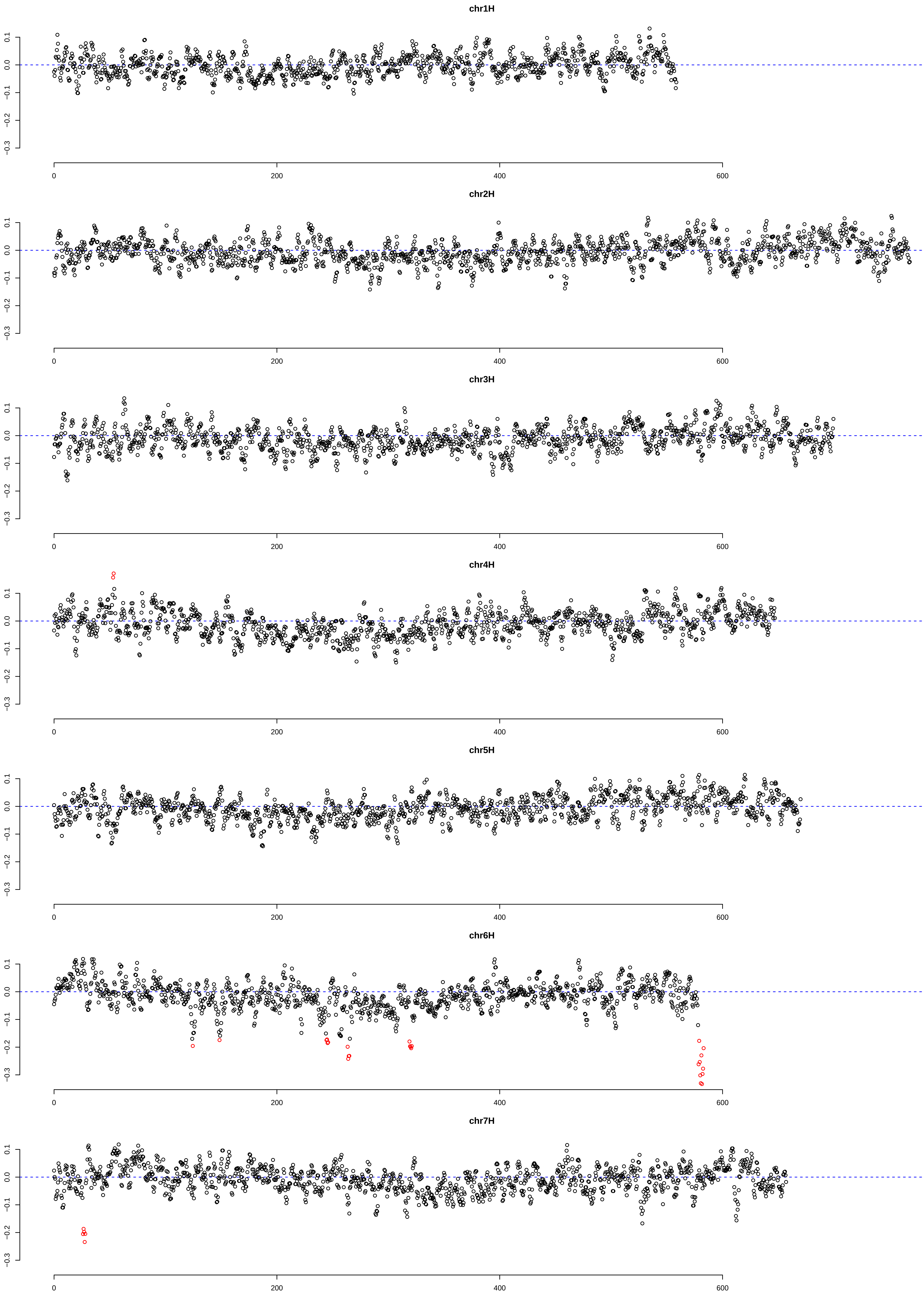

chr1H
chr2H
chr3H
chr4H
chr5H
chr6H
chr7H
0.1
0.0
-0.1
-0.2
-0.3
0
200
400
600

ERR699859

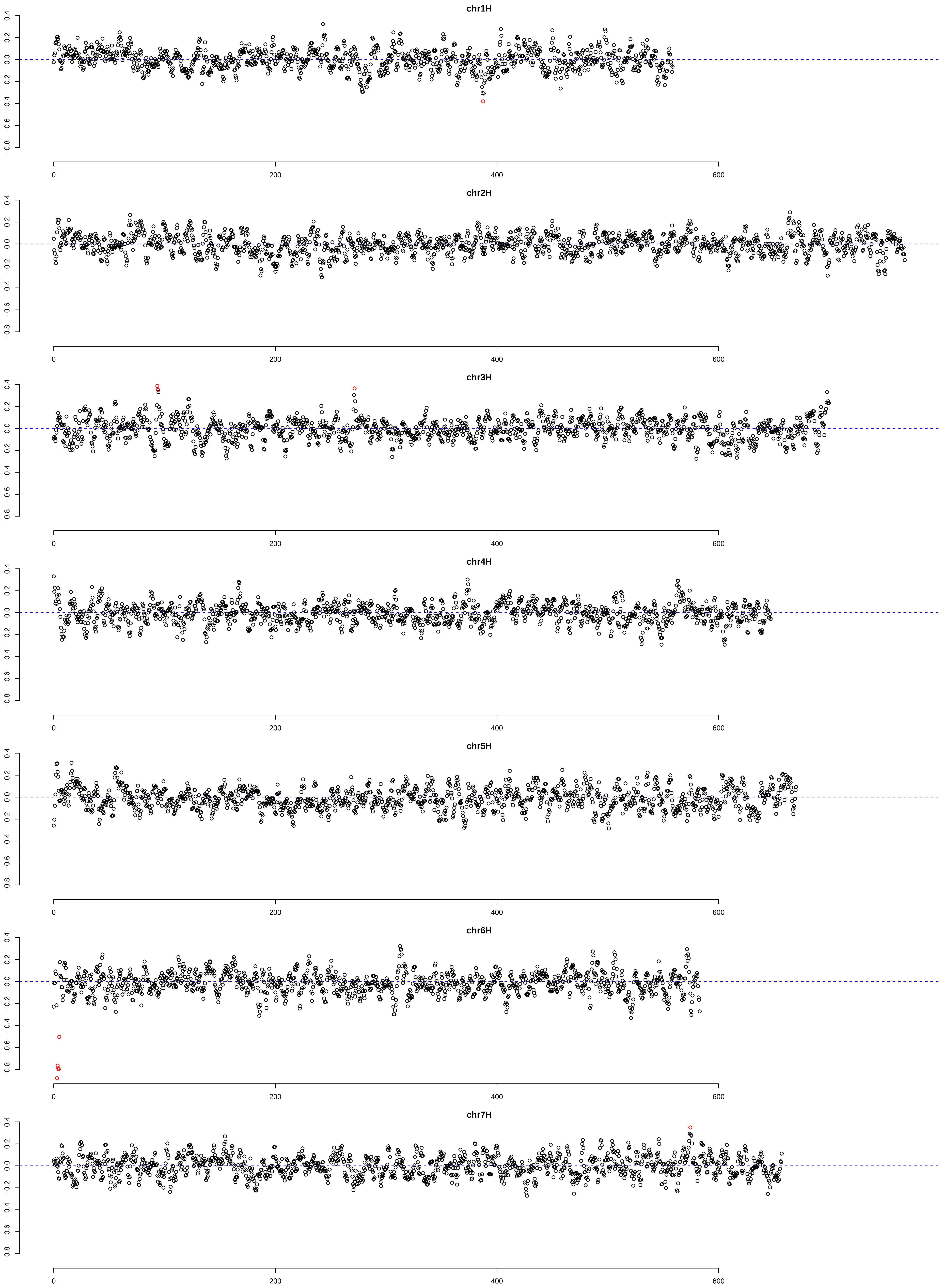
chr1H
chr2H
chr3H
chr4H
chr5H
chr6H
chr7H
0.4
0.2
0.0
−0.2
−0.4
−0.6
−0.8
0
200
400
600

ERR699860

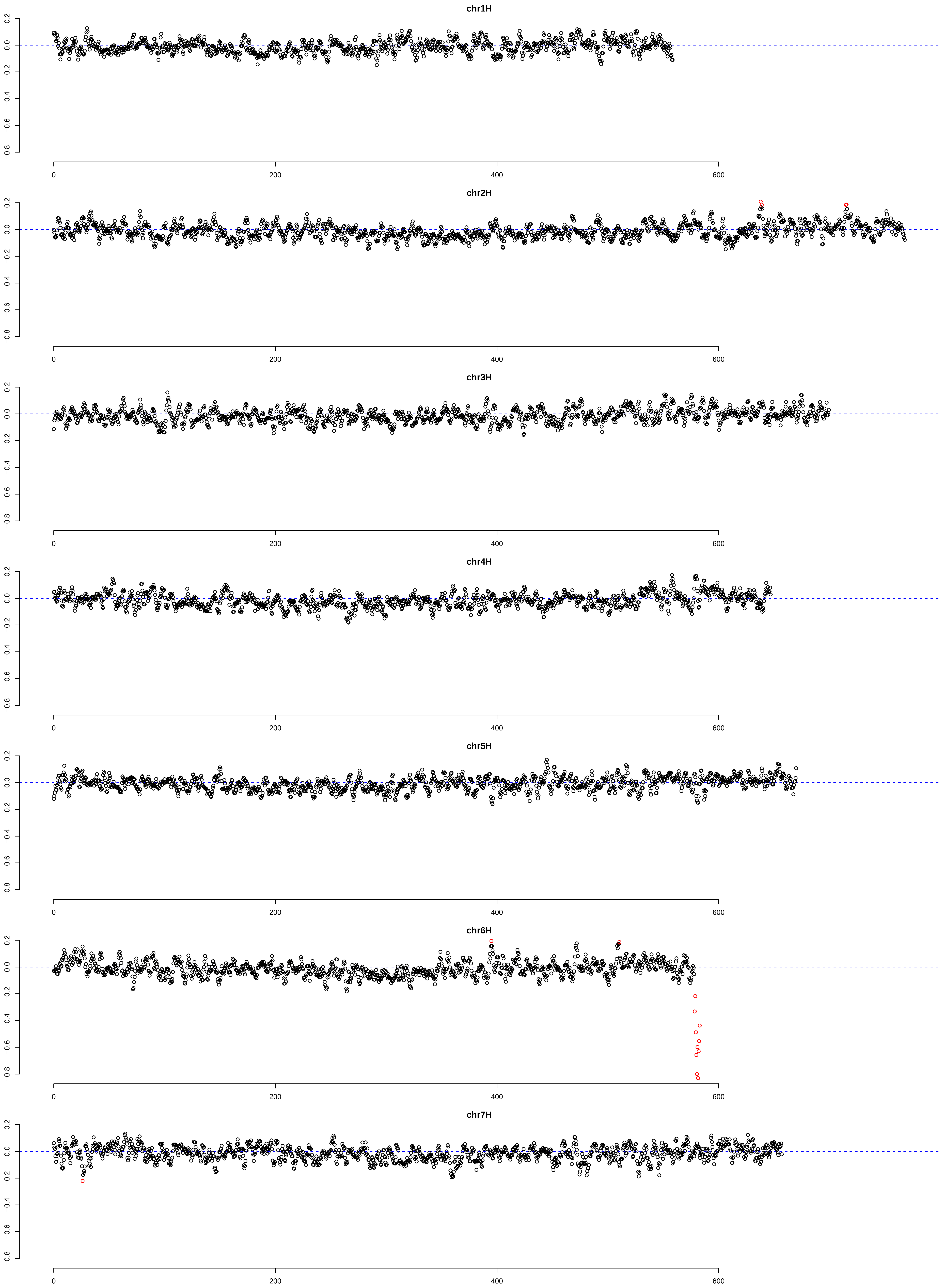
chr1H
chr2H
chr3H
chr4H
chr5H
chr6H
chr7H
0.2
0.0
-0.2
-0.4
-0.6
-0.8
0
200
400
600

ERR699861

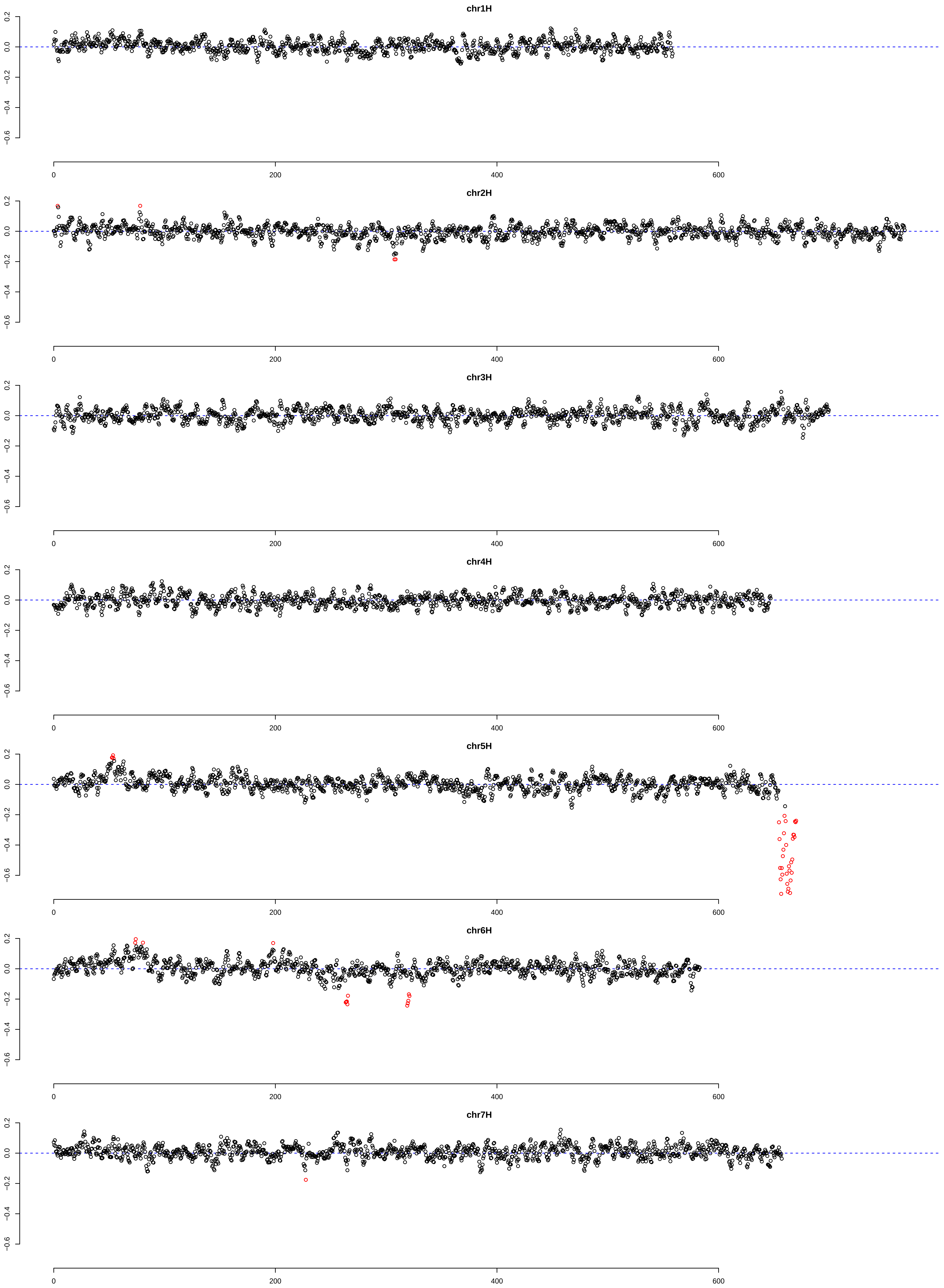
chr1H
chr2H
chr3H
chr4H
chr5H
chr6H
chr7H
0.2
0.0
-0.2
-0.4
-0.6
0
200
400
600

ERR699862

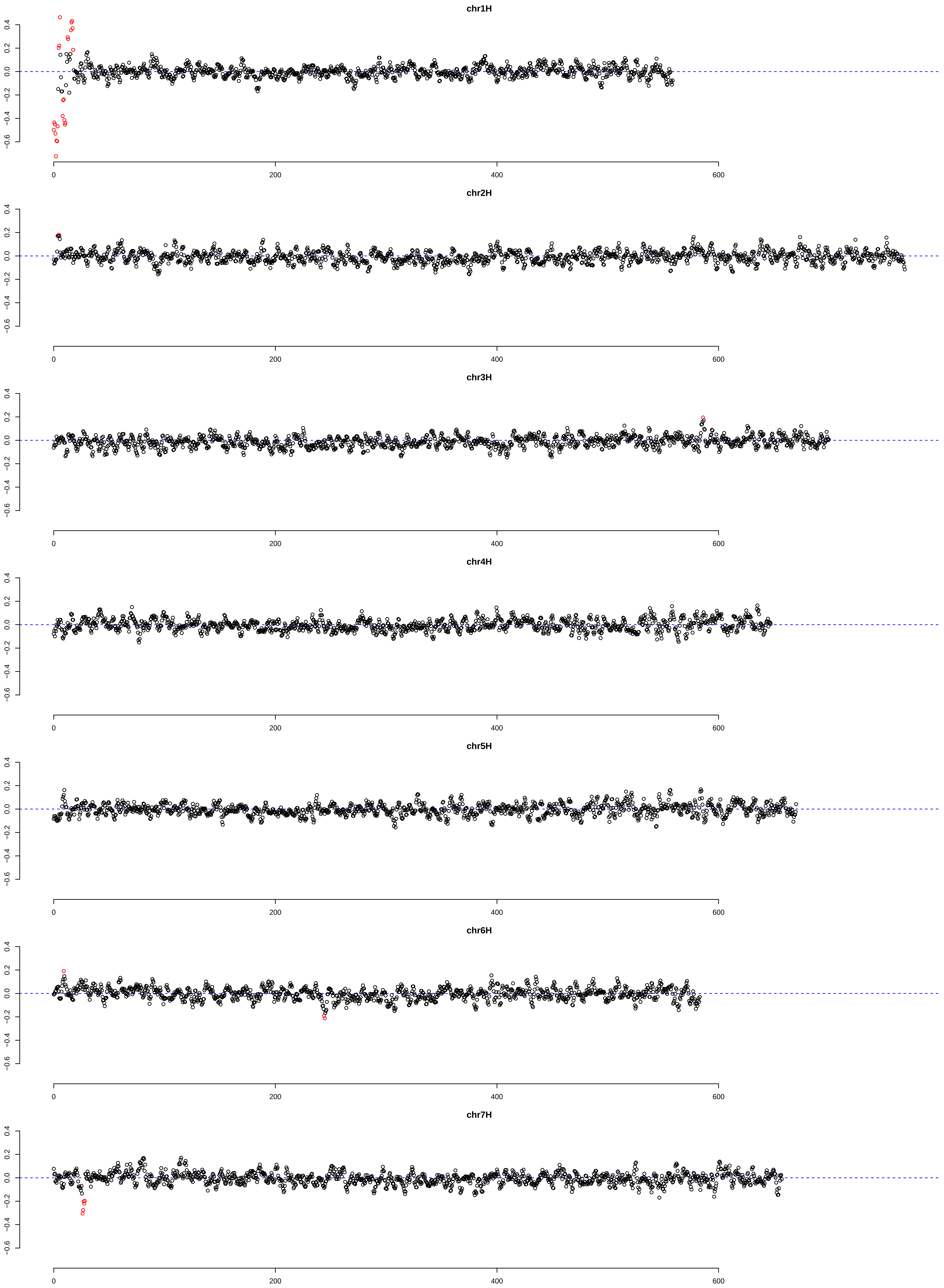

chr1H
chr2H
chr3H
chr4H
chr5H
chr6H
chr7H
0.4
0.2
0.0
-0.2
-0.4
-0.6
0
200
400
600

ERR699863

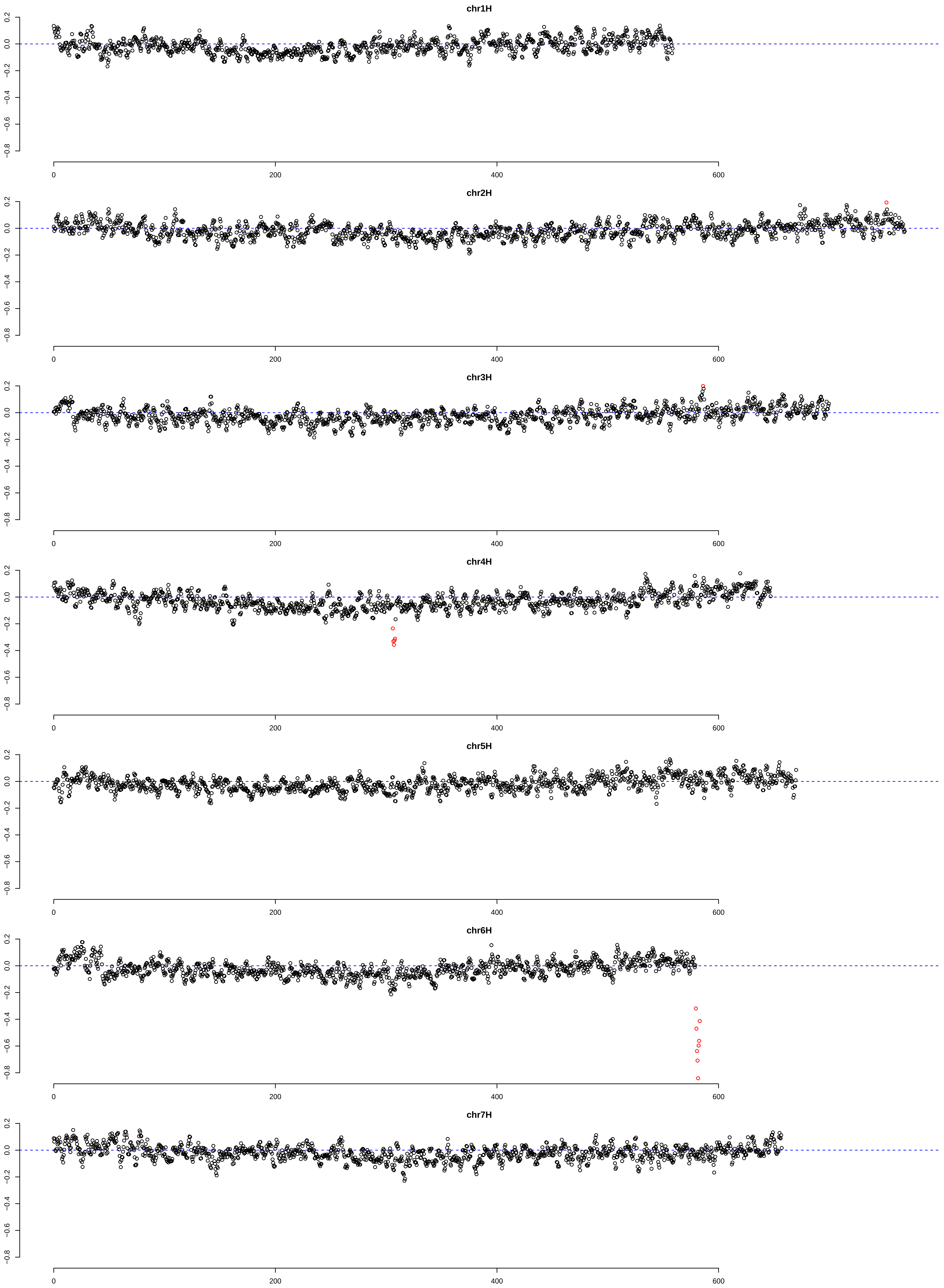
chr1H
chr2H
chr3H
chr4H
chr5H
chr6H
chr7H
0.2
0.0
-0.2
-0.4
-0.6
-0.8
0
200
400
600

ERR699864

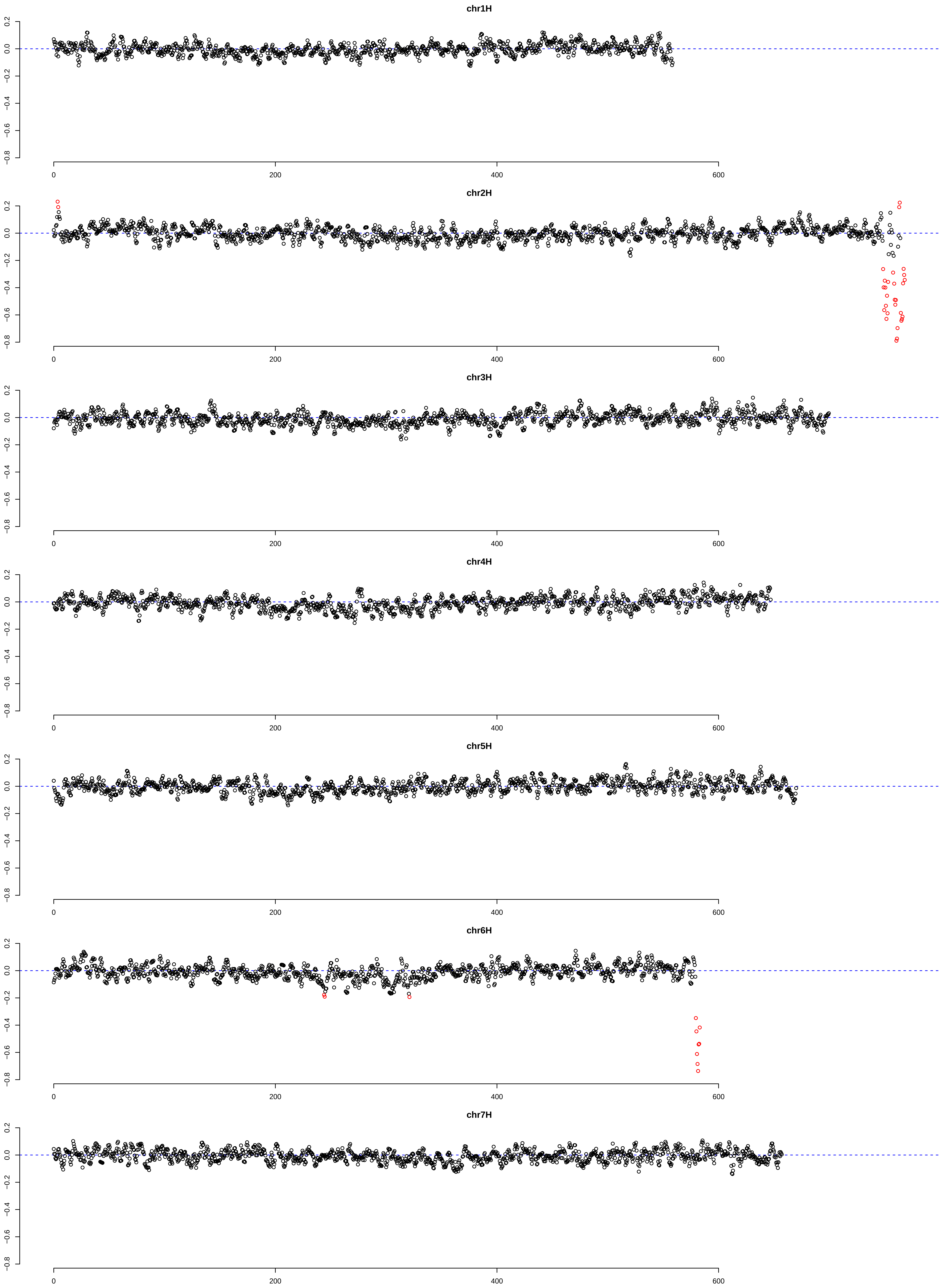

chr1H
chr2H
chr3H
chr4H
chr5H
chr6H
chr7H
0.2
0.0
-0.2
-0.4
-0.6
-0.8
0
200
400
600

ERR699865

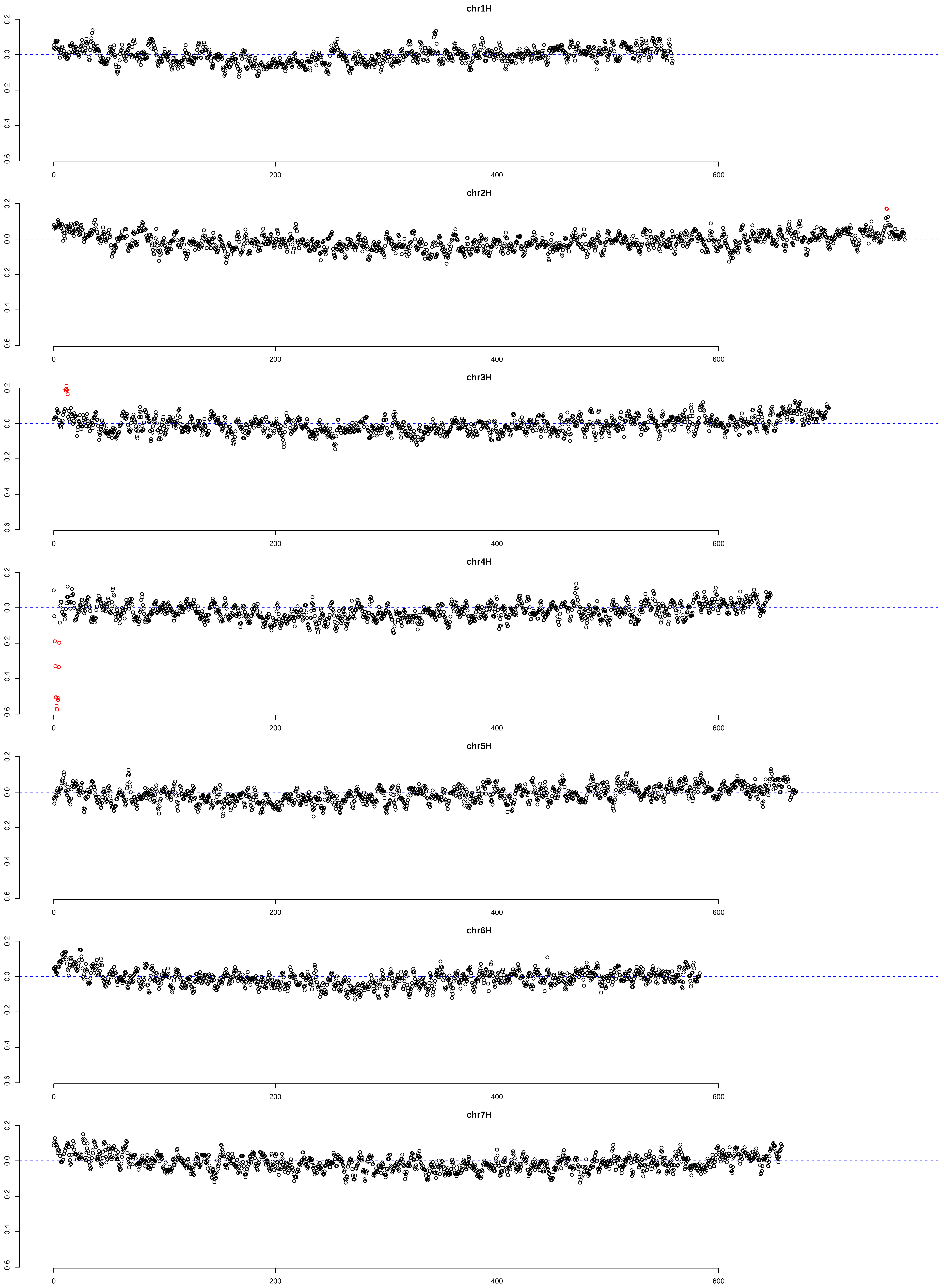
chr1H
chr2H
chr3H
chr4H
chr5H
chr6H
chr7H
0.2
0.0
-0.2
-0.4
-0.6
0
200
400
600

ERR699866

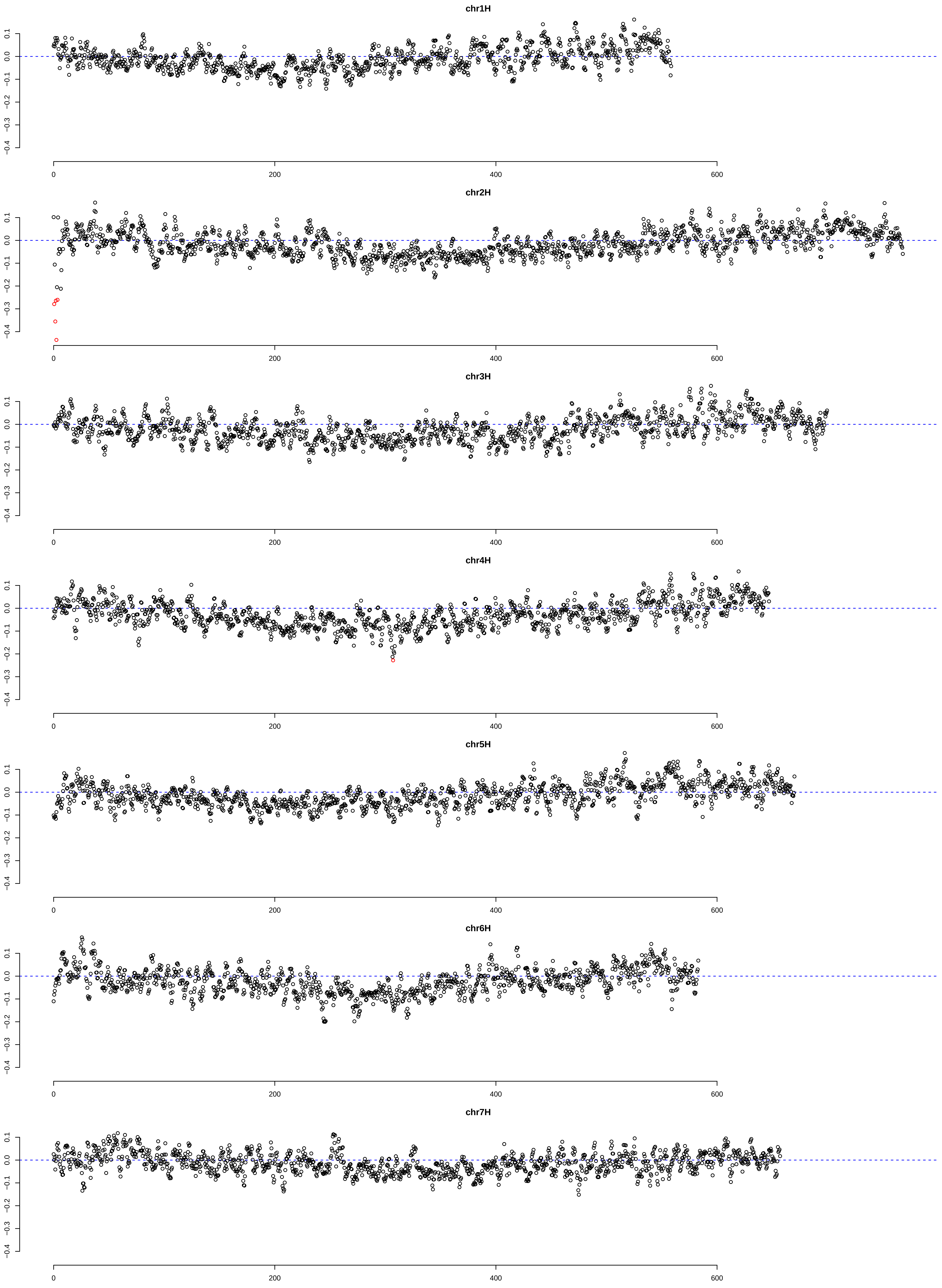
chr1H
chr2H
chr3H
chr4H
chr5H
chr6H
chr7H
0.1
0.0
-0.1
-0.2
-0.3
-0.4
0
200
400
600

ERR699867

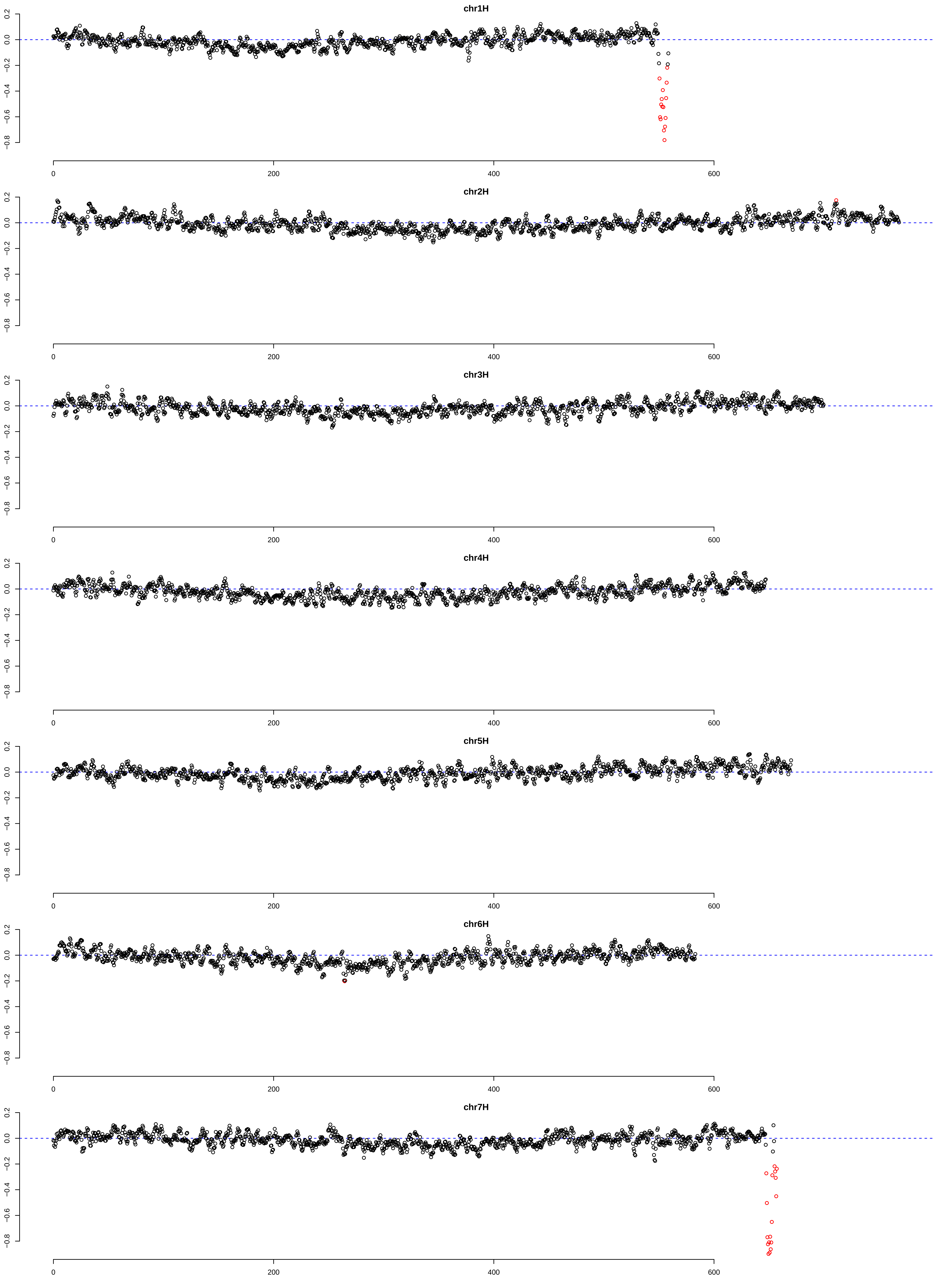
chr1H
chr2H
chr3H
chr4H
chr5H
chr6H
chr7H
0.2
0.0
-0.2
-0.4
-0.6
-0.8
0
200
400
600

ERR699868

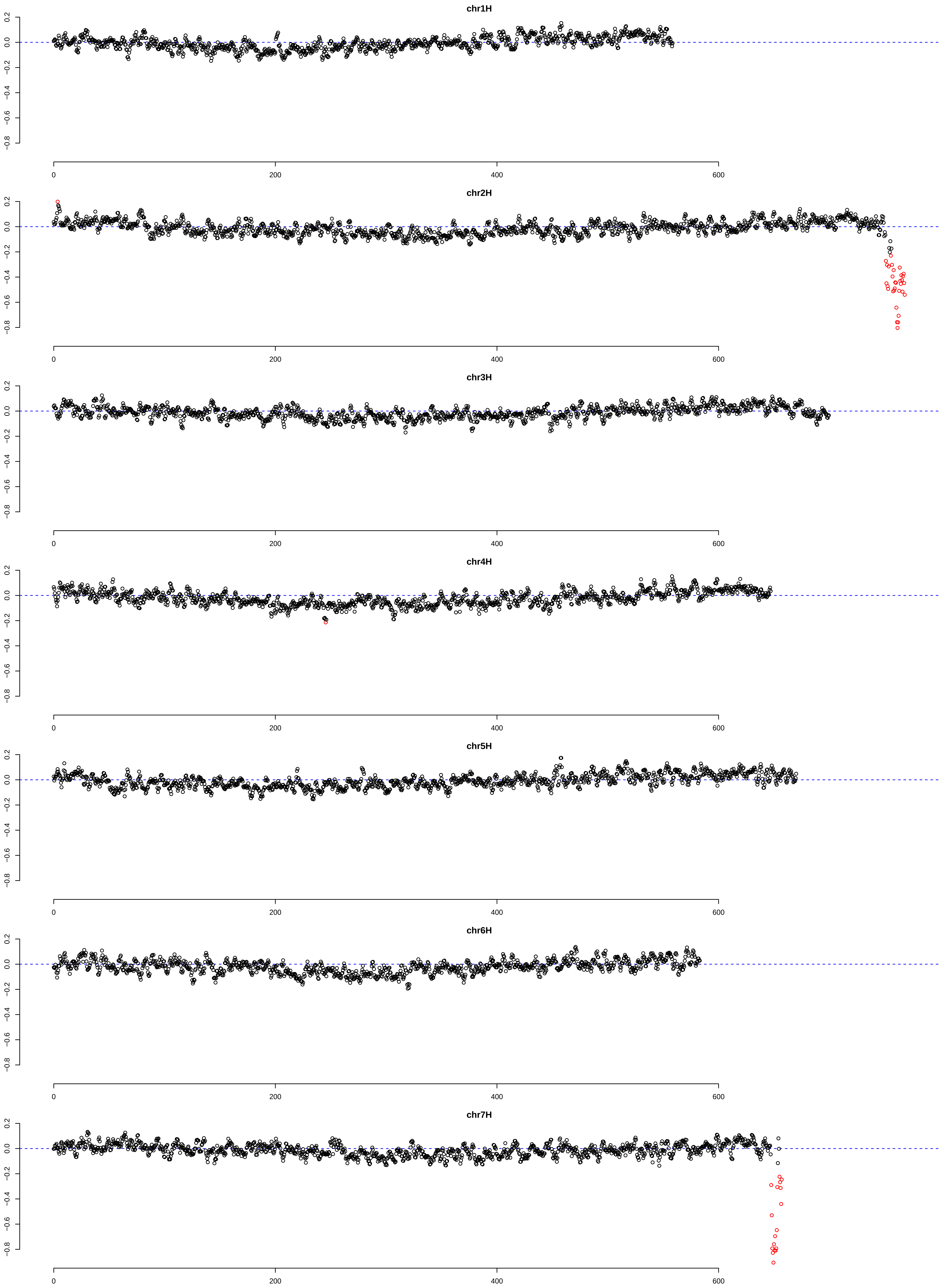
chr1H
chr2H
chr3H
chr4H
chr5H
chr6H
chr7H
0.2
0.0
-0.2
-0.4
-0.6
-0.8
0
200
400
600

ERR699869

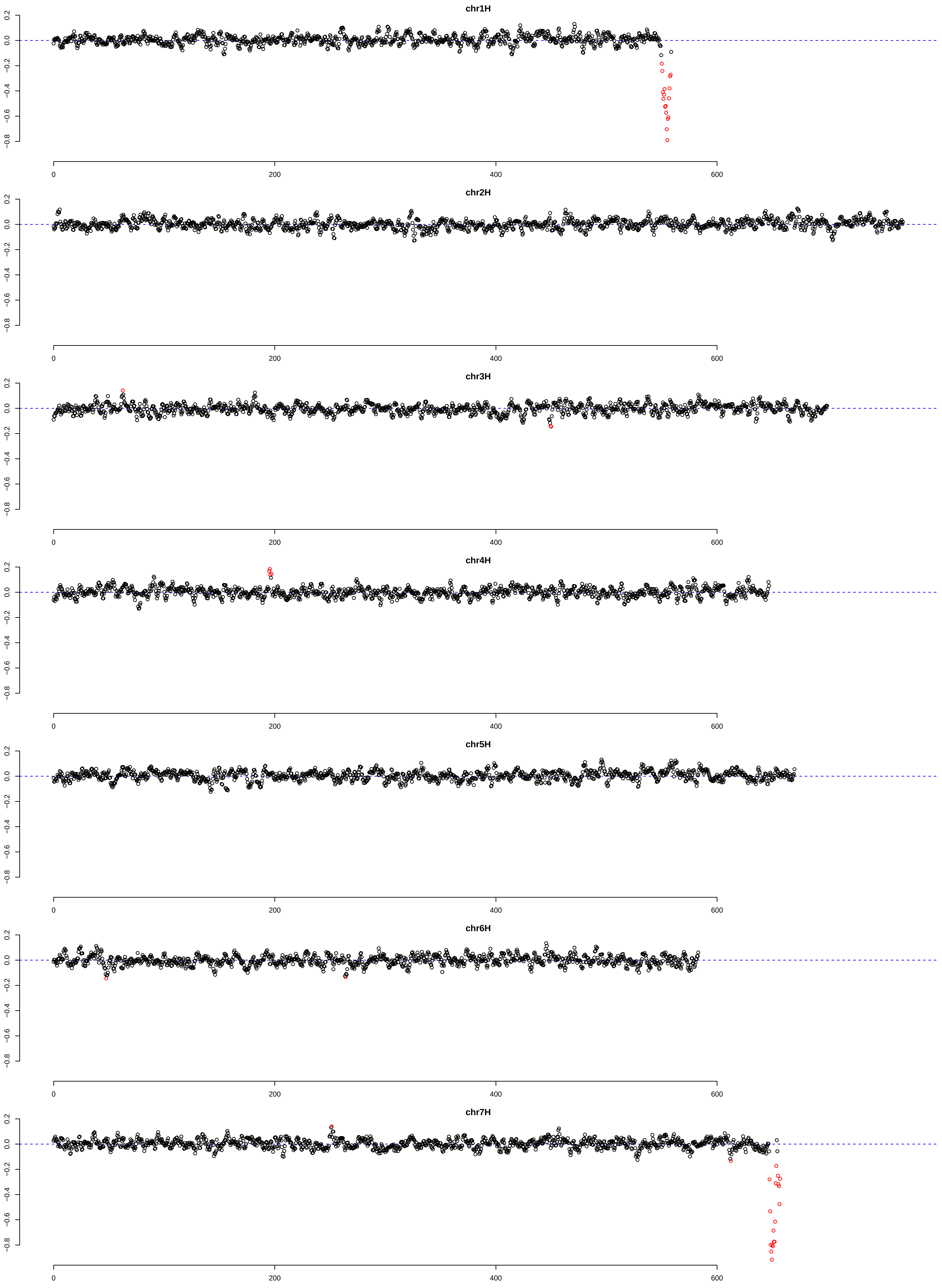

chr1H
chr2H
chr3H
chr4H
chr5H
chr6H
chr7H
0.2
0.0
-0.2
-0.4
-0.6
-0.8
0
200
400
600

ERR699870

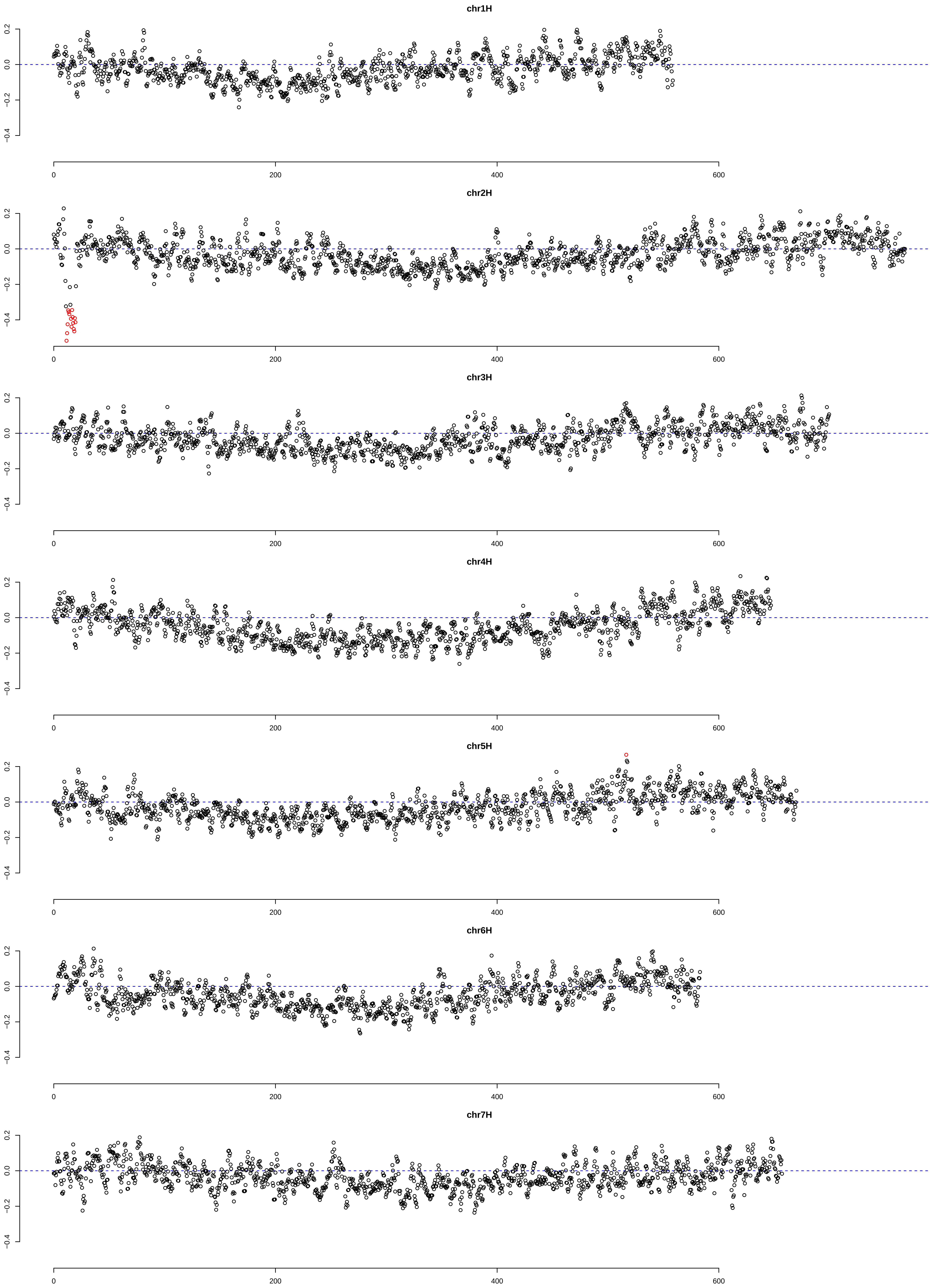
chr1H
chr2H
chr3H
chr4H
chr5H
chr6H
chr7H
0.2
0.0
-0.2
-0.4
0
200
400
600

ERR699871

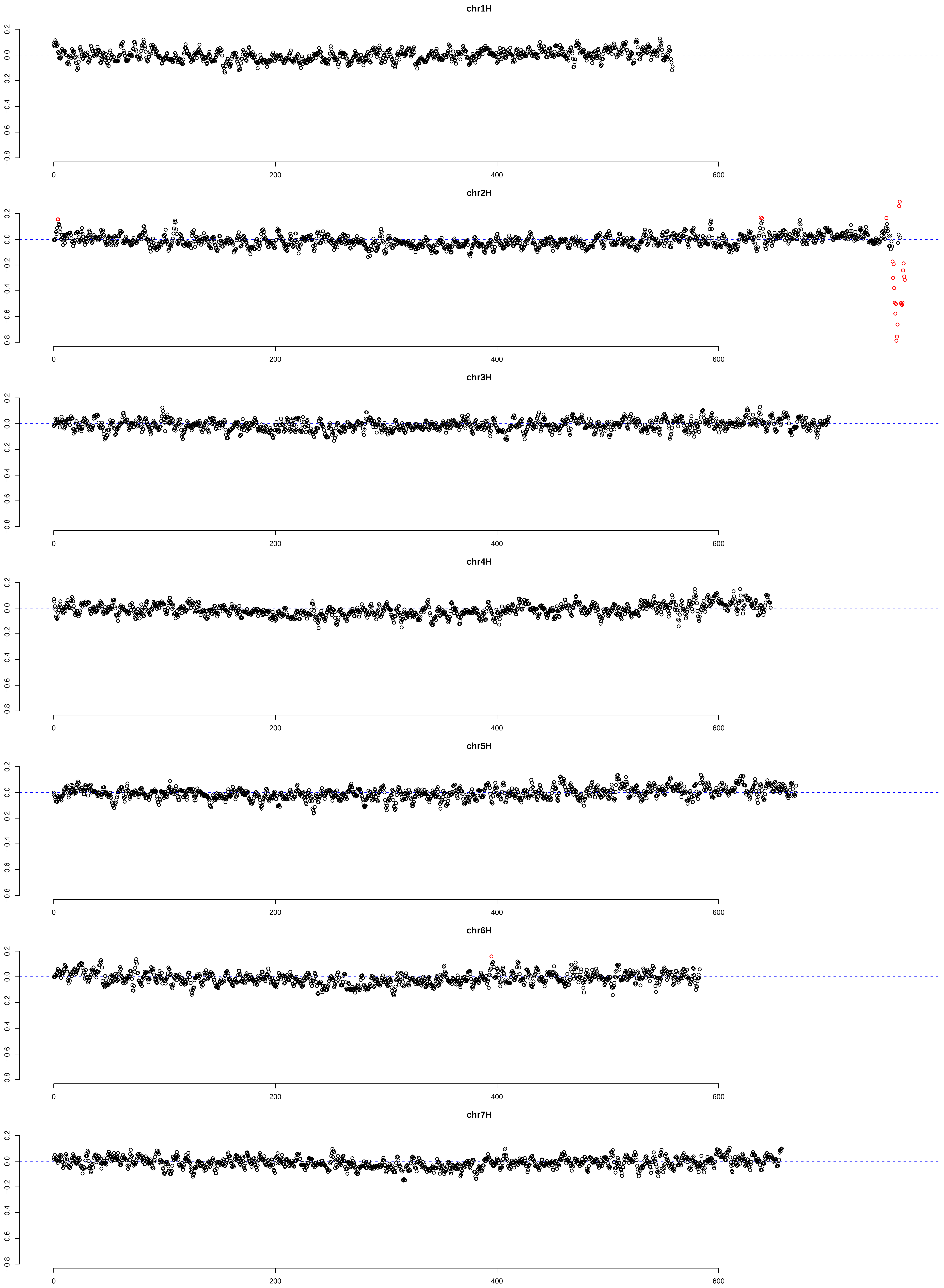

chr1H
chr2H
chr3H
chr4H
chr5H
chr6H
chr7H
0.2
0.0
-0.2
-0.4
-0.6
-0.8
0
200
400
600

ERR699872

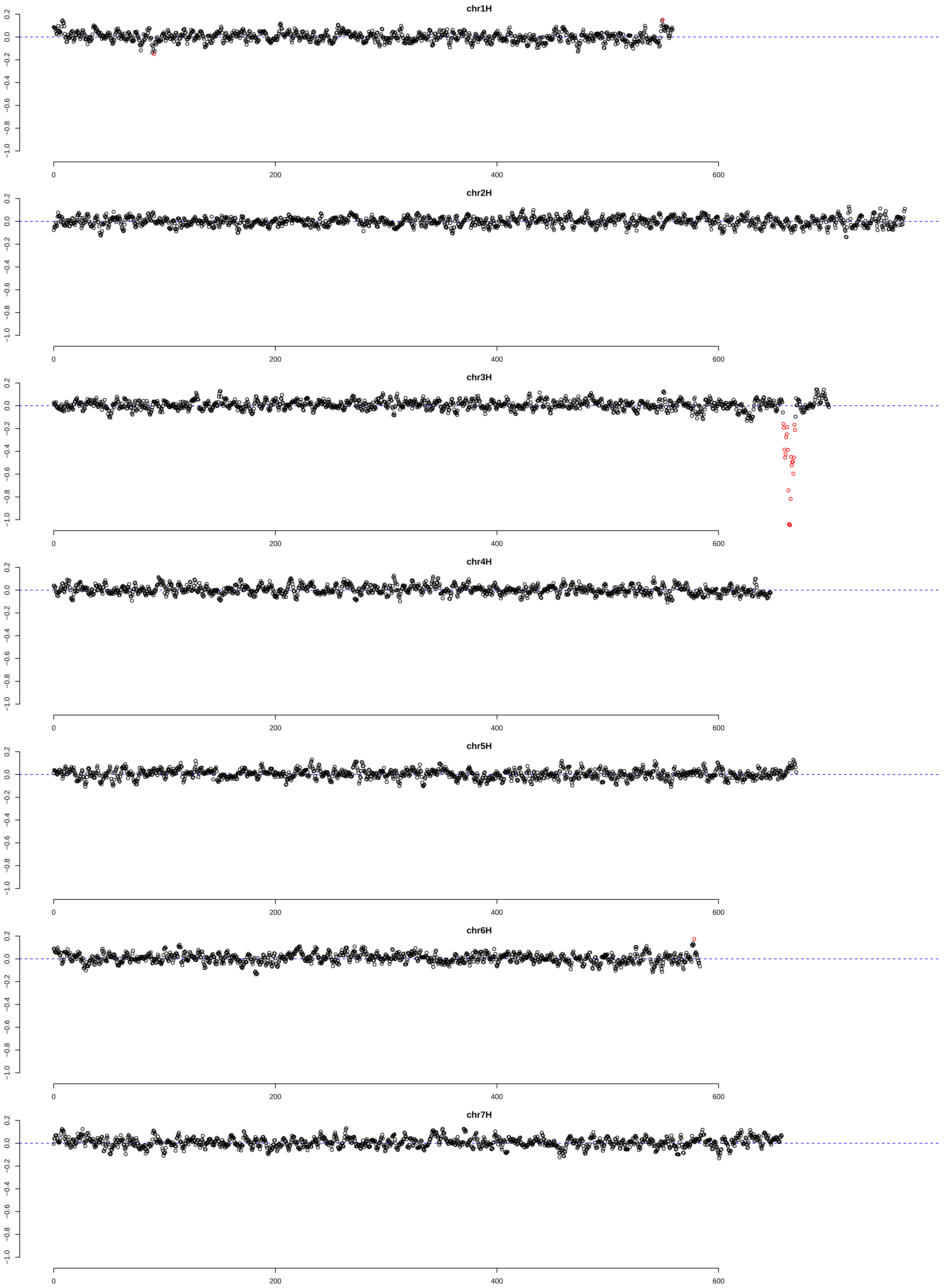
chr1H
chr2H
chr3H
chr4H
chr5H
chr6H
chr7H
0.2
0.0
-0.2
-0.4
-0.6
-0.8
-1.0
0
200
400
600

ERR699873

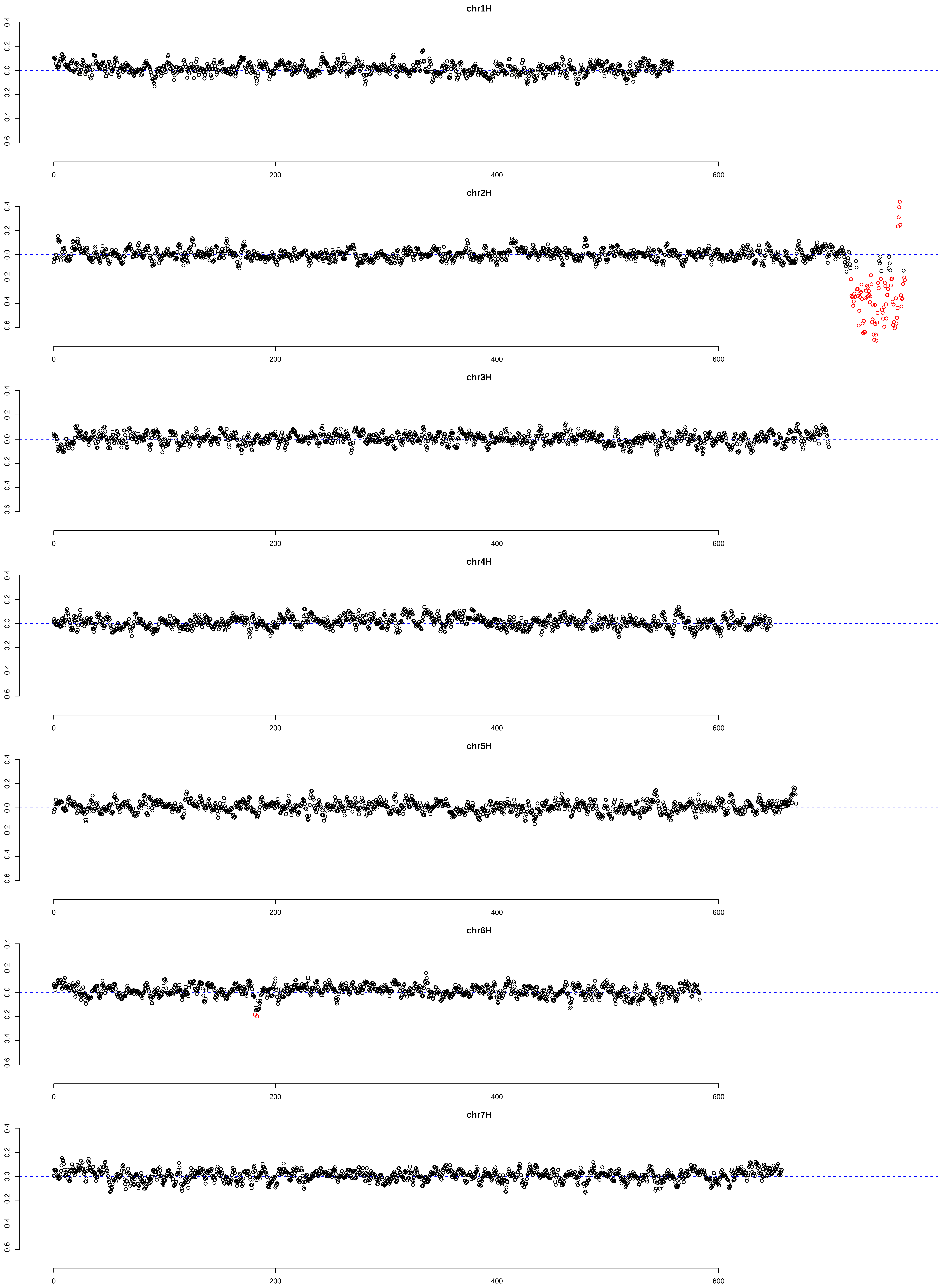

chr1H
chr2H
chr3H
chr4H
chr5H
chr6H
chr7H
0.4
0.2
0.0
-0.2
-0.4
-0.6
0
200
400
600

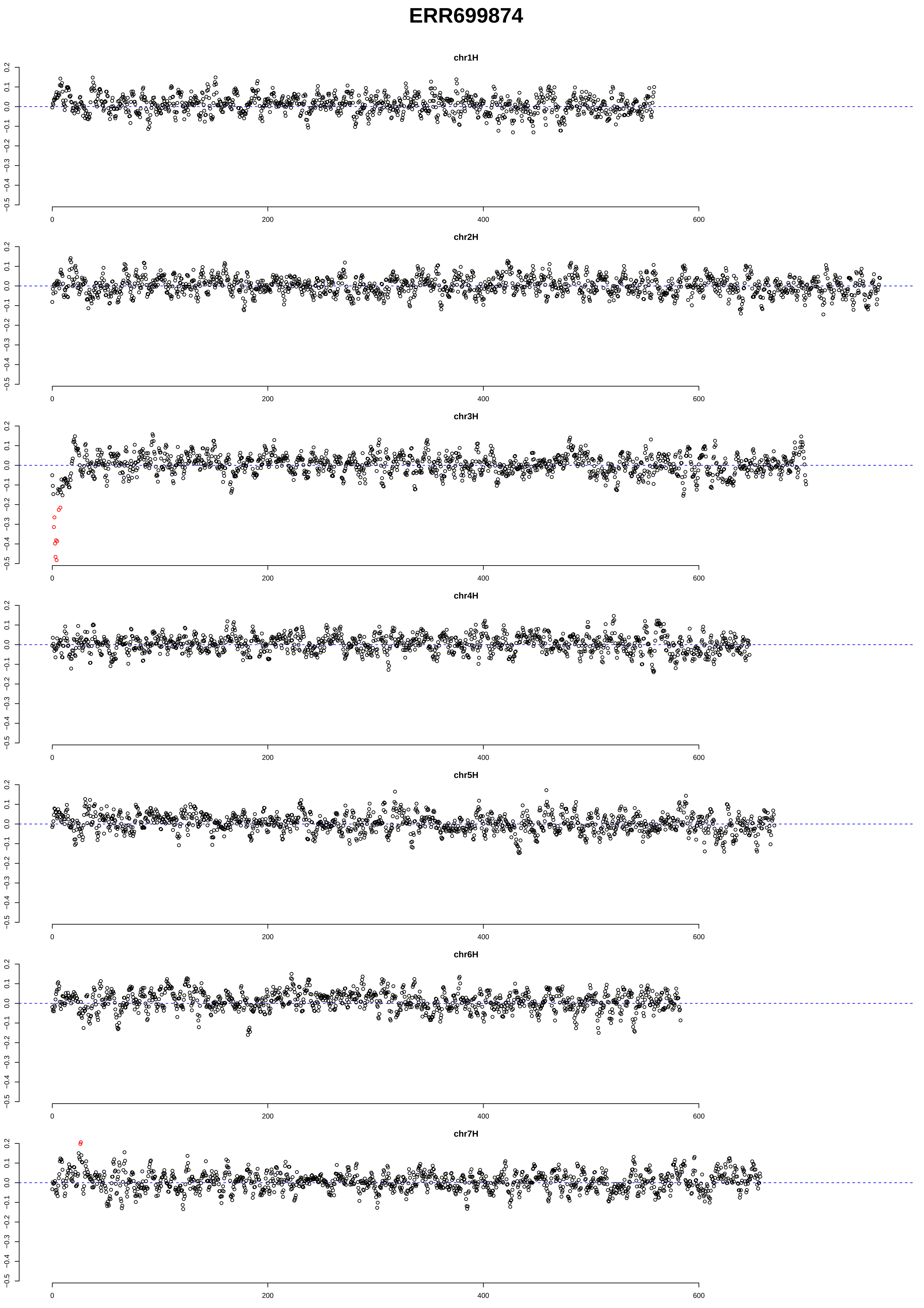
ERR699874
chr1H
chr2H
chr3H
chr4H
chr5H
chr6H
chr7H
0
200
400
600
0.2
0.1
0.0
-0.1
-0.2
-0.3
-0.4
-0.5

ERR699875

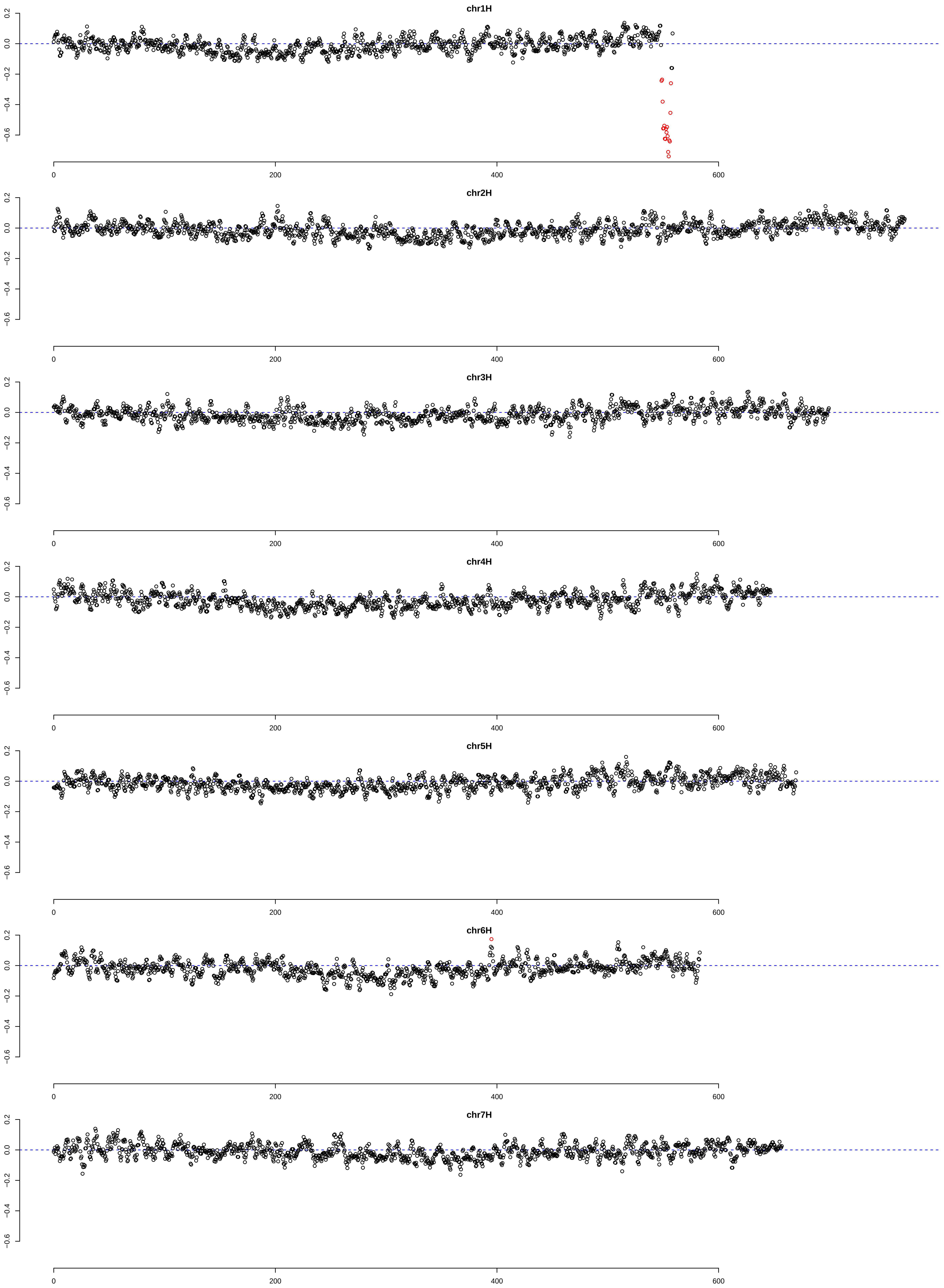
chr1H
chr2H
chr3H
chr4H
chr5H
chr6H
chr7H
0.2
0.0
-0.2
-0.4
-0.6
0
200
400
600

ERR699876

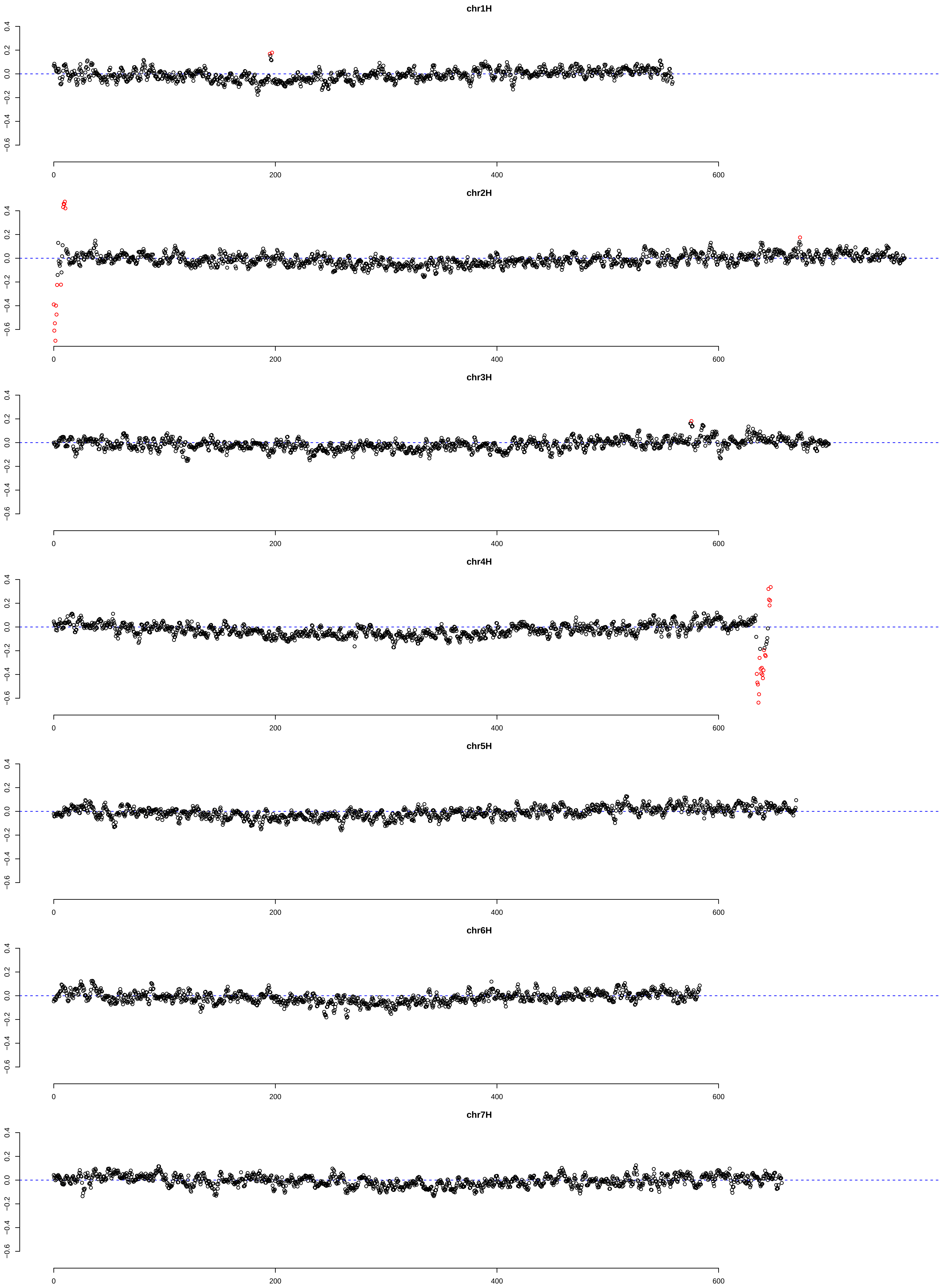
chr1H
chr2H
chr3H
chr4H
chr5H
chr6H
chr7H
0.4
0.2
0.0
-0.2
-0.4
-0.6
0
200
400
600

ERR699877

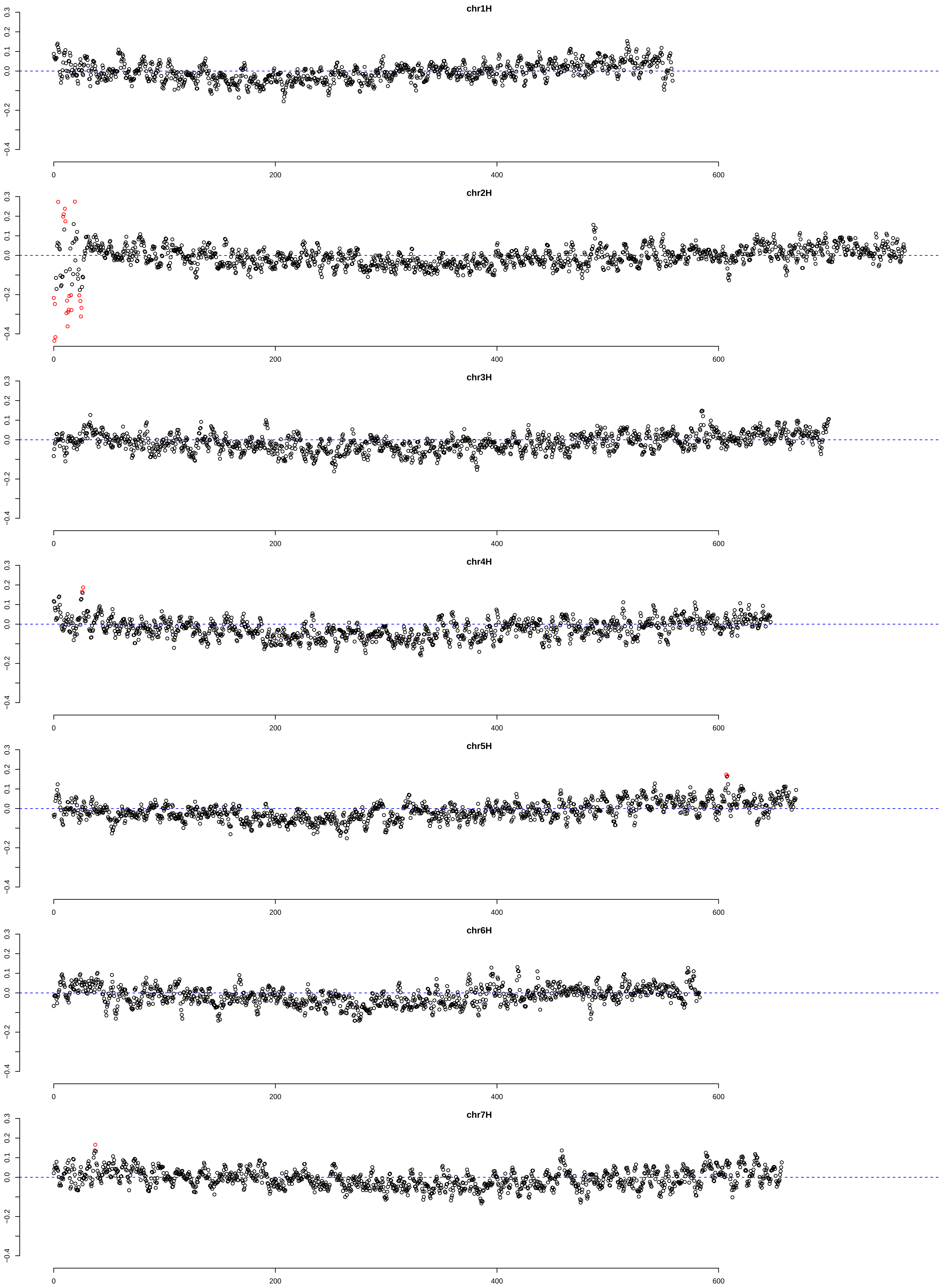

chr1H
chr2H
chr3H
chr4H
chr5H
chr6H
chr7H
0
200
400
600
0.3
0.2
0.1
0.0
-0.2
-0.4

ERR699878

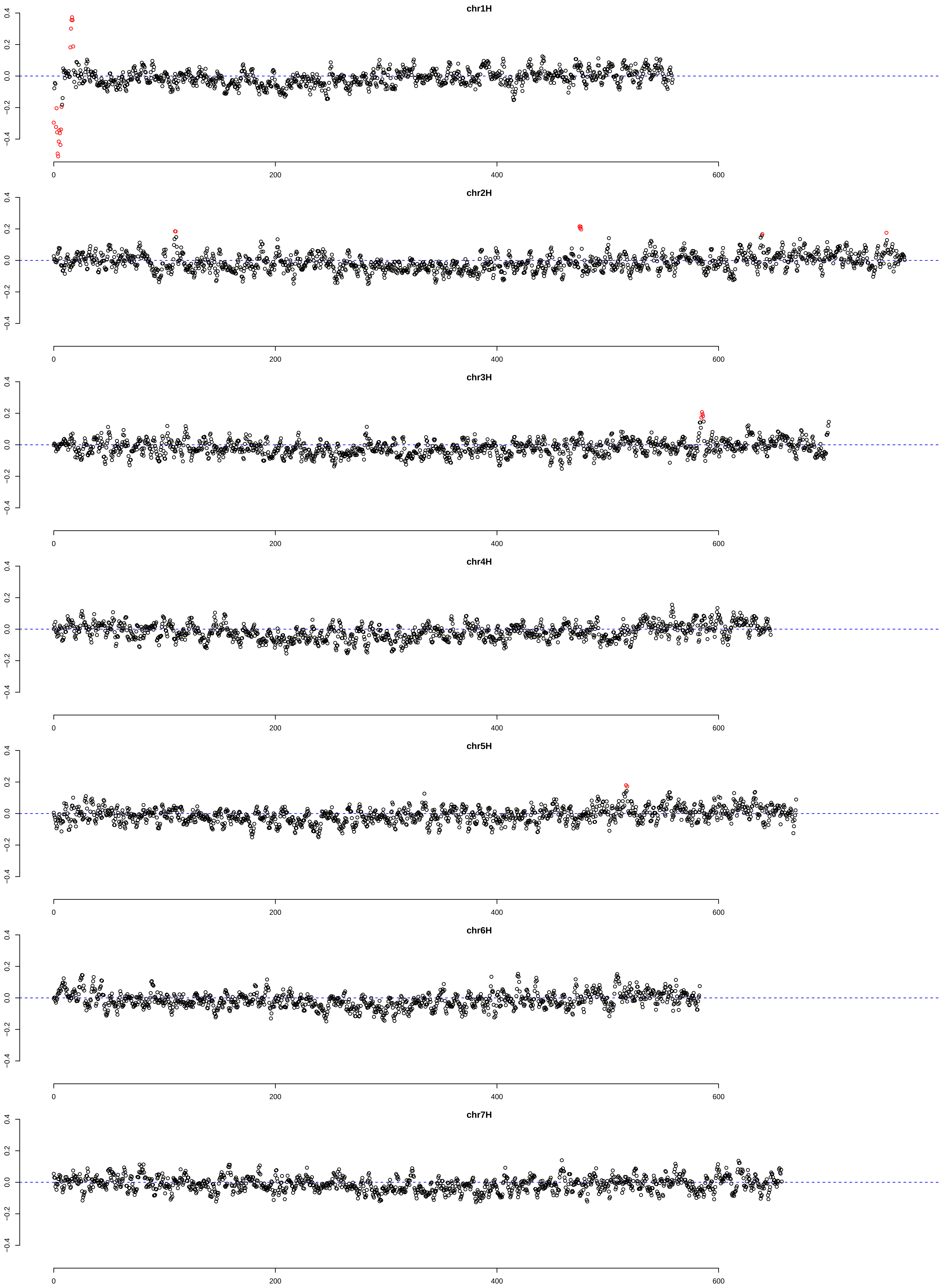

ERR699879

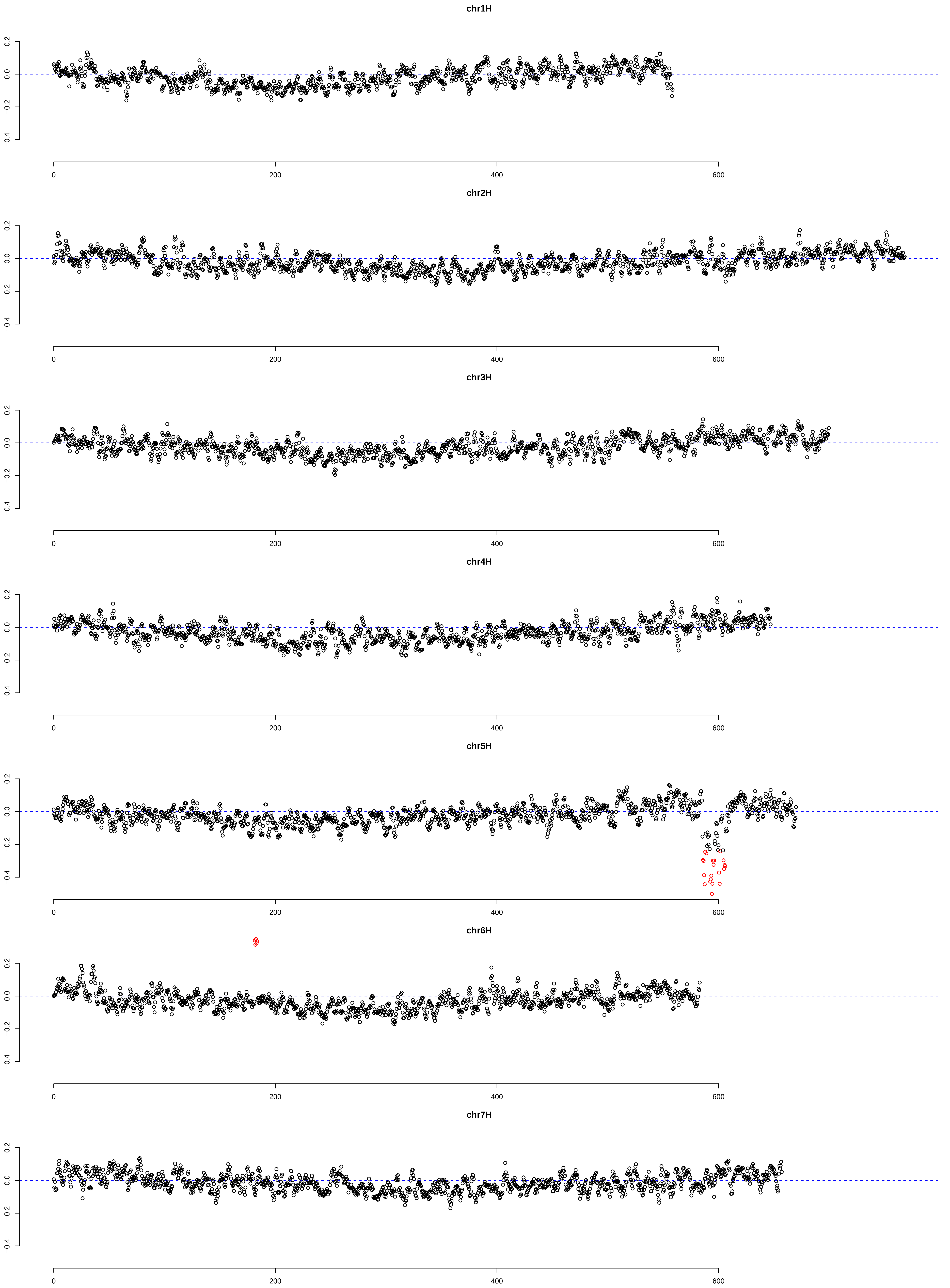
chr1H
chr2H
chr3H
chr4H
chr5H
chr6H
chr7H
0.2
0.0
-0.2
-0.4
0
200
400
600

ERR699880

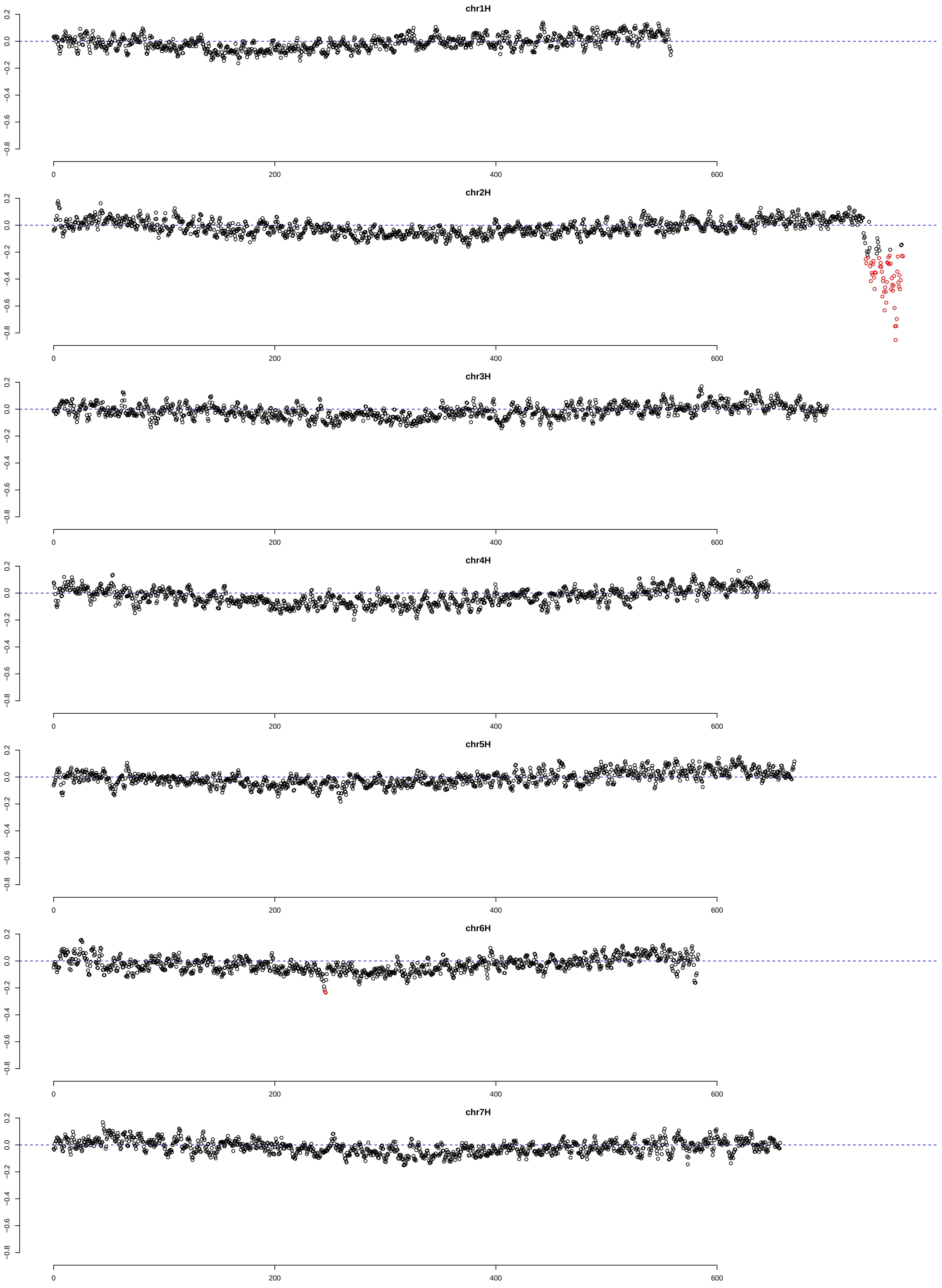

chr1H
chr2H
chr3H
chr4H
chr5H
chr6H
chr7H
0.2
0.0
-0.2
-0.4
-0.6
-0.8
0
200
400
600

ERR699881

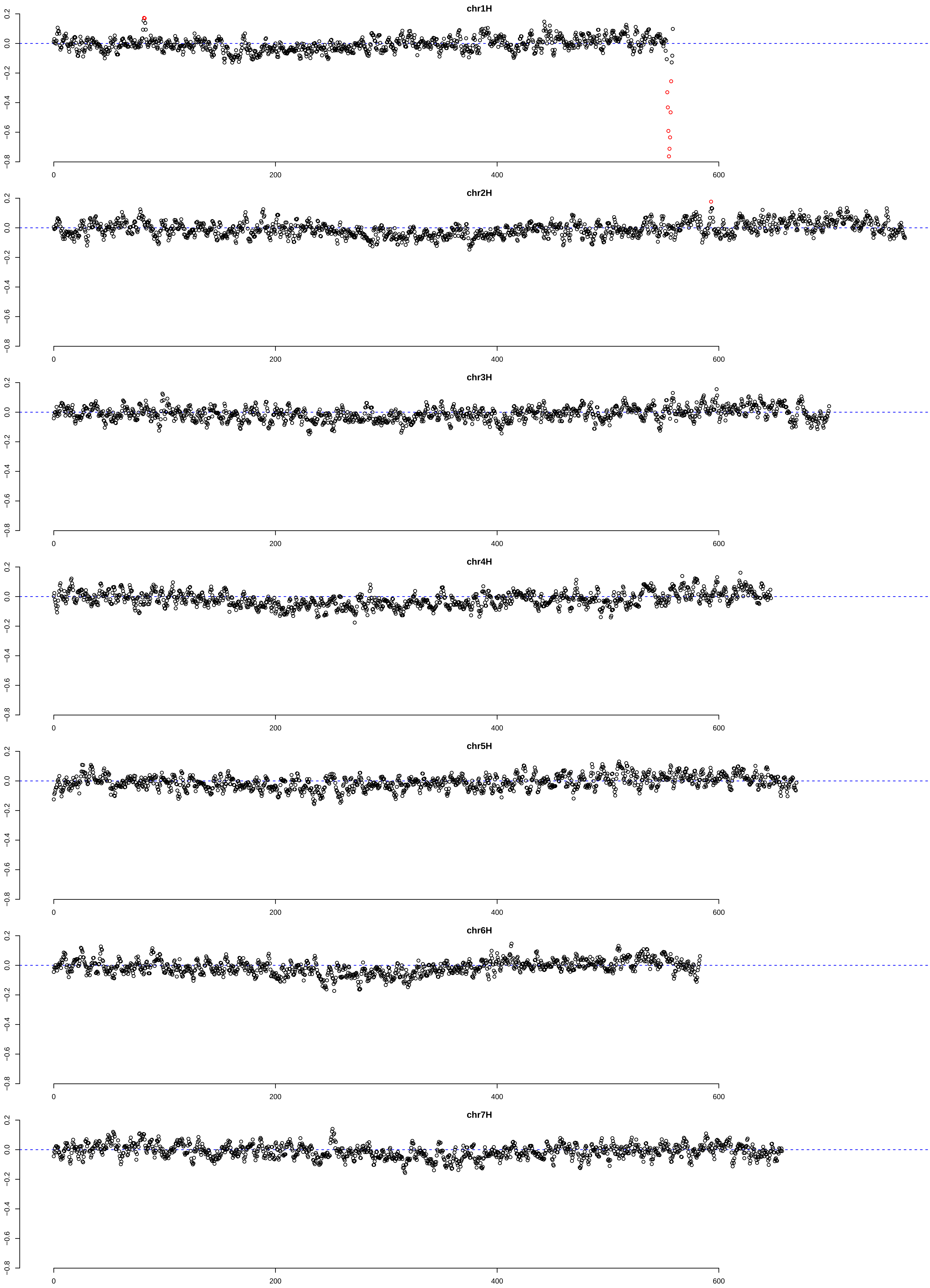
chr1H
chr2H
chr3H
chr4H
chr5H
chr6H
chr7H
0.2
0.0
-0.2
-0.4
-0.6
-0.8
0
200
400
600

ERR699882

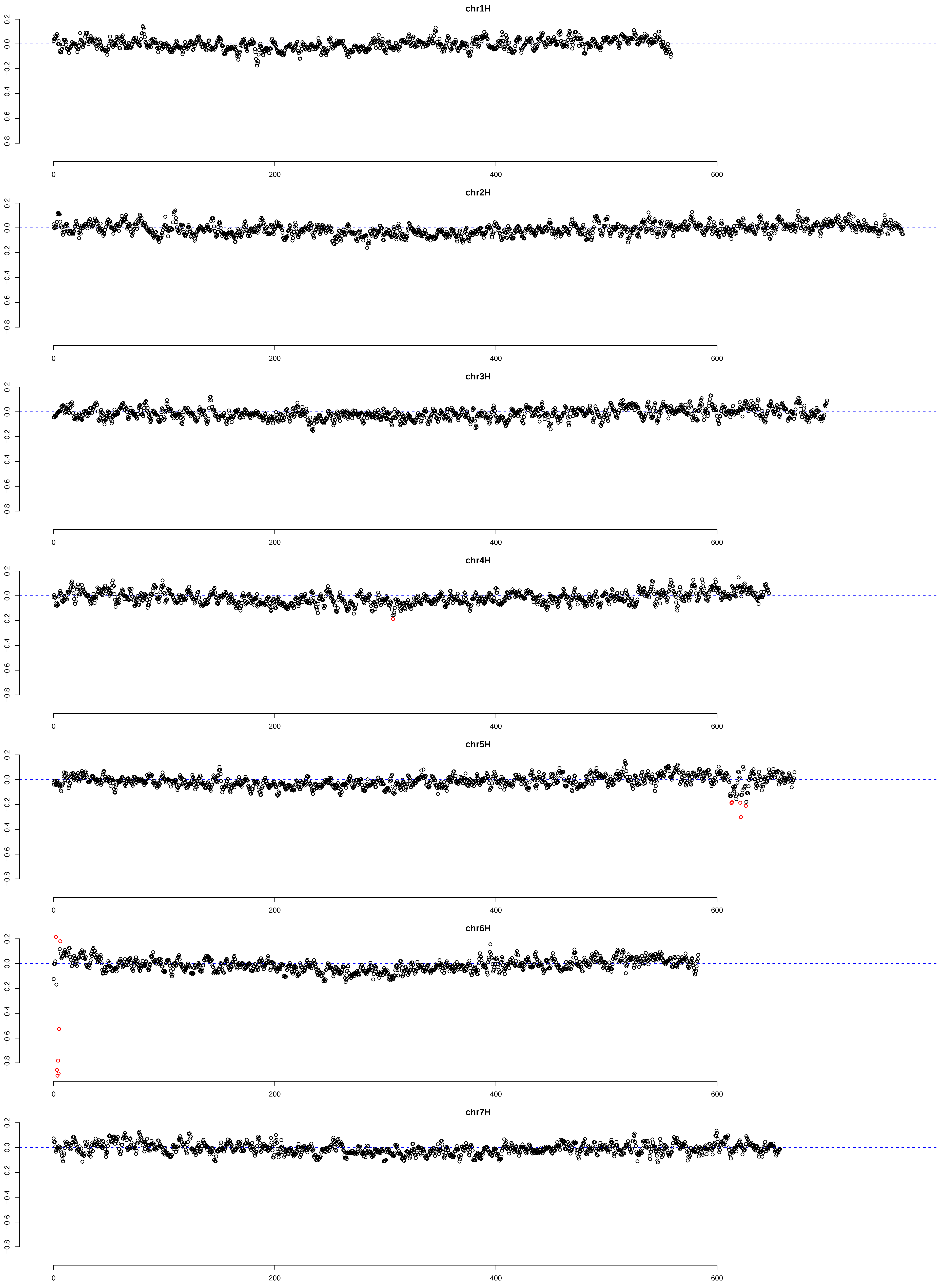

chr1H
chr2H
chr3H
chr4H
chr5H
chr6H
chr7H
0.2
0.0
-0.2
-0.4
-0.6
-0.8
0
200
400
600

ERR699883

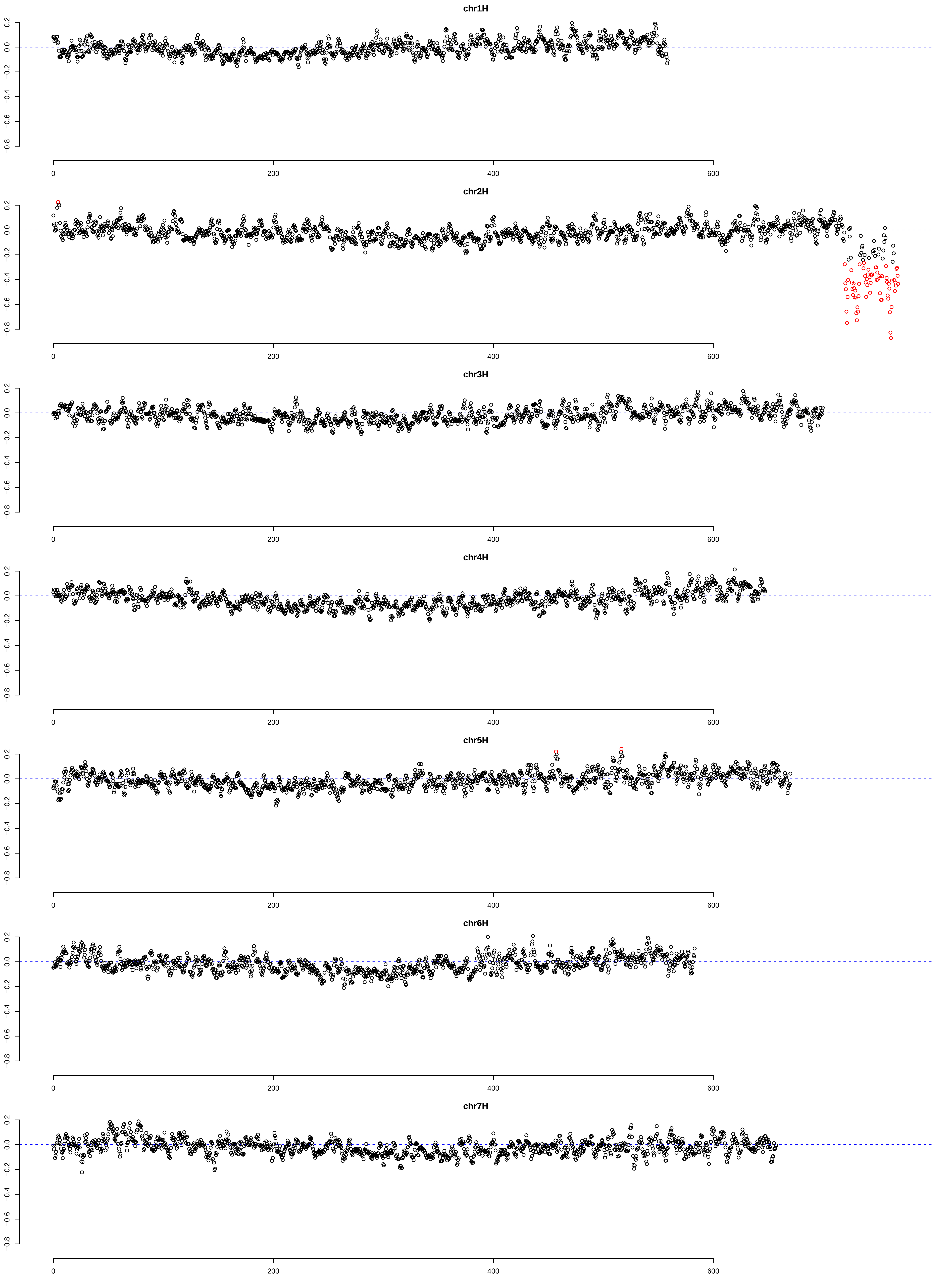
chr1H
chr2H
chr3H
chr4H
chr5H
chr6H
chr7H
0.2
0.0
−0.2
−0.4
−0.6
−0.8
0
200
400
600

ERR699884

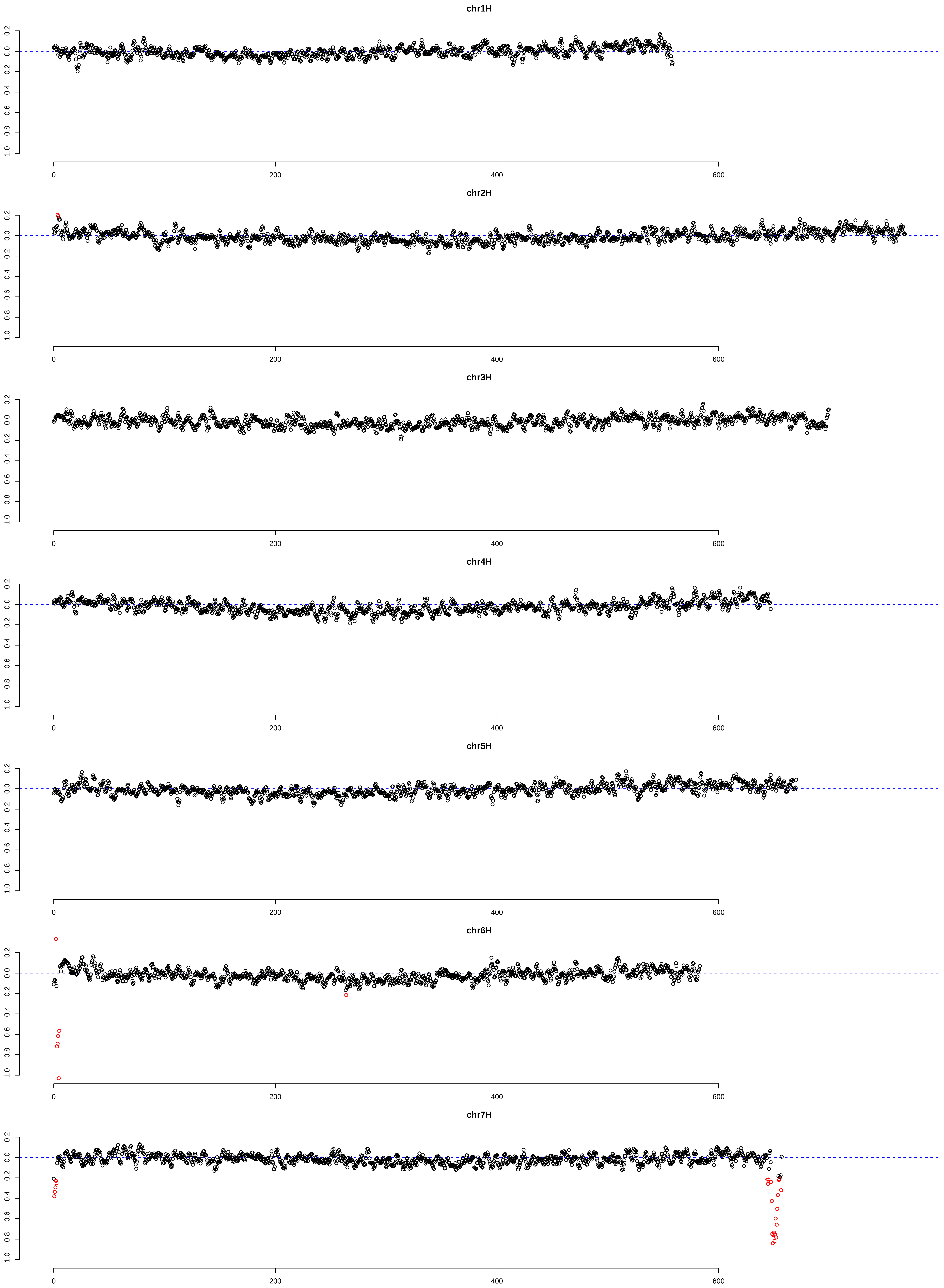
chr1H
chr2H
chr3H
chr4H
chr5H
chr6H
chr7H
0.2
0.0
−0.2
−0.4
−0.6
−0.8
−1.0
0
200
400
600

ERR699885

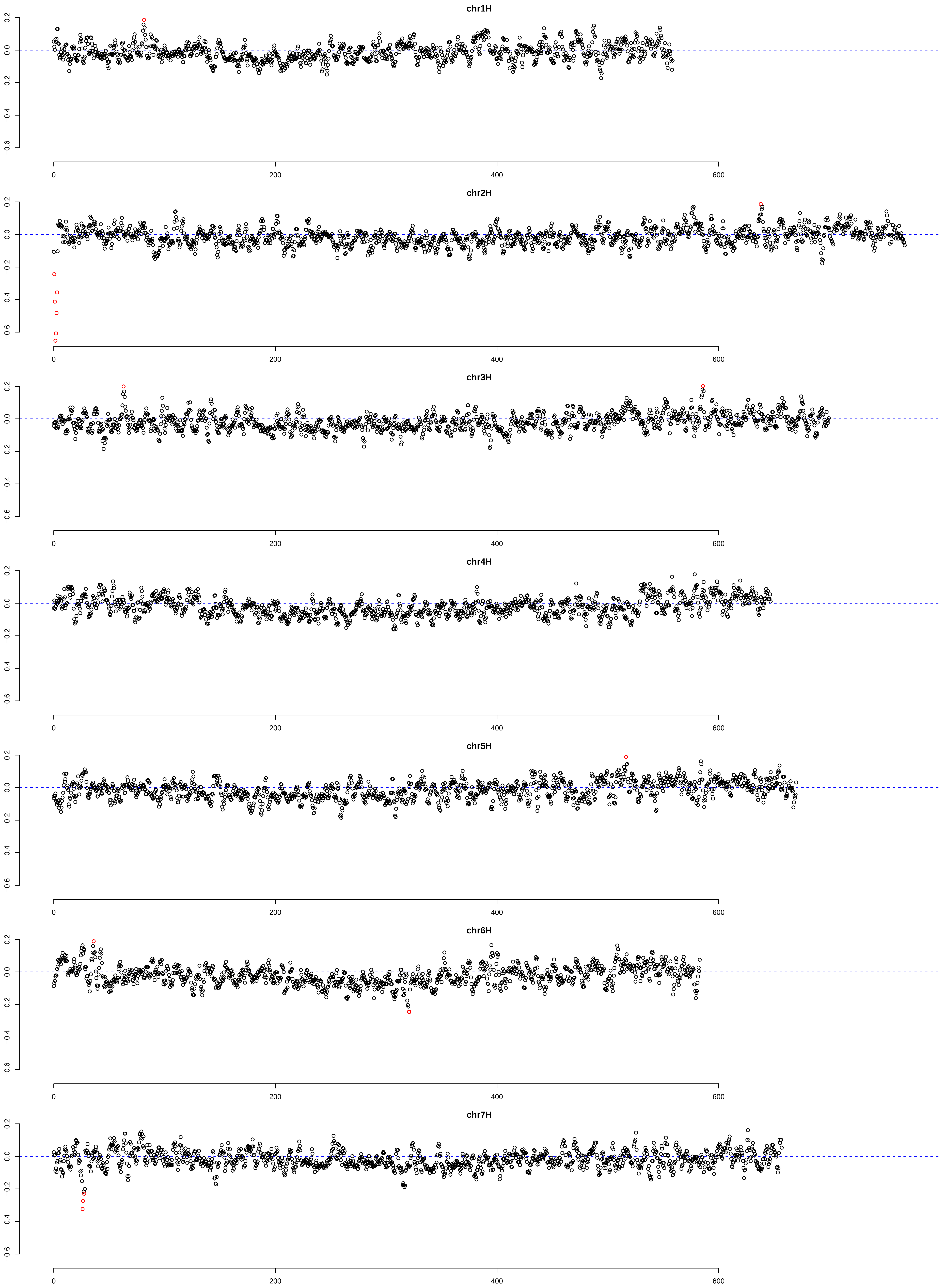
chr1H
chr2H
chr3H
chr4H
chr5H
chr6H
chr7H
0.2
0.0
-0.2
-0.4
-0.6
0
200
400
600

ERR699886

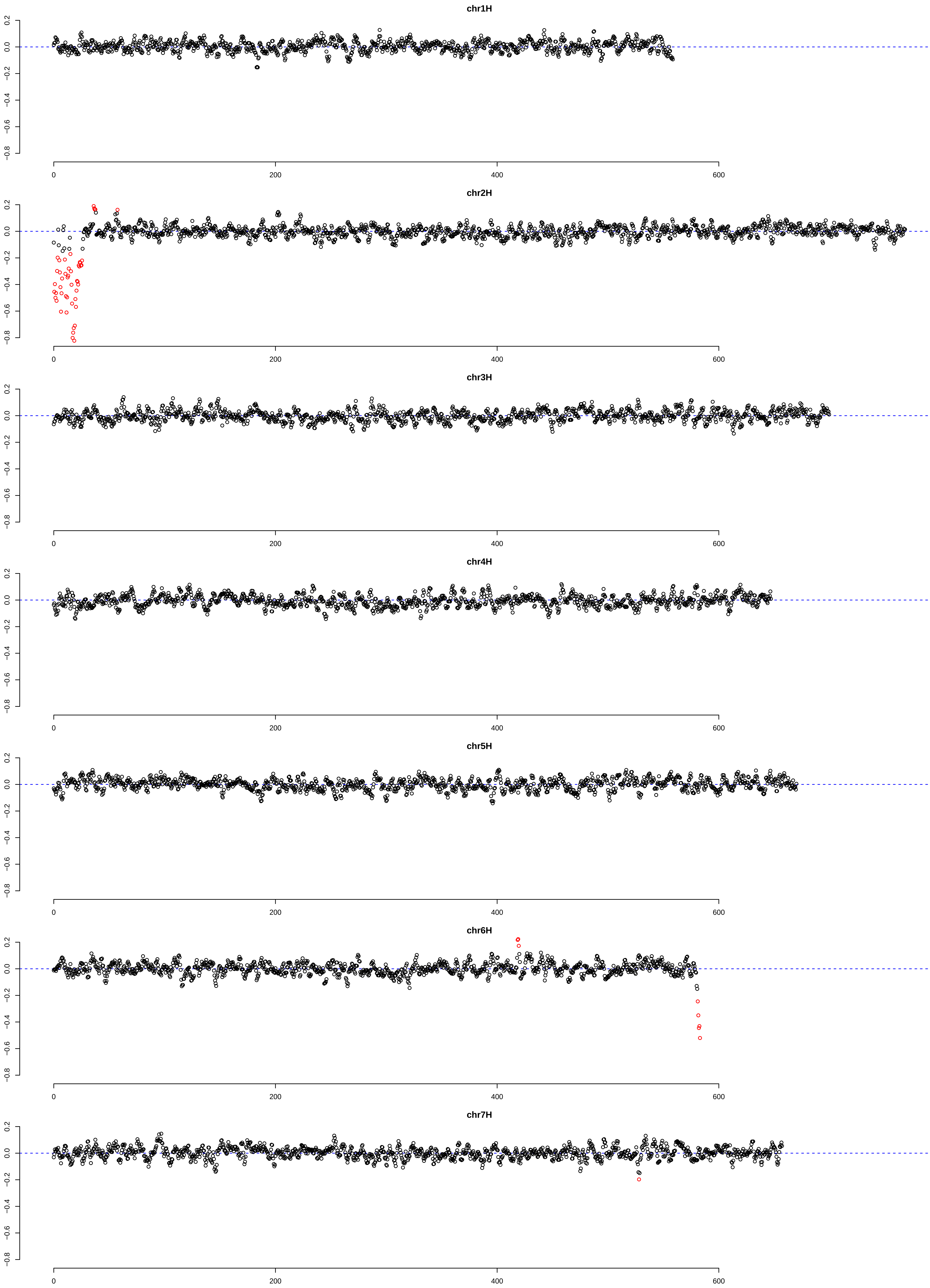

chr1H
0.2
0.0
-0.2
-0.4
-0.6
-0.8
0
200
400
600
chr2H
chr3H
chr4H
chr5H
chr6H
chr7H

ERR699887

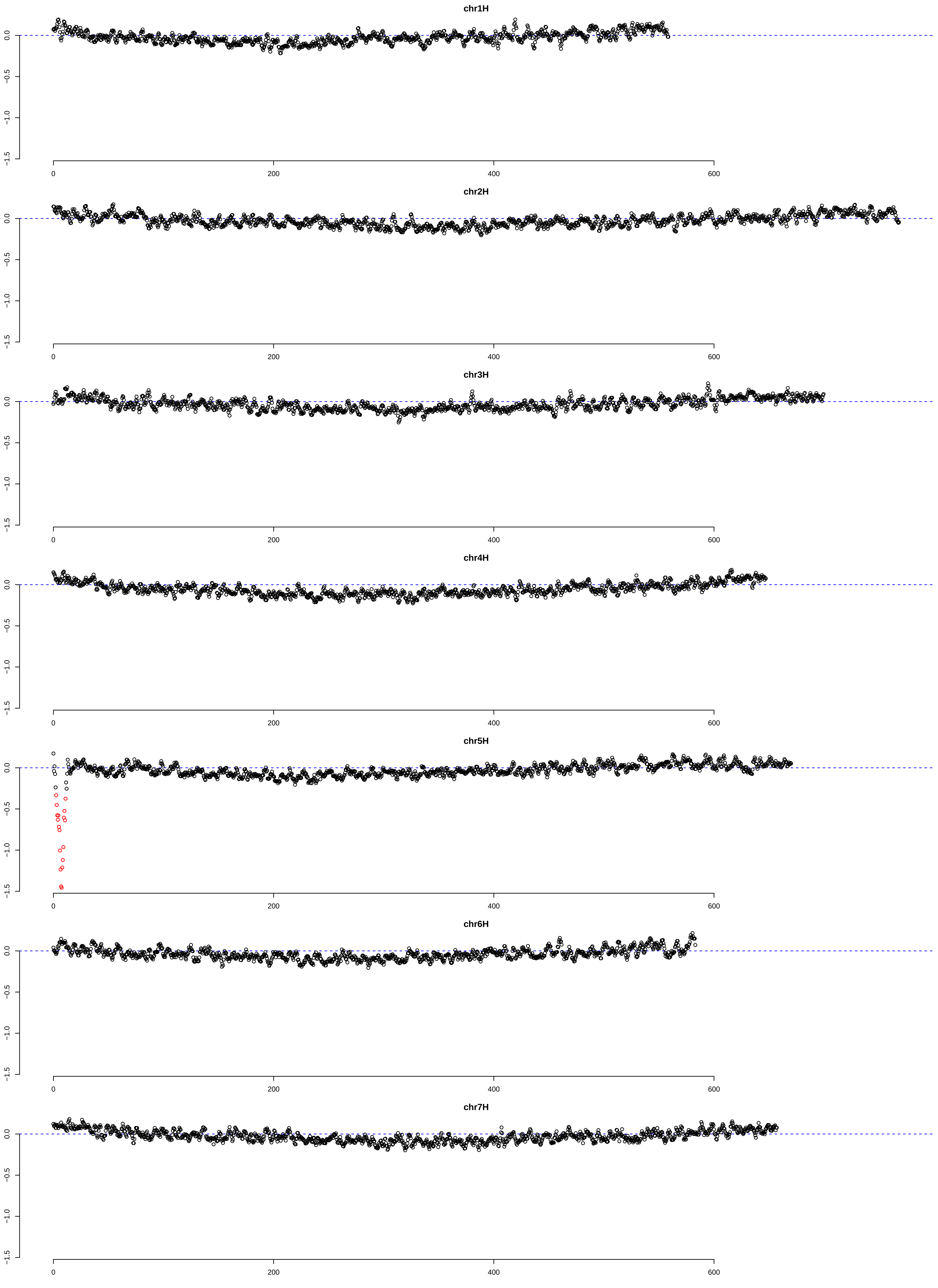

chr1H
chr2H
chr3H
chr4H
chr5H
chr6H
chr7H
0.0
-0.5
-1.0
-1.5
0
200
400
600

ERR699888

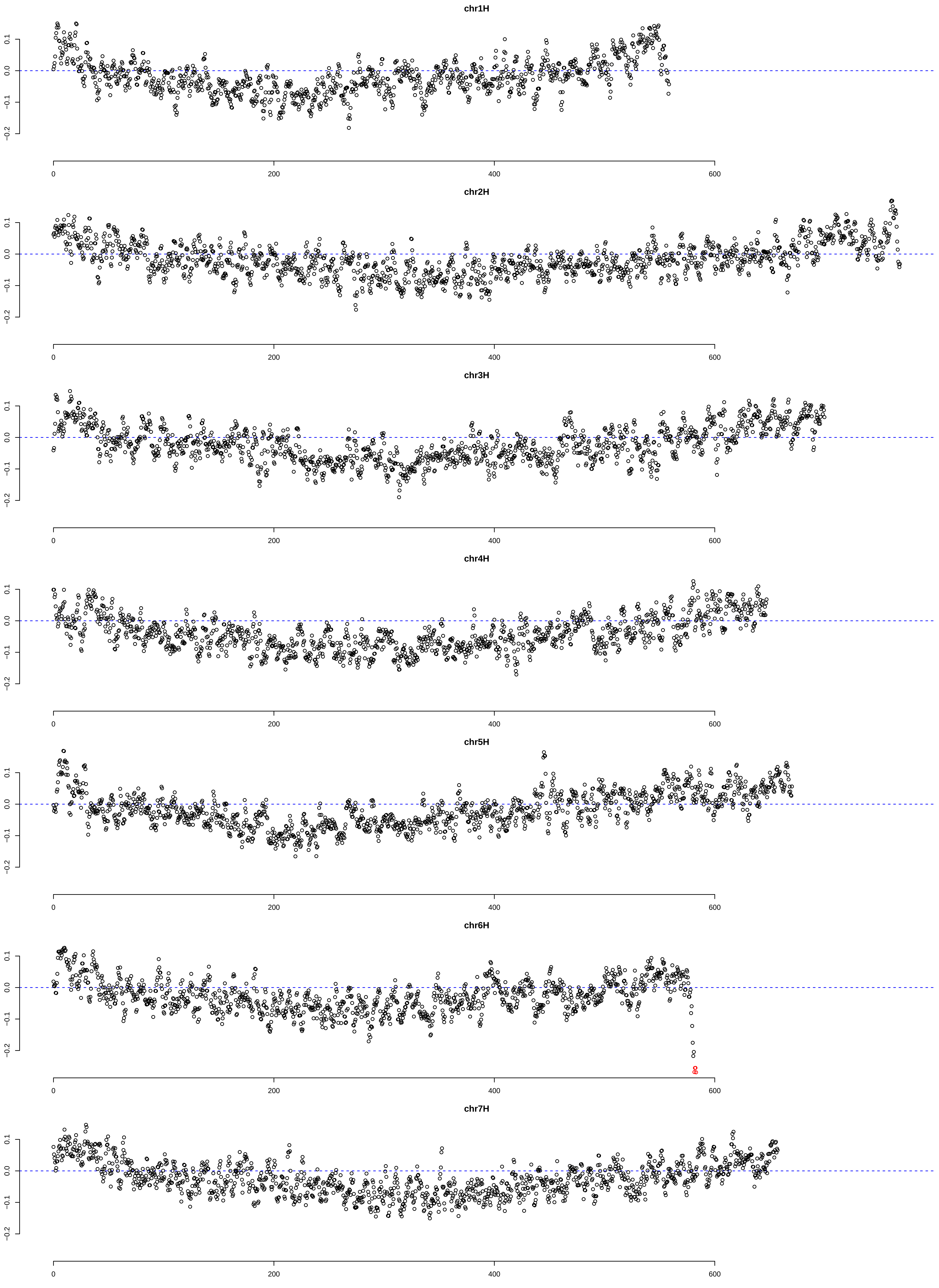
chr1H
chr2H
chr3H
chr4H
chr5H
chr6H
chr7H
0.1
0.0
-0.1
-0.2
0
200
400
600

ERR699889

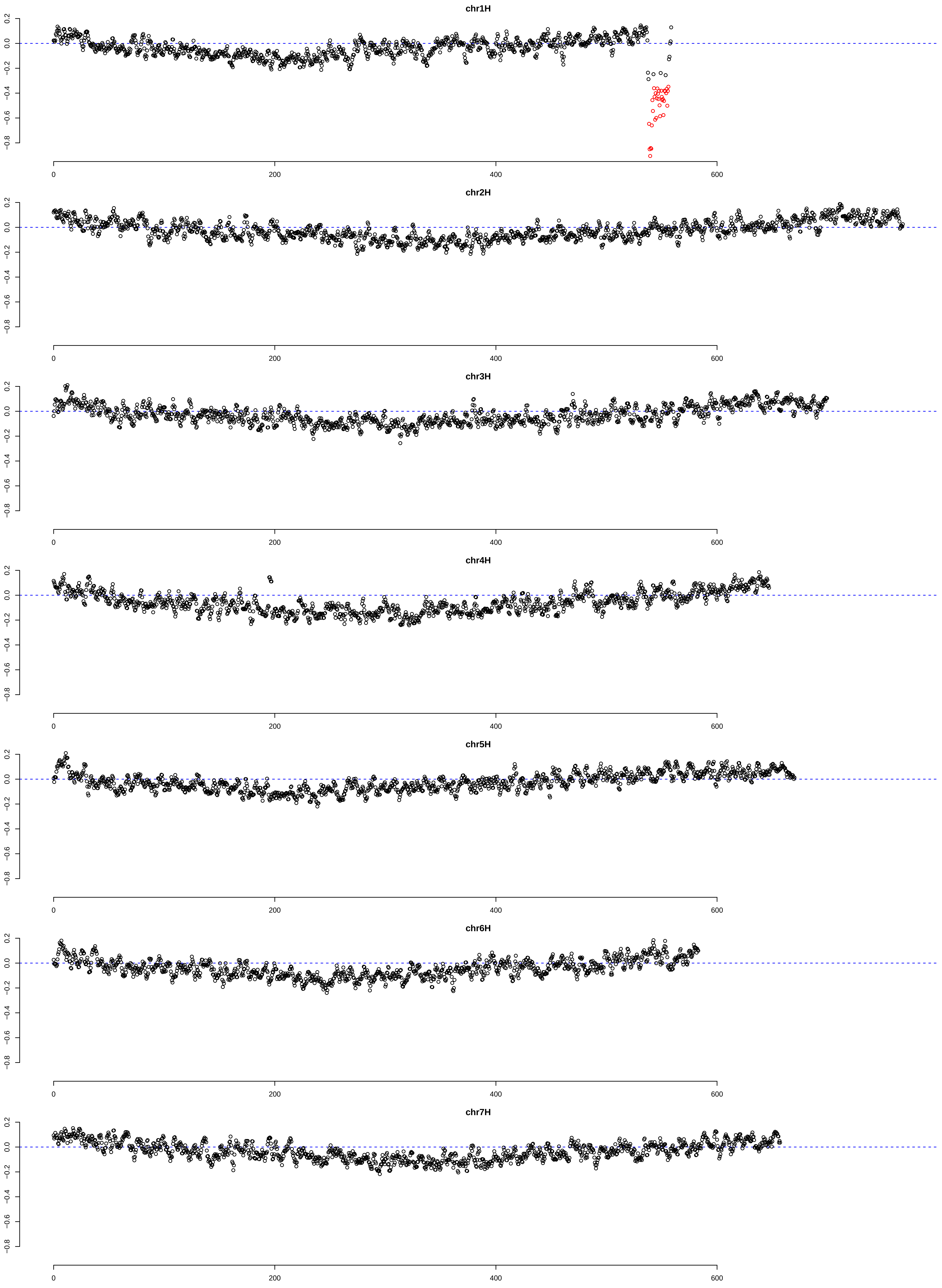
chr1H
chr2H
chr3H
chr4H
chr5H
chr6H
chr7H
0.2
0.0
-0.2
-0.4
-0.6
-0.8
0
200
400
600

ERR699890

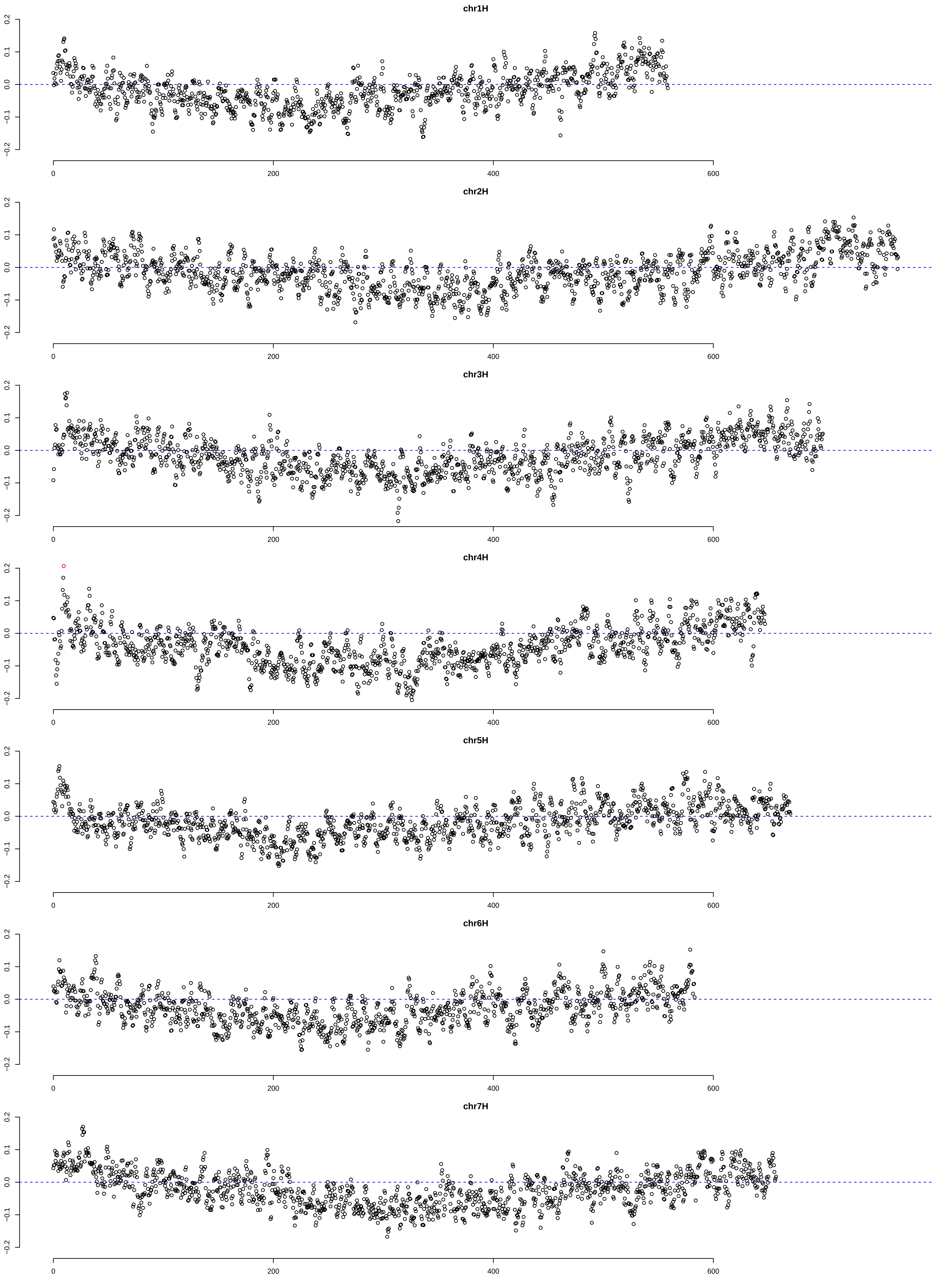

chr1H
chr2H
chr3H
chr4H
chr5H
chr6H
chr7H
-0.2
-0.1
0.0
0.1
0.2
0
200
400
600

ERR699891

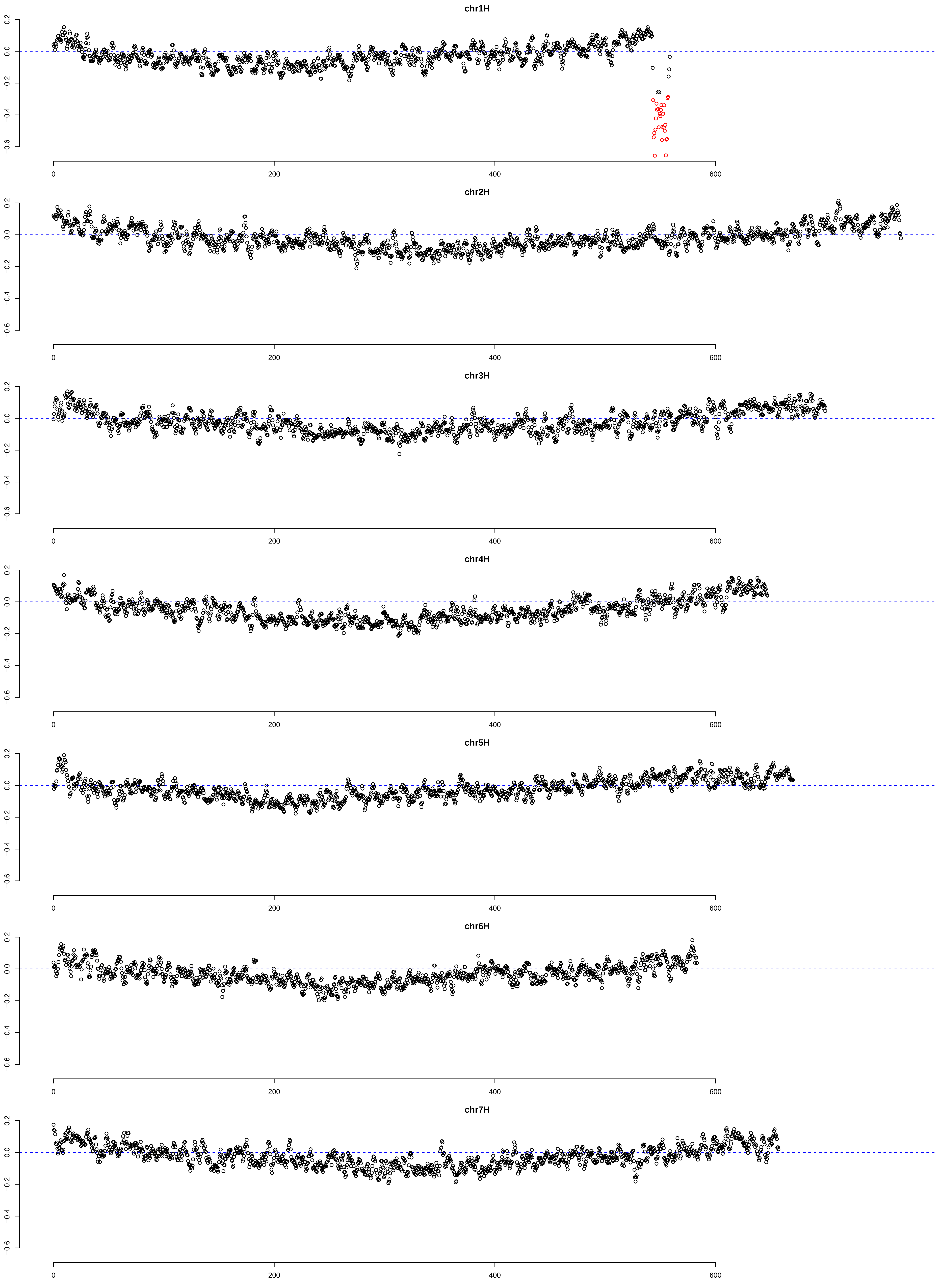
chr1H
chr2H
chr3H
chr4H
chr5H
chr6H
chr7H
0.2
0.0
-0.2
-0.4
-0.6
0
200
400
600

ERR699892

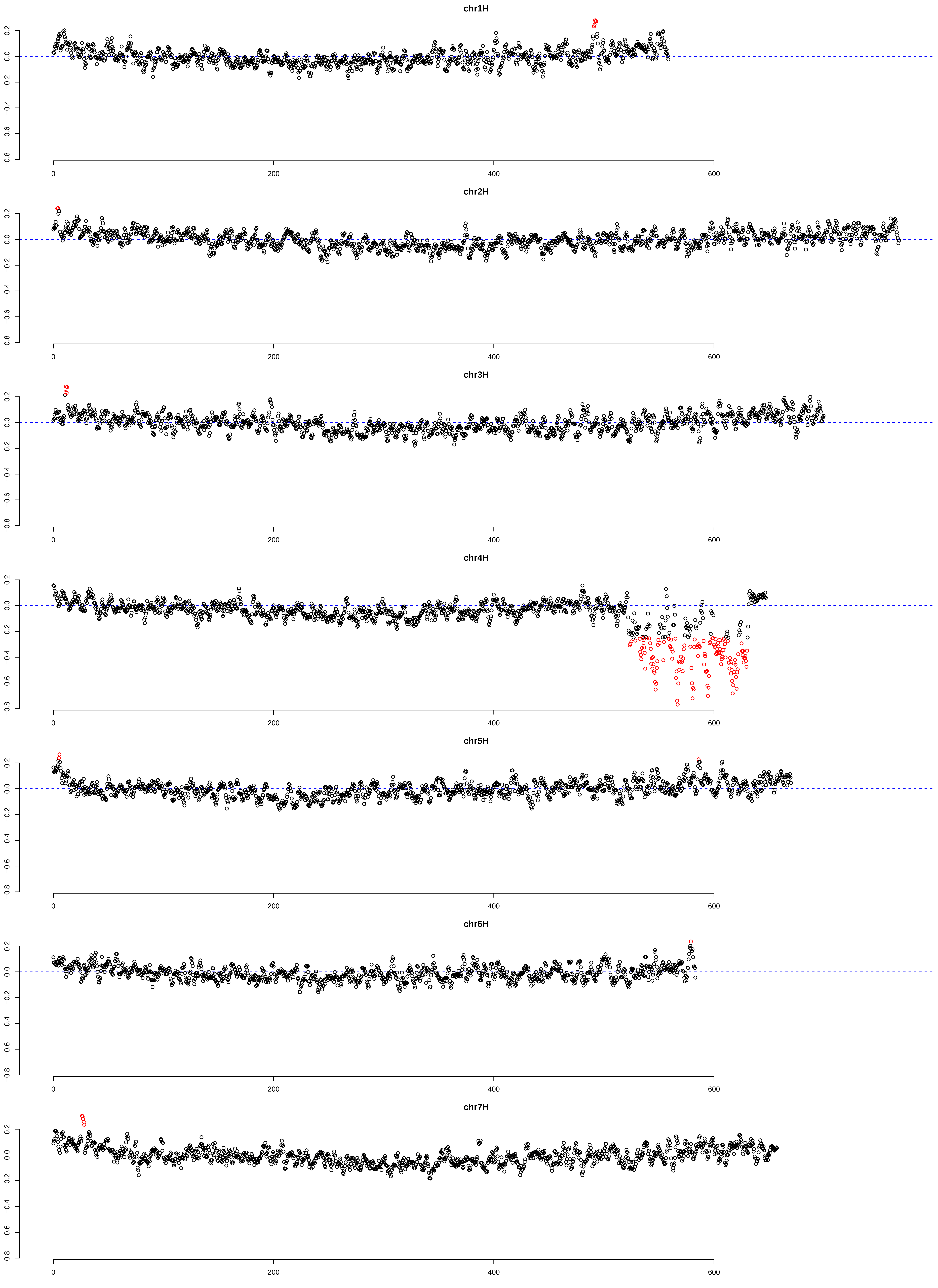

chr1H
chr2H
chr3H
chr4H
chr5H
chr6H
chr7H
0.2
0.0
-0.2
-0.4
-0.6
-0.8
0
200
400
600

ERR699893

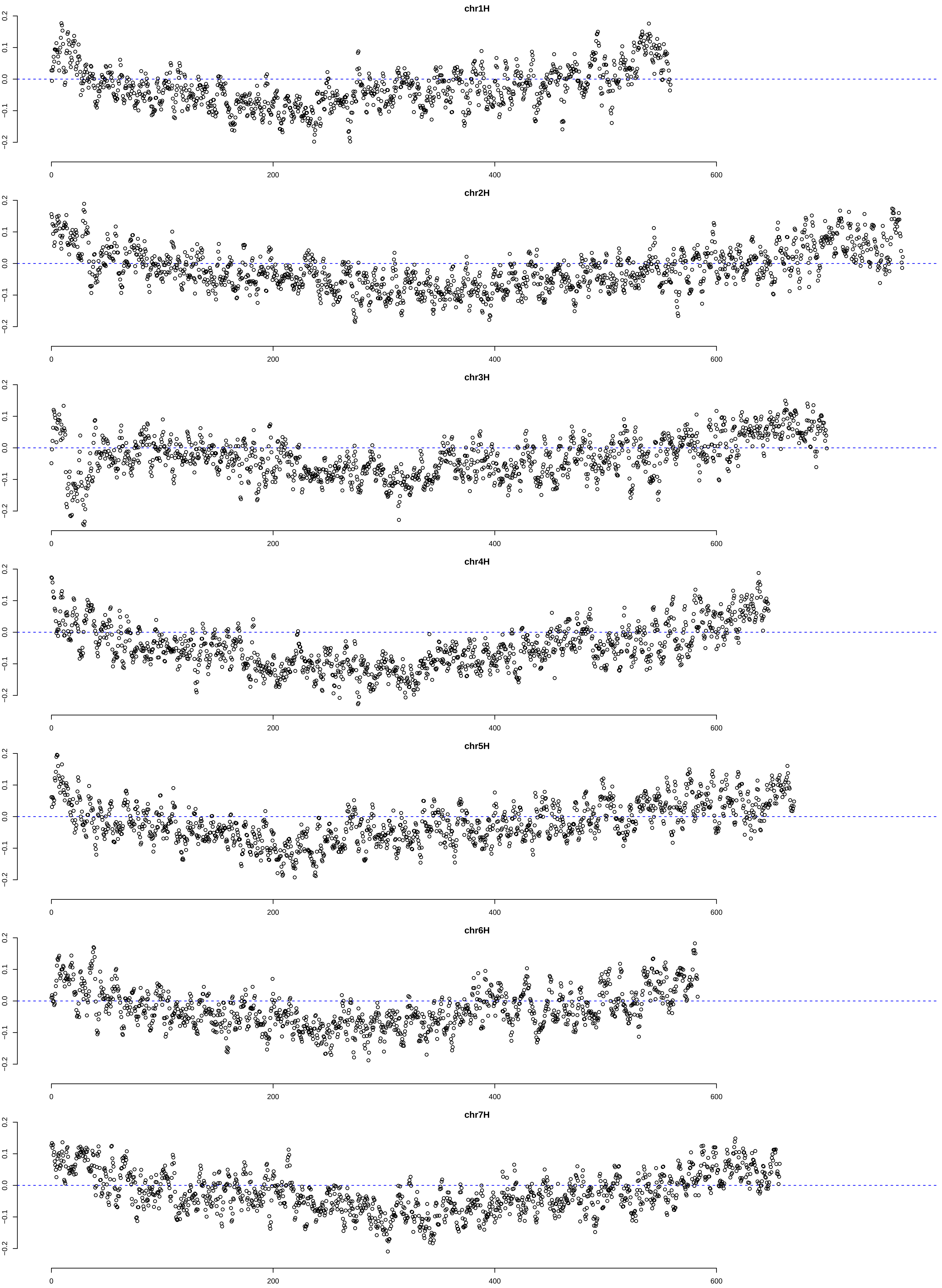
chr1H
chr2H
chr3H
chr4H
chr5H
chr6H
chr7H
0
200
400
600
-0.2
-0.1
0.0
0.1
0.2

ERR699894

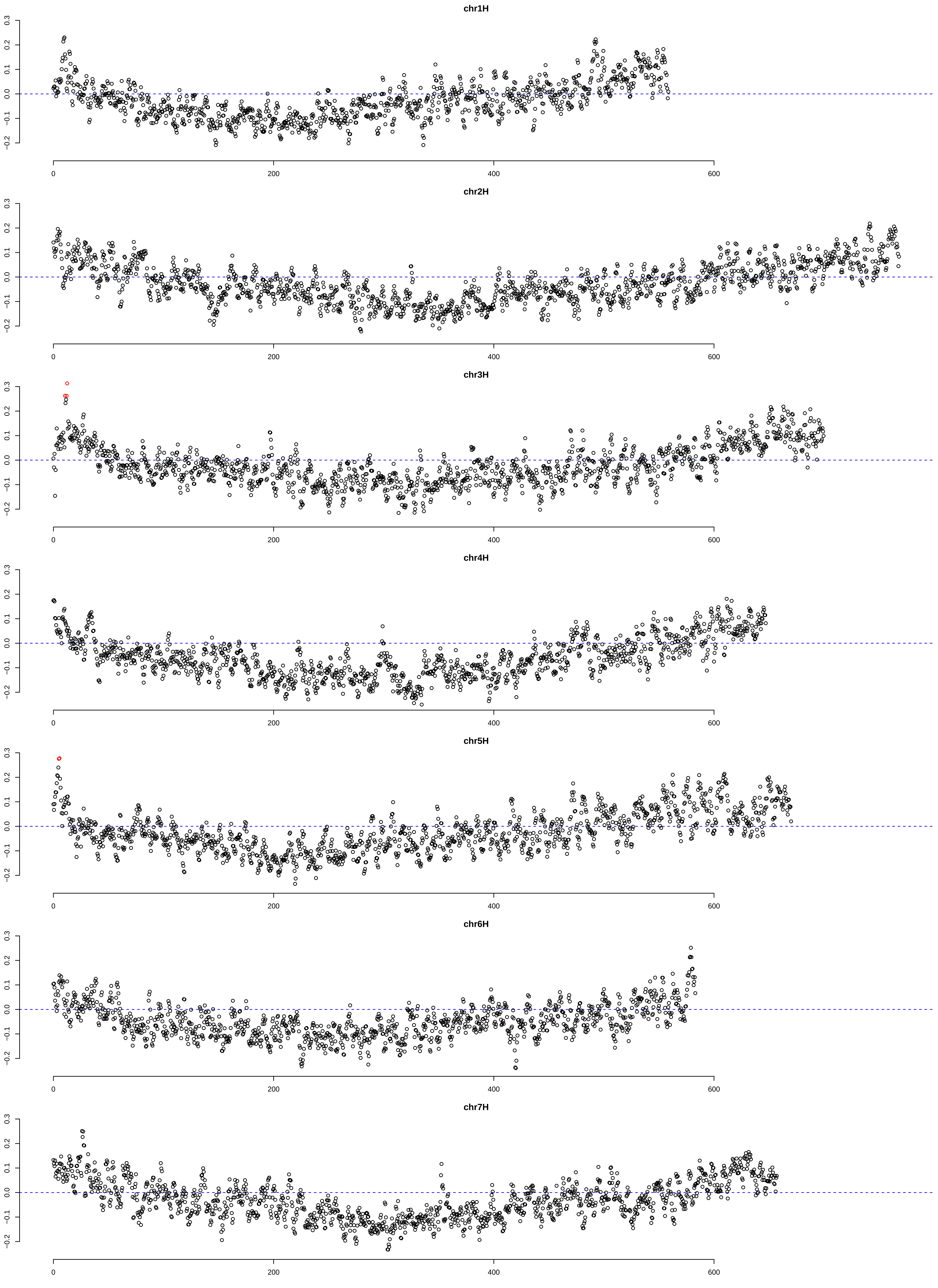
chr1H
chr2H
chr3H
chr4H
chr5H
chr6H
chr7H
-0.2
-0.1
0.0
0.1
0.2
0.3
0
200
400
600

ERR699895

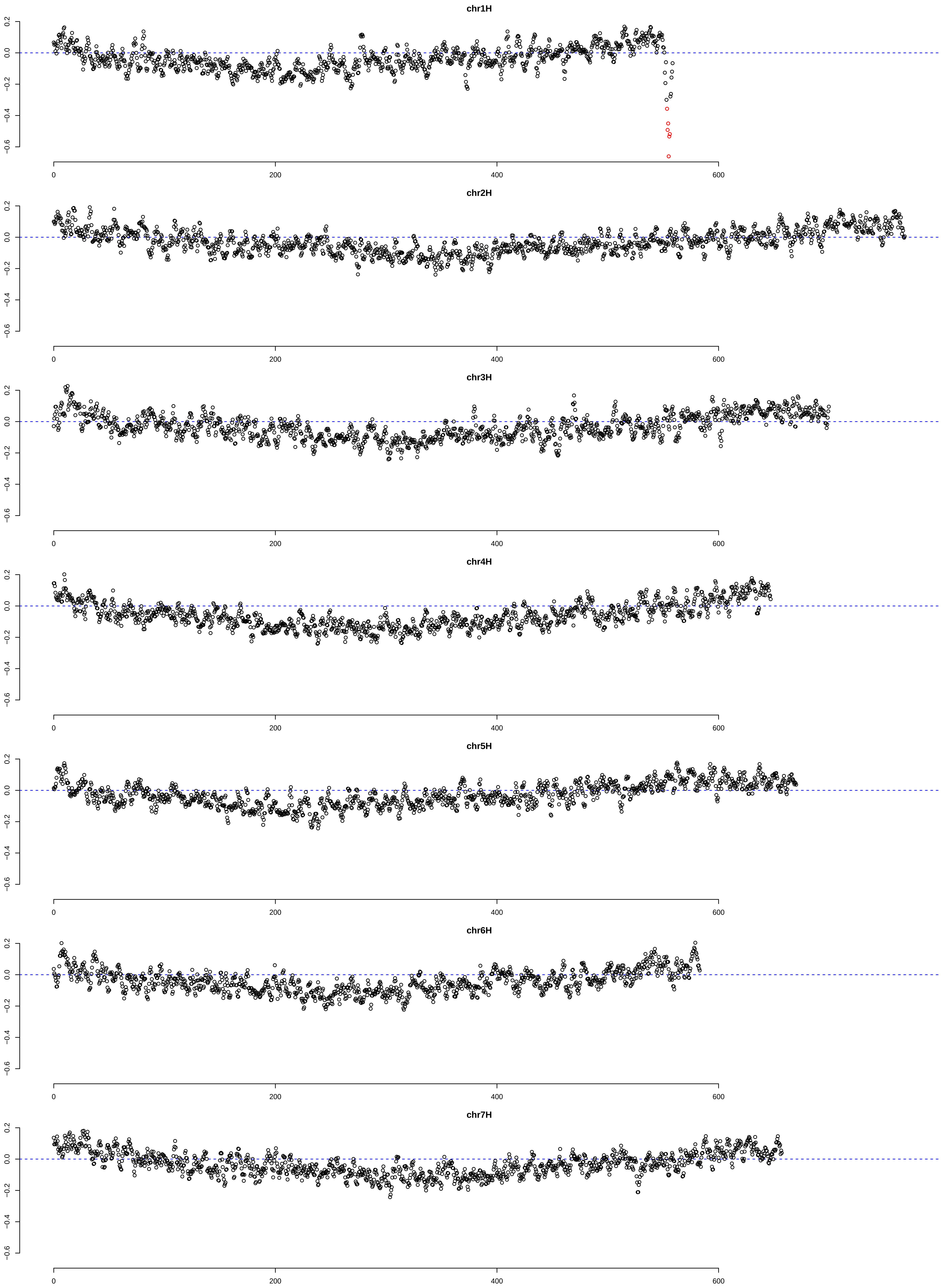
chr1H
chr2H
chr3H
chr4H
chr5H
chr6H
chr7H
0.2
0.0
-0.2
-0.4
-0.6
0
200
400
600

ERR699896

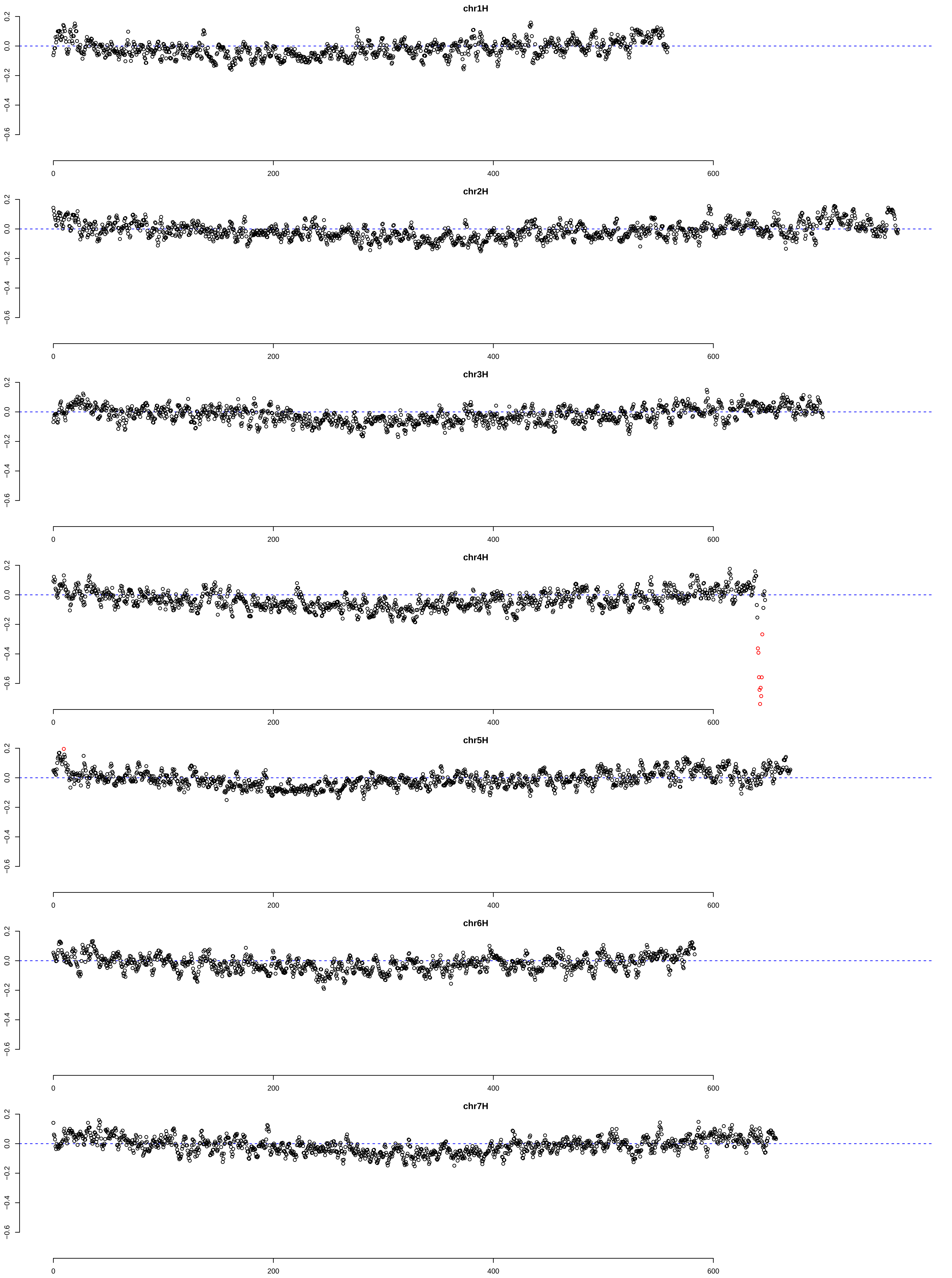
chr1H
chr2H
chr3H
chr4H
chr5H
chr6H
chr7H
0.2
0.0
-0.2
-0.4
-0.6
0
200
400
600

ERR699897

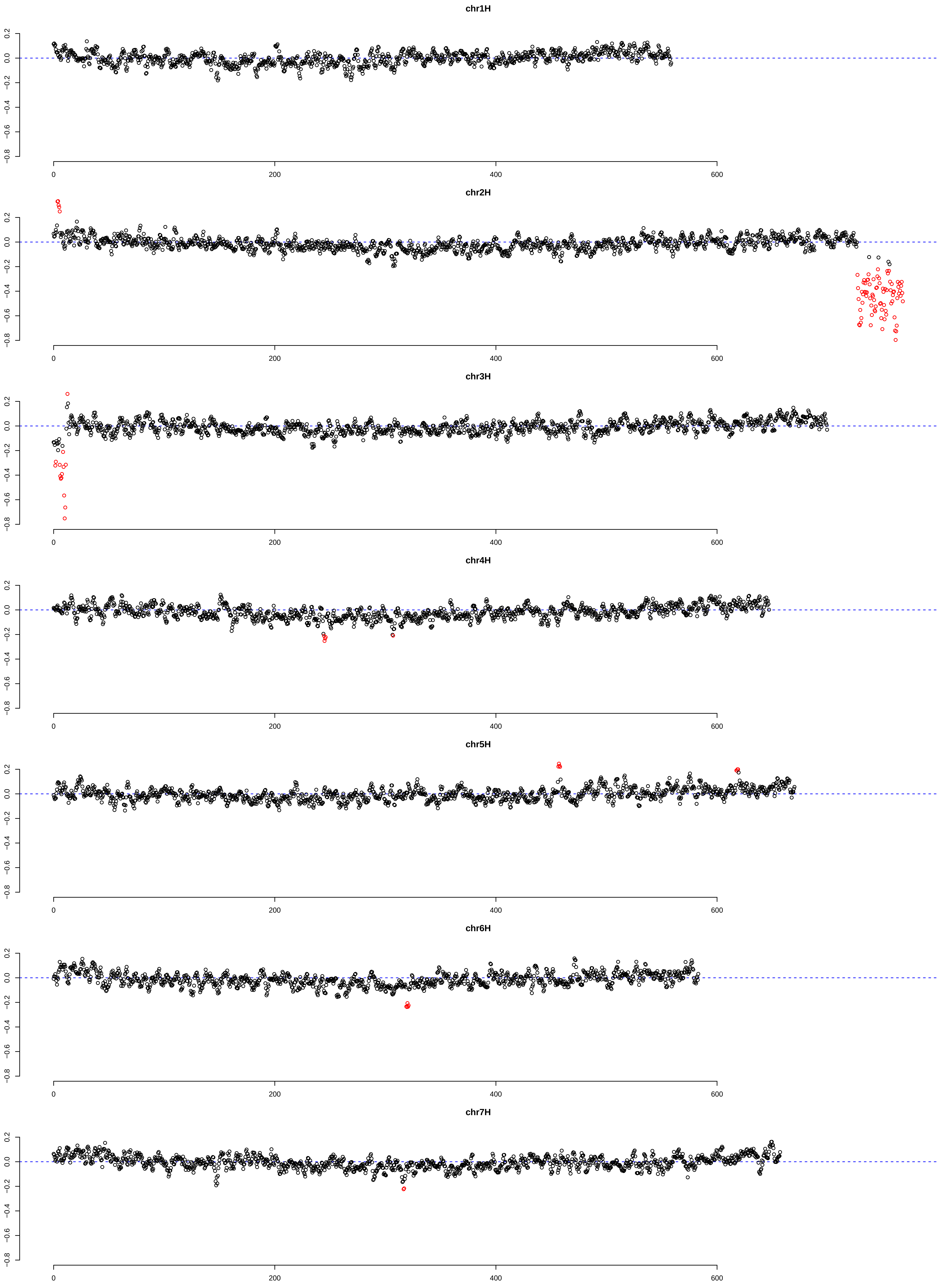
chr1H
chr2H
chr3H
chr4H
chr5H
chr6H
chr7H
0.2
0.0
-0.2
-0.4
-0.6
-0.8
0
200
400
600

ERR699898

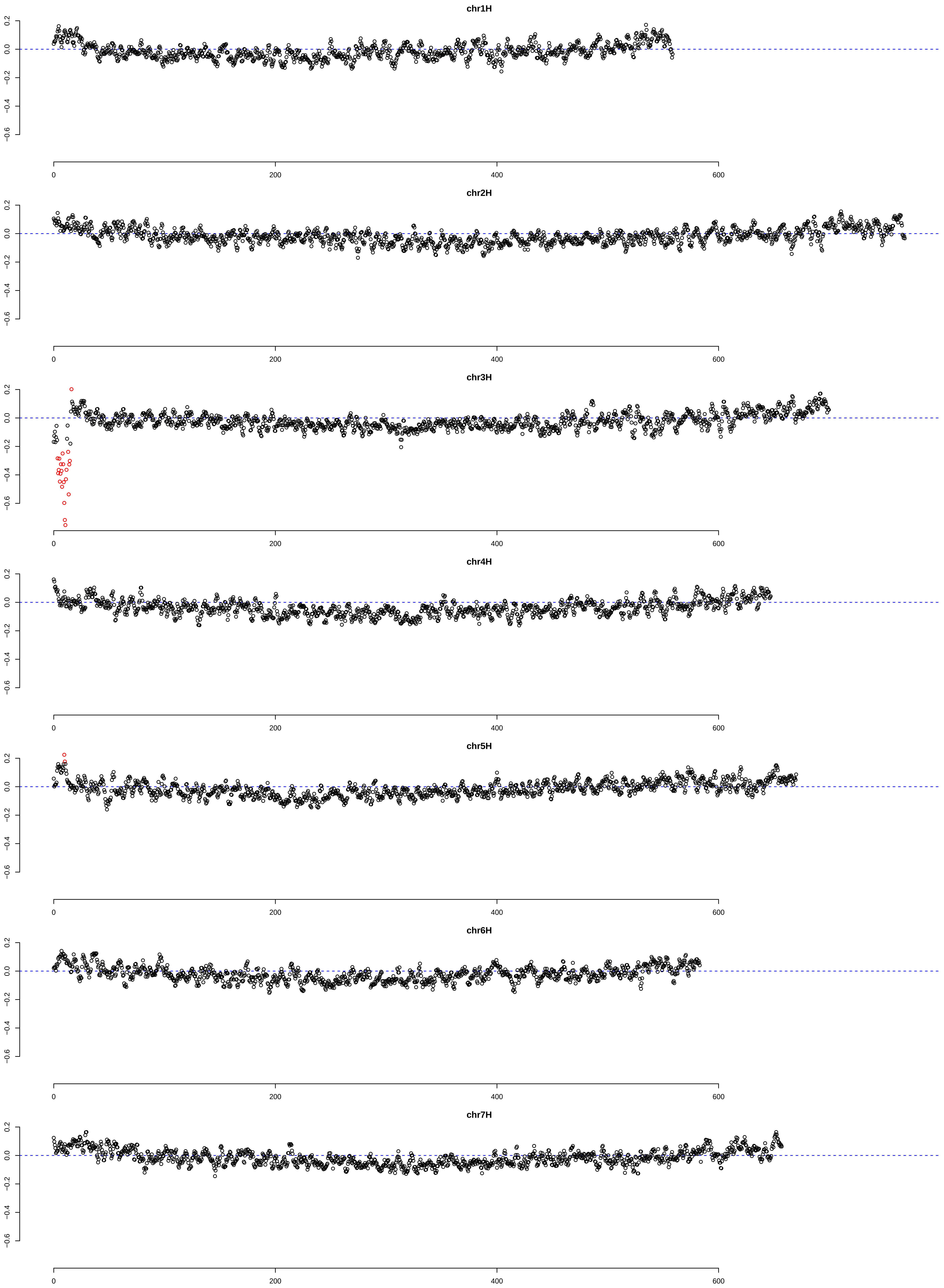
chr1H
chr2H
chr3H
chr4H
chr5H
chr6H
chr7H
0.2
0.0
-0.2
-0.4
-0.6
0
200
400
600

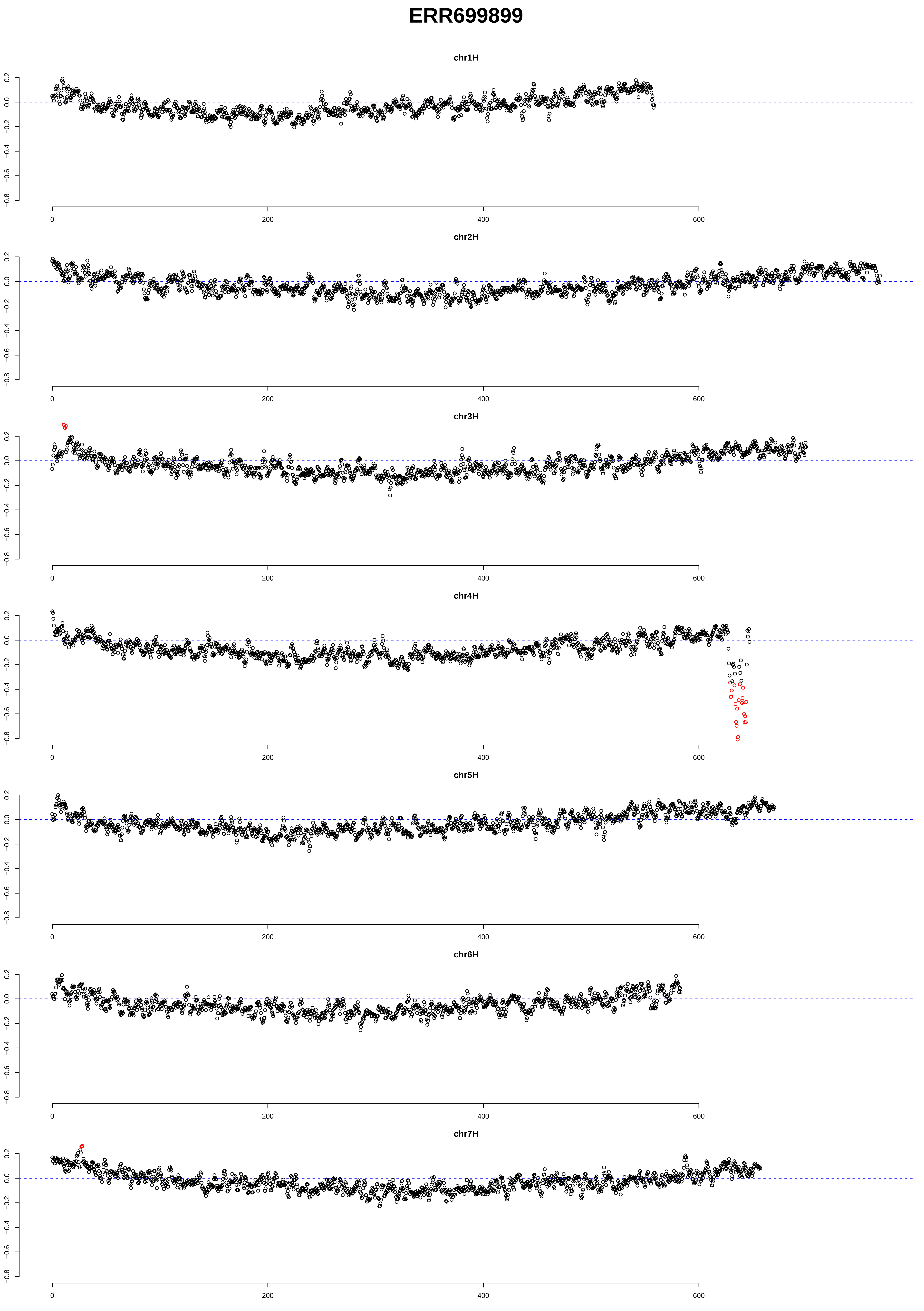
ERR699899
chr1H
chr2H
chr3H
chr4H
chr5H
chr6H
chr7H
0.2
0.0
-0.2
-0.4
-0.6
-0.8
0
200
400
600

ERR699900

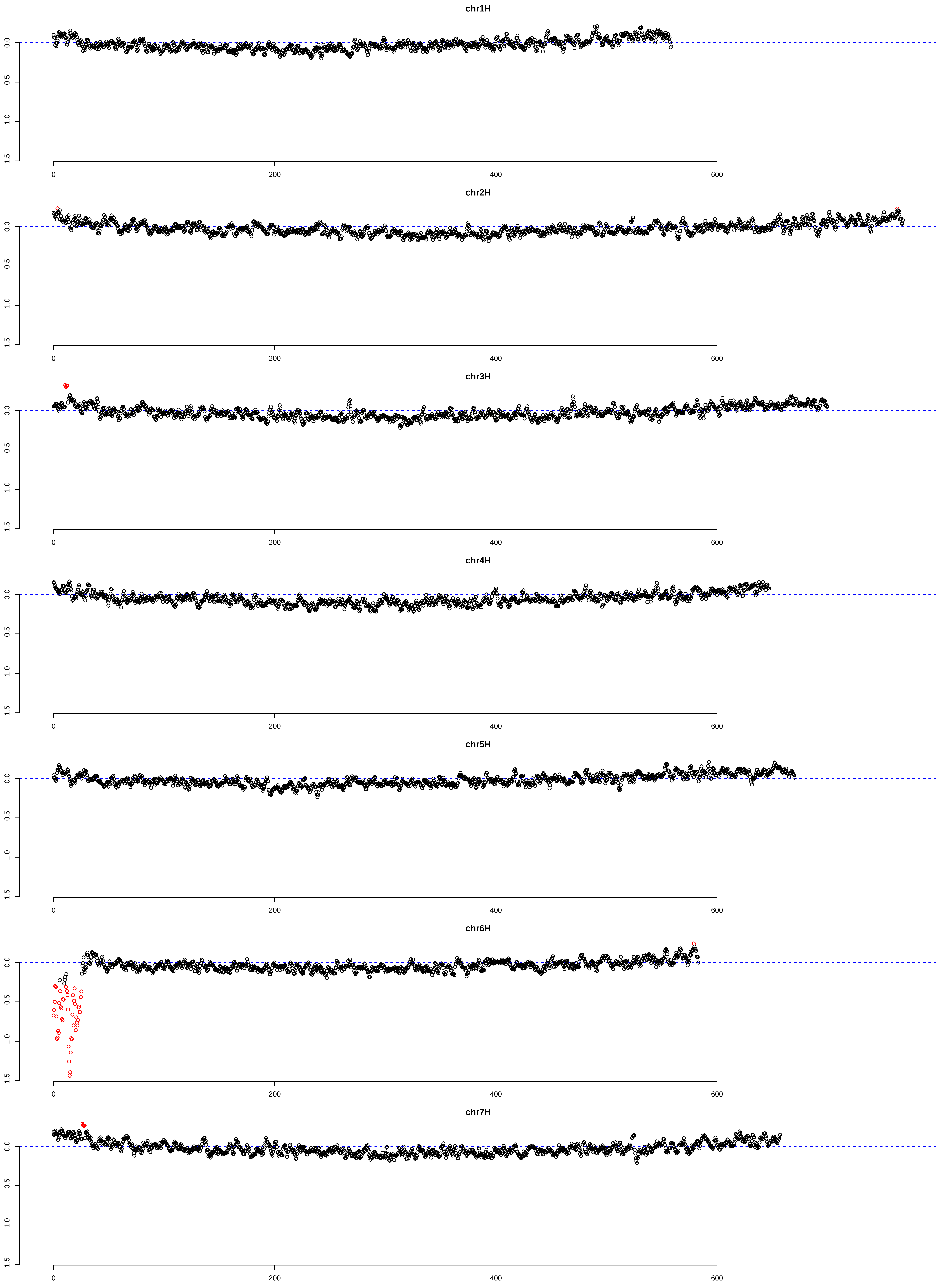
chr1H
chr2H
chr3H
chr4H
chr5H
chr6H
chr7H
0.0
-0.5
-1.0
-1.5
0
200
400
600

ERR699901

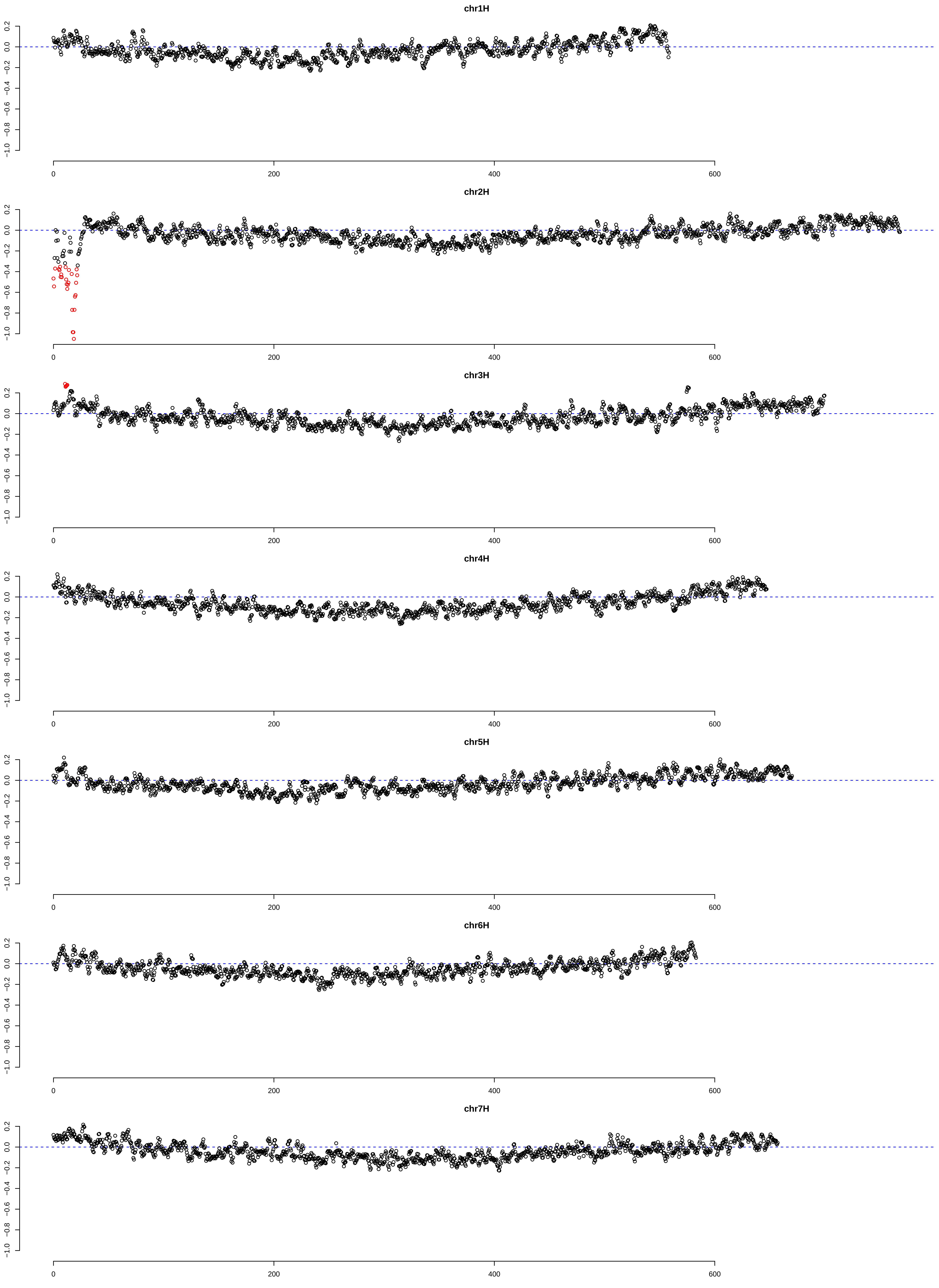
chr1H
chr2H
chr3H
chr4H
chr5H
chr6H
chr7H
0.2
0.0
−0.2
−0.4
−0.6
−0.8
−1.0
0
200
400
600

ERR699902

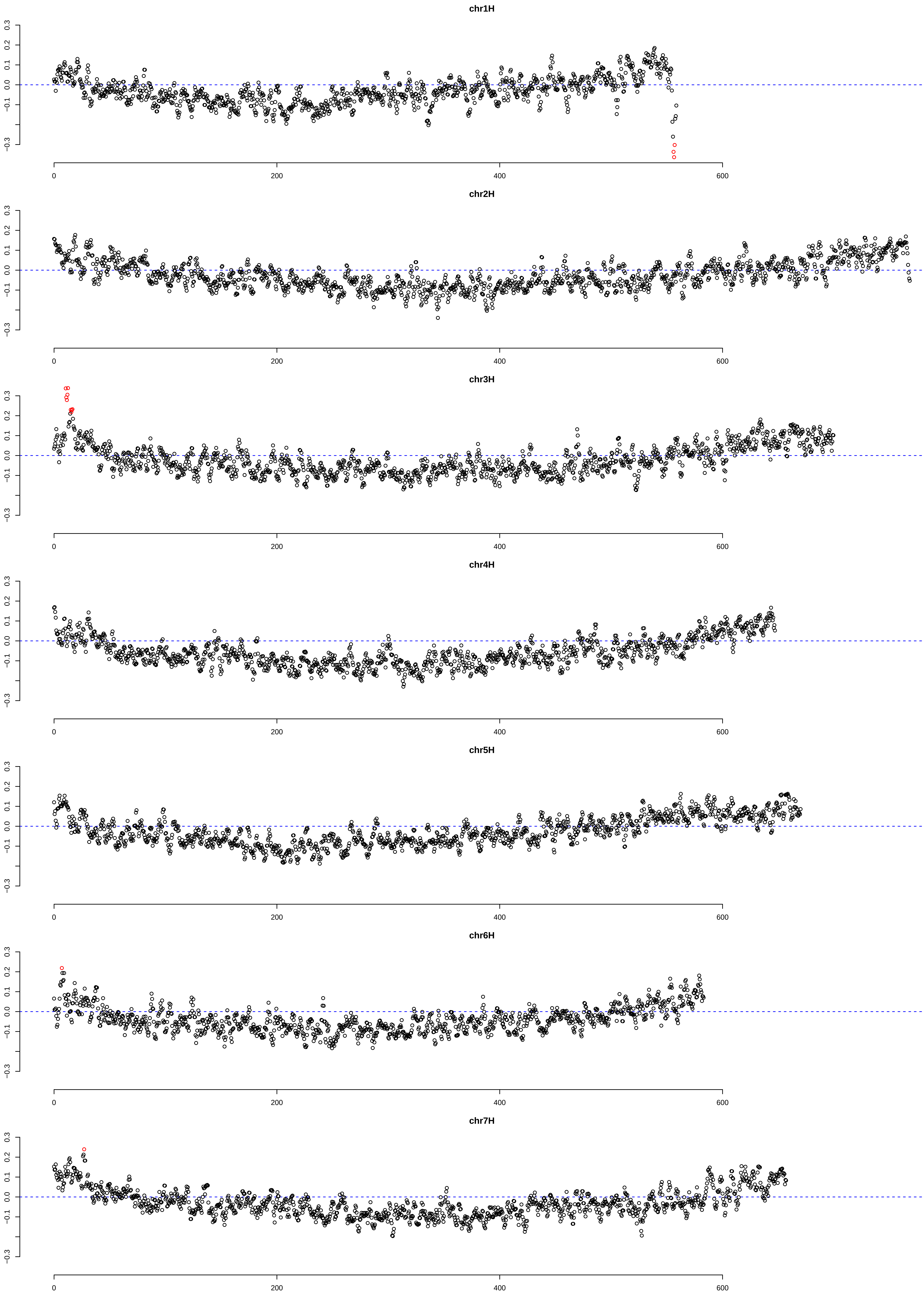
chr1H
chr2H
chr3H
chr4H
chr5H
chr6H
chr7H
0
200
400
600
-0.3
-0.1
0.0
0.1
0.2
0.3

ERR699903

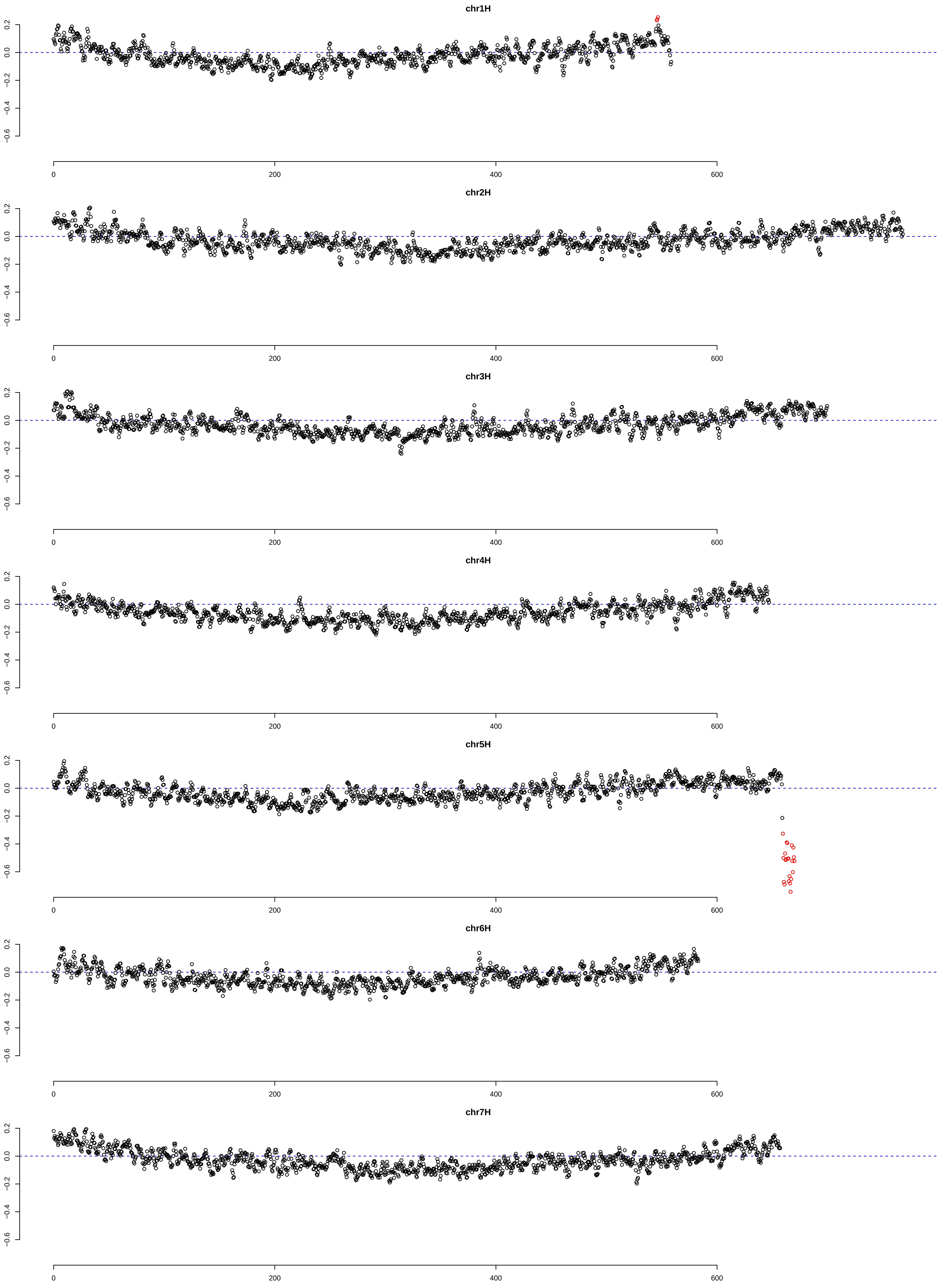
chr1H
chr2H
chr3H
chr4H
chr5H
chr6H
chr7H
0.2
0.0
-0.2
-0.4
-0.6
0
200
400
600

ERR699904

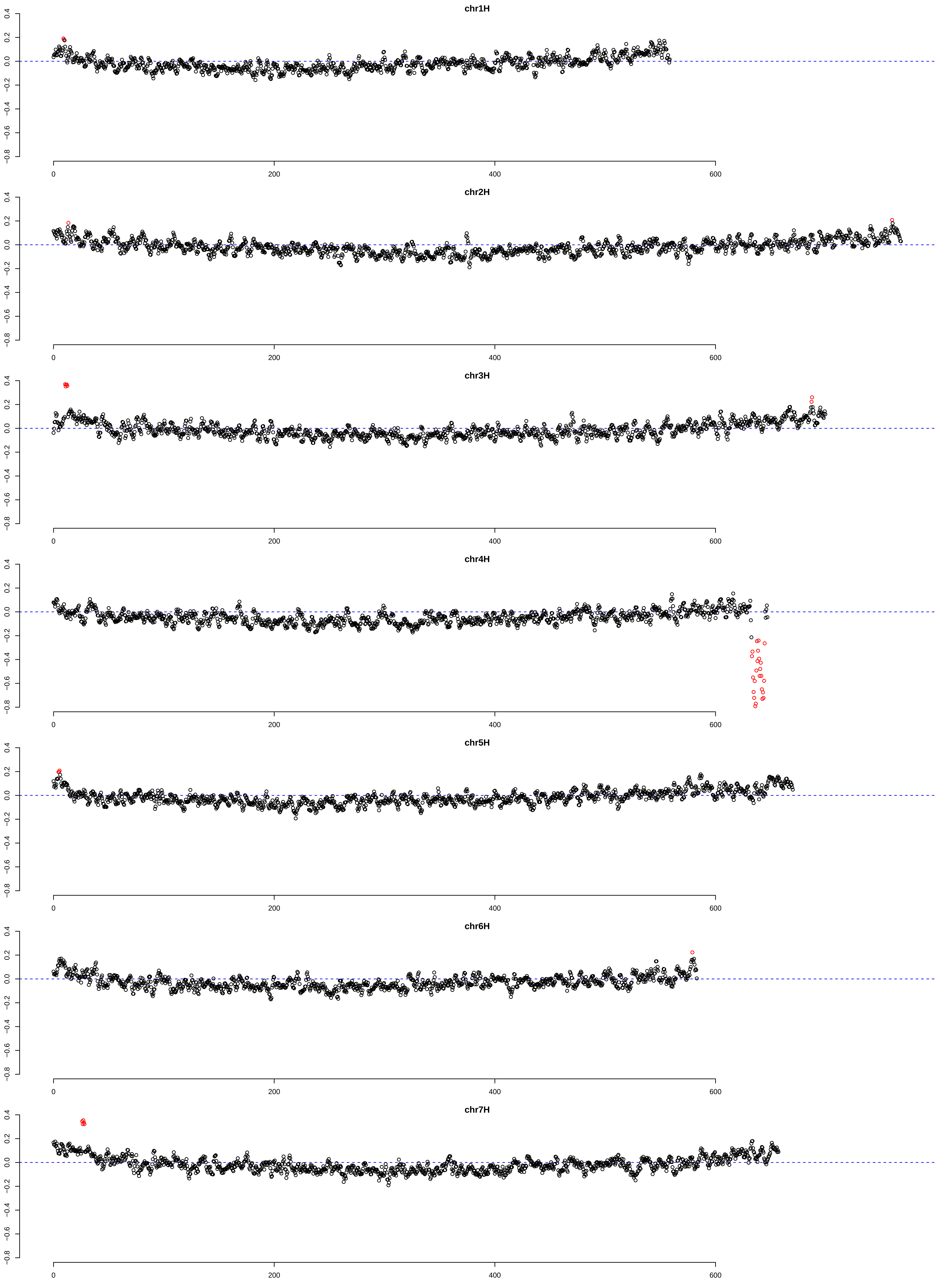

chr1H
chr2H
chr3H
chr4H
chr5H
chr6H
chr7H
0.4
0.2
0.0
-0.2
-0.4
-0.6
-0.8
0
200
400
600

ERR699905

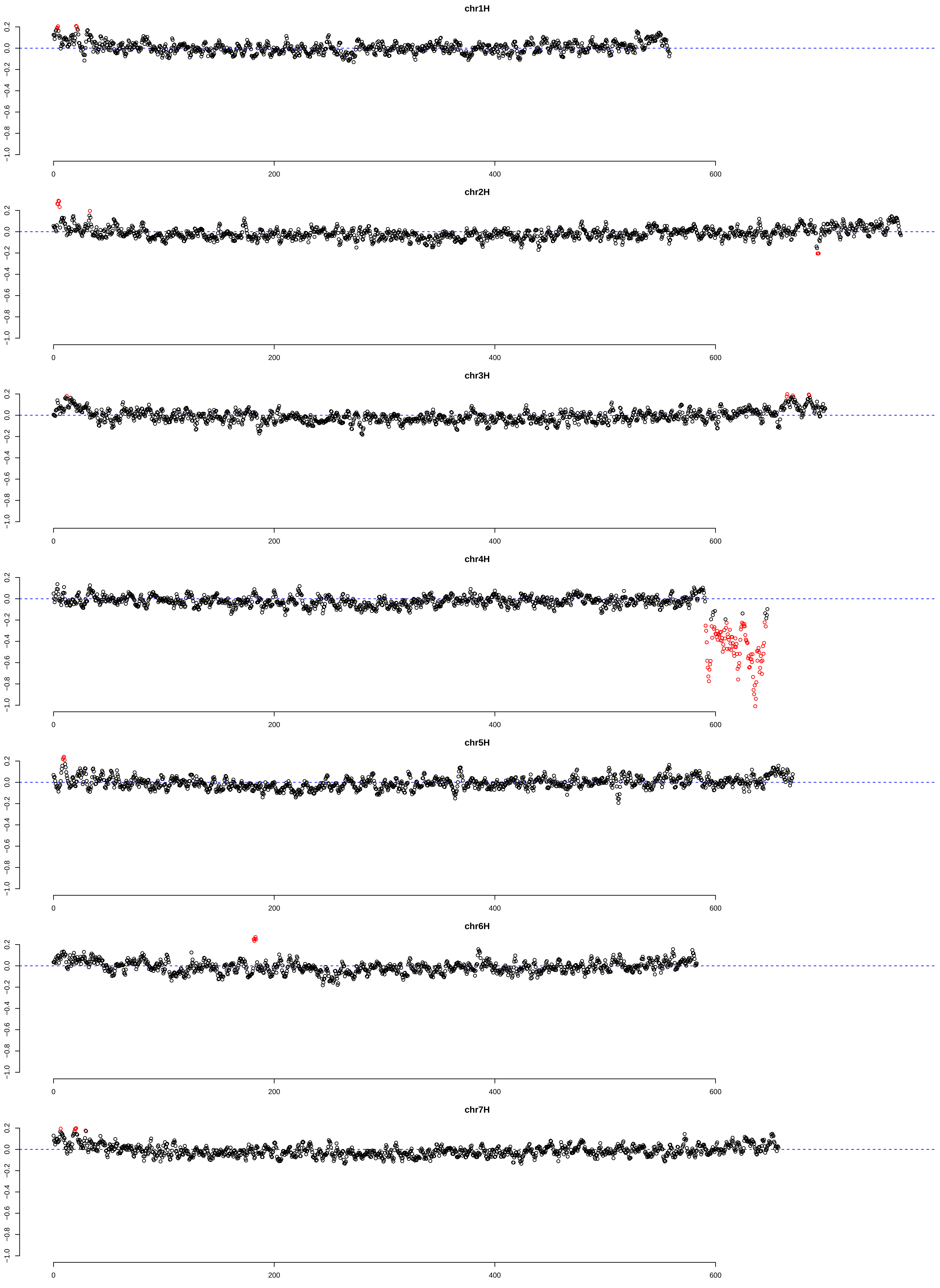
chr1H
chr2H
chr3H
chr4H
chr5H
chr6H
chr7H
0.2
0.0
−0.2
−0.4
−0.6
−0.8
−1.0
0
200
400
600

ERR699906

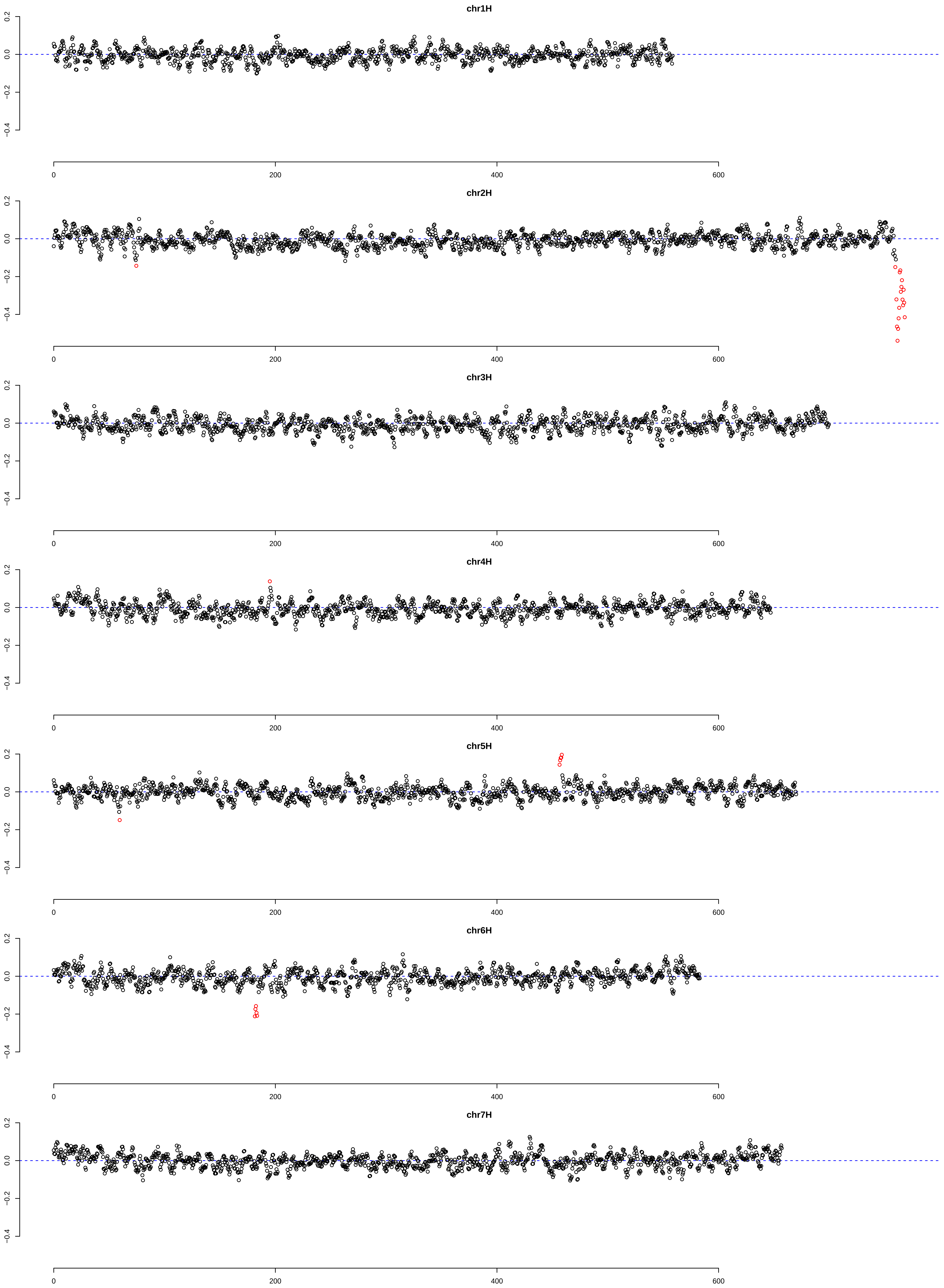
chr1H
chr2H
chr3H
chr4H
chr5H
chr6H
chr7H
0.2
0.0
-0.2
-0.4
0
200
400
600

ERR699908

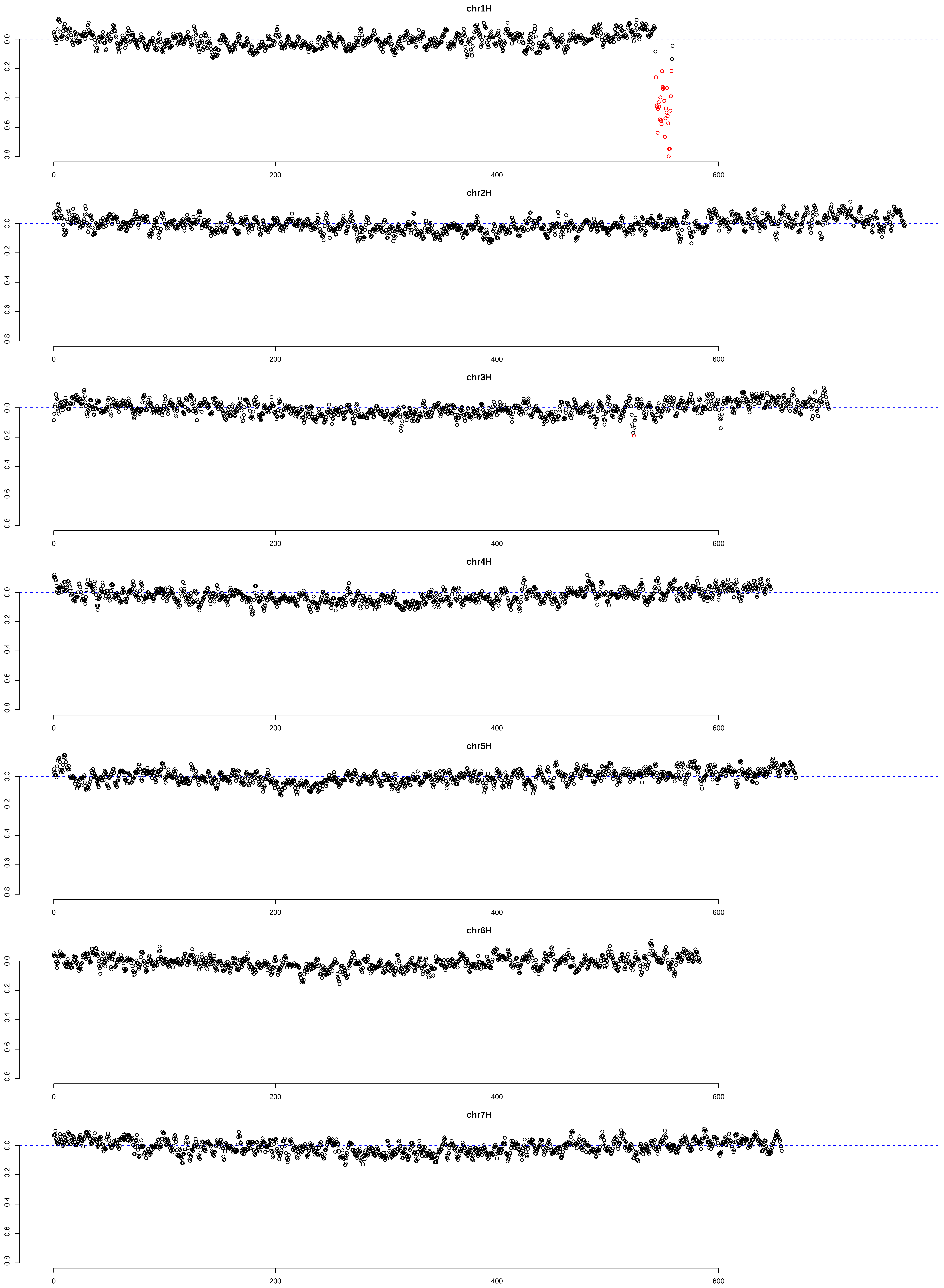

chr1H
chr2H
chr3H
chr4H
chr5H
chr6H
chr7H
0.0
−0.2
−0.4
−0.6
−0.8
0
200
400
600

ERR699909

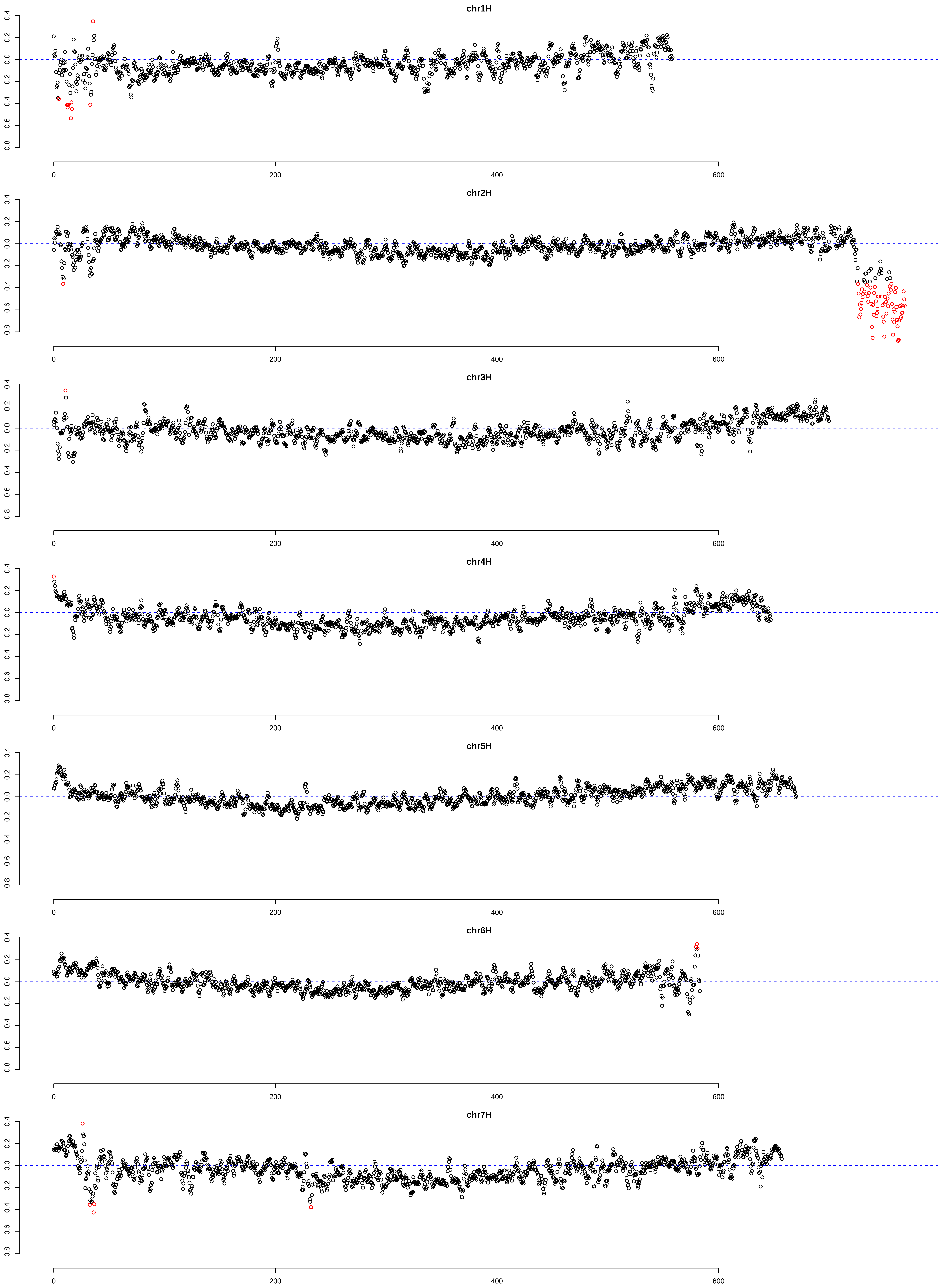
chr1H
chr2H
chr3H
chr4H
chr5H
chr6H
chr7H
0.4
0.2
0.0
−0.2
−0.4
−0.6
−0.8
0
200
400
600

ERR699910

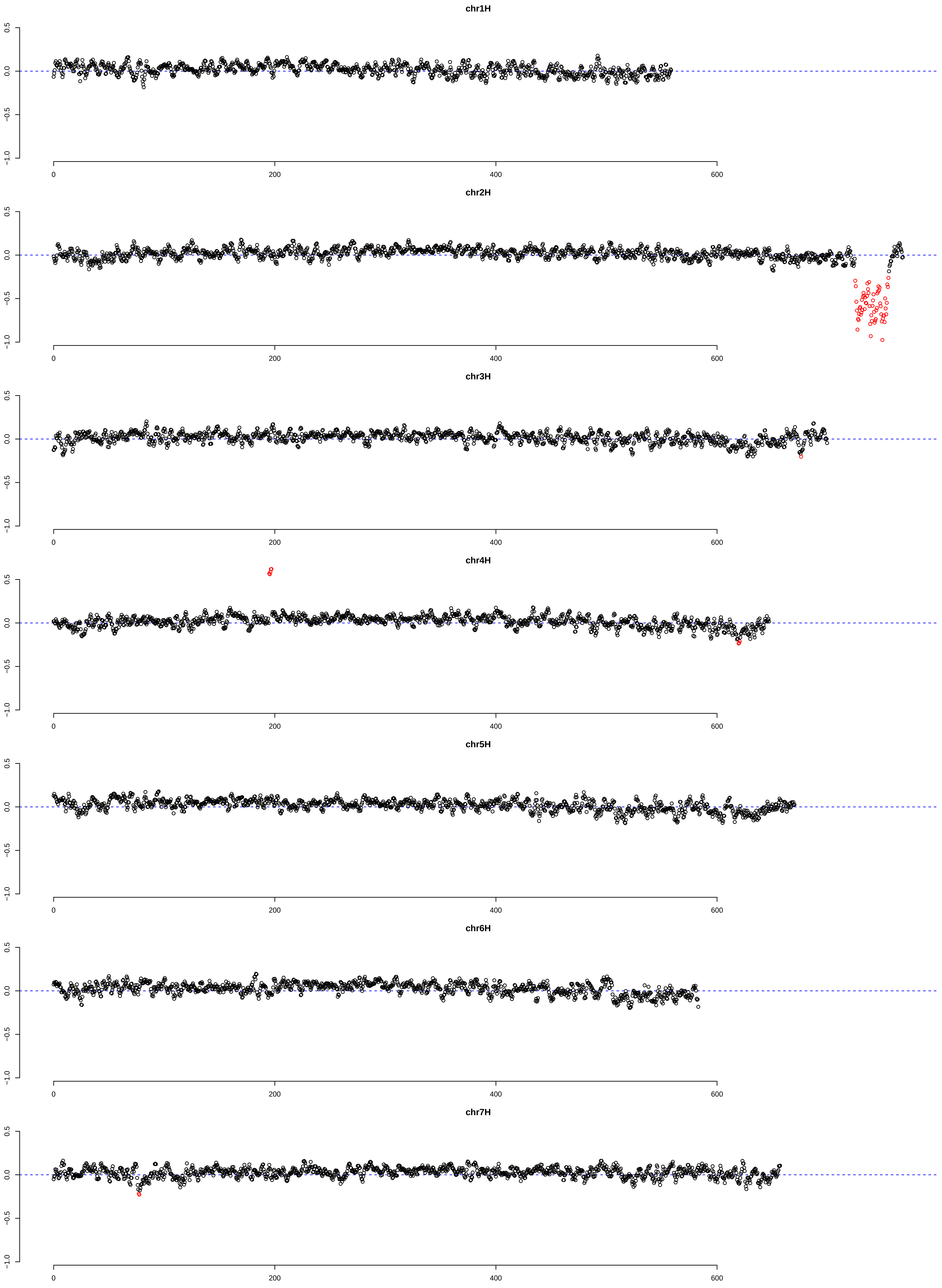

chr1H
chr2H
chr3H
chr4H
chr5H
chr6H
chr7H
0.5
0.0
−0.5
−1.0
0
200
400
600

ERR699912

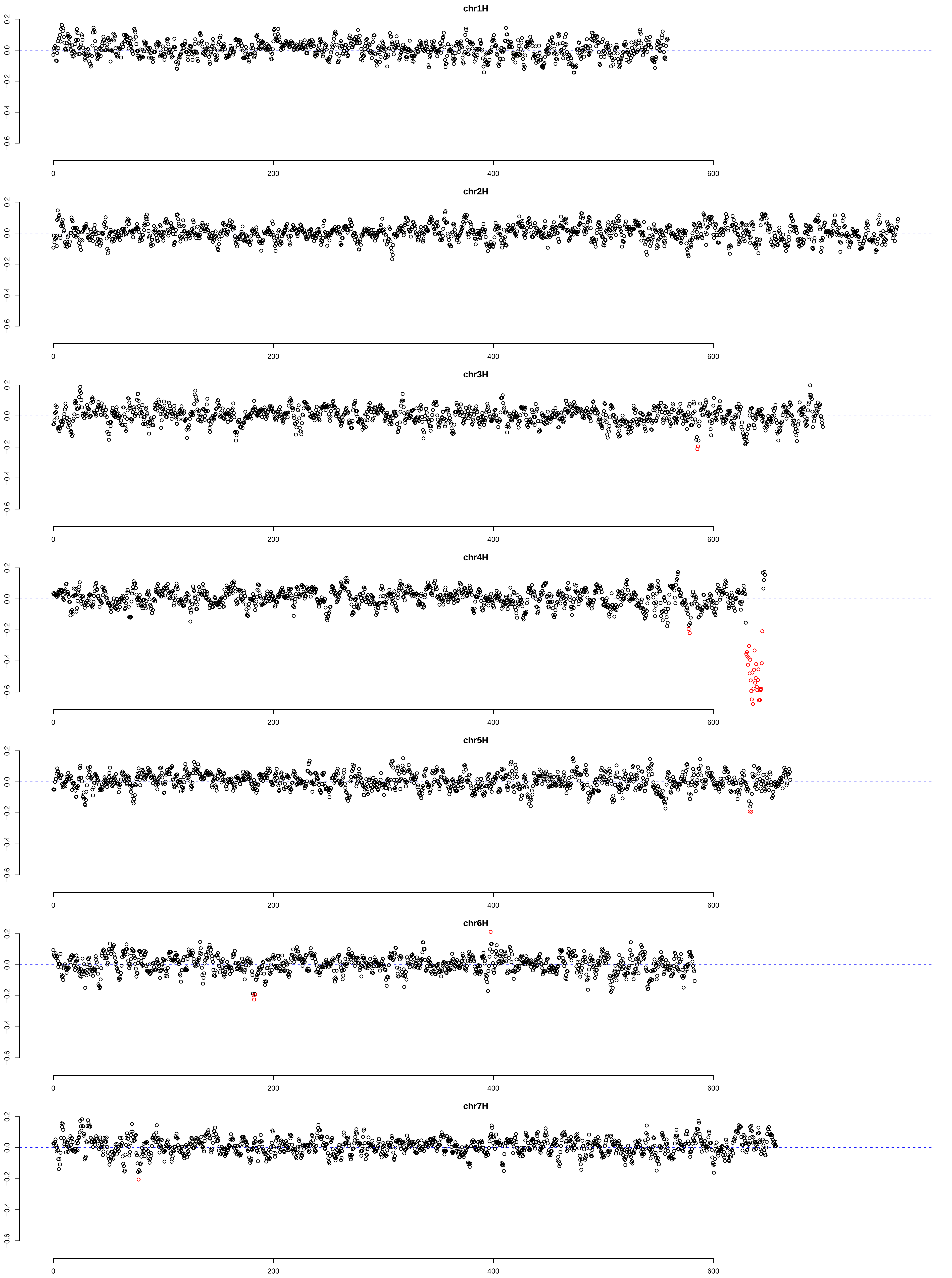
chr1H
chr2H
chr3H
chr4H
chr5H
chr6H
chr7H
0.2
0.0
-0.2
-0.4
-0.6
0
200
400
600

ERR699913

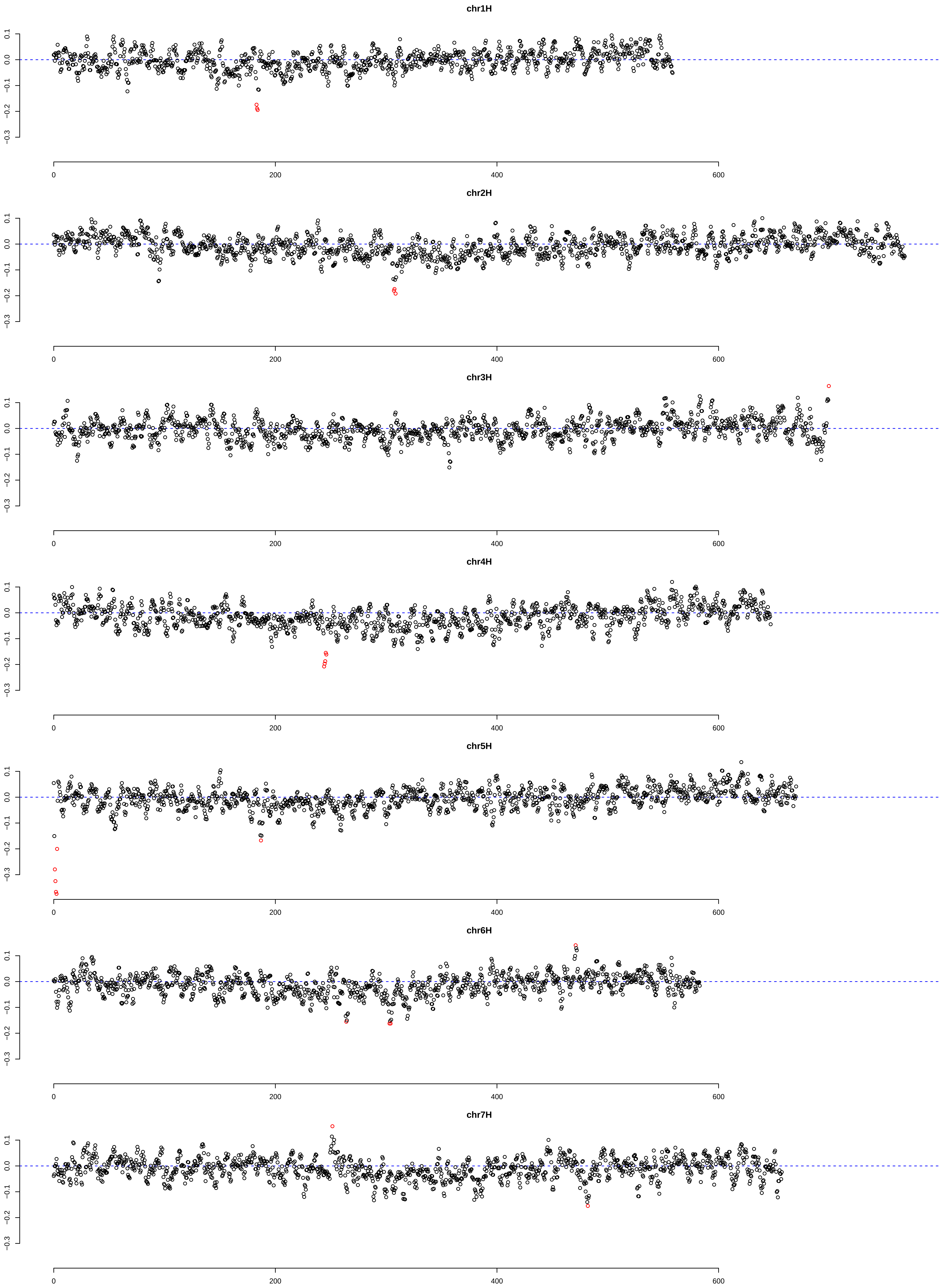
chr1H
chr2H
chr3H
chr4H
chr5H
chr6H
chr7H
0.1
0.0
-0.1
-0.2
-0.3
0
200
400
600

ERR699914

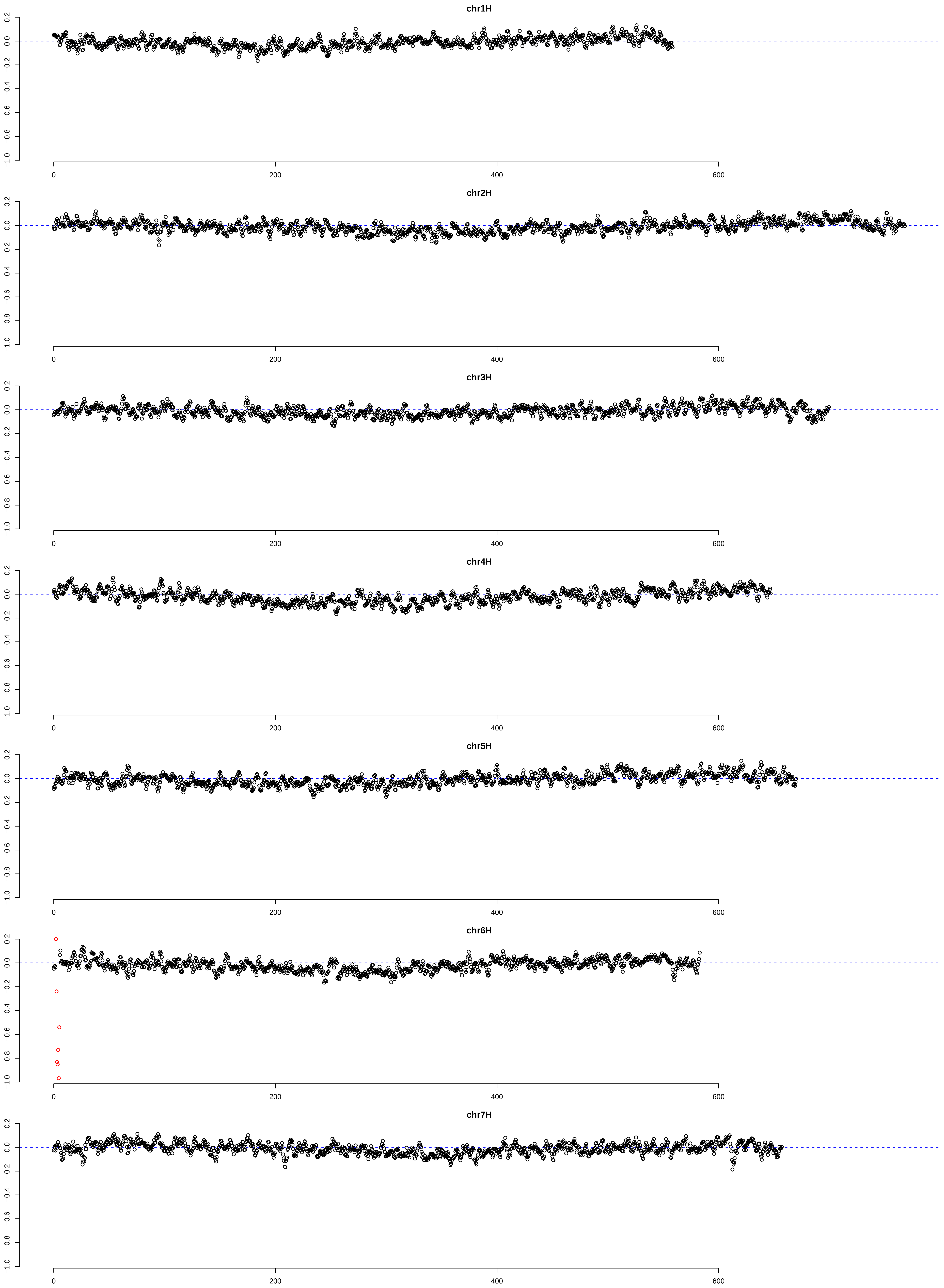
chr1H
chr2H
chr3H
chr4H
chr5H
chr6H
chr7H
0.2
0.0
-0.2
-0.4
-0.6
-0.8
-1.0
0
200
400
600

ERR699915

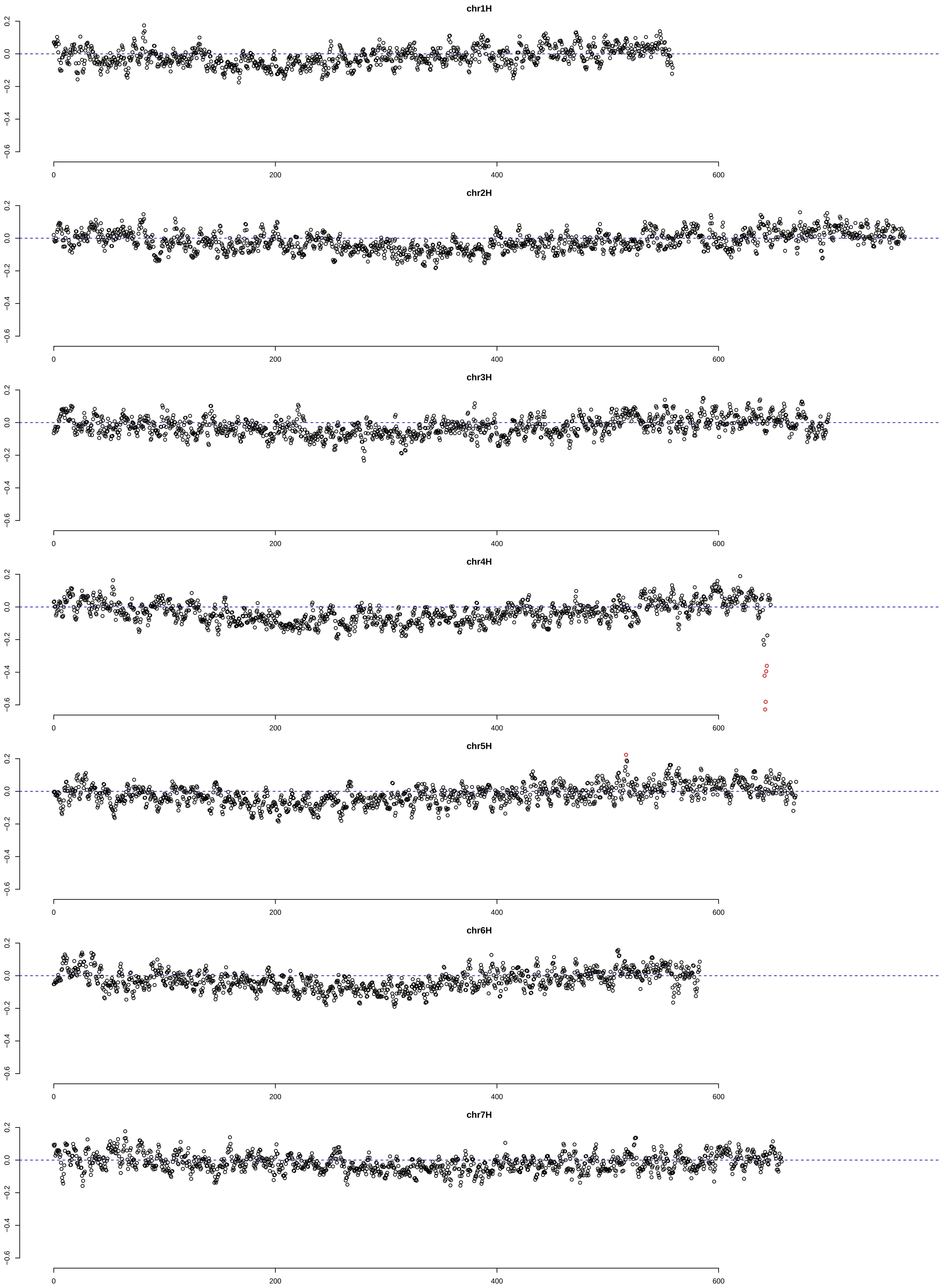

chr1H
chr2H
chr3H
chr4H
chr5H
chr6H
chr7H
0.2
0.0
-0.2
-0.4
-0.6
0
200
400
600

ERR699916

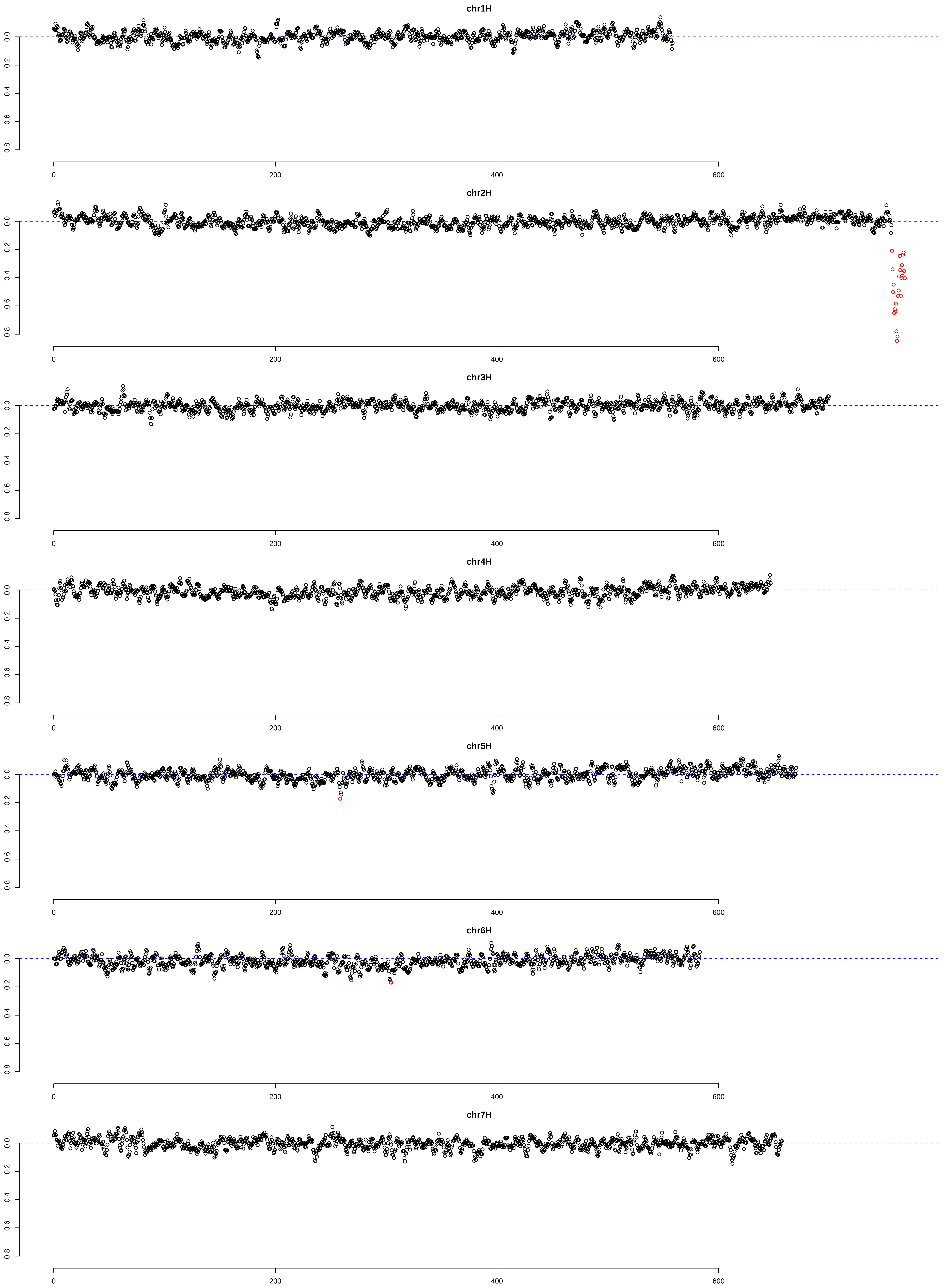
chr1H
chr2H
chr3H
chr4H
chr5H
chr6H
chr7H
0.0
-0.2
-0.4
-0.6
-0.8
0
200
400
600

ERR699917

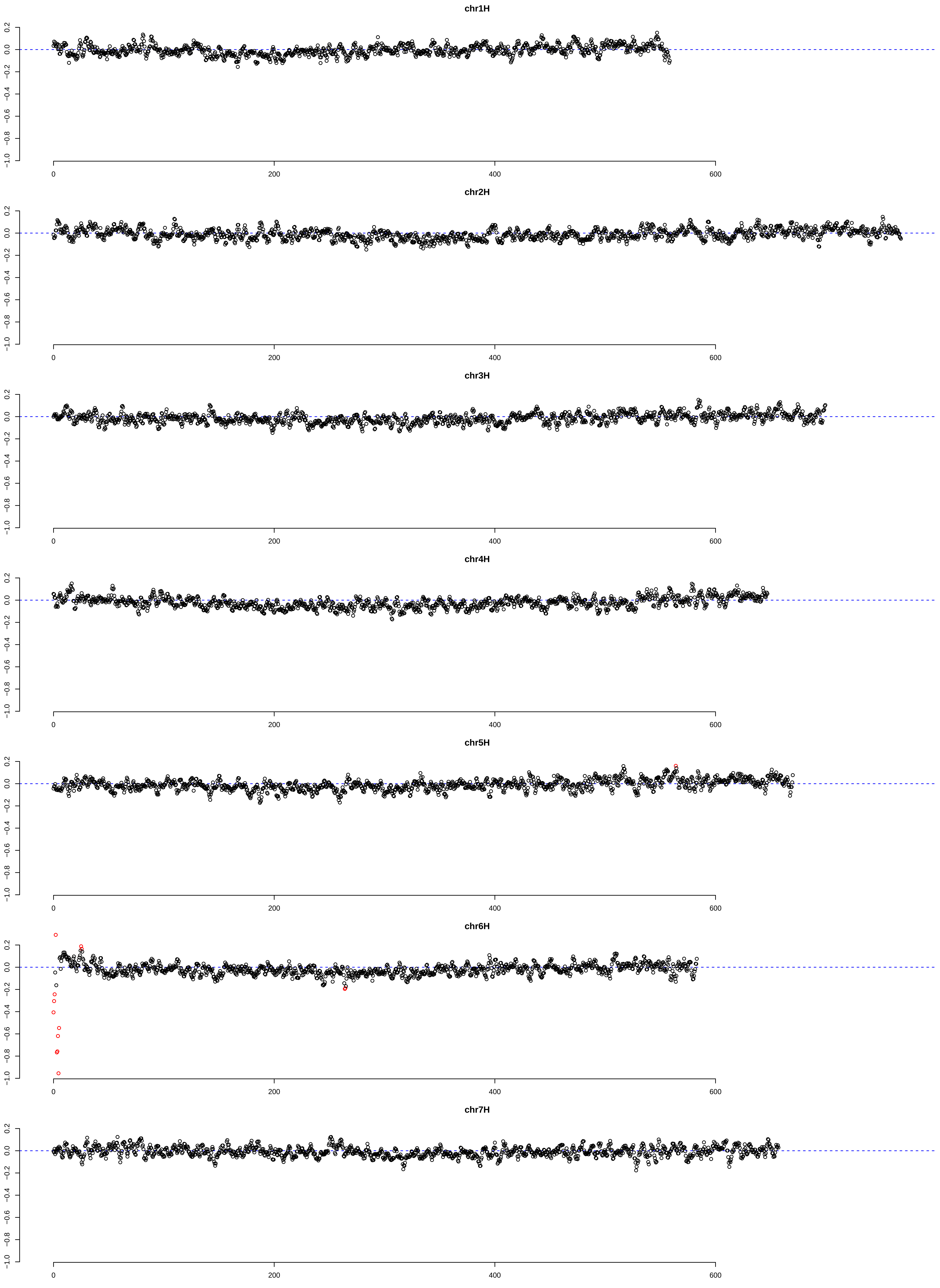

ERR699918

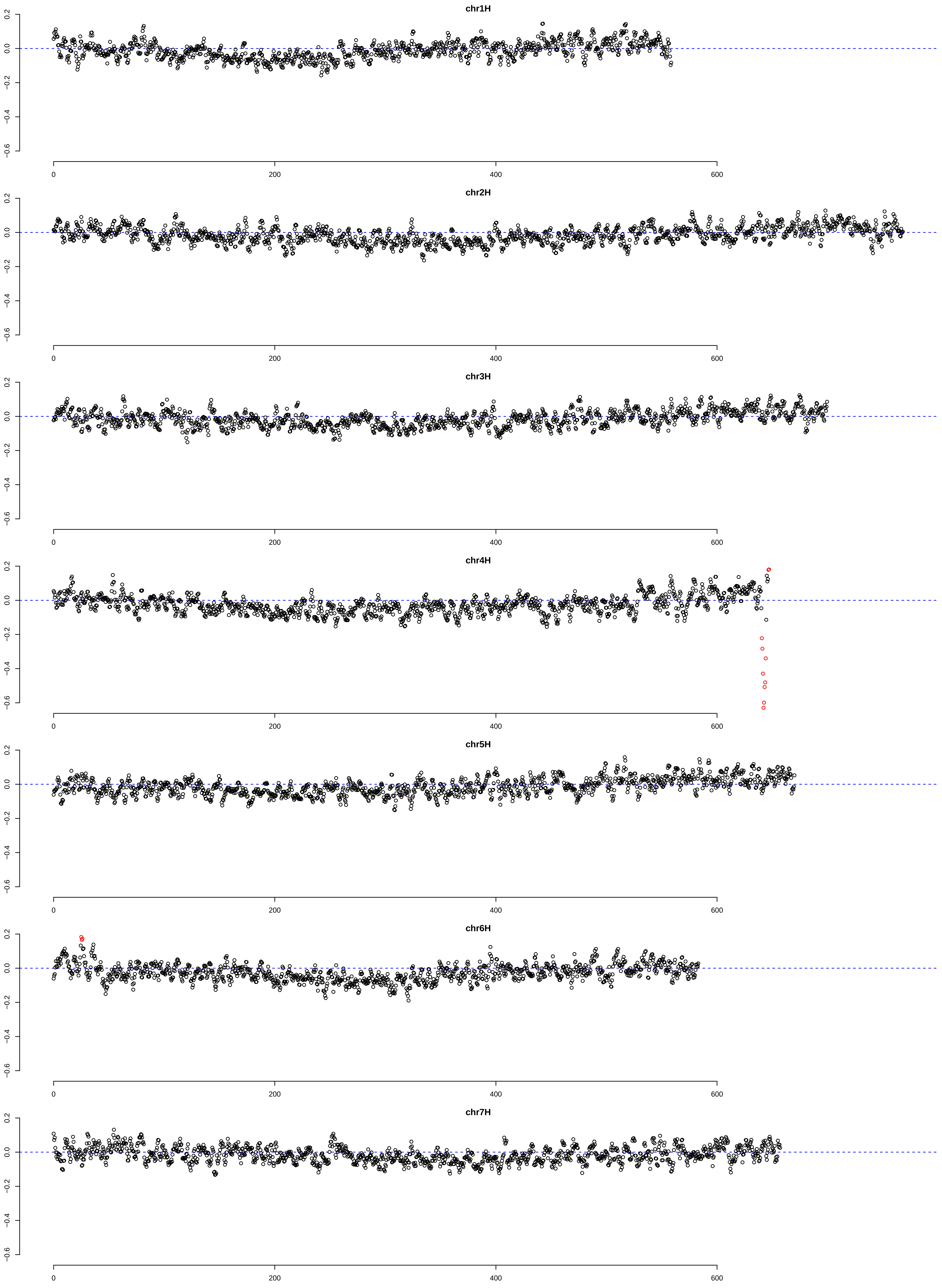

chr1H
chr2H
chr3H
chr4H
chr5H
chr6H
chr7H
0.2
0.0
-0.2
-0.4
-0.6
0
200
400
600

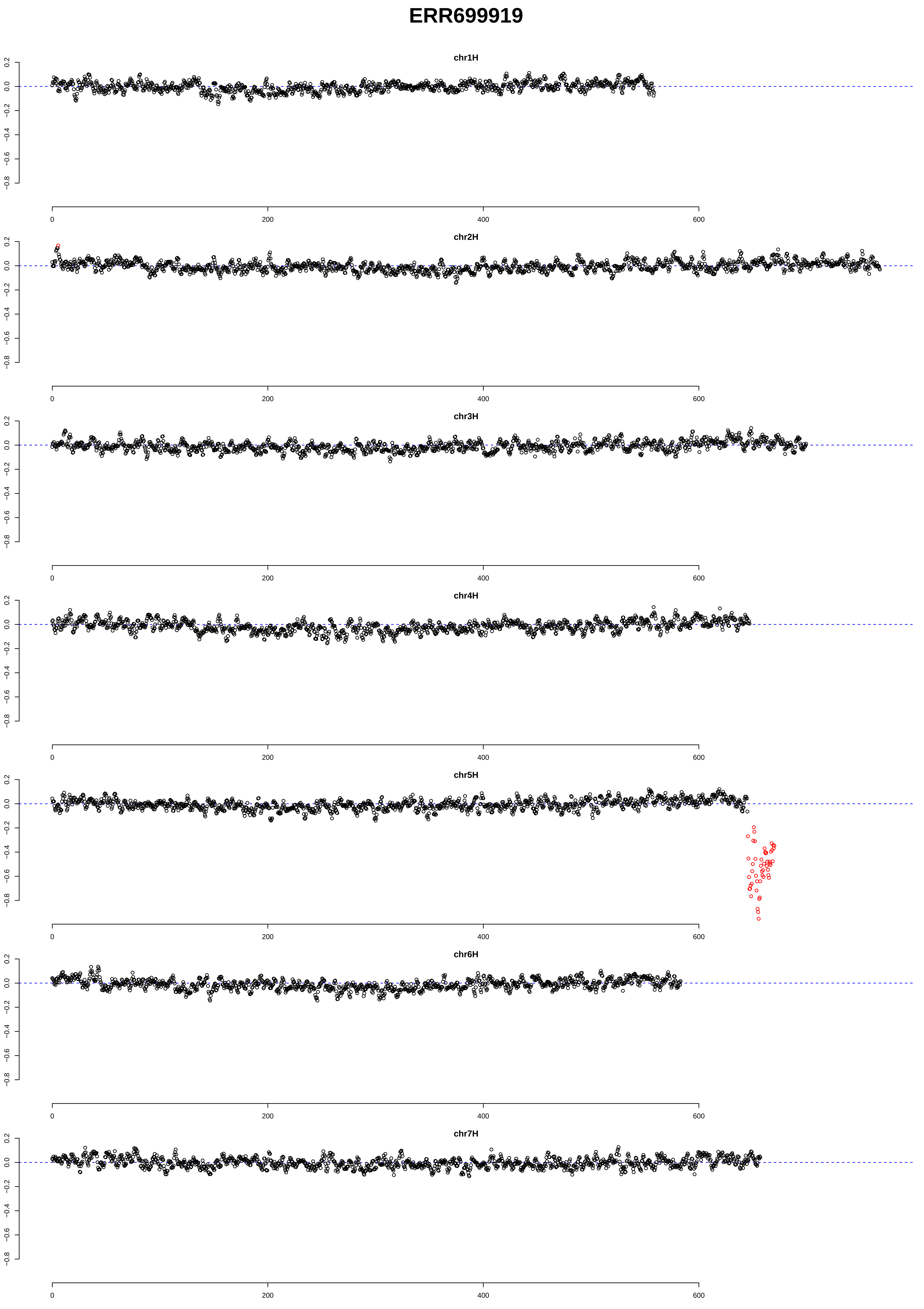
ERR699919
chr1H
chr2H
chr3H
chr4H
chr5H
chr6H
chr7H
0.2
0.0
-0.2
-0.4
-0.6
-0.8
0
200
400
600

ERR699920

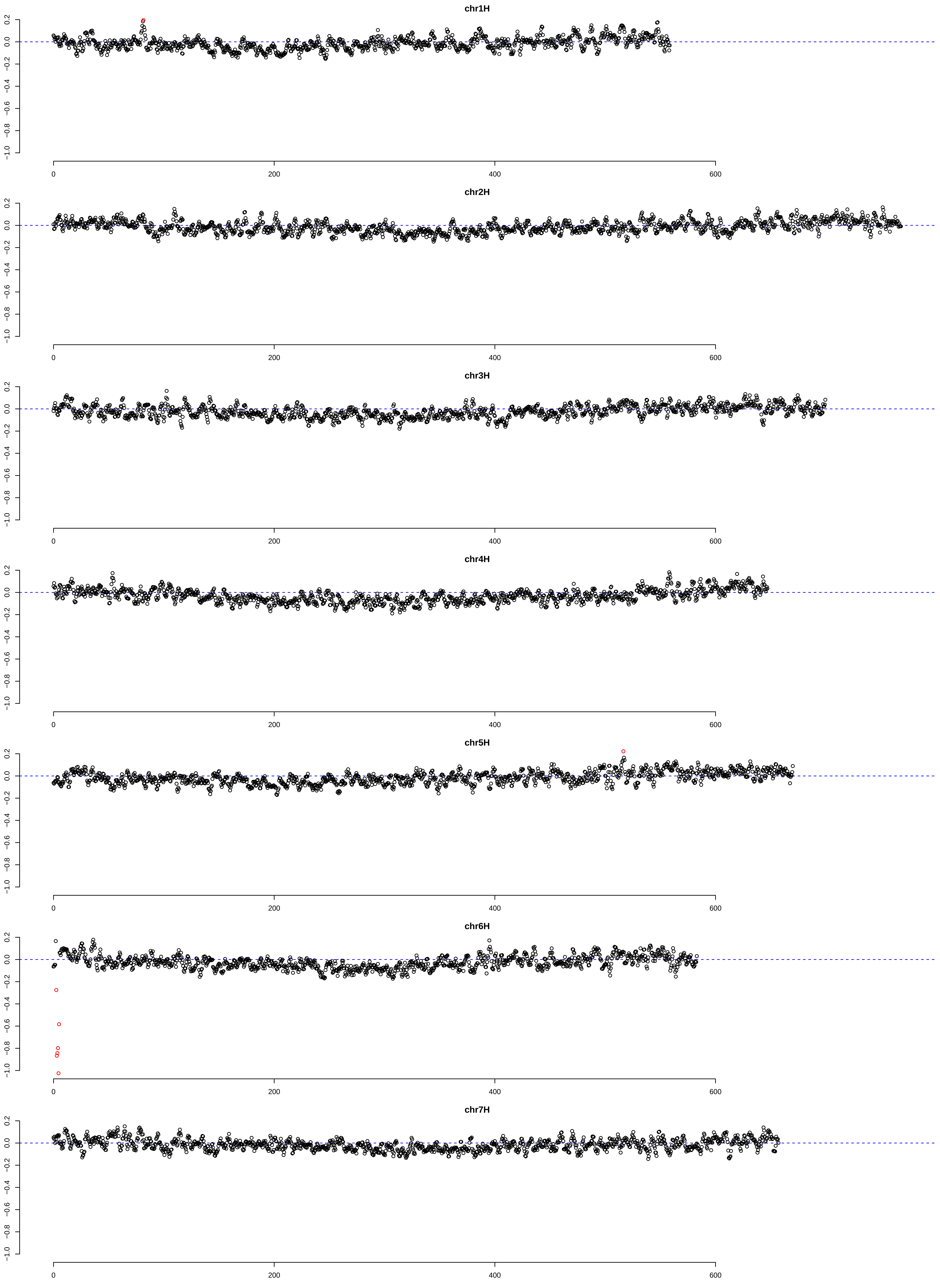
chr1H
chr2H
chr3H
chr4H
chr5H
chr6H
chr7H
0.2
0.0
−0.2
−0.4
−0.6
−0.8
−1.0
0
200
400
600

ERR699921

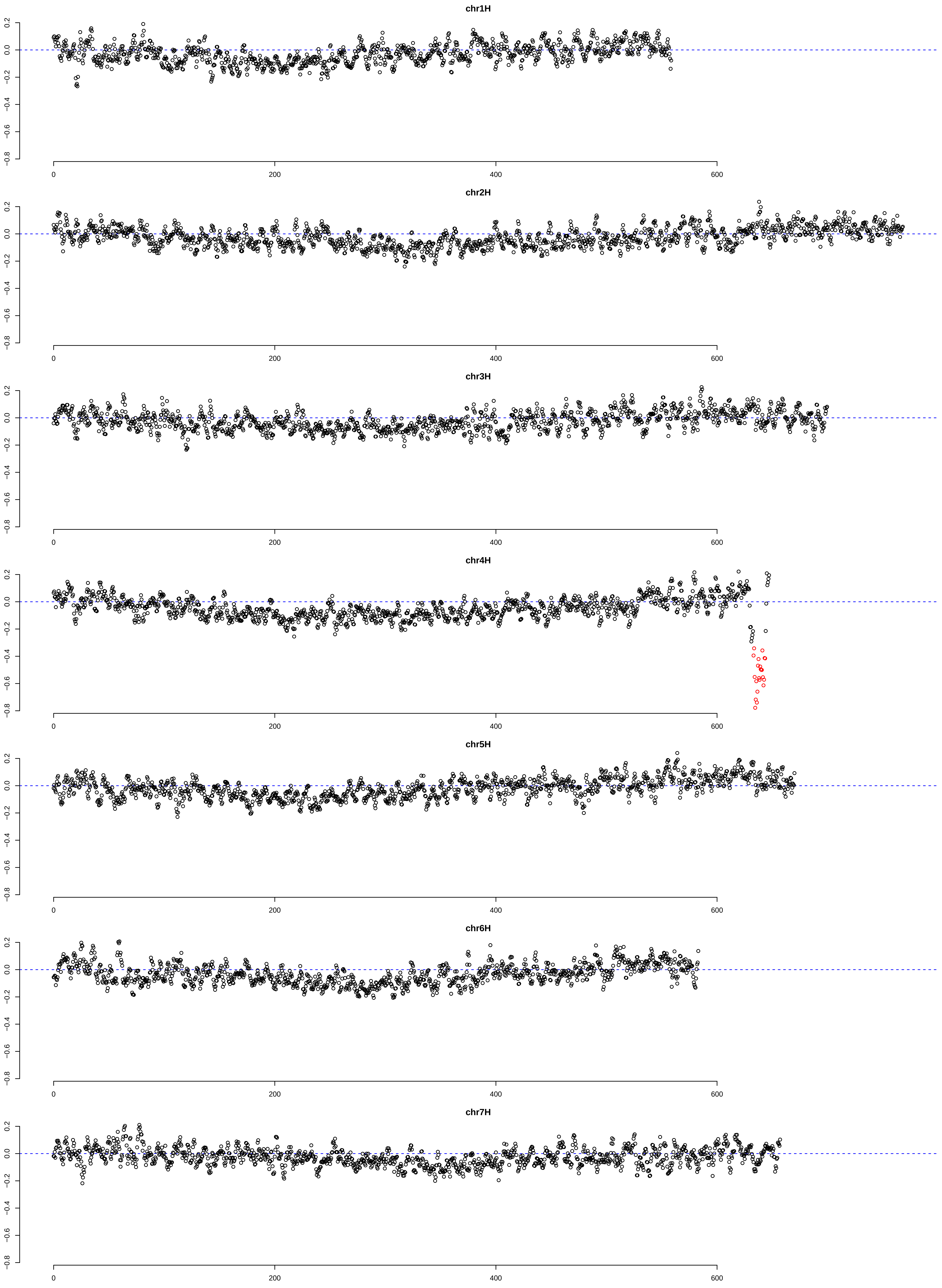

chr1H
chr2H
chr3H
chr4H
chr5H
chr6H
chr7H
0.2
0.0
-0.2
-0.4
-0.6
-0.8
0
200
400
600

ERR699922

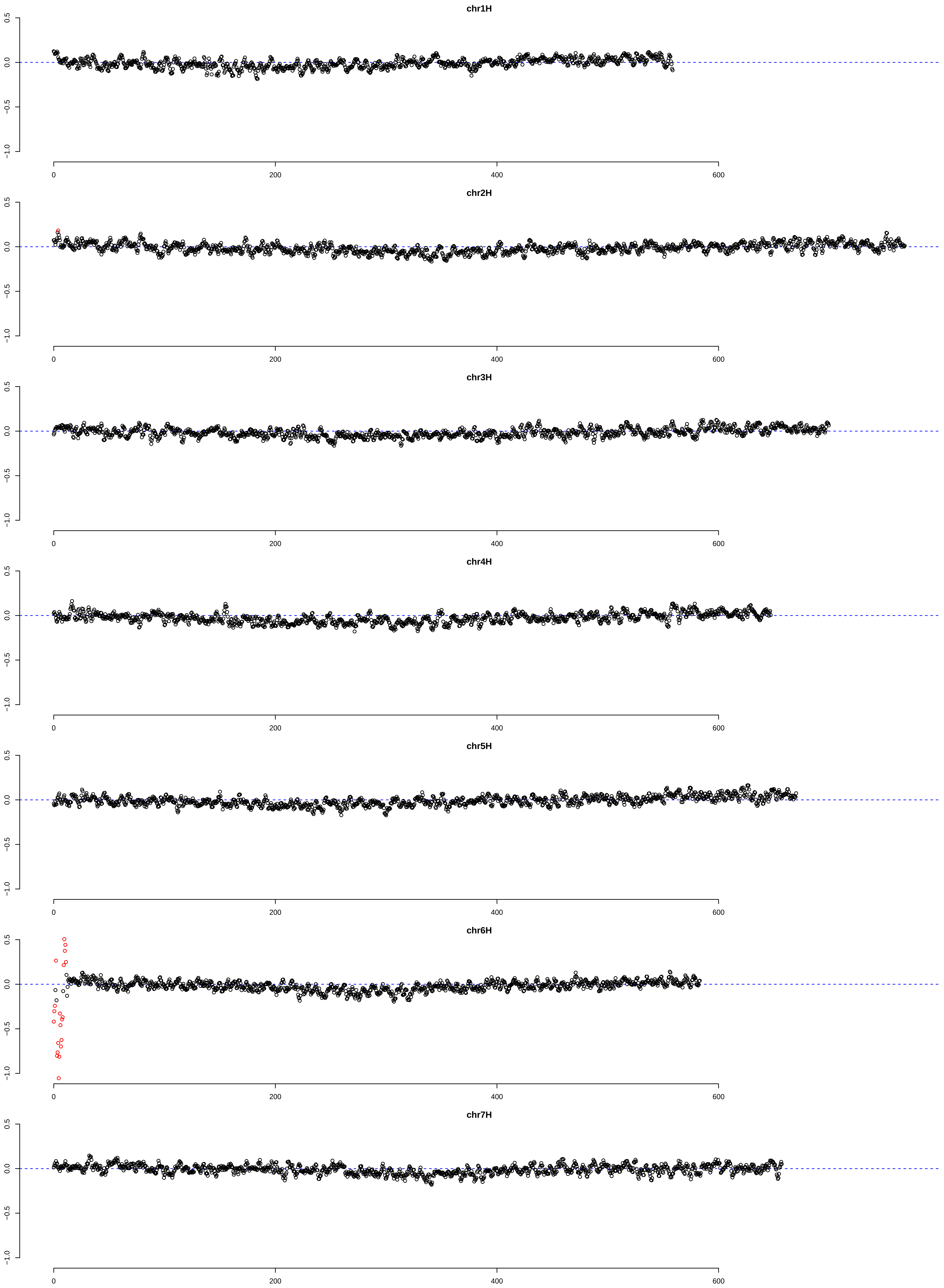

chr1H
chr2H
chr3H
chr4H
chr5H
chr6H
chr7H
0.5
0.0
−0.5
−1.0
0
200
400
600

ERR699923

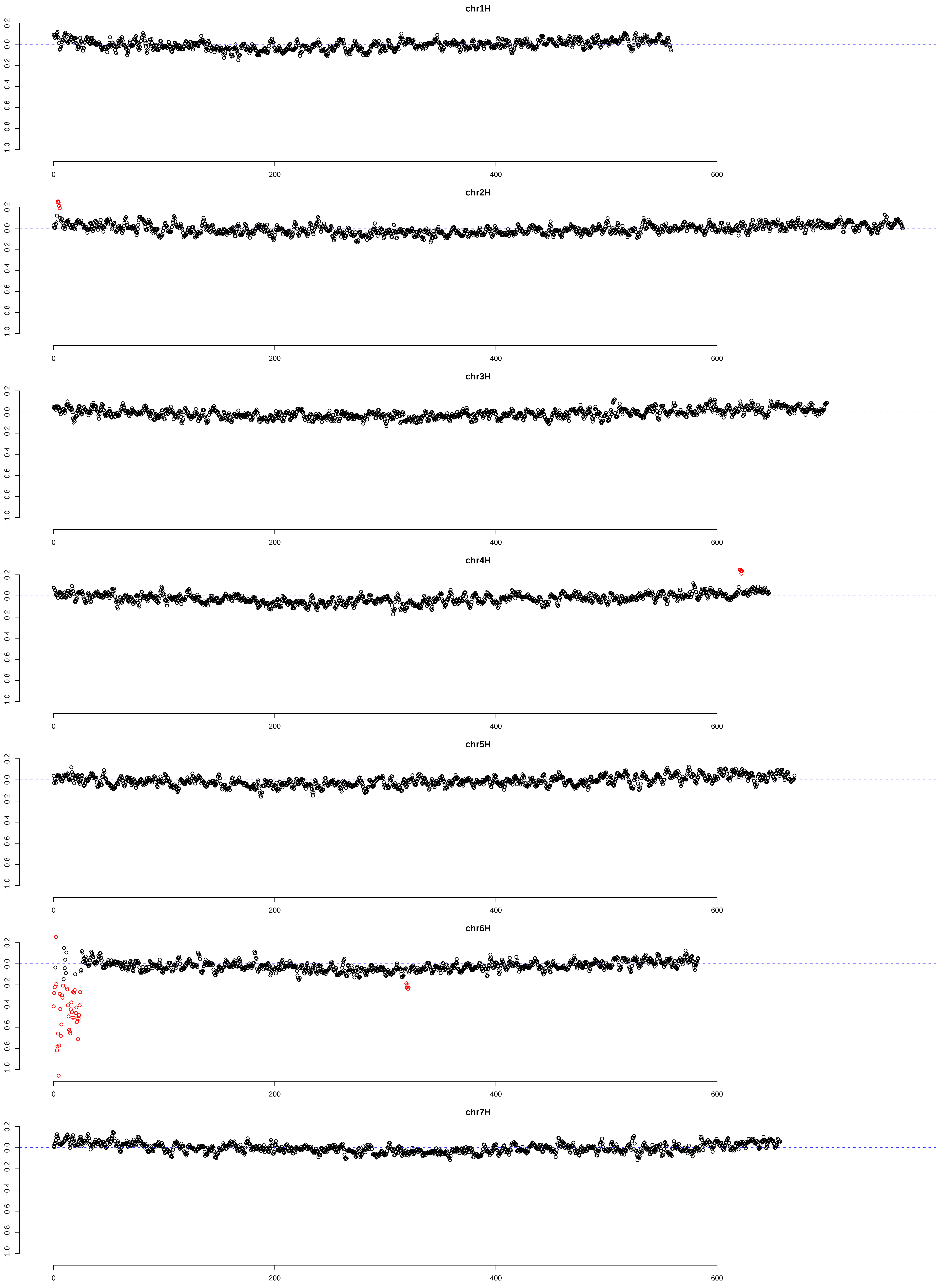

chr1H
chr2H
chr3H
chr4H
chr5H
chr6H
chr7H
0.2
0.0
−0.2
−0.4
−0.6
−0.8
−1.0
0
200
400
600

ERR699924

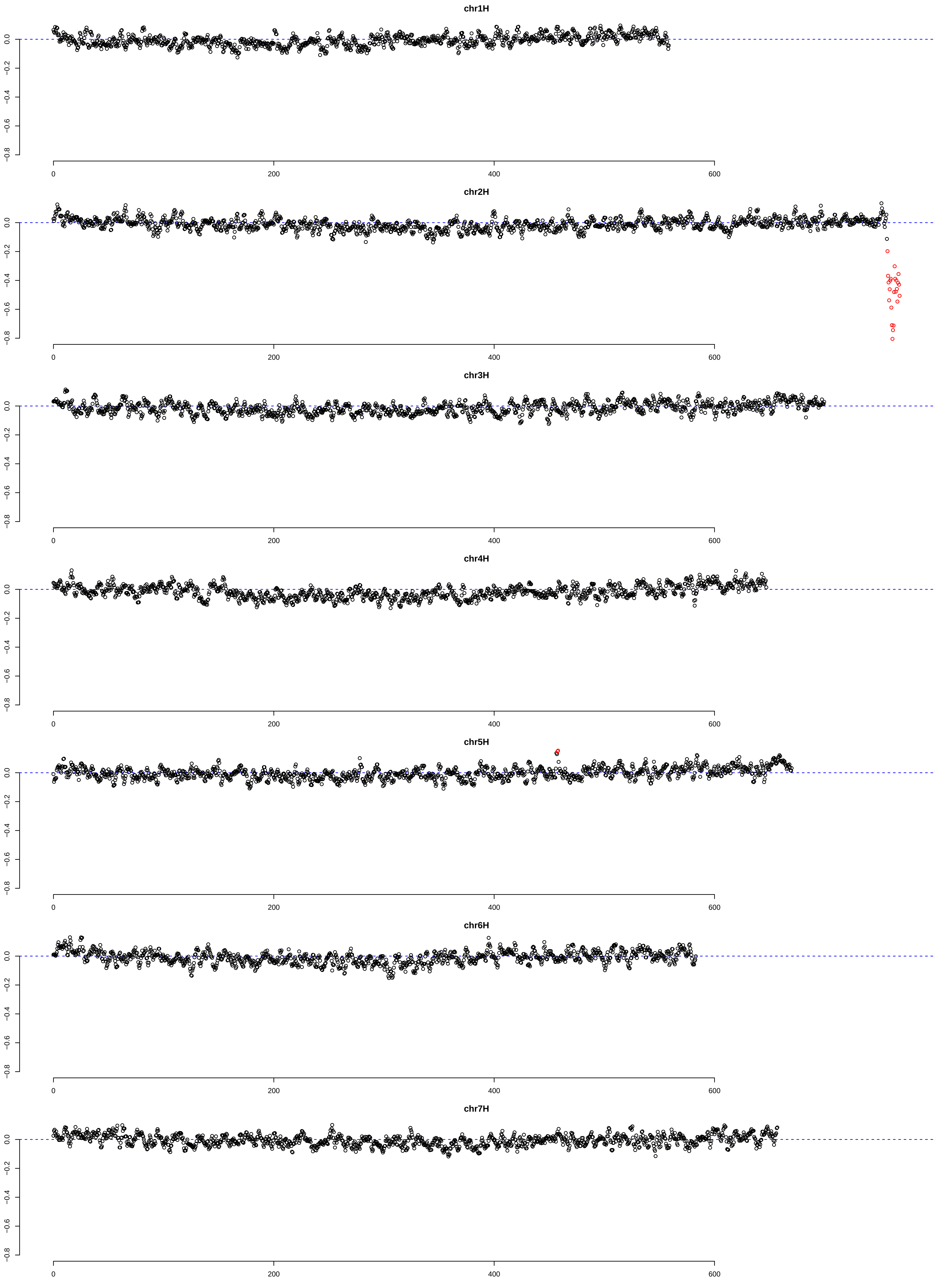
chr1H
chr2H
chr3H
chr4H
chr5H
chr6H
chr7H
0.0
-0.2
-0.4
-0.6
-0.8
0
200
400
600

ERR699925

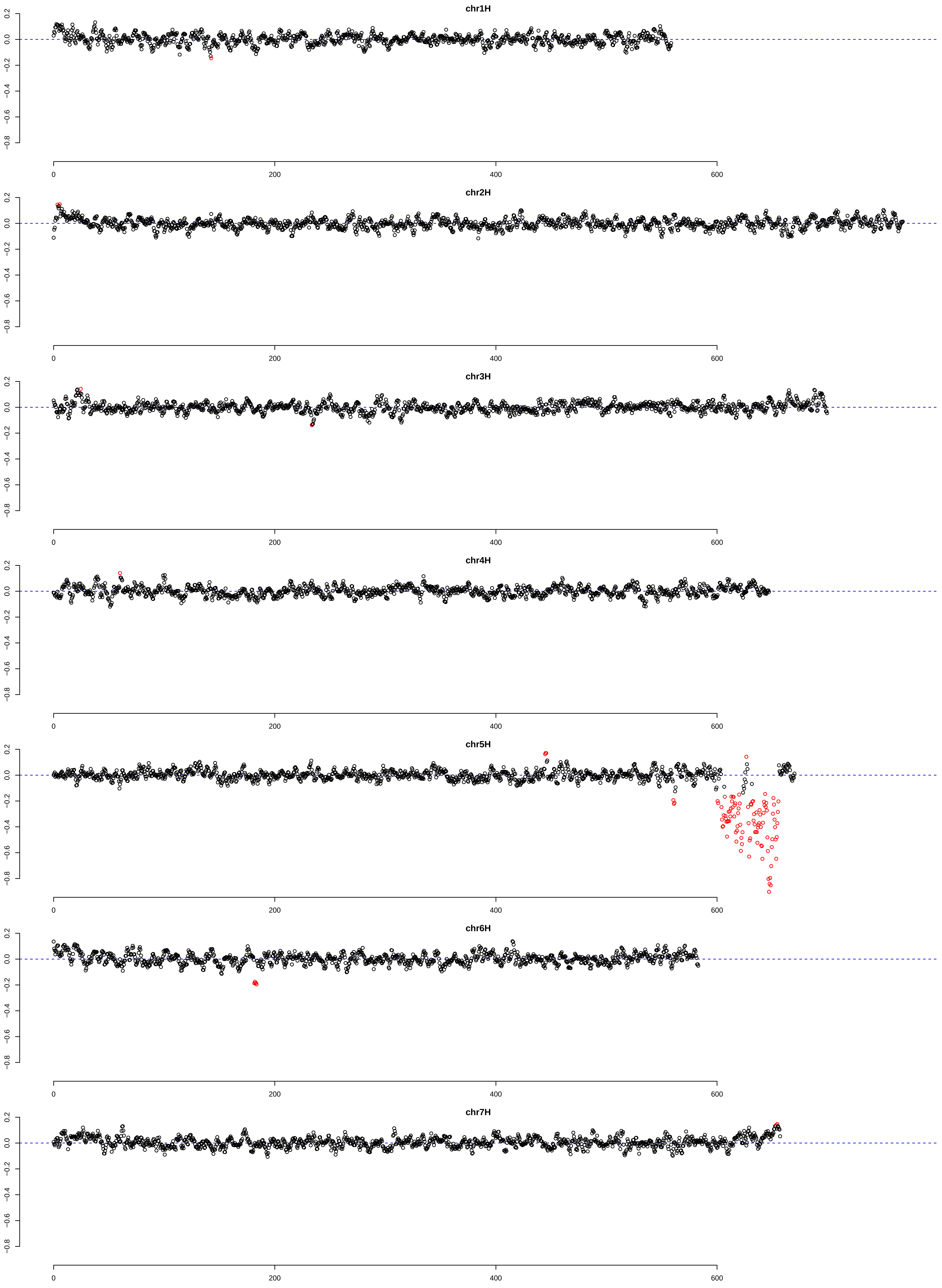

chr1H
chr2H
chr3H
chr4H
chr5H
chr6H
chr7H
0.2
0.0
-0.2
-0.4
-0.6
-0.8
0
200
400
600

ERR699926

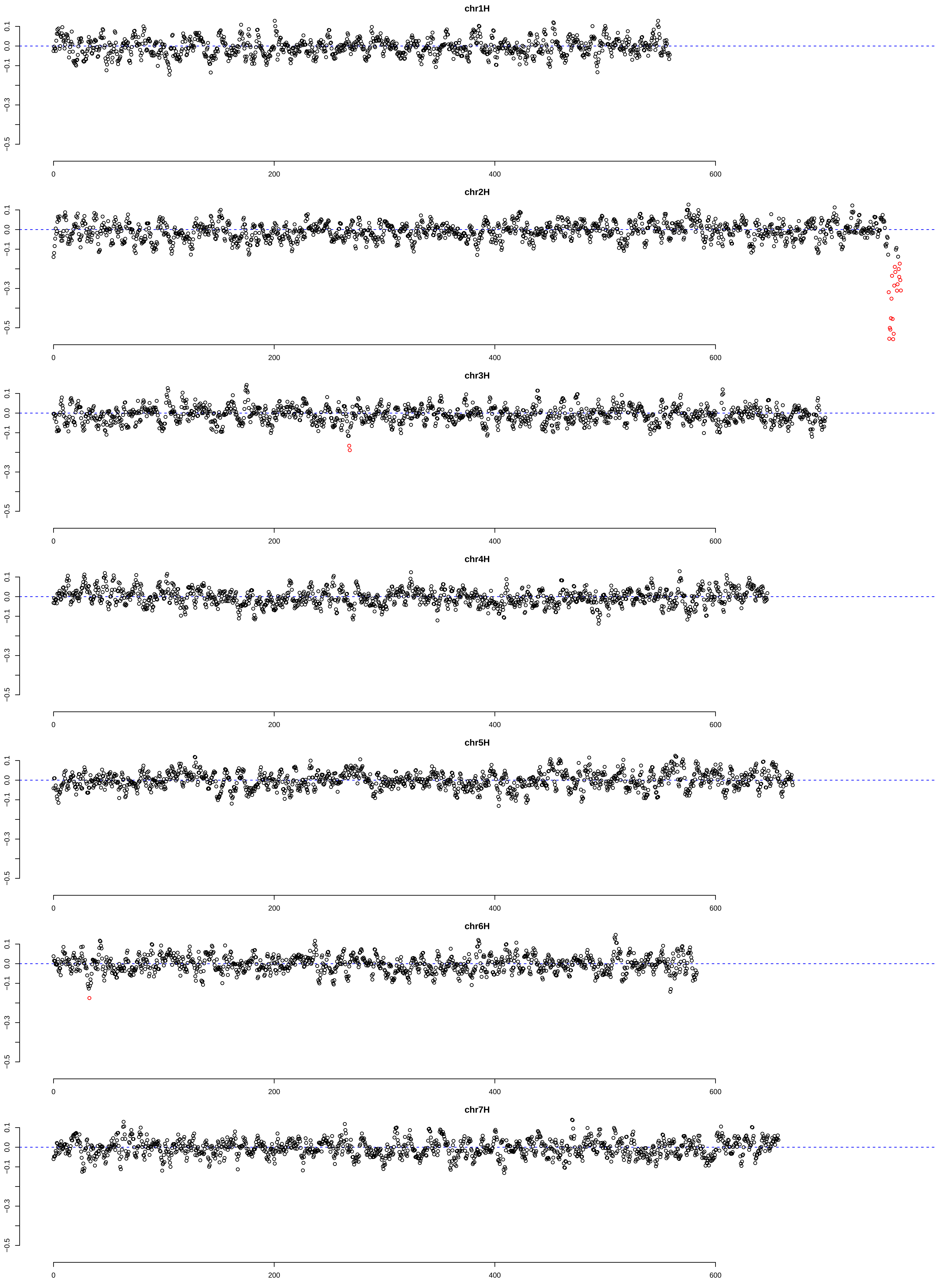
chr1H
chr2H
chr3H
chr4H
chr5H
chr6H
chr7H
0.1
0.0
-0.1
-0.3
-0.5
0
200
400
600

ERR699927

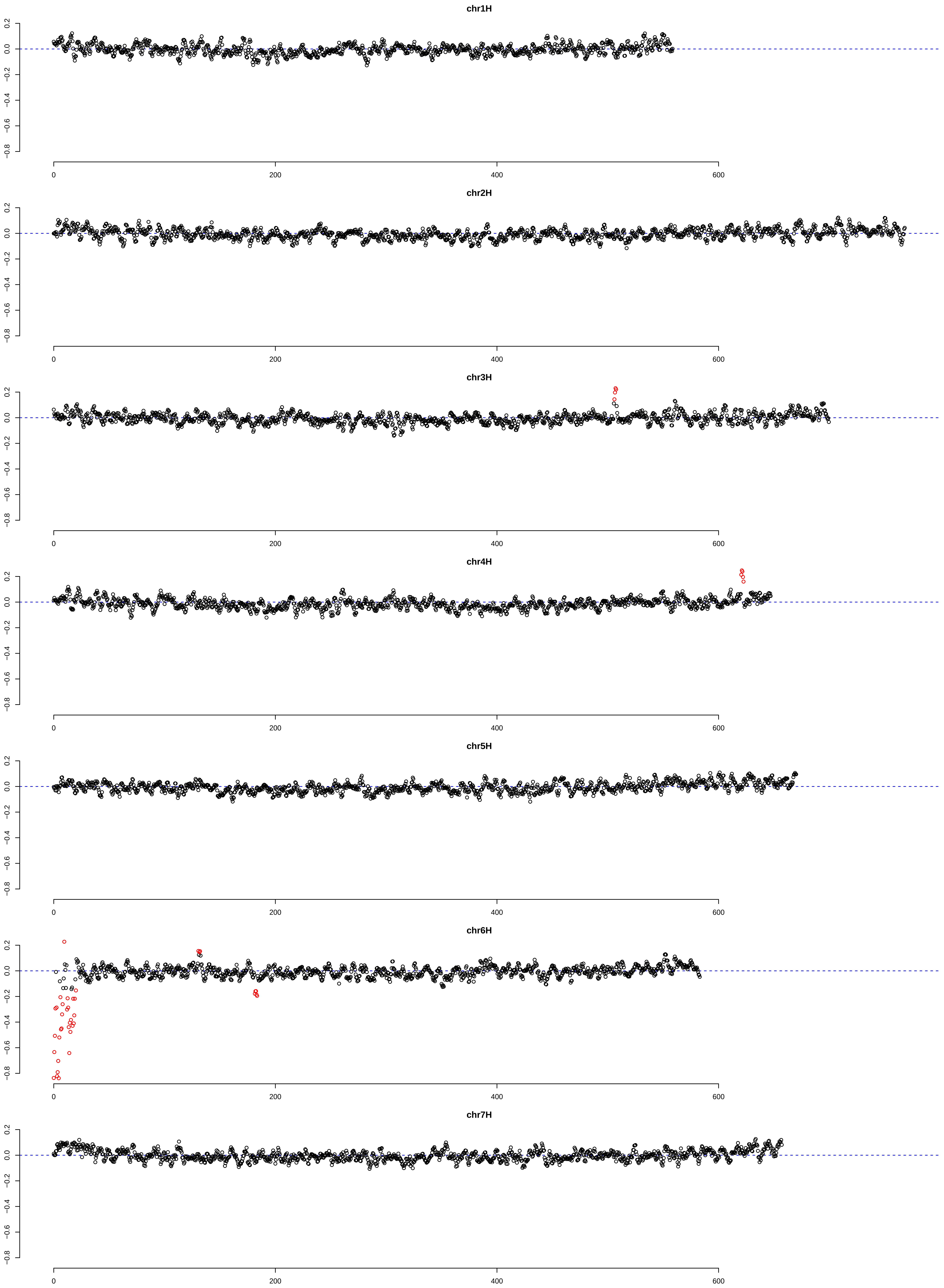

chr1H
chr2H
chr3H
chr4H
chr5H
chr6H
chr7H
0.2
0.0
-0.2
-0.4
-0.6
-0.8
0
200
400
600

ERR699928

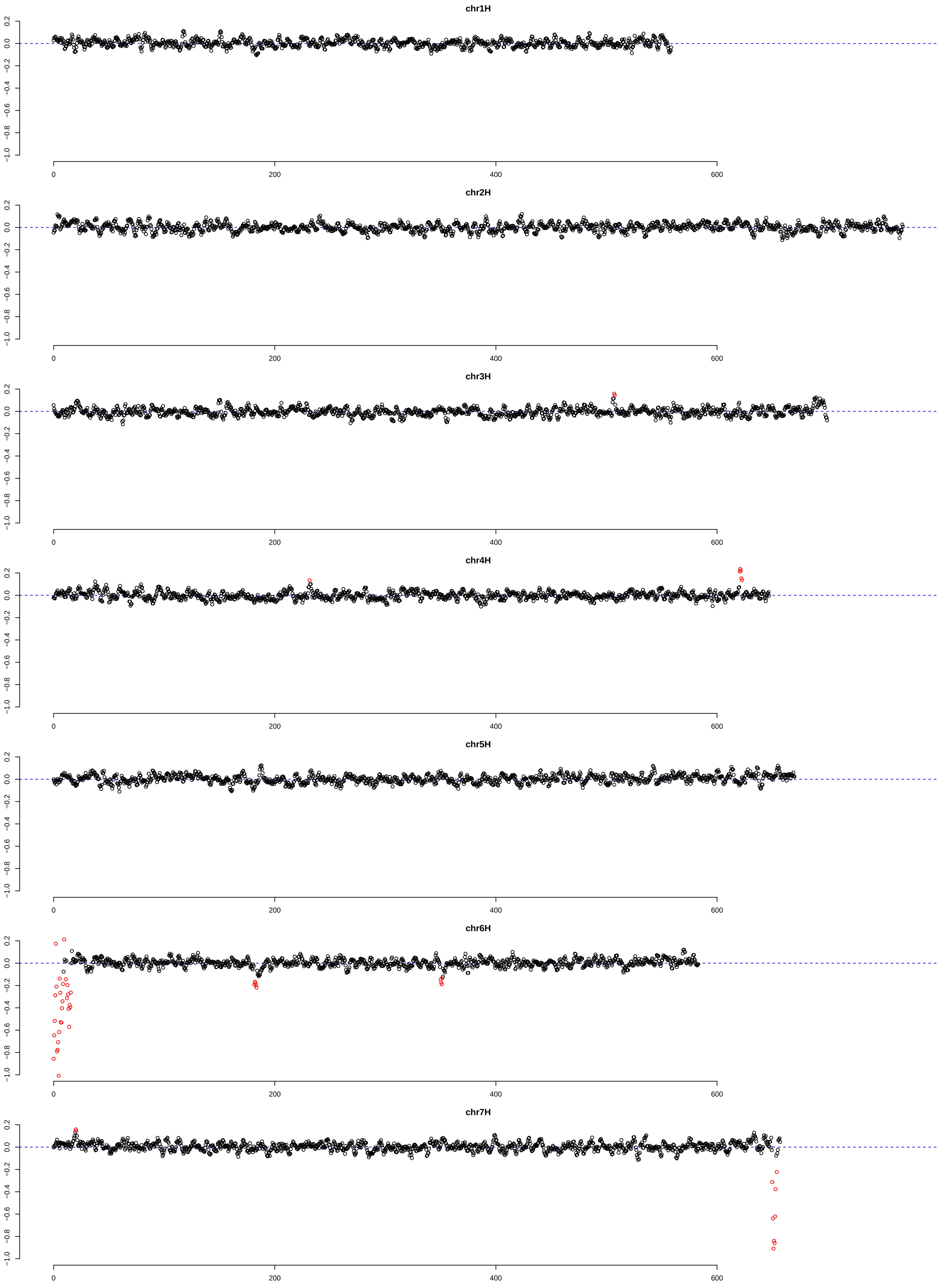
chr1H
chr2H
chr3H
chr4H
chr5H
chr6H
chr7H
0.2
0.0
−0.2
−0.4
−0.6
−0.8
−1.0
0
200
400
600

ERR699929

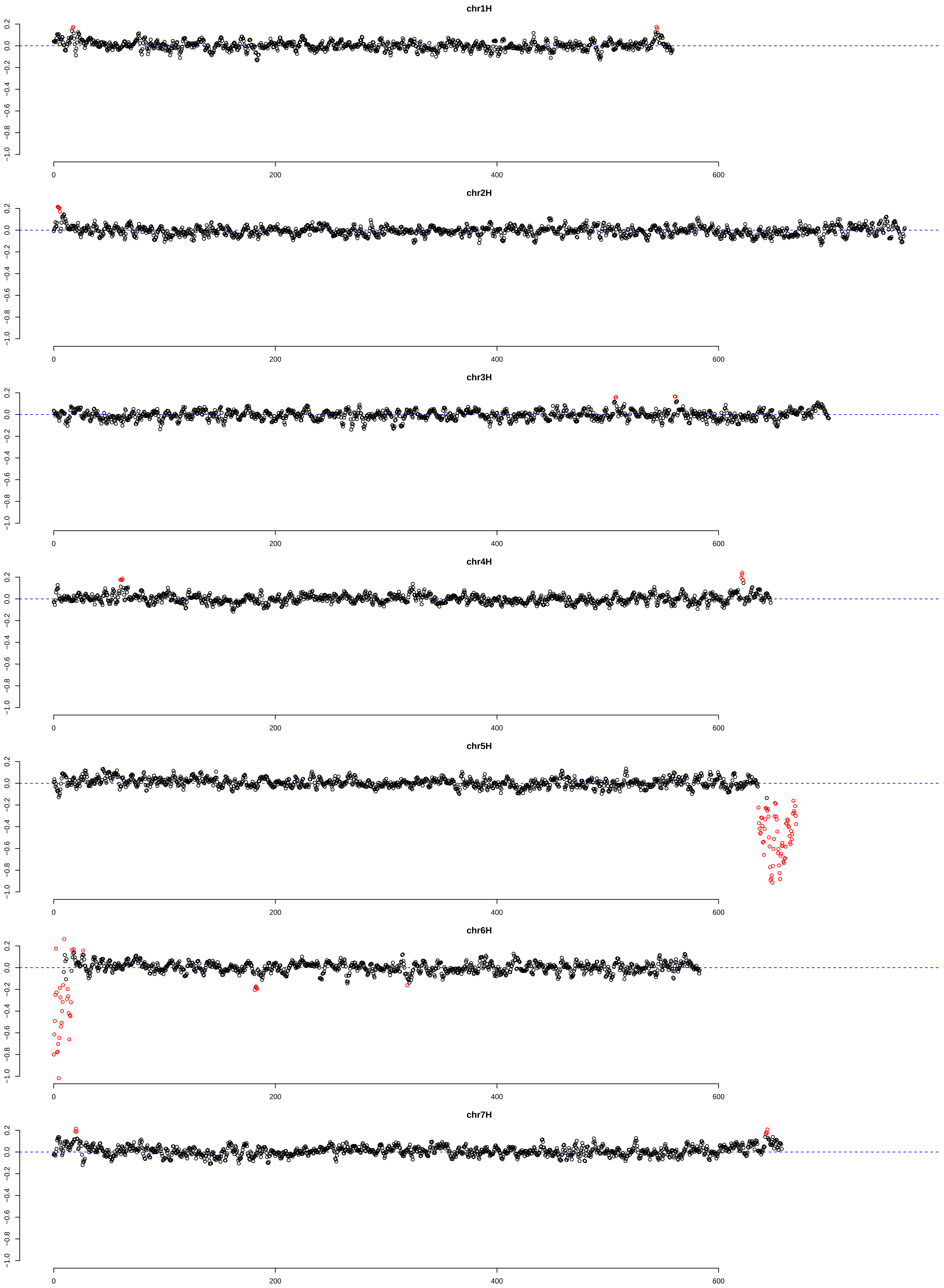
chr1H
chr2H
chr3H
chr4H
chr5H
chr6H
chr7H
0.2
0.0
−0.2
−0.4
−0.6
−0.8
−1.0
0
200
400
600

ERR699930

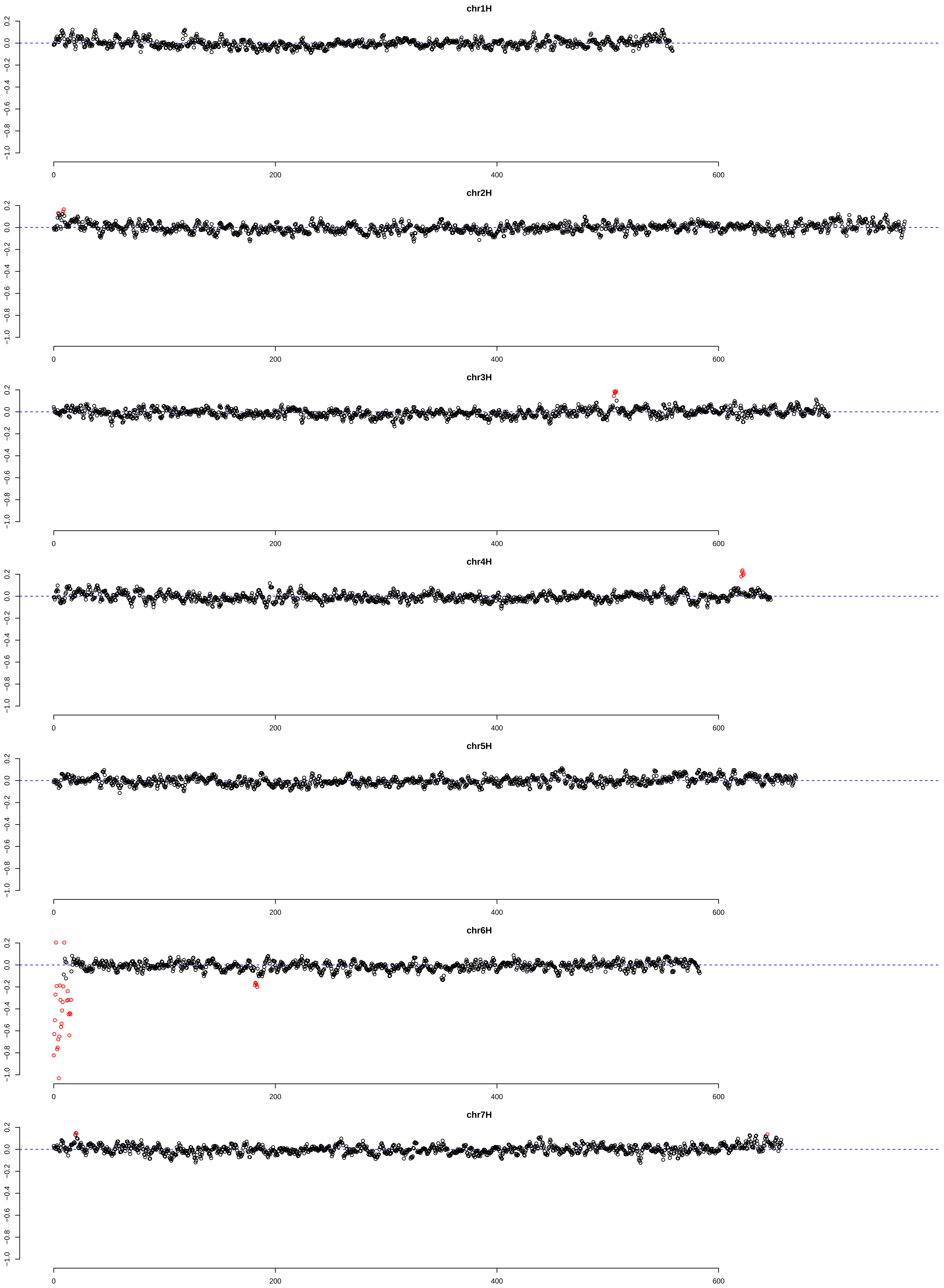
chr1H
chr2H
chr3H
chr4H
chr5H
chr6H
chr7H
0.2
0.0
−0.2
−0.4
−0.6
−0.8
−1.0
0
200
400
600

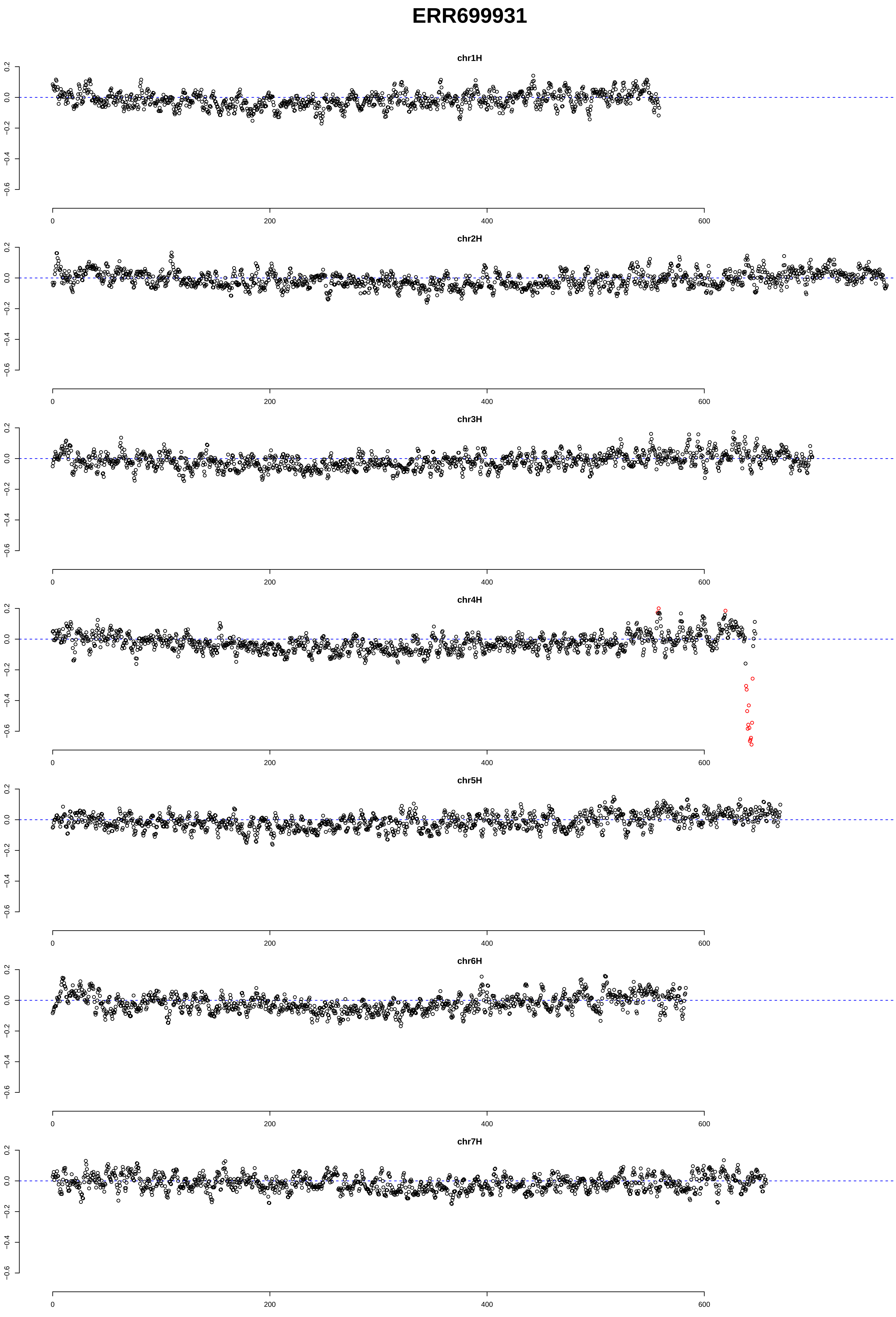
ERR699931
chr1H
chr2H
chr3H
chr4H
chr5H
chr6H
chr7H
0.2
0.0
-0.2
-0.4
-0.6
0
200
400
600

ERR699932

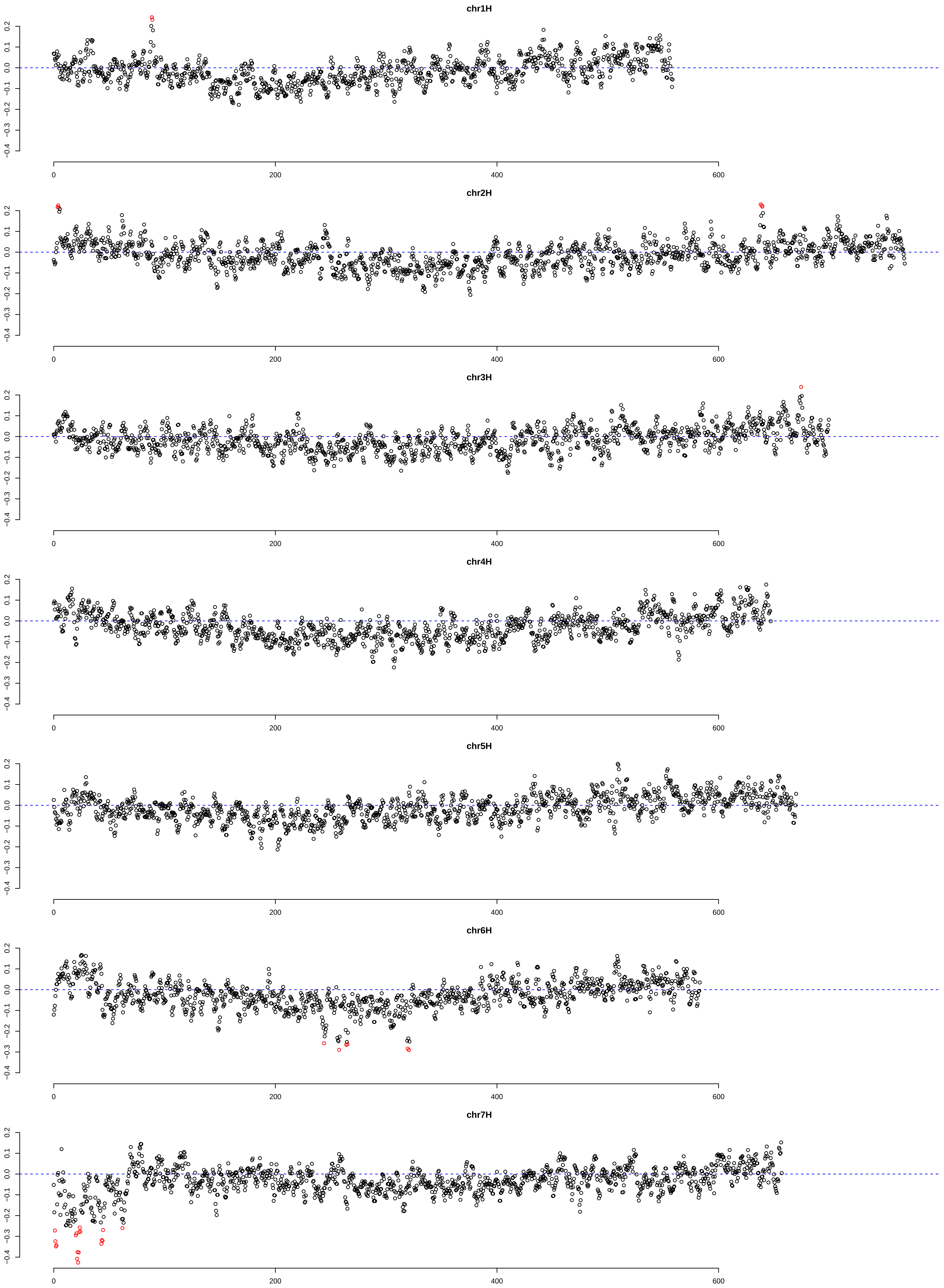
chr1H
chr2H
chr3H
chr4H
chr5H
chr6H
chr7H
0.2
0.1
0.0
−0.1
−0.2
−0.3
−0.4
0
200
400
600

ERR699933

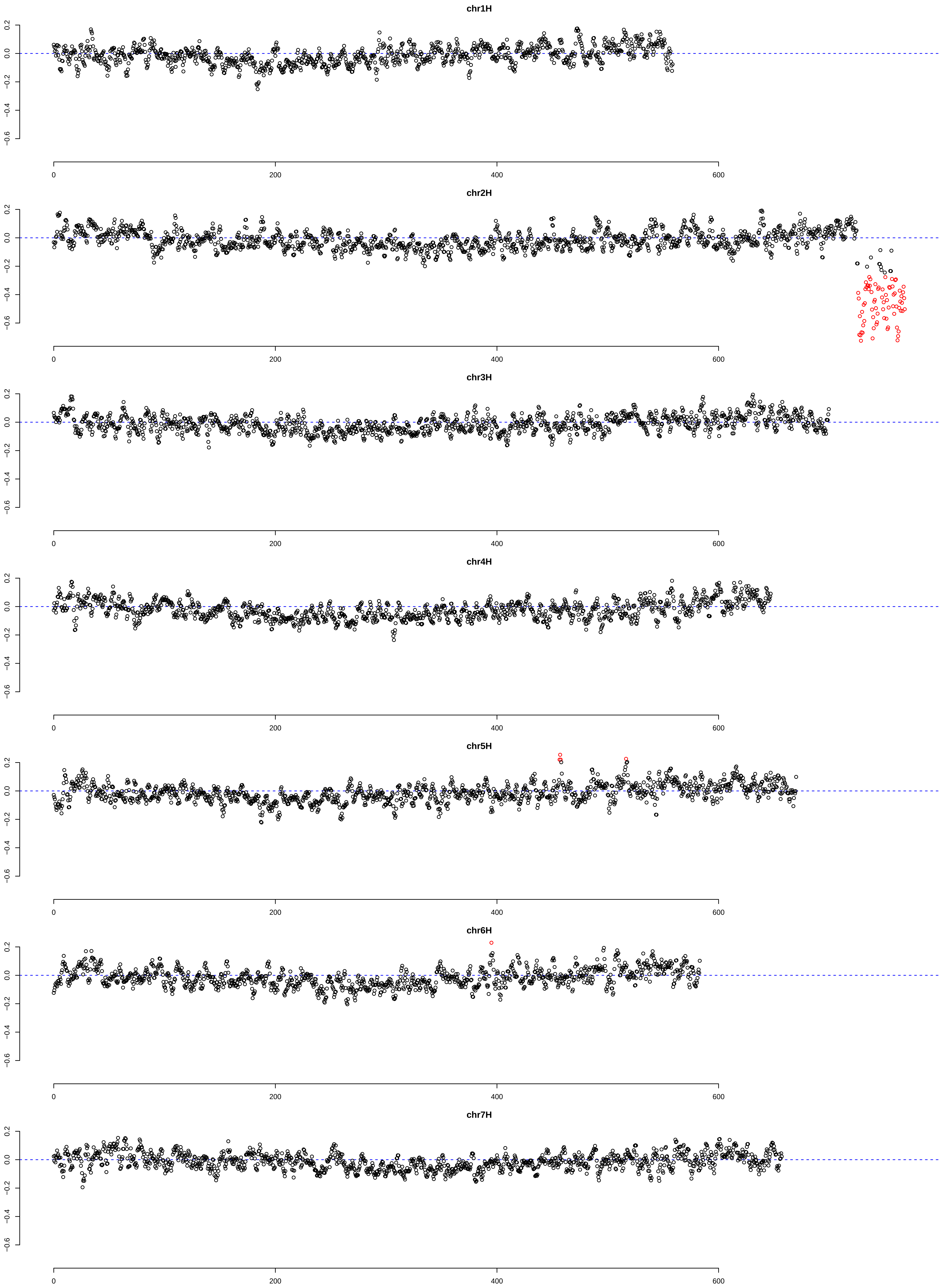

chr1H
chr2H
chr3H
chr4H
chr5H
chr6H
chr7H
0.2
0.0
-0.2
-0.4
-0.6
0
200
400
600

ERR699934

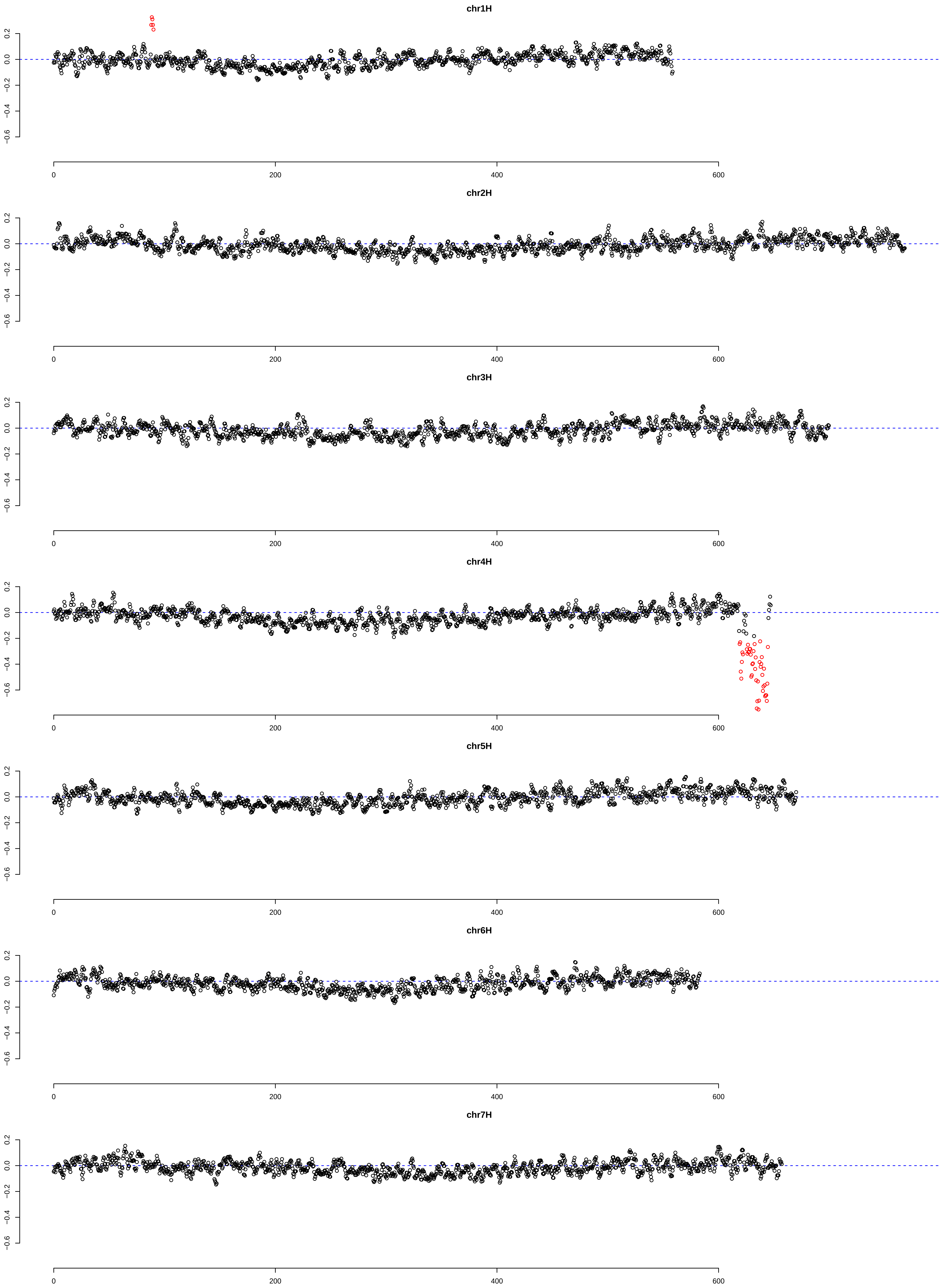
chr1H
chr2H
chr3H
chr4H
chr5H
chr6H
chr7H
0.2
0.0
-0.2
-0.4
-0.6
0
200
400
600

ERR699935

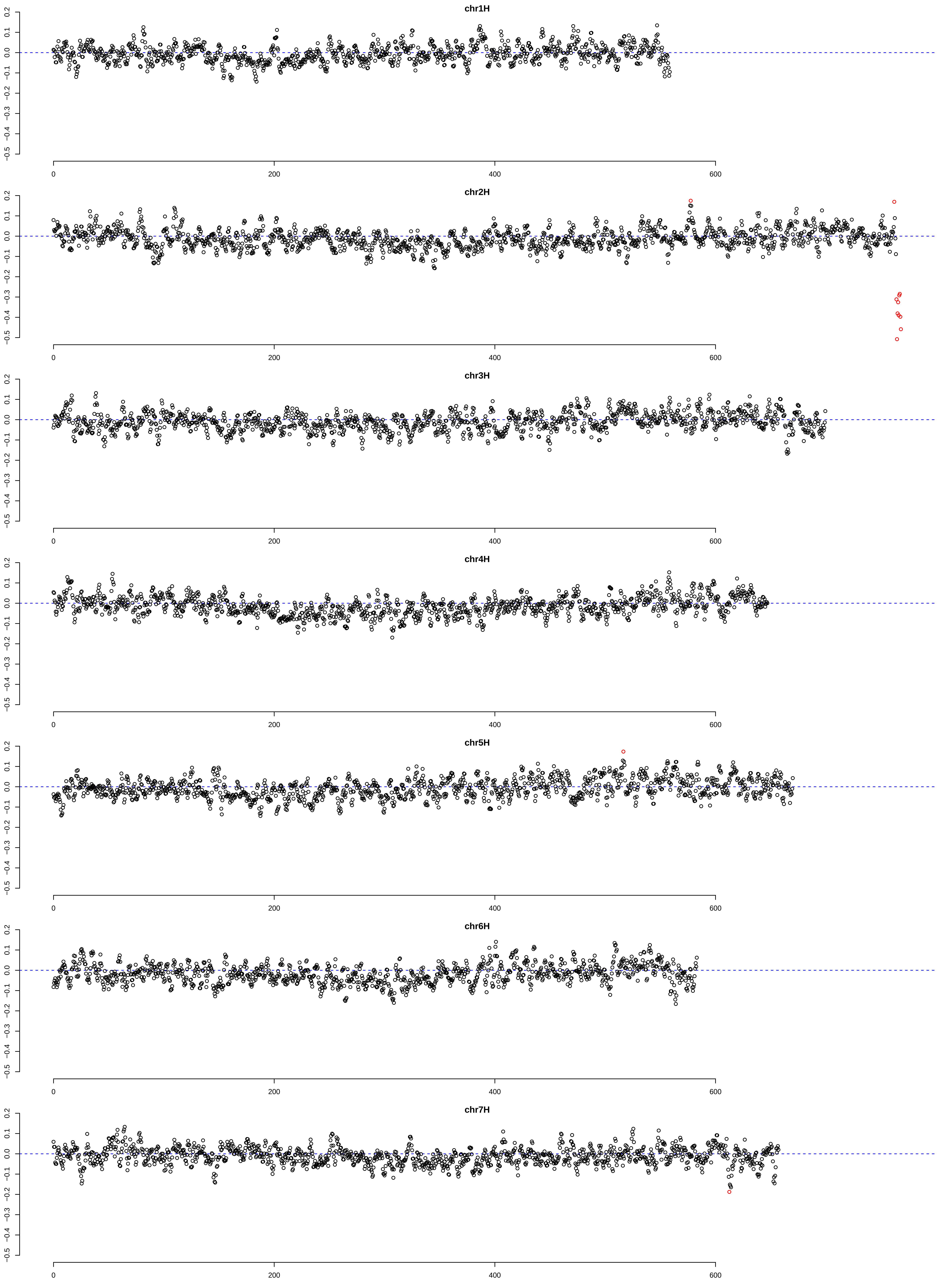

chr1H
chr2H
chr3H
chr4H
chr5H
chr6H
chr7H
0.2
0.1
0.0
−0.1
−0.2
−0.3
−0.4
−0.5
0
200
400
600

ERR699937

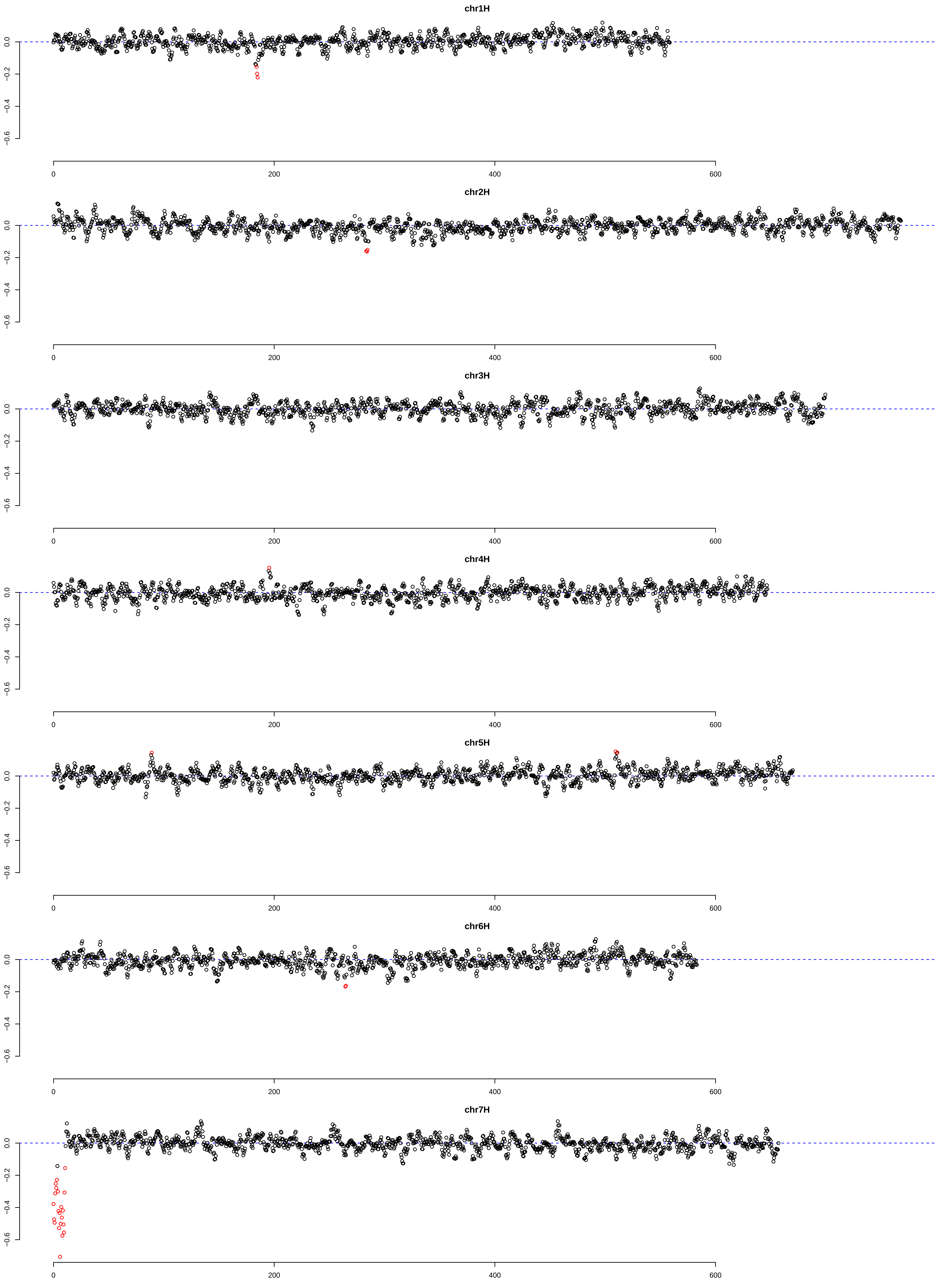

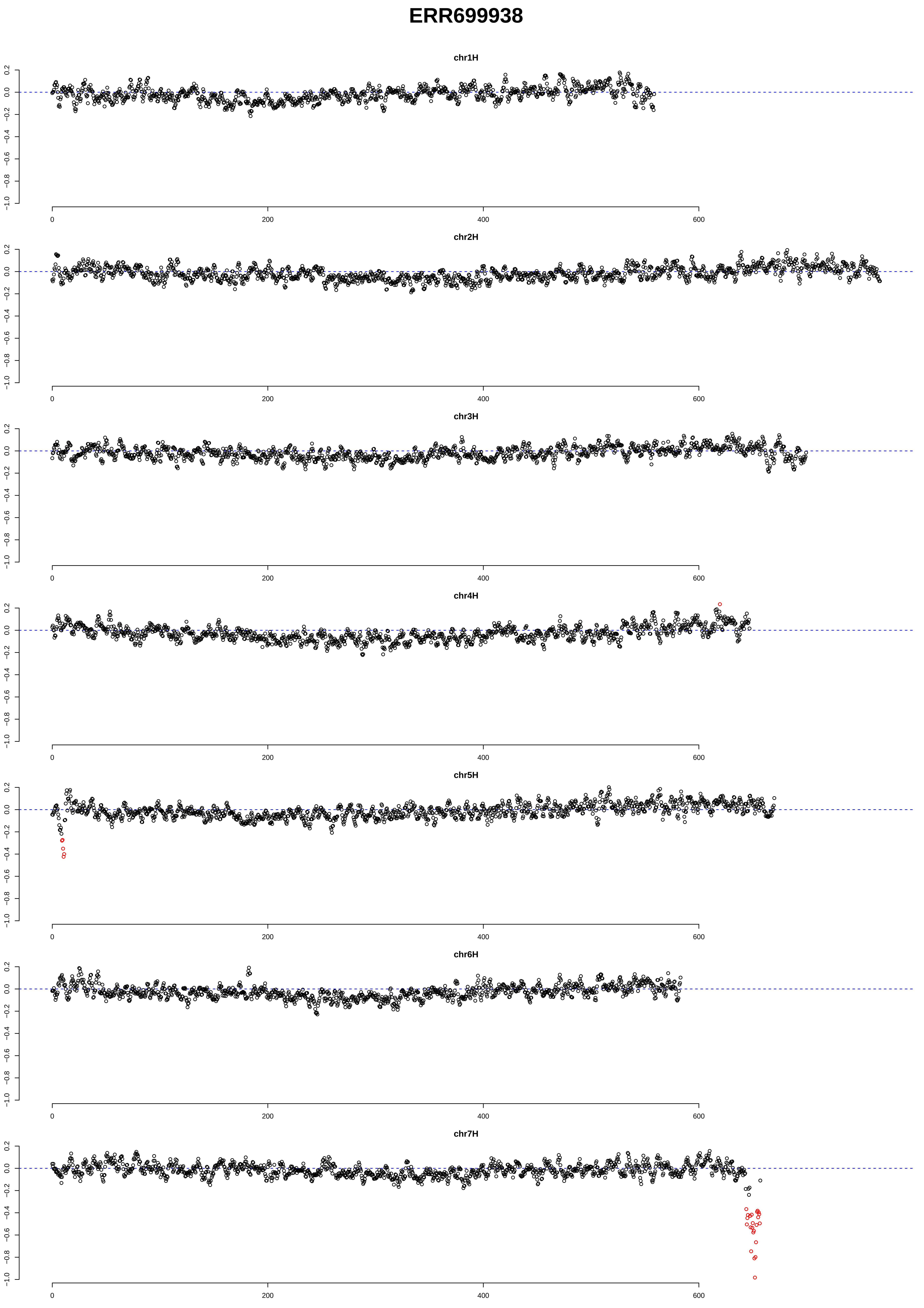
ERR699938
chr1H
chr2H
chr3H
chr4H
chr5H
chr6H
chr7H
0.2
0.0
−0.2
−0.4
−0.6
−0.8
−1.0
0
200
400
600
