# Supplementary material for: Detecting Large Chromosomal Modifications Using Short Read Data From Genotyping-by-Sequencing
Source: Front Plant Sci. 2019 Sep 24;10:1133. doi: 10.3389/fpls.2019.01133 (PMC6771380; doi:10.3389/fpls.2019.01133)
Supplement: Supplemental Data Sheet 2 — Coverage profiles of large chromosomal modifications with decreased coverage form selected wheat genotypes. [file DataSheet_2.pdf]

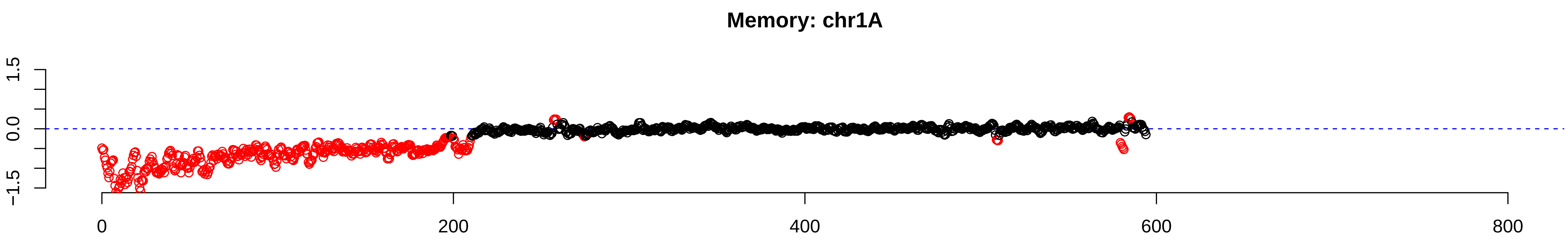

Memory: chr1A
1.5
0.0
−1.5
0
200
400
600
800

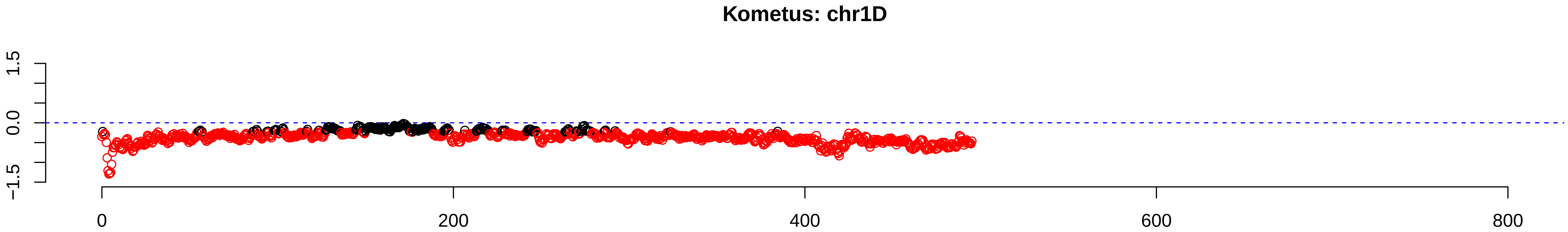
Kometus: chr1D
1.5
0.0
−1.5
0
200
400
600
800

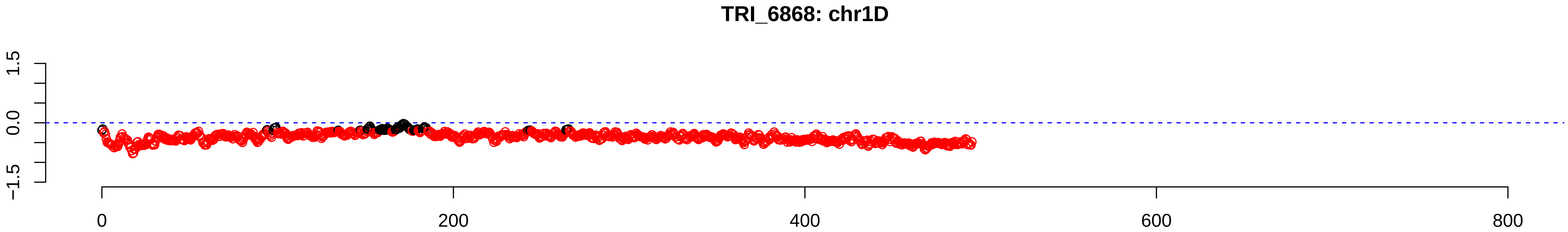
TRI_6868: chr1D
1.5
0.0
−1.5
0
200
400
600
800

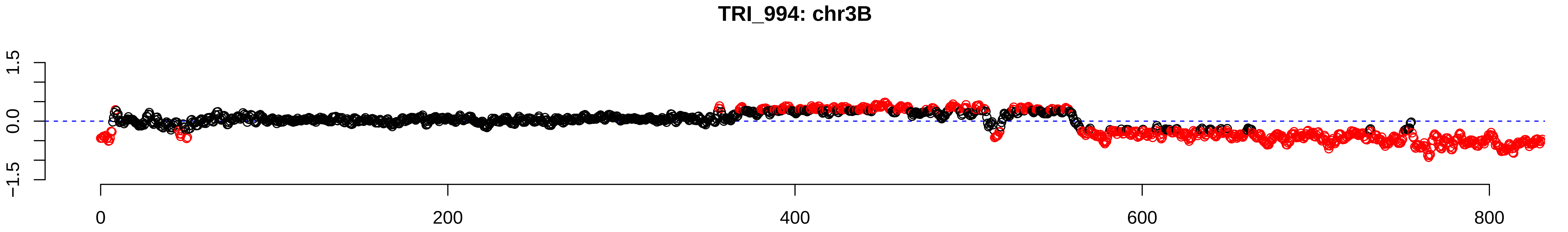
TRI_994: chr3B
1.5
0.0
−1.5
0
200
400
600
800

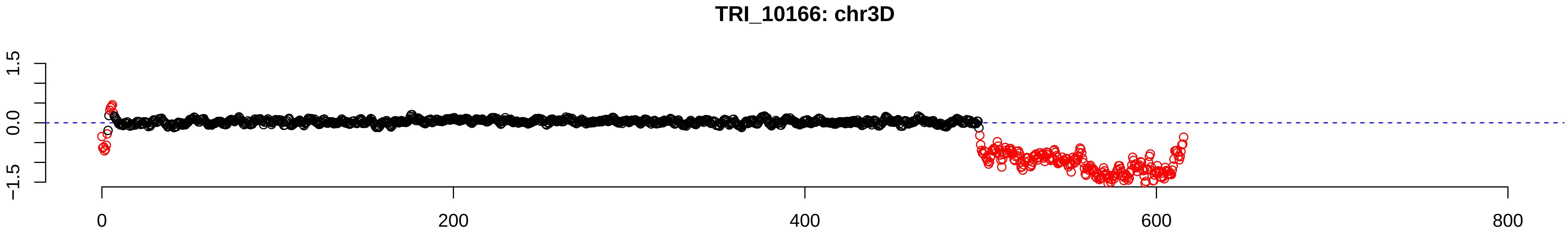

TRI_10166: chr3D
1.5
0.0
−1.5
0
200
400
600
800

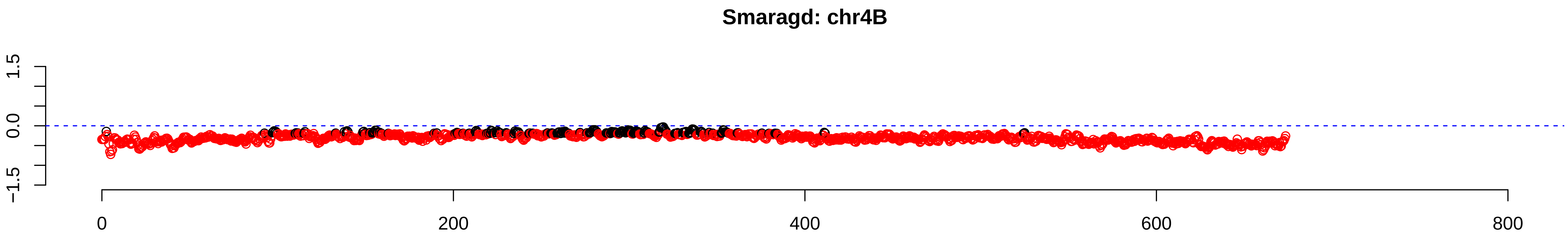
Smaragd: chr4B
1.5
0.0
−1.5
0
200
400
600
800

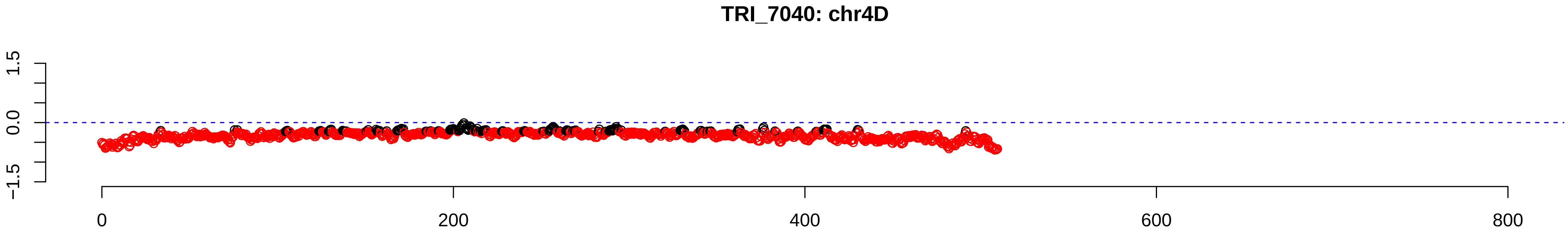

TRI_7040: chr4D
1.5
0.0
−1.5
0
200
400
600
800

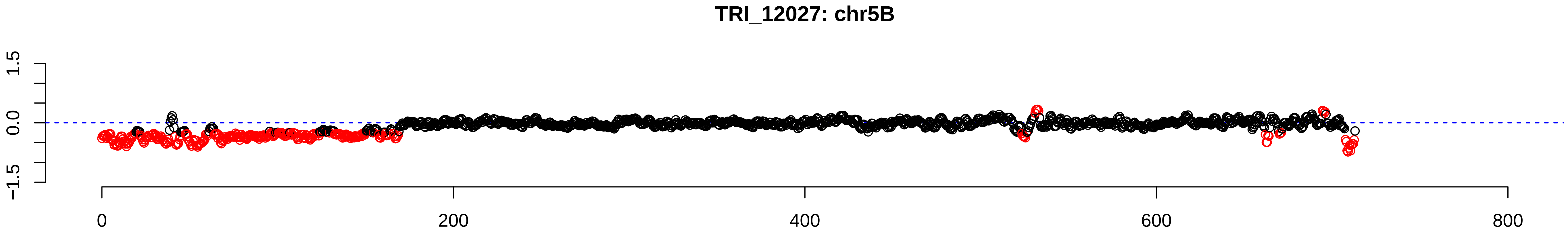

TRI_12027: chr5B
1.5
0.0
−1.5
0
200
400
600
800

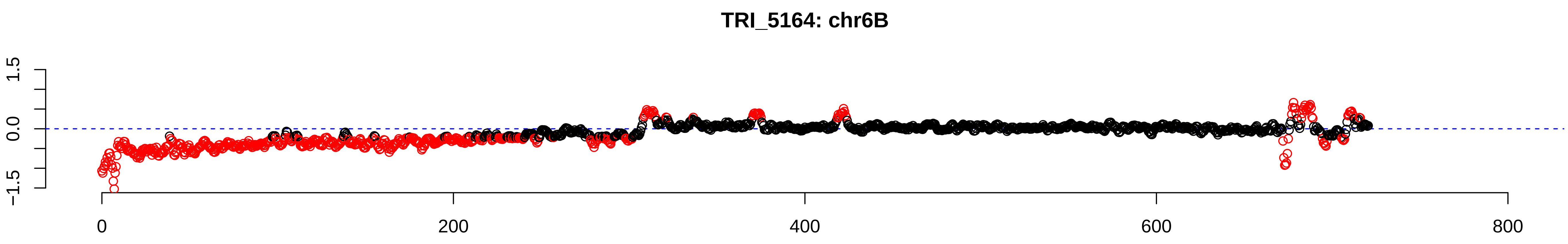
TRI_5164: chr6B
1.5
0.0
−1.5
0
200
400
600
800

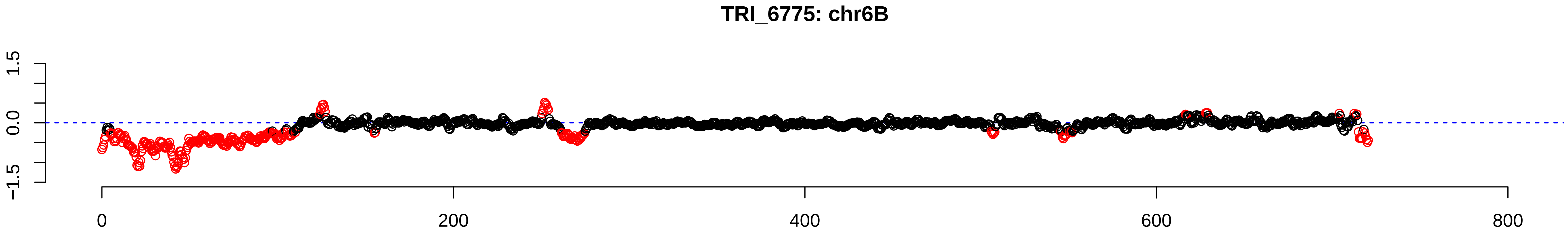
TRI_6775: chr6B
1.5
0.0
−1.5
0
200
400
600
800

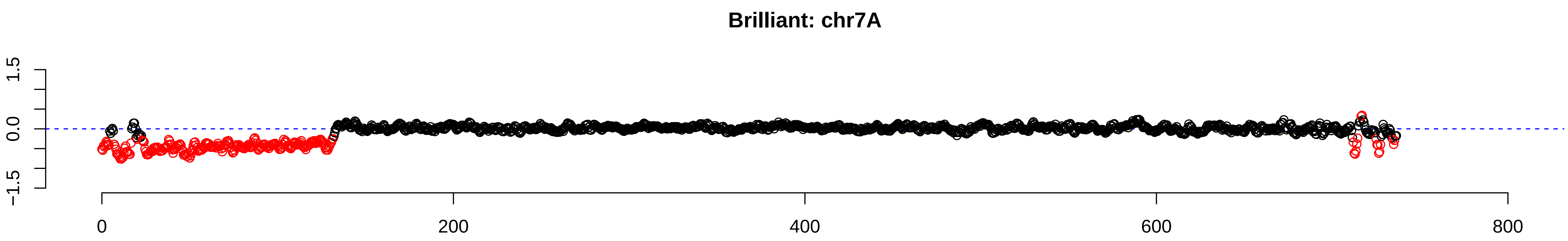

Brilliant: chr7A
1.5
0.0
−1.5
0
200
400
600
800

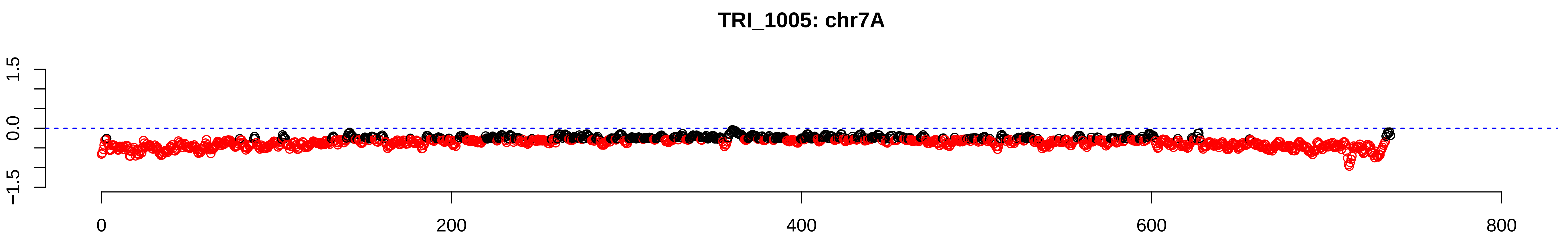
TRI_1005: chr7A
1.5
0.0
−1.5
0
200
400
600
800
