# Supplementary figures and images for: Detecting Large Chromosomal Modifications Using Short Read Data From Genotyping-by-Sequencing
Source: Front Plant Sci. 2019 Sep 24;10:1133. doi: 10.3389/fpls.2019.01133 (PMC6771380; doi:10.3389/fpls.2019.01133)

TRI\_6874: chr1A

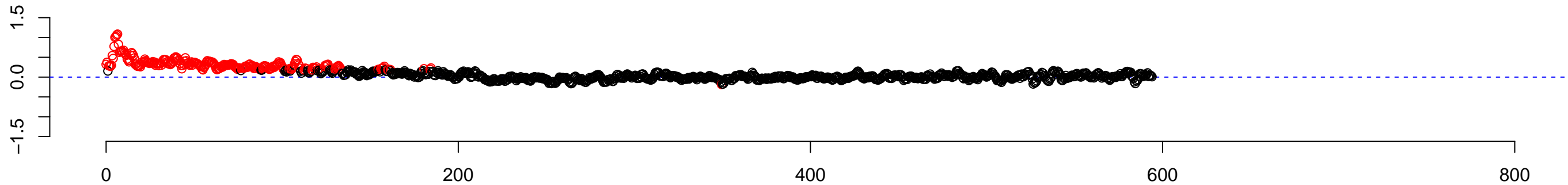

TRI\_5042: chr2D

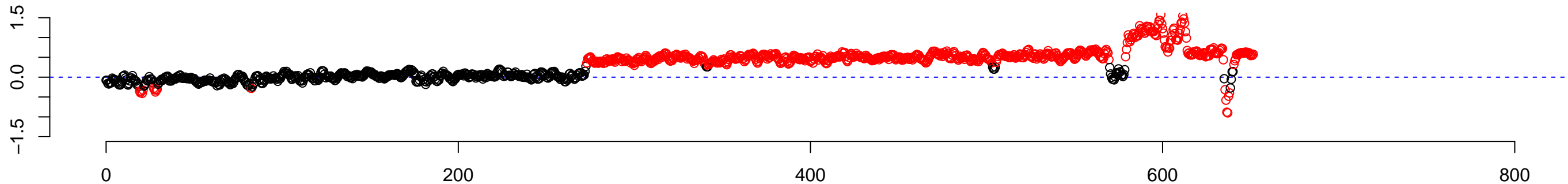

TRI\_7716: chr2D

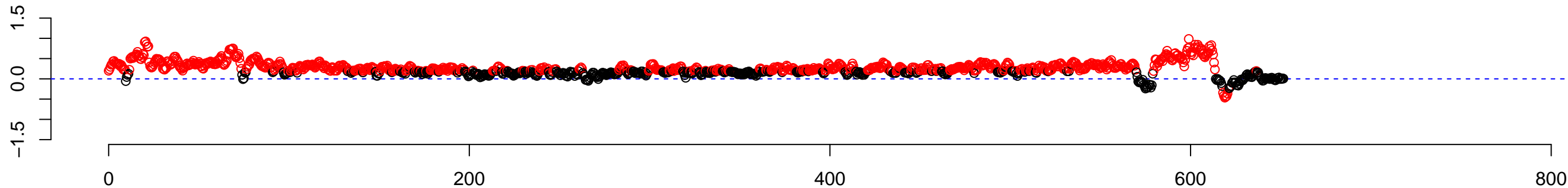

Supplement: Supplemental Data Sheet 3 — Coverage profiles of large chromosomal modifications with increased coverage form selected wheat genotypes. [file DataSheet_3.pdf]
